# 37th International Symposium on Intensive Care and Emergency Medicine (part 1 of 3): Brussels, Belgium. 21–24 March 2017

**DOI:** 10.1186/s13054-017-1628-y

**Published:** 2017-03-21

**Authors:** V. Karavana, I. Smith, G. Kanellis, I. Sigala, T. Kinsella, S. Zakynthinos, L. Liu, J. Chen, X. Zhang, A. Liu, F. Guo, S. Liu, Y. Yang, H. Qiu, D. G. Grimaldi, SR The SRLF Trial Group, E. Kaya, O. Acicbe, I. Kayaalp, S. Asar, M. Dogan, G. Eren, O. Hergunsel, D. Pavelescu, I. Grintescu, L. Mirea, M. Guanziroli, M. Gotti, A. Marino, M. Cressoni, G. Vergani, C. Chiurazzi, D. Chiumello, L. Gattinoni, M. Guanziroli, M. Gotti, G. Vergani, M. Cressoni, C. Chiurazzi, A. Marino, S. Spano, D. Chiumello, L. Gattinoni, M. Guanziroli, M. Gotti, G. Vergani, A. Marino, M. Cressoni, C. Chiurazzi, D. Chiumello, L. Gattinoni, F. Massaro, A. Moustakas, S. Johansson, A. Larsson, G. Perchiazzi, X. W. Zhang, F. M. Guo, J. X. Chen, M. Xue, Y. Yang, H. B. Qiu, J. X. Chen, L. Liu, L. Yang, X. W. Zhang, F. M. Guo, Y. Yang, H. B. Qiu, M. Fister, R. Knafelj, M. A. Suzer, M. E. Kavlak, H. K. Atalan, B. Gucyetmez, N. Cakar, D. Weller, A. F. Grootendorst, A. Dijkstra, T. M. Kuijper, B. I. Cleffken, A. Regli, B. De Keulenaer, P. Van Heerden, D. Hadfield, P. A. Hopkins, B. Penhaligon, F. Reid, N. Hart, G. F. Rafferty, G. Grasselli, T. Mauri, M. Lazzeri, E. Carlesso, B. Cambiaghi, N. Eronia, E. Maffezzini, A. Bronco, C. Abbruzzese, N. Rossi, G. Foti, G. Bellani, A. Pesenti, G. Li Bassi, M. Panigada, O. Ranzani, T. Kolobow, A. Zanella, M. Cressoni, L. Berra, V. Parrini, H. Kandil, G. Salati, S. Livigni, S. Livigni, A. Amatu, M. Girardis, M. Barbagallo, G. Moise, G. Mercurio, A. Costa, A. Vezzani, S. Lindau, J. Babel, M. Cavana, A. Torres, M. Panigada, G. Li Bassi, O. T. Ranzani, T. Kolobow, A. Zanella, M. Cressoni, L. Berra, V. Parrini, H. Kandil, G. Salati, S. Livigni, A. Amatu, M. Girardis, M. Barbagallo, G. Moise, G. Mercurio, A. Costa, A. Vezzani, S. Lindau, J. Babel, M. Cavana, A. Torres, M. Umbrello, M. Taverna, P. Formenti, G. Mistraletti, F. Vetrone, A. Marino, G. Vergani, A. Baisi, D. Chiumello, A. G. Garnero, D. N. Novotni, J. A. Arnal, M. Urner, E. Fan, M. Dres, S. Vorona, L. Brochard, N. D. Ferguson, E. C. Goligher, C. Leung, G. Joynt, W. Wong, A. Lee, C. Gomersall, S. Poels, M. Casaer, M. Schetz, G. Van den Berghe, G. Meyfroidt, B. Holzgraefe, L. B. Von Kobyletzki, A. Larsson, G. Cianchi, F. Becherucci, S. Batacchi, M. Cozzolino, F. Franchi, S. Di Valvasone, M. C. Ferraro, A. Peris, H. Phiphitthanaban, P. Wacharasint, V. Wongsrichanalai, A. Lertamornpong, O. Pengpinij, A. Wattanathum, N. Oer-areemitr, M. Boddi, G. Cianchi, E. Cappellini, M. Ciapetti, S. Batacchi, G. Di Lascio, M. Bonizzoli, M. Cozzolino, A. Peris, C. Lazzeri, G. Cianchi, M. Bonizzoli, G. Di Lascio, M. Cozzolino, A. Peris, M. L. Katsin, M. Y. Hurava, A. M. Dzyadzko, A. Hermann, P. Schellongowski, A. Bojic, K. Riss, O. Robak, W. Lamm, W. Sperr, T. Staudinger, L. Tadini Buoninsegni, M. Bonizzoli, M. Cozzolino, J. Parodo, A. Ottaviano, L. Cecci, E. Corsi, V. Ricca, A. Peris, A. Perez Ruiz de Garibay, B. Ende-Schneider, C. Schreiber, B. Kreymann, F. Turani, M. Resta, D. Niro, P. Castaldi, G. Boscolo, G. Gonsales, S. Martini, A. Belli, L. Zamidei, M. Falco, T. Lamas, J. Mendes, A. Galazzi, T. Mauri, B. Benco, F. Binda, L. Masciopinto, M. Lazzeri, E. Carlesso, A. Lissoni, G. Grasselli, I. Adamini, A. Pesenti, T. Thamjamrassri, J. Watcharotayangul, P. Numthavaj, S. Kongsareepong, J. Higuera, D. Cabestrero, L. Rey, G. Narváez, A. Blandino, M. Aroca, S. Saéz, R. De Pablo, A. Mohamed, M. Sklar, L. Munshi, T. Mauri, M. Lazzeri, L. Alban, C. Turrini, M. Panigada, P. Taccone, E. Carlesso, C. Marenghi, S. Spadaro, G. Grasselli, C. Volta, A. Pesenti, J. Higuera, D. Cabestrero Alonso, A. Blandino, G. Narváez, L. Rey González, M. Aroca, S. Saéz, R. De Pablo, A. Franci, G. Stocchi, G. Cappuccini, F. Socci, M. Cozzolino, C. Guetti, P. Rastrelli, A. Peris, A. Nestorowicz, J. Glapinski, A. Fijalkowska-Nestorowicz, J. Wosko, A. Fijalkowska-Nestorowicz, J. Glapinski, J. Wosko, F. Duprez, T. Bonus, G. Cuvelier, S. Mashayekhi, S. Ollieuz, G. Reychler, T. Bonus, F. Duprez, G. Cuvelier, S. Mashayekhi, S. Ollieuz, G. Reychler, I. Kuchyn, K. Bielka, A. Sergienko, H. Jones, C. Day, S. C. Park, S. R. Yeom, S. N. Myatra, S. Gupta, V. Rajnala, J. Divatia, J. Villalobos Silva, O. Aguilera Olvera, R. Cavazos Schulte, M. Castañeda Bermudez, L. Pariente Zorrilla, H. Lopez Ferretis, K. Trejo García, N. Balciuniene, J. Ramsaite, O. Kriukelyte, A. Krikscionaitiene, T. Tamosuitis, P. Terragni, L. Brazzi, D. Falco, L. Pistidda, G. Magni, L. Bartoletti, L. Mascia, C. Filippini, V. Ranieri, A. Kyriakoudi, N. Rovina, O. Koltsida, E. Konstantellou, M. Kardara, E. Kostakou, G. Gavriilidis, I. Vasileiadis, N. Koulouris, A. Koutsoukou, W. Van Snippenburg, A. Kröner, M. Flim, M. Buise, R. Hemler, P. Spronk, A. Regli, B. Noffsinger, B. De Keulenaer, B. Singh, L. Hockings, P. Van Heerden, C. Spina, A. Bronco, F. Magni, C. Di Giambattista, A. Vargiolu, G. Bellani, G. Foti, G. Citerio, G. Scaramuzzo, S. Spadaro, A. D. Waldmann, S. H. Böhm, R. Ragazzi, C. A. Volta, S. J. Heines, U. Strauch, M. C. Van de Poll, P. M. Roekaerts, D. C. Bergmans, S. Sosio, S. Gatti, E. Maffezzini, V. Punzi, A. Asta, G. Foti, G. Bellani, J. Glapinski, J. Mroczka, A. Nestorowicz, A. Fijalkowska-Nestorowicz, A. I Yaroshetskiy, N. A. Rezepov, I. A. Mandel, B. R. Gelfand, E. Ozen, E. Karakoc, A. Ayyildiz, S. Kara, S. Ekemen, B. Buyukkidan Yelken, W. Saasouh, J. Freeman, A. Turan, Z. Hajjej, W. Sellami, M. Bousselmi, W. Samoud, H. Gharsallah, I. Labbene, M. Ferjani, L. Vetrugno, F. Barbariol, F. Forfori, I. Regeni, G. Della Rocca, D. Jansen, A. Jonkman, J. Doorduin, L. Roesthuis, J. Van der Hoeven, L. Heunks, S. Arrigoni Marocco, M. Bottiroli, R. Pinciroli, V. Galanti, A. Calini, M. Gagliardone, G. Bellani, R. Fumagalli, S. Gatti, C. Abbruzzese, D. Ippolito, V. L. Sala, V. Meroni, A. Bronco, G. Foti, G. Bellani, M. Elbanna, Y. Nassar, A. Abdelmohsen, M. Yahia, S. Mongodi, F. Mojoli, G. Via, G. Tavazzi, F. Fava, M. Pozzi, G. A. Iotti, B. Bouhemad, F. Ruiz-Ferron, J. Serrano Simón, M. Gordillo-Resina, V. Chica-Saez, M. Ruiz Garcia, R. Vela-Colmenero, M. Redondo-Orts, C. Gontijo-Coutinho, T. Ozahata, P. Nocera, D. Franci, T. Santos, M. Carvalho-Filho, O. Fochi, S. Gatti, M. Nacoti, D. Signori, A. Bronco, D. Bonacina, G. Bellani, E. Bonanomi, S. Mongodi, E. Bonvecchio, A. Stella, E. Roldi, A. Orlando, M. Luperto, B. Bouhemad, G. A. Iotti, F. Mojoli, D. Trunfio, G. Licitra, R. Martinelli, D. Vannini, G. Giuliano, L. Vetrugno, F. Forfori, E. Näslund, L. G. Lindberg, I. Lund, A. Larsson, R. Frithiof, A. Nichols, J. Freeman, S. Pentakota, B. Kodali, A. Pranskunas, I. Kiudulaite, J. Simkiene, D. Damanskyte, Z. Pranskuniene, J. Arstikyte, D. Vaitkaitis, V. Pilvinis, M. Brazaitis, R. Pool, H. Haugaa, A. Botero, D. Escobar, D. Maberry, T. Tønnessen, B. Zuckerbraun, M. Pinsky, H. Gomez, H. Lyons, A. Trimmings, R. Domizi, C. Scorcella, E. Damiani, S. Pierantozzi, S. Tondi, V. Monaldi, A. Carletti, S. Zuccari, E. Adrario, P. Pelaia, A. Donati, S. Kazune, A. Grabovskis, K. Volceka, U. Rubins, M. Bol, M. Suverein, T. Delnoij, R. Driessen, S. Heines, T. Delhaas, M. Vd Poll, J. Sels, M. Jozwiak, M. Chambaz, P. Sentenac, C. Richard, X. Monnet, J. L. Teboul, Z. Bitar, O. Maadarani, R. Al Hamdan, W. Huber, M. Malbrain, M. Chew, J. Mallat, T. Tagami, S. Hundeshagen, S. Wolf, W. Huber, S. Mair, R. Schmid, J. Aron, M. Adlam, G. Dua, L. Mu, L. Chen, J. Yoon, G. Clermont, A. Dubrawski, Z. Duhailib, K. Al Assas, A. Shafquat, N. Salahuddin, J. Donaghy, P. Morgan, L. Valeanu, M. Stefan, S. Provenchere, D. Longrois, A. Shaw, M. G. Mythen, D. Shook, D. Hayashida, X. Zhang, S. H. Munson, A. Sawyer, M. Mariyaselvam, M. Blunt, P. Young, N. Nakwan, B. Khwannimit, P. Checharoen, D. Berger, P. Moller, S. Bloechlinger, A. Bloch, S. Jakob, J. Takala, J. M. Van den Brule, R. Stolk, E. Vinke, L. M. Van Loon, P. Pickkers, J. G. Van der Hoeven, M. Kox, C. W. Hoedemaekers, P. Werner-Moller, S. Jakob, J. Takala, D. Berger, P. Bertini, F. Guarracino, D. Colosimo, S. Gonnella, G. Brizzi, G. Mancino, R. Baldassarri, M. R. Pinsky, P. Bertini, S. Gonnella, G. Brizzi, G. Mancino, D. Amitrano, F. Guarracino, T. Goslar, D. Stajer, P. Radsel, R. De Vos, N. Bussink-van Dijk, G. Stringari, G. Cogo, A. Devigili, M. Ceola Graziadei, E. Bresadola, P. Lubli, S. Amella, F. Marani, E. Polati, L. Gottin, L. Colinas, G. Hernández, R. Vicho, M. Serna, A. Canabal, R. Cuena, M. Jozwiak, J. Gimenez, J. L. Teboul, P. Mercado, F. Depret, C. Richard, X. Monnet, Z. Hajjej, W. Sellami, K. Sassi, H. Gharsallah, I. Labbene, M. Ferjani, A. Herner, R. Schmid, W. Huber, N. Abded, Y. Nassar, M. Elghonemi, A. Monir, J. Nikhilesh, T. Apurv, A. U. Uber, A. Grossestreuer, A. Moskowitz, P. Patel, M. J. Holmberg, M. W. Donnino, C. A. Graham, K. Hung, R. Lo, L. Y. Leung, K. H. Lee, C. Y. Yeung, S. Y. Chan, N. Trembach, I. Zabolotskikh, J. Caldas, R. Panerai, L. Camara, G. Ferreira, J. Almeida, G. Queiroz de Oliveira, J. Jardim, E. Bor-Seng-Shu, M. Lima, R. Nogueira, F. Jatene, S. Zeferino, F. Galas, T. Robinson, L. A. Hajjar, J. Caldas, R. Panerai, G. Ferreira, L. Camara, S. Zeferino, J. Jardim, E. Bor-Seng-Shu, M. Oliveira, R. Norgueira, R. Groehs, L. Ferreira-Santos, F. Galas, G. Oliveira, J. Almeida, T. Robinson, F. Jatene, L. Hajjar, G. Ferreira, J. Ribeiro, F. Galas, F. Gaiotto, L. Lisboa, J. Fukushima, S. Rizk, J. Almeida, F. Jatene, E. Osawa, R. Franco, R. Kalil, L. Hajjar, M. Chlabicz, B. Sobkowicz, K. Kaminski, R. Kazimierczyk, W. Musial, A. Tycińska, M. Siranovic, A. Gopcevic, Z. G. Gavranovic, A. H. Horvat, H. Krolo, B. Rode, L. Videc, A. Trifi, S. Abdellatif, K. Ben Ismail, A. Bouattour, F. Daly, R. Nasri, S. Ben Lakhal, A. Beurton, J. L. Teboul, V. Girotto, L. Galarza, C. Richard, X. Monnet, A. Beurton, J. L. Teboul, V. Girotto, L. Galarza, C. Richard, X. Monnet, V. Girotto, J. L. Teboul, A. Beurton, L. Galarza, T. Guedj, X. Monnet, L. Galarza, P. Mercado, J. L. Teboul, V. Girotto, A. Beurton, C. Richard, X. Monnet, M. Karaman Iliæ, L. Sakic, V. NN, L. Stojcic, M. Jozwiak, F. Depret, J. L. Teboul, J. Alphonsine, C. Lai, C. Richard, X. Monnet, N. Tapanwong, P. Chuntupama, P. Wacharasint, W. Huber, J. Hoellthaler, T. Lahmer, R. Schmid, H. Latham, C. D. Bengtson, L. Satterwhite, M. Stites, S. Q. Simpson, H. Latham, C. D. Bengtson, L. Satterwhite, M. Stites, S. Q. Simpson, T. Skladzien, M. Cicio, J. Garlicki, W. Serednicki, J. Wordliczek, P. Vargas, A. Salazar, P. Mercado, M. Espinoza, J. Graf, N. Kongpolprom, N. Sanguanwong, S. Jonnada, C. Gerrard, N. Jones, T. Morley, P. T. Thorburn, A. Trimmings, T. Musaeva, I. Zabolotskikh, A. Salazar, P. Vargas, P. Mercado, M. Espinoza, J. Graf, S. Horst, M. Lipcsey, R. Kawati, A. Pikwer, J. Rasmusson, M. Castegren, A. Shilova, A. Yafarova, M. Gilyarov, A. Shilova, A. Yafarova, M. Gilyarov, D. L. Loncar Stojiljkovic, A. Ulici, S. Reidt, T. Lam, J. Jancik, D. Ragab, K. Taema, W. Farouk, M. Saad, X. Liu, M. J. Holmberg, A. Uber, S. Montissol, M. Donnino, L. W. Andersen, F. Perlikos, M. Lagiou, A. Papalois, C. Kroupis, I. Toumpoulis, E. Osawa, D. Carter, S. Sardo, J. Almeida, F. Galas, S. Rizk, R. Franco, L. Hajjar, G. Landoni, S. Kongsayreepong, R. Sungsiri, P. Wongsripunetit, P. Marchio, S. Guerra-Ojeda, M. Gimeno-Raga, M. D. Mauricio, S. L. Valles, C. Aldasoro, A. Jorda, M. Aldasoro, J. M. Vila, U. B. Borg, A. M. Neitenbach, M. García, P. Guijo González, M. Gracia Romero, P. Saludes Orduña, A. Gil Cano, A. Rhodes, R. M. Grounds, M. Cecconi, C. Lee, F. Hatib, Z. Jian, J. Rinehart, J. De Los Santos, C. Canales, M. Cannesson, M. I. Monge García, F. Hatib, Z. Jian, T. Scheeren, Z. Jian, F. Hatib, M. Pinsky, V. Chantziara, A. Vassi, G. Michaloudis, E. Sanidas, S. Golemati, R. M. Bateman, A. Mokhtar, W. Omar, K. Abdel Aziz, H. El Azizy, D. L. Lykke Nielsen, J. G. Holler, A. Lassen, M. Eriksson, G. Strandberg, M. Lipcsey, A. Larsson, C. Capoletto, J. Almeida, G. Ferreira, J. Fukushima, R. Nakamura, S. Risk, E. Osawa, C. Park, G. Oliveira, F. Galas, R. Franco, L. Hajjar, F. Dias, N. D’Arrigo, F. Fortuna, S. Redaelli, L. Zerman, L. Becker, T. Serrano, L. Cotes, F. Ramos, L. Fadel, F. Coelho, C. Mendes, J. Real, B. Pedron, M. Kuroki, E. Costa, L. Azevedo

**Affiliations:** 1 George P. Livanos and Marianthi Simou Laboratories, Athens, Greece; 2 Rigel Pharmaceuticals, Inc, South San Francisco, CA USA; 3 Evangelismos Hospital, Athens, Greece; 4 Department of Critical Care Medicine, Nanjing Zhongda Hospital, School of Medicine, Southeast University, Nanjing, China; 5 Hôpital Erasme, Brussels, Belgium; 6 SRLF, Paris, France; 7 Bakirkoy Dr.Sadi Konuk Training and Research Hospital, Istanbul, Turkey; 8 Emergency Hospital Floreasca, Bucharest, Romania; 9 Università degli Studi di Milano, Milano, Italy; 10 ASST Santi Paolo e Carlo, Milano, Italy; 11 Humanitas, Rozzano, Italy; 12 University of Gottingen, Gottingen, Germany; 13 Università degli Studi di Milano, Milano, Italy; 14 ASST Santi Paolo e Carlo, Milano, Italy; 15 Humanitas, Rozzano, Italy; 16 University of Gottingen, Gottingen, Germany; 17 Università degli Studi di Milano, Milano, Italy; 18 ASST Santi Paolo e Carlo, Milano, Italy; 19 Humanitas, Rozzano, Italy; 20 University of Gottingen, Gottingen, Germany; 21 Policlinico di Bari, Bari, Italy; 22 Uppsala University Biomedical Center, Uppsala, Sweden; 23 Akademiska Sjukhuset, Uppsala University, Uppsala, Sweden; 24 Department of Critical Care Medicine, Nanjing Zhongda Hospital, School of Medicine, Southeast University, Nanjing, China; 25 Department of Critical Care Medicine, Nanjing Zhongda Hospital, School of Medicine, Southeast University, Nanjing, China; 26 Rihard Knafelj, Ljubljana, Slovenia; 27 Cankaya Hospital, Ankara, Turkey; 28 Atasehir Memorial Hospital, Istanbul, Turkey; 29 Acibadem Fulya Hospital, Istanbul, Turkey; 30 Acibadem University School of Medicine, Istanbul, Turkey; 31 Maasstad Hospital, Rotterdam, Netherlands; 32 Fiona Stanley Hospital, Perth, Australia; 33 Hadassah University Hospital, Jerusalem, Israel; 34 King’s College London, London, UK; 35 King’s College Hospital, London, UK; 36 Guy’s and St Thomas’ Hospital, London, UK; 37 Fondazione IRCCS Ca’ Granda Ospedale Maggiore Policlinico, Milan, Italy; 38 University of Milan, Milan, Italy; 39 University of Ferrara, Ferrara, Italy; 40 University of Milan-Bicocca, Monza, Italy; 41 Hospital Clinic, Barcelona, Spain; 42 Policlinico di Milano, Milan, Italy; 43 National Institutes of Health, Bethesda, USA; 44 Ospedale San Paolo, Milan, Italy; 45 Massachusetts General Hospital, Boston, USA; 46 Ospedale Nuovo del Mugello, Borgo San Lorenzo, Italy; 47 Gruppo Ospedaliero San Donato, San Donato Milanese, Italy; 48 Arcispedale S. Maria Nuova, Reggio Emilia, Italy; 49 Ospedale San Giovanni Bosco, Torino, Italy; 50 Policlinico San Matteo, Pavia, Italy; 51 Policlinico di Modena, Modena, Italy; 52 Azienda Ospedaliero-Universitaria di Parma, Parma, Italy; 53 Ospedale Citta di Sesto San Giovanni, Sesto San Giovanni, Italy; 54 Policlinico Gemelli, Roma, Italy; 55 University Hospital Frankfurt, Frankfurt, Germany; 56 University Hospital Zagreb, Zagreb, Croatia; 57 Ospedale Santa Chiara, Trento, Italy; 58 Policlinico di Milano, Milan, Italy; 59 Hospital Clinic, Barcelona, Spain; 60 National Institutes of Health, Bethesda, USA; 61 Ospedale San Paolo, Milan, Italy; 62 Massachussets General Hospital, Boston, USA; 63 Ospedale Nuovo del Mugello, Borgo San Lorenzo, Italy; 64 Gruppo Ospedaliero San Donato, San Donato Milanese, Italy; 65 Arcispedale S. Maria Nuova, Reggio Emilia, Italy; 66 Ospedale San Giovanni Bosco, Torino, Italy; 67 Policlinico San Matteo, Pavia, Italy; 68 Policlinico di Modena, Modena, Italy; 69 Azienda Ospedaliero-Universitaria di Parma, Parma, Italy; 70 Ospedale di Sesto San Giovanni, Sesto San Giovanni, Italy; 71 Policlinico Gemelli, Rome, Italy; 72 University Hospital Frankfurt, Frankfurt, Germany; 73 University Hospital Center Zagreb, Zagreb, Croatia; 74 Ospedale Santa Chiara, Trento, Italy; 75 Ospedale San Paolo, Università degli Studi di Milano, Milano, Italy; 76 Hôpital Sainte Musse, Toulon, France; 77 Hamilton medical, Bonaduz, Switzerland; 78 University of Toronto, Toronto, Canada; 79 Chinese University of Hong Kong, Sha Tin, NT Hong Kong; 80 UZ Leuven, Leuven, Belgium; 81 Karolinska Institutet, Stockholm, Sweden; 82 Lund University, Karlstad University, Lund, Sweden; 83 Uppsala University, Uppsala, Sweden; 84 Careggi Teaching Hospital, Florence, Italy; 85 University of Siena, Siena, Italy; 86 Phramonkutklao hospital, Bangkok, Thailand; 87 Careggi Teaching Hospital, Florence, Italy; 88 Careggi Teaching Hospital, Florence, Italy; 89 Republican Scientific and Practical Center for Organ and Tissue Transplantation, Minsk, Belarus; 90 Medical University of Vienna, Vienna, Austria; 91 Careggi Teaching Hospital, Florence, Italy; 92 Hepa Wash GmbH, Munich, Germany; 93 Aurelia and European Hospital, Rome, Italy; 94 Istituti clinici S Donato MI, Milan, Italy; 95 Marino Hospital, Cagliari, Italy; 96 Ospedale dell’ Angelo, Mestre, Italy; 97 Santo Stefano Hospital, Prato, Italy; 98 Egas Moniz Hosp., Lisboa, Portugal; 99 Fernando da Fonseca Hosp., Lisboa, Portugal; 100 Fondazione IRCCS Ca’ Granda Ospedale Maggiore Policlinico, University of Milan, Milano, Italy; 101 Sant’Anna Hospital, University of Ferrara, Ferrara, Italy; 102 Mahidol University, Bangkok, Thailand; 103 Ramón y Cajal University Hospital, Madrid, Spain; 104 Sinai Health System, Toronto, Canada; 105 University of Milan, Milan, Italy; 106 University of Ferrara, Ferrara, Italy; 107 Fondazione IRCCS Ca’ Granda Ospedale Maggiore Policlinico, Milan, Italy; 108 Ramón y Cajal University Hospital, Madrid, Spain; 109 Careggi Teaching Hospital, Florence, Italy; 110 Medical University, Lublin, Poland; 111 Wroclaw University of Technology, Wroclaw, Poland; 112 Medical University, Lublin, Poland; 113 SPSK No4, Lublin, Poland; 114 Medical University, Lublin, Poland; 115 Wroc3aw University of Technology, Wroclaw, Poland; 116 SPSK No4, Lublin, Poland; 117 Epicura, Hornu, Belgium; 118 Condorcet, Tournai, Belgium; 119 UCL, Bruxelles, Belgium; 120 Epicura, Hornu, Belgium; 121 Condorcet, Tournai, Belgium; 122 UCL, Bruxelles, Belgium; 123 Institute of Postgraduate Education Bogomolets National Medical University, Kyiv, Ukraine; 124 Plymouth hospitals NHS Trust, Plymouth, UK; 125 Royal Devon and Exeter Hospital, Exeter, UK; 126 Pusan National University Hospital, Busan, South Korea; 127 Tata Memorial Hospital, Mumbai, India; 128 Hospital General “Norberto Treviño”, CD. Victoria, Mexico; 129 Hospital Infantil de Tamaulipas, Cd. Victoria, Mexico; 130 Lithuanian University of Health Sciences, Kaunas, Lithuania; 131 University of Sassari, Sassari, Italy; 132 Città della Salute e della Scienza Torino, Torino, Italy; 133 Sapienza University of Rome Policlinico Umberto I Hospital, Roma, Italy; 134 ICU, 1st Department of Pulmonary Medicine, “Sotiria” Hospital, Athens Medical School, Athens, Greece; 135 Gelre Hospitals Apeldoorn, Apeldoorn, Netherlands; 136 Catharina Hospital Eindhoven, Eindhoven, Netherlands; 137 Fiona Stanley Hospital, Perth, Australia; 138 SCGH, Perth, Australia; 139 The Alfred Hospital, Melbourne, Australia; 140 Hadassah University Hospital, Jerusalem, Israel; 141 University of Milano Bicocca, Monza, Italy; 142 San Gerardo Hospital, Monza, Italy; 143 University of Ferrara, Ferrara, Italy; 144 Swisstom AG, Landquart, Switzerland; 145 University Hospital Maastricht, Maastricht, Netherlands; 146 University of Milano Bicocca, Milan, Italy; 147 Wroclaw University of Science and Technology, Wrocław, Poland; 148 Lublin Medical Univesity, Lublin, Poland; 149 Pirogov Russian National Research Medical University, Moscow, Russia; 150 City Hospital#67, Moscow, Russia; 151 Federal research and clinical center for special methods of healthcare and medical technology of FMBA, Moscow, Russia; 152 Eskisehir Osmangazi Uni. Faculty of Medicine, Eskisehir, Turkey; 153 Cleveland Clinic Foundation, Cleveland, OH USA; 154 Respiratory Motion, Inc, Waltham, MA USA; 155 Military Hospital of Tunis, Tunis, Tunisia; 156 University-Hospital, Udine, Italy; 157 University-Hospital of Pisa, Pisa, Italy; 158 Radboudumc, Nijmegen, Netherlands; 159 VU Medical Center, Amsterdam, Netherlands; 160 Milano Niguarda, Milano, Italy; 161 Ospedale San Gerardo, Monza, Italy; 162 University of Milano-Bicocca, Milano, Italy; 163 Fondazione IRCCS Ca’ Granda Ospedale Maggiore Policlinico, Milano, Italy; 164 San Gerardo Hospital, Monza, Italy; 165 Hospital San Gerardo, Monza, Italy; 166 University of Milano-Bicocca, San Gerardo Hospital, Monza, Italy; 167 Cairo University, Giza, Egypt; 168 Fondazione IRCCS Policlinico S. Matteo, University of Pavia, Pavia, Italy; 169 CHU Dijon, Dijon, France; 170 Complejo hospitalario de Jaen, Jaen, Spain; 171 Hospital Reina Sofía, Cordoba, Spain; 172 Unicamp, Campinas, Brazil; 173 Papa Giovanni XXIII Hospital, Bergamo, Italy; 174 University of Milano-Bicocca, Milano, Italy; 175 San Gerardo Hospital, Monza, Italy; 176 Università degli studi Milano Bicocca, San Gerardo Hospital, Monza, Italy; 177 Fondazione IRCCS Policlinico S. Matteo, University of Pavia, Pavia, Italy; 178 CHU Dijon, Dijon, France; 179 Univesity Hosptial, Pisa, Italy; 180 Univesity anesthesia and intensive care unit, University of Pisa, PISA, Italy; 181 Azienda Ospedaliero Universitaria di Udine, Udine, Italy; 182 Karolinska Institutet, Stockholm, Sweden; 183 Linköping University, Linköping, Sweden; 184 Uppsala University, Uppsala, Sweden; 185 Brigham & Women’s Hospital, Boston, MA USA; 186 Respiratory Motion, Inc, Waltham, MA USA; 187 Lithuanian university of health sciences, Kaunas, Lithuania; 188 Lithuanian Sports University, Kaunas, Lithuania, Kaunas Lithuania; 189 University of Pittsburgh, Pittsburgh, PA USA; 190 Oslo University Hospital, Oslo, Norway; 191 Staten Island University Hospital, New York, NY USA; 192 Bronx-Lebanon Hospital, New York, NY USA; 193 East Sussex Healthcare, East Sussex, UK; 194 Università Politecnica delle Marche, Ancona, Italy; 195 Hospital of Traumatology and Orthopaedics, Riga, Latvia; 196 University of Latvia, Riga, Latvia; 197 MUMC, Netherlands, Netherlands; 198 Hôpitaux universitaires Paris-Sud, Hôpital de Bicêtre, Inserm UMR S_999, Univ Paris-Sud, Le Kremlin-Bicêtre, France; 199 Hôpitaux universitaires Paris-Sud, Hôpital de Bicêtre, Inserm UMR S_999, Univ Paris-Sud, Le Kremlin-Bicêtre, France; 200 zouheir bitar, Fahahil, Kuwait; 201 Klinikum rechts der Isar, Technical University of Munich, Munich, Germany; 202 University Hospital, Ghent, Belgium; 203 Centre Hospitalier, Lens, France; 204 Nagayama Hospital, Tokyo, Japan; 205 Charité, Campus Virchow-Klinikum, Berlin, Germany; 206 Klinikum rechts der Isar, Technical University of Munich, Munich, Germany; 207 St Georges Hospital, London, UK; 208 GSTT, London, UK; 209 Carnegie Mellon University, Pittsburgh, PA USA; 210 University of Pittsburgh, Pittsburgh, PA USA; 211 Zainab Al Duhailib, Riyadh, Saudi Arabia; 212 East Surrey Hospital, Redhill, UK; 213 Hopital Bichat, Paris, France; 214 Vanderbilt University Medical Center, Nashville, TN USA; 215 UCL, London, UK; 216 Brigham and Womens Hospital, Boston, MA USA; 217 Boston Strategic Partners, Boston, MA USA; 218 Queen Elizabeth Hospital, Kings Lynn, UK; 219 Division of Critical Care Medicine, Hat Yai, Thailand; 220 Division of Cardiology, Prince of Songkla University, Hat Yai, Thailand; 221 Inselspital, Bern University Hospital, University of Bern, Bern, Switzerland; 222 Institute of Clinical Sciences at the Sahlgrenska Academy, University of Gothenburg, Sahlgrenska University Hospital, Gothenburg, Sweden; 223 Radboud UMC, Nijmegen, Netherlands; 224 Inselspital, University Hospital of Bern, Bern, Switzerland; 225 Azienda Ospedaliero Universitaria Pisana, Pisa, Italy; 226 Meyer Children Hospital, Florence, Italy; 227 University of Pittsburgh, Pittsburgh, PA USA; 228 Azienda Ospedaliero Universitaria Pisana, Pisa, Italy; 229 University center Ljubljana, Ljubljana, Slovenia; 230 Academic hospital Maastricht, Maastricht, Netherlands; 231 AOUI Verona, Verona, Italy; 232 Virgen de la Salud Hospital, Madrid, Spain; 233 Quironsalud Palmaplanas Hospital, Palma de Mallorca, Spain; 234 Marina Salud Denia Hospital, Denia, Spain; 235 Hôpitaux universitaires Paris-Sud, Hôpital de Bicêtre, Inserm UMR S_999, Univ Paris-Sud, Le Kremlin-Bicêtre, France; 236 Military Hospital of Tunis, Tunis, Tunisia; 237 Klinikum rechts der Isar; Technical University of Munich, Munich, Germany; 238 University of Cairo, Cairo, Egypt; 239 CHL Hospitals, Indore, India; 240 Beth Israel Deaconess Medical Center, Boston, MA USA; 241 The Chinese University of Hong Kong, Hong Kong, Hong Kong; 242 Prince of Wales Hospital, Hong Kong, Hong Kong; 243 Kuban State Medical University, Krasnodar, Russia; 244 University of Sao Paulo, Sao Paulo, Brazil; 245 University of Leicester, Leicester, UK; 246 University of Sao Paulo, Sao Paulo, Brazil; 247 University of Leicester, Leicester, UK; 248 University of Sao Paulo, Brazil, Sao Paulo, Brazil; 249 Medical University of Bialystok, Bialystok, Poland; 250 Clinical hospital center, Zagreb, Croatia; 251 University hospital center of La Rabta, Tunis, Tunisia; 252 Hôpital de Bicêtre, Le Kremlin Bicêtre, France; 253 Hôpital de Bicêtre, Hôpitaux universitaires Paris-Sud, Assistance publique – Hôpitaux de Paris, Le kremlin bicêtre, France; 254 Hôpital de Bicêtre, Le Kremlin Bicêtre, France; 255 Hôpital de Bicêtre, Le Kremlin-Bicêtre, France; 256 Service de réanimation médicale, Inserm UMR S_999, Université Paris-Sud, Le Kremlin-Bicêtre, France; 257 Hôpital de Bicêtre, Hôpitaux universitaires Paris-Sud, Assistance publique – Hôpitaux de Paris, Le Kremlin-Bicêtre, France; 258 Hôpital de Bicêtre, Le Kremlin-Bicêtre, France; 259 Clinical Hospital Sveti Duh, Faculty of Medicine, University of Osijek, Zagreb, Croatia; 260 University of Zagreb, School of Medicine, Zagreb, Croatia; 261 Hôpitaux universitaires Paris-Sud, Hôpital de Bicêtre, Inserm UMR S_999, Univ Paris-Sud, Le Kremlin-Bicêtre, France; 262 Phramongkutklao Hospital, Bangkok, Thailand; 263 Klinikum rechts der Isar; Technical University of Munich, Munich, Germany; 264 University of Kansas Medical Center, Kansas City, Kansas USA; 265 University of Kansas Medical Center, Kansas City, Kansas USA; 266 Szpital Uniwersytecki, Cracow, Poland; 267 Clinica Alemana, Santiago, Chile; 268 Chulalongkorn University, Bangkok, Thailand; 269 University of Cambridge, Cambridge, UK; 270 Papworth Hospital, Papworth, UK; 271 East Sussex Healthcare NHS Trust, Eastbourne, UK; 272 Kuban State Medical University, Krasnodar, Russia; 273 Clinica Alermana, Santiago, Chile; 274 Department of Surgical Sciences, Uppsala, Sweden; 275 Eskilstuna County Hospital, Eskilstuna, Sweden; 276 Gävle County Hospital, Gävle, Sweden; 277 Karolinska University Hospital Solna, Stockholm, Sweden; 278 Moscow Clinical City Hospital #1 named after N. Pirogov, Moscow, Russia; 279 Moscow Clinical City Hospital #1 named after N. Pirogov, Moscow, Russia; 280 Special Gynecological Hospital, Jevremova, 11000 Serbia; 281 Hennepin County Medical Center, Minneapolis, MN USA; 282 Cairo University, Cairo, Egypt; 283 Beth Israel Deaconess Medical Center, Boston, MA USA; 284 Evangelismos Hospital, Athens, Greece; 285 Attikon Hospital, Athens, Greece; 286 ELPEN Research and Experimental Center, Athens, Greece; 287 Department of Cardiac Surgery, Attikon Hospital, Athens, Greece; 288 Heart Institute, Sao Paulo, Brazil; 289 San Raffaele, Milan, Italy; 290 Siriraj Hospital, Mahidol University, Bangkok, Thailand; 291 University of Valencia, Valencia, Spain; 292 Medtronic, Boulder, CO USA; 293 St. George’s Healthcare NHS Trust and St George’s University of London, London, UK; 294 Hospital SAS de Jerez, Jerez de la Frontera, Spain; 295 Edwards Lifesciences, Irvine, CA USA; 296 UC Irvine School of Medicine, Irvine, CA USA; 297 UCLA David Geffen School of Medicine, CA Los Angeles, USA; 298 Hospital SAS de Jerez, Jerez de la Frontera, Spain; 299 Edwards Lifesciences, Irvine, CA USA; 300 University Medical Center Groningen, Groningen, Netherlands; 301 Edwards Lifesciences, Irvine, CA USA; 302 University of Pittsburgh, Pittsburgh, PA USA; 303 Saint Savvas Hospital, Athens, Greece; 304 Cardiology Dep, Laiko General Hospital, Athens, Greece; 305 Medical School, National Kapodistrian University of Athens, Athens, Greece; 306 University of Western Ontario, London, Canada; 307 Salam International Hospital, Cairo, Egypt; 308 Cairo University Hospital, Cairo, Egypt; 309 Odense University Hospital, Odense C, Denmark; 310 Surgical Sciences, Uppsala, Sweden; 311 Medical Sciences, Uppsala, Sweden; 312 Cancer Institute of the University of Sao Paulo, Sao Paulo, Brazil; 313 Hospital Pompeia, Caxias do Sul, Brazil; 314 Research and Education Institute, Hospital Sirio-Libanes, São Paulo, Brazil

## P1 Inhibition of jak/stat3 signaling pathway by small molecule r548 prevents inflammation in experimental murine lung injury model

### V. Karavana^1^, I. Smith^2^, G. Kanellis^3^, I. Sigala^1^, T. Kinsella^2^, S. Zakynthinos^1^

#### ^1^George P. Livanos and Marianthi Simou Laboratories, Athens, Greece; ^2^Rigel Pharmaceuticals, Inc, South San Francisco, CA, USA; ^3^Evangelismos Hospital, Athens, Greece

**Introduction:** Aberrant inflammation is a hallmark of acute respiratory distress syndrome (ARDS) pathophysiology. JAK/STAT3 pathway is critical for macrophages and neutrophils activation and persistent inflammation. This study aims to investigate the therapeutic potential of inhibiting JAK1/3 activity using the small-molecule R548 inhibitor in LPS induce lung injury model.

**Methods:** Lung injury was induced in adult male C57BL/6 mice, by intratracheal LPS administration followed by post subcutaneous injection of R548 inhibitor R548 inhibitor, prodrug for the active compound, R507 (Rigel Pharmaceuticals Inc.). Mice sacrificed at 6 h and 24 h after LPS administration. Lung inflammation was examined by protein content, number and type of inflammatory cells in bronchoalveolar lavage fluid (BALF). Protein expression levels of JAK1, p-STAT3, ERK1/2 were analysed by Western blotting.

**Results:** LPS administration increased BALF cellularity, total protein content, and neutrophils cells number at both 6 h and 24 h. Elevated levels of JAK1, p-STAT3 and ERK1/2 protein expression were observed. In addition, post LPS treatment with the JAK 1/3 inhibitor significantly reduces BALF protein content (P < 0.05), total cells number (P < 0.01), neutrophils cells number (P < 0.01) as early as 6 h. Moreover, R548 treatment decreased JAK1 protein expression by 2 fold (P < 0.01) and p-STAT3 levels by 2.7 fold (P < 0.001) below the LPS group.

**Conclusions:** These data suggest the JAK/STAT3 signaling pathway plays a critical role in ARDS mediated lung inflammation and injury. Additional studies are warranted to further investigate JAK/STAT3 inhibition as a therapeutic treatment for this serious and life threatening disease.

## P2 Stable genetic alterations of CXCR7 regulate the CXCL12/CXCR7 axis, a new "passport" ± for the homing of mscs

### L. Liu, J. Chen, X. Zhang, A. Liu, F. Guo, S. Liu, Y. Yang, H. Qiu

#### Department of Critical Care Medicine, Nanjing Zhongda Hospital, School of Medicine, Southeast University, Nanjing, China

**Introduction:** Mesenchymal stem cells (MSCs) have several properties that make them attractive therapeutic candidates for treatment of acute disease, but in vivo homing, MSCs does not appear to be highly efficient. CXCL12/CXCR7 axis not only improves the motility of stem cells, but also regulates many essential biological processes. The aim of this study is to evaluate the effects of overexpressing or suppressing CXCR7 on proliferation and migration abilities of mice mesenchymal stem cells.

**Methods:** The lentivirus vector overexpressing and suppressing the murine CXCR7 gene was transducted into mMSCs. The transfection efficiency of LV in passage 20 transduced-mMSCs was identified using fluorescence microscopy, and the percentage of ZsGreen positive cells was determined by flow cytometry analysis (FCM). CXCR7 mRNA expression in mMSCs was verified by quantitative real-time PCR, and CXCR7 protein expression was analyzed by FCM. The effect of CXCR7 on the migration of mMSCs was evaluated using the scratch assay and the transwell migration assay. In transwell assay, 50 ng/ml CXCL12 was added in two groups to simulation inflammation microenvironments. The ELISA assay was used to detect the concentration of VCAM-1, CXCL12 the supernatant.

**Results:** The efficiencies of the lentiviral vector transduction of MSC-OE-CXCR7 (overexpression of CXCR7), MSC-OENC-CXCR7 (normal control of overexpression of CXCR7), MSC-Sh-CXCR7 (suppression of CXCR7) and MSC-ShNC-CXCR7 (normal control of suppression of CXCR7) after 20 passages in mMSCs were 91.29%, 91.39%, 91.69% and 91.28% respectively. The CXCR7 mRNA and protein expression were significantly higher in the MSC-OE-CXCR7 cells than in the MSC-OENC-CXCR7 cells, and were significantly lower in the MSC-Sh-CXCR7 when compared with the MSC-ShNC-CXCR7. Moreover, CXCR7 gene overexpression promoted mMSCs migration. In contrast, the suppression of CXCR7 inhibited mMSCs migration when compared with the MSC-ShNC-CXCR7 group. Overexpression CXCR7 increased MSC-secreted CXCL12 and VCAM-1, which contributed to the improvement of mMSCs homing.

**Conclusions:** Overexpression CXCR7 improved the homing abilities of mMSCs, which might attribute mainly to increasement of CXCL12 and VCAM-1 secreted by mMSCs.

**Fig. 1 (abstract P2).**
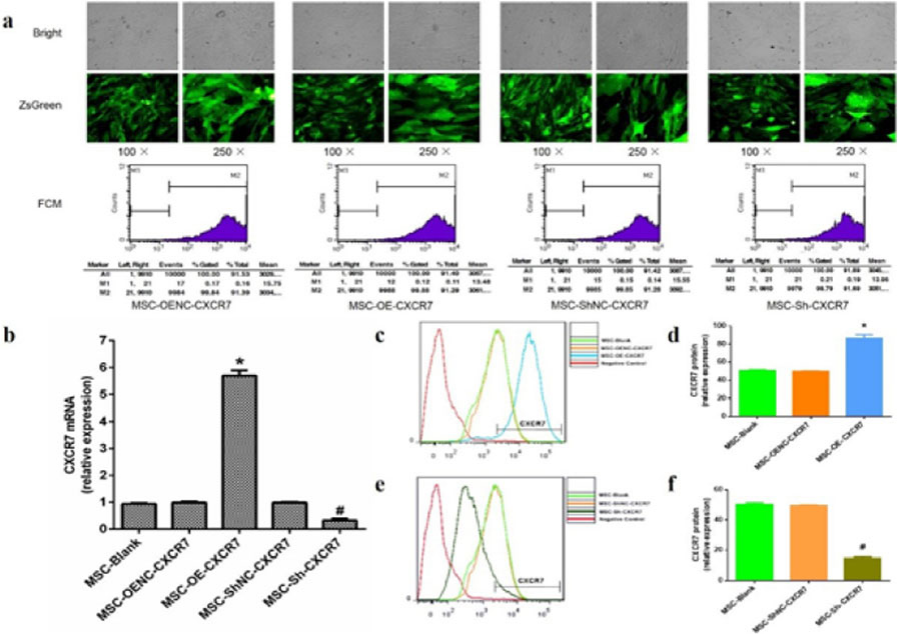
Legend 1: Figure 1 Long-term transgene expression efficiency in mMSCs after lentiviral vector transduction

**Fig. 2 (abstract P2).**
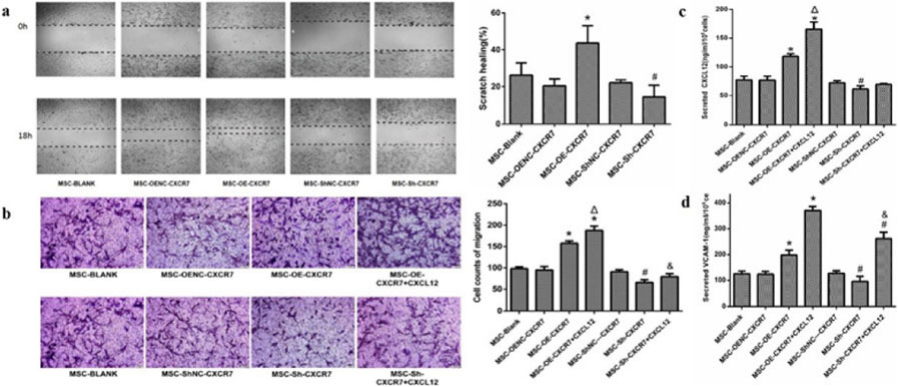
Legend 2: Figure 2 The effect of CXCR7 on the migration of mMSCs. The concentration of VCAM-1 and CXCL12 in the supernatant of Transwell Chambers was examined using ELISA assay.

## P3 Withdrawn

## P4 Spectrum study severe hypoxemia: prevalence, treatment and outcome in 2016

### D. G. Grimaldi^1^, SR The SRLF Trial Group^2^

#### ^1^Hôpital Erasme, Brussels, Belgium; ^2^SRLF, Paris, France

**Introduction:** Limited information exists about the prevalence, subsequent management, and outcomes of hypoxemia among ICU patients.

The aim of the study was to assess the prevalence of hypoxemia among ICU patients and to stratify them according to the severity of hypoxemia (PaO2/FIO2 between 300 and 201 in mild, between 200 and 101 in moderate, and <101 mm Hg in severe). Management and outcomes of hypoxemic patients and the proportion of them who met the criteria for acute respiratory distress syndrome (ARDS) were analyzed

**Methods:** This study was an international, multicenter, 1-day point prevalence study conducted in 117 units during the spring of 2016. All patients already hospitalized or newly admitted the day of the study were susceptible to be enrolled.

Hypoxemia was defined as a P/F ratio of 300 mmHg or less

**Results:** Of 1604 patients included, 859 (54%) were hypoxemic, 440 (51%) were mildly, 345 (40%) moderately and 74 (9%) severely hypoxemic. Among hypoxemic patients, 183 (21%) had ARDS (37% mild, 46% moderate and 16% severe). Characteristics of patients according to hypoxemia severity are reported in the Table 1. Pneumonia was the main cause of hypoxemia (53%) and of ARDS (79%). Modalities of oxygen treatment vari.

Among hypoxemic patients under invasive mechanical ventilation, 77% received a tidal volume of 8 mL/kg or less of predicted body weight with a median PEEP level of 6 [5–10] cmH2O. Among ARDS patients, 145 (83%) received a tidal volume of 8 mL/kg or less with a median PEEP of 8 [6–12] cmH2O and a median plateau pressure of 23 [19–27] cmH2O.

In the whole population, median ICU length of stay was 12 days [5–28] and the ICU mortality was 20%. In the hypoxemic population, median ICU length of stay appeared to be higher (16 [7–32] days) and further increased in ARDS patients (20 [11–38] days, P < 0.001). ICU mortality was 27% in hypoxemic patients (21% in mild hypoxemic patients, 26% in moderate hypoxemic patients, and 50% in severe hypoxemic patients, P < 0.001)

**Conclusions:** Hypoxemia affected more than half of the patients in ICU, whereas only 21% of hypoxemic patients met ARDS criteria. Mortality was significantly associated with severity of hypoxemia.

**Table 1 (abstract P4).** See text for description

	All hypoxemic n = 859	Mild hypoxemia n = 440	Moderate hypoxemia n = 345	Severe hypoxemia n = 74	P value
Age	64 (53–73)	65 (54–74)	65 (53–73)	58 (47–69)	0.006
Sex	586 (68)	298 (68)	236 (68)	52 (70)	0.91
Medical patients	675 (79)	327 (75)	281 (82)	67 (91)	0.004
SAPS-2	43 (31–57)	42 (30–56)	43 (31–57)	45 (34–61)	0.32
ARDS (Berlin definition)	183 (21)	68 (16)	85 (25)	30 (41)	<0.001
Invasive ventilation	525 (61)	239 (54)	231 (67)	55 (74)	0.001
ICU length of stay	16 (7–32)	15 (7–32)	16 (8–31)	18 (7–37)	0.89
ICU mortality	225 (27)	92 (21)	96 (27)	37 (50)	<0.00

Legend Table 1: Patients’ characteristics according to hypoxemia severity

## P5 Correlation of horowitz ratio with oxygen saturation index in ARDS

### E. Kaya, O. Acicbe, I. Kayaalp, S. Asar, M. Dogan, G. Eren, O. Hergunsel

#### Bakirkoy Dr. Sadi Konuk Training and Research Hospital, Istanbul, Turkey

**Introduction:** In definition of ARDS PaO2 and PaO2/FiO2 (Horowitz) ratio are the most reliable parameters used. In this study it is aimed to investigate the correlation of Horowitz ratio with oxygenation saturation index (OSI) derived from SpO2 instead of PaO2.

**Methods:** Demographical data of 307 patients treated with mechanical ventilation in our ICU between 01.01.2014–31.12.2015 due to respiratory failure were revised from iMDsoft (Metavision) system together with FiO2, SpO2, PaO2, Paw parameters. Oxygenation saturation index was calculated with the formula using [FiO2 x Paw]/SpO2. ROC (receiver operator characteristic) analysis was used to search for the relation of Horowitz ratio and OSI.

**Results:** The median age of the patients were 58,5 years (IQR 18–96). For the patients with Horowitz ratio < 100 cut-off value for OSI was found to be 7,0768 (sensitivity 94.4% and specificity 86.9%). OSI cut-off value was 3,7178 for Horowitz ratio < 200 (sensitivity 90.2% and specificity 79%). For those with Horowitz ratio < 300, OSI cut-off value was calculated as 2,561 (sensitivity 83.3% and specificity 72%).

**Conclusions:** OSI was defined for ARDS diagnosis in pediatric population which includes the parameter of mean airway pressure in its formula rendering this index a more objective parameter for ventilatory support instead of PaO2/FiO2 ratio. It was a big controversy before Berlin definition that ARDS definition was relying on cirteria which do not directly consider the extent of ventiatory support on oxygenation, however there is still a debate that Berlin definition did not bring a solution in that aspect. Therefore, we think that this study, which we aim to reveal the OSI values that correlate with Horowitz values of mild, moderate and severe ARDS, should be supplemented with other studies and validated by further prospective studies, considering OSI as a reflection of positive pressure changes affecting oxygenation in ARDS.

**References**

1. Thomas NJ, et al. Pediatr Crit Care Med. 2010; 11(1): 12–17

2. Rotta AT, et al. Rev Bras Ter Intensiva. 2015;27(3):266–273

## P6 Ards features of the severe burn patients from colective tragedy

### D. Pavelescu, I. Grintescu, L. Mirea

#### Emergency Hospital Floreasca, Bucharest, Romania

**Introduction:** ARDS is an independent risk factor for death in burn patients, with appreciable mortality of 26–58%. More than 30% of thermal injured patients have a concomitant smoke inhalation injury

**Methods:** A retrospective observational study which include 14 young patients 21–36 y.o. with severe burns to the face, neck, chest, extremities, admitted in the ICU of level I trauma Center after a deadly fire in Bucharest in an enclosed space with air temperature in 60 seconds between 900–1500 degrees C, in which 64 people were killed and 147 severely injured.

All of them had signs and symptoms of acute hypoxia requiring mechanical ventilation.

We asses the incidence of smoke inhalation injury, the CO toxicity, the etiology, the development time, the relation between severity of ARDS and % of BSA, the mortality

**Results:** All of them have smoke inhalation injury and CO toxicity, the etiology was multifactorial (trauma, multiple transfusion, sepsis, smoke injury, resuscitated cardio-respiratory arrest), there was a strong correlation between the severity of ARDS and the % of Burn Surface Area, the mortality was extremely high

**Conclusions:** The severity of burn associated with inhalatory injury dramatically increase the incidence of ARDS and despite new promising therapeutic interventions, ARDS still remain a devastating disease in severe burn patients.

**Fig. 3: (abstract P6).**
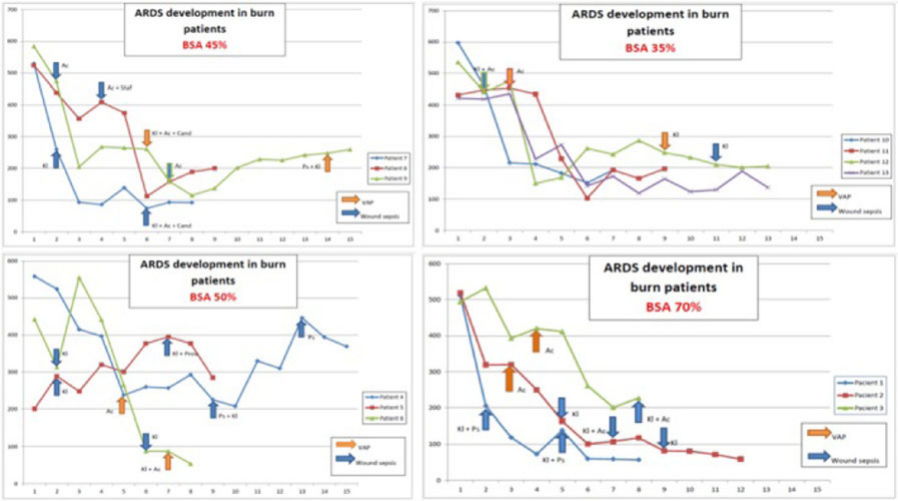
See text for description

## P7 Relationship between energy load and lung regional inflation status in ARDS patients: a CT scan study

### M. Guanziroli^1^, M. Gotti^2^, A. Marino^1^, M. Cressoni^1^, G. Vergani^1^, C. Chiurazzi^3^, D. Chiumello^1^, L. Gattinoni^4^

#### ^1^Università degli Studi di Milano, Milano, Italy; ^2^ASST Santi Paolo e Carlo, Milano, Italy; ^3^Humanitas, Rozzano, Italy; ^4^University of Gottingen, Gottingen, Germany

**Introduction:** Tidal volume, pressure and flow are components of the energy load (EL). We investigated the relationship between intra-tidal lung inflation status variation and EL in mechanically ventilated ARDS patients.

**Methods:** Twenty-eight ARDS patients underwent end-inspiratory and end-expiratory low dose CT scans at PEEP 5 and 15 cmH2O, maintaining the same tidal volume 7 ± 1.8 mL/kg and respiratory rate 15 ± 4.2 breaths/min. Quantitative CT scan analysis was performed to obtain the amount of not, poorly, well and over inflated tissue (g). EL was computed as the area between the inspiratory limb of pressure-volume curve and the y axis, summed to the energy needed to inflate the PEEP volume. EL at PEEP 5 and 15 cmH2O was then normalized by the End-Expiratory Lung Volume at 5 cmH2O (EELV5).

**Fig. 4 (abstract P7).**
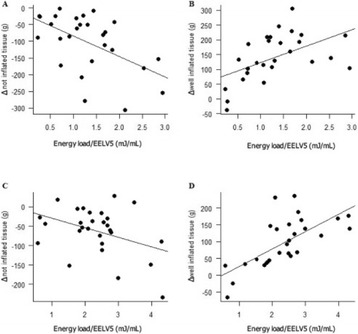
**a** and **b**: PEEP 5 cmh_2_O; **c** and **d** 15 cmh_2_O

**Results:** EL/EELV5 (mJ/mL) is lower at PEEP 5 than 15 cmH2O (1.3 ± 0.73 and 2.4 ± 0.95, p < 0.001). Higher EL/EELV5, respectively for PEEP 5 and 15 cmH2O, is associated with a decrease in not inflated tissue (Δ not inflated tissue = −22.87–61.38*EL/EELV5, r2 = 0.28, p < 0.0001 and Δ not inflated tissue = −4.35–24.59*EL/EELV5, r2 = 0.15, p < 0.01) and with an increase in well inflated tissue (Δ well inflated tissue = 67.72 + 55.91*EL/EELV5, r2 = 0.27, p < 0.0001 and Δ well inflated tissue = −25.13 + 51.91*EL/EELV5, r2 = 0.46, p < 0.0001) at the end of inspiration (Fig. 4). No relationships were found between poorly or over inflated tissue and total energy load.

**Conclusions:** Higher PEEP is associated to higher EL. Higher EL, increasing lung stress and intra-tidal opening and closing, could be unprotective.

## P8 Relationship between total or lung superimposed pressure and absolute esophageal pressure in ARDS: a CT scan study

### M. Guanziroli^1^, M. Gotti^2^, G. Vergani^1^, M. Cressoni^1^, C. Chiurazzi^3^, A. Marino^1^, S. Spano^1^, D. Chiumello^1^, L Gattinoni^4^

#### ^1^Università degli Studi di Milano, Milano, Italy; ^2^ASST Santi Paolo e Carlo, Milano, Italy; ^3^Humanitas, Rozzano, Italy; ^4^University of Gottingen, Gottingen, Germany

**Introduction:** In ARDS patients lung collapse is due to lung superimposed pressure (SP) [1].

SP seems to change as a function of pleural pressure, and both these pressures change as a function of the sterno-vertebral level [2].

Absolute esophageal pressure (Pes) reflects mid-lung pleural pressure, according to the level where the esophageal balloon is placed [1]. However, Pes is unrelated to all morphological indexes of disease severity (not inflated tissue, lung weight, functional residual capacity) and is unrelated to maximal SP [1].

Our aim is to verify if Pes is related to the total and/or the lung SP at the esophageal catheter balloon level.

**Methods:** Ninety-two ARDS patients underwent an end-expiratory CT scan at PEEP 5 cmH2O. Quantitative CT scan analysis was performed to compute the total and the lung SP at the esophageal catheter balloon level in the slice above the diaphragm.

Pes was recorded at PEEP 5 cmH2O.

**Fig. 5 (abstract P8).**
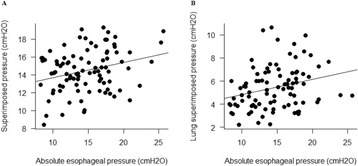
See text for description

**Results:** The total and the lung SP and Pes at PEEP 5 cmH2O are weakly related, respectively (Fig. 5):

- total SP (cmH2O) = 11.97 + 0.17 * Pes (cmH2O), r2 = 0.08, p < 0.01
- lung SP (cmH2O) = 3.47 + 0.13 * Pes (cmH2O), r2 = 0.07, p = 0.01

As shown, Pes is greater than either the total and the lung SP at the level of the esophageal balloon.

**Conclusions:** If the increased total or lung SP is the main mechanism for lung collapse in ARDS patients and PEEP keeps the lung open mainly by counter-balancing the SP, Pes could not be used to select the best PEEP.

**References**

[1] Pelosi P. Am J Respir Crit Care Med 2001; 164:122–130

[2] Bottino N. Crit Care 2000; 4:P115

## P9 Relationship between energy load and lung inhomogeneity in ARDS patients: a CT scan study

### M. Guanziroli^1^, M. Gotti^2^, G. Vergani^1^, A. Marino^1^, M. Cressoni^1^, C. Chiurazzi^3^, D. Chiumello^1^, L. Gattinoni^4^

#### ^1^Università degli Studi di Milano, Milano, Italy; ^2^ASST Santi Paolo e Carlo, Milano, Italy; ^3^Humanitas, Rozzano, Italy; ^4^University of Gottingen, Gottingen, Germany

**Introduction:** In ARDS patients, lung is inhomogeneous [1]. Energy load (EL) describes the interaction between the ventilator and the respiratory system [2]. We investigated the relationship between intra-tidal lung inhomogeneity variation and EL in ARDS patients.

**Methods:** Twenty-eight ARDS patients underwent a series of end-inspiratory and end-expiratory low dose CT scans at 2 different levels of PEEP, 5 and 15 cmH2O, maintaining the same tidal volume 7 ± 1.8 mL/kg and respiratory rate 15 ± 4.2 breaths/min.

Lung inhomogeneity extent is defined as the fraction of lung volume presenting an inflation ratio greater than 1.61 (95th percentile of homogeneous lungs) [1].

The airway pressure-volume curve at the 2 ventilatory settings was obtained. EL was computed as the area between the inspiratory limb of that curve and the volume axis, summed to the energy needed to inflate the PEEP volume [2].

**Fig. 6 (abstract P9).**
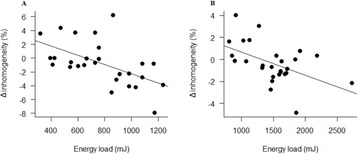
**a** PEEP 5 cmH_2_O; **a** PEEP 15 cmH_2_O

**Results:** At both PEEP levels, lung inhomogeneity variation (end inspiration - end expiration) is related to total EL (Fig. 6):

- PEEP 5: Δ inhomogeneity(%) = 4.38–0.01*EL(mJ), r2 = 0.31, p < 0.001
- PEEP 15: Δ inhomogeneity(%) = 2.78–0.002*EL(mJ), r2 = 0.26, p < 0.0001.

**Conclusions:** During mechanical ventilation, the EL applied to the respiratory system is spent to increase homogeneity of the lung parenchyma. We could speculated that EL is spent at the interfaces between regions with different inflation status.

**References**

[1] Cressoni M. Am J Respir Crit Care Med 2014; 189:149

[2] Gattinoni L. Intensive Care Med 2016; 42:1567

## P10 Does galunisertib reduce edema in experimental ARDS? An exploratory study

### F. Massaro^1^, A. Moustakas^2^, S. Johansson^2^, A. Larsson^3^, G. Perchiazzi^3^

#### ^1^Policlinico di Bari, Bari, Italy; ^2^Uppsala University Biomedical Center, Uppsala, Sweden; ^3^Akademiska Sjukhuset, Uppsala University, Uppsala, Sweden

**Introduction:** The transforming growth factor β (TGF-β) pathway is activated in experimental ARDS and is associated with increased pulmonary edema (1) because it reduces transepithelial sodium transport by inducing endocytosis of ENaC (the epithelial sodium channel). In this way TGF-β decreases the osmotic gradient for re-absorption of water from the alveolar and interstitial spaces (2). We hypothesized that blocking TGF-β signaling with Galunisertib (LY2157299 monohydrate, a TGFβ R1 kinase inhibitor) would reduce edema and tested this in a porcine model of ARDS using aerosolized Galunisertib.

**Methods:** Five piglets (25–30 kg) were randomized to receive (n = 3) or not (n = 2) Galunisertib 50 μM by aerosol. The animals were anesthetized, tracheotomized and ventilated in volume controlled mode (VT: 8 ml/kg, FiO2: 70%, RR:30, PEEP:5 cmH2O, I:E = 1:2). Cardiac output and extravascular lung water (EVLW) were continuously measured (PiCCO, Pulsion Medical System). We measured pressure/volume curves and arterial pH, paO2, paCO2, HCO3- at baseline, immediately after induction of ARDS and then hourly for 6 h. ARDS was induced by repeated lung lavages and injurious ventilation. After the establishment of the ARDS model, Galunisertib was administered and the experiment was continued for 6 h, after which the animals were sacrificed. Histological samples from lungs, heart, kidney and liver were taken. Activation of the TGF-β pathway was immunohistochemically evaluated using an antibody against phosphorylated Smad2 (Small Mother Against Decapentaplegic) and activation of mechanical signaling in the lungs was evaluated using an antibody against phosphorylated FAK (Focal Adhesion Kinase). Moreover, we measured the wet/dry weight ratio of the lung samples.

**Results:** Two animals did not survive the experiment. The sample size is too small to draw any strong conclusion. However, we could not find any major difference in EVLW, wet/dry ratio, blood gas parameters or in lung mechanics between the two groups during the tested time period. However, the lung samples from the treated group showed a lower degree of TGF-β pathway activation, while the control samples from liver or heart showed no influence by Galunisertib.

**Conclusions:** We cannot prove or disprove that TGF-β blockade reduces pulmonary edema associated with experimental ARDS. However, the TGF-β blocker seemed to reach its target as indicated by reduced activation of the TGF-β pathway in the lungs, suggesting that aerosol administration is feasible.

**References**

1. Acta Anaesthesiol Scand. 2016;60(1):79–92,

2. Proc Natl Acad Sci USA. 2014;111(3):E374-83

## P11 Stable over expression of p130 and E2F4 makes a difference on multipotential differentiation in bone marrow derived MSCs

### X. W. Zhang, F. M. Guo, J. X. Chen, M. Xue, Y. Yang, H. B. Qiu

#### Department of Critical Care Medicine, Nanjing Zhongda Hospital, School of Medicine, Southeast University, Nanjing, China

**Introduction:** Bone marrow derived mesenchymal stem cells (BMSCs) are proved to have potential therapeutic effects in ARDS models. However, the mechanism of differentiation in MSCs is still unknown. There is growing evidence suggesting p130 or E2F4 plays an important role in regulating cellular differentiation. Our aim is to evaluate the role of p130/E2F4 in regulating the differentiation of mouse MSCs (mMSCs).

**Methods:** mMSCs with p130 or E2F4 overexpression were constructed using lentiviral vectors. The transfection efficiency of mMSCs was identified using fluorescence microscopy, and the percentage of GFP positive cells was determined by flow cytometry analysis. The mRNA and protein levels in MSC-p130 (overexpressing p130) and MSC-E2F4 (overexpressing E2F4) were detected by qRT-PCR and Western blotting assay, respectively. The effect of p130/E2F4 on the multipotential differentiation abilities of mMSCs were evaluated by adipogenesis, osteoblastic and chondrogenic differentiation medium respectively. The expression of the osteogenic gene OSX, adipogenisis gene PPAR-¦Ã and chondrogenic gene Sox9 measured by qRT-PCR were used to evaluate the differentiation of each group of mMSCs treated with differentiation induction media.

**Results:** The transduction efficiencies mediated by the lentiviral vectors were 80.3–84.4% and were maintained over 20 passages of mMSCs. The p130 or E2F4 mRNA expression was significantly higher in the MSC-p130 and MSC-E2F4 cells than in the MSC-NC cells. Similar results for p130 or E2F4 protein expression were also observed in Western blotting. Moreover, p130/E2F4 gene overexpression promoted the differentiation of mMSCs into osteoblasts, while inhibiting adipogenesis and chondrogenic differentiation of mMSCs.

**Conclusions:** The successful construction of stable and long-term mMSCs lines with overexpressing p130/E2F4 change the multipotential differentiation of mMSCs.

**References**

1. Liu AR et al.: J Cell Physiol 2013; 228: 1270.

**Fig. 7 (abstract P11).**
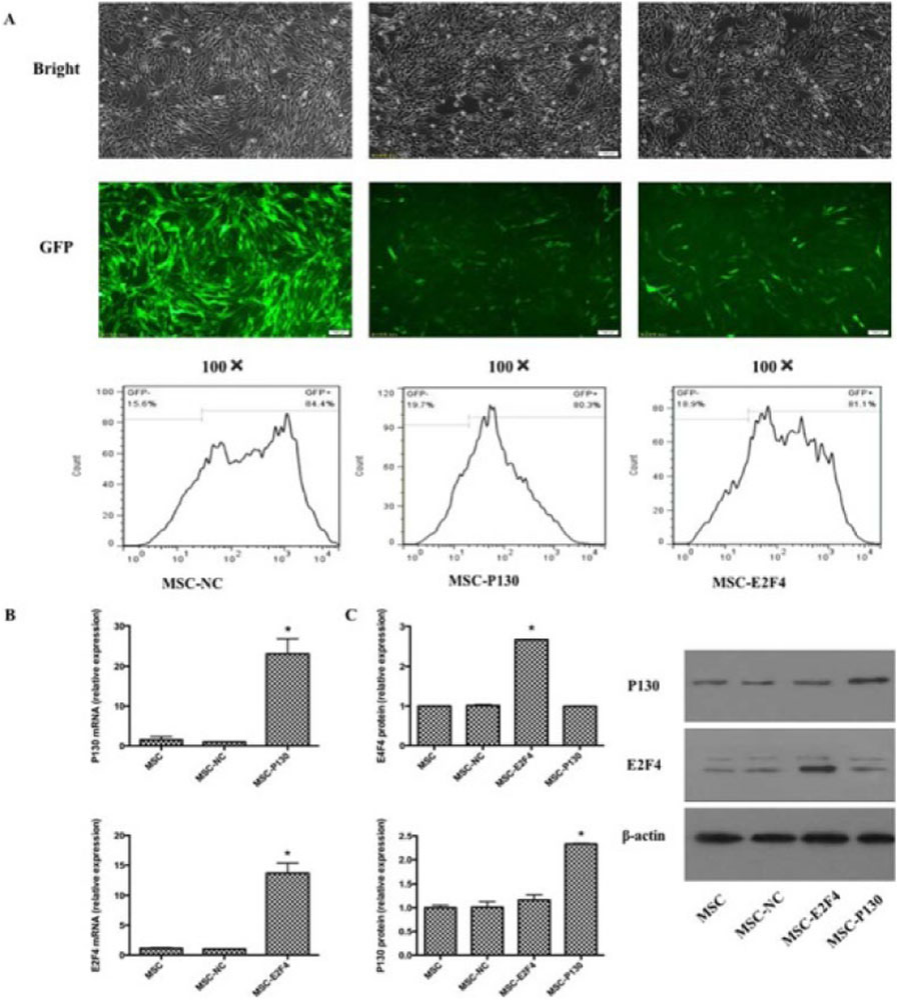
Legend 1: Long-term transgene expression efficiency in mMSCs

**Fig. 8 (abstract P11).**
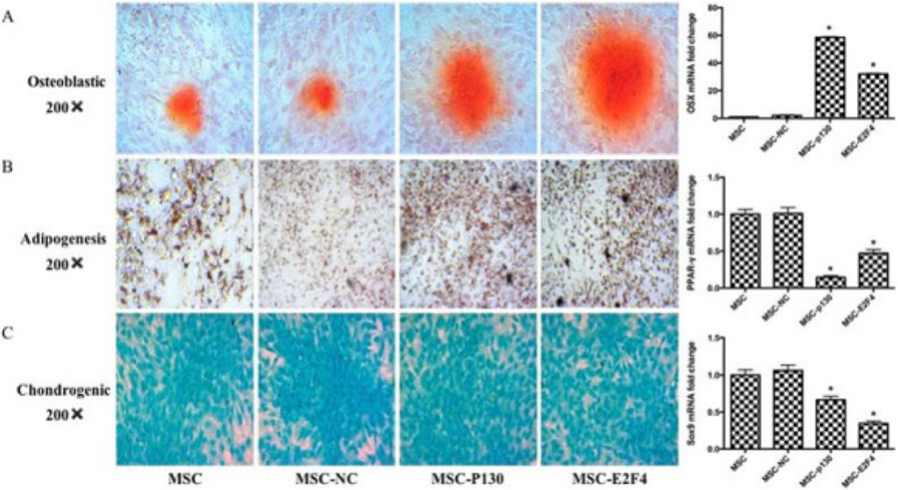
Legend 2: The effect of p130 or E2F4 on multipotential differentiation of mMSCs

## P12 The homing and protective effects of mesenchymal stem cells overexpressing CXCR7 in LPS-induced acute respiratory distress syndrome mice

### J. X. Chen, L. Liu, L. Yang, X. W. Zhang, F. M. Guo, Y. Yang, H. B. Qiu

#### Department of Critical Care Medicine, Nanjing Zhongda Hospital, School of Medicine, Southeast University, Nanjing, China

**Introduction:** The low efficiency of homing to injured organ in mesenchymal stem cells (MSCs) has become the bottleneck for the treatment of acute respiratory distress syndrome (ARDS). In a previous in vitro study, the results showed that the CXCL12/CXCR7 axis promoted the migration of MSCs. It is hypothesized that MSCs overexpressing CXCR7 could further benefits LPS-induced ARDS mice.

**Methods:** mMSCs were transfected with CXCR7 by a lentiviral vector and were transplanted into mice following LPS-induced intratracheal lung injury. Histopathology with haematoxylin and eosin staining and lung injury scoring was performed to evaluate lung tissue injury. Homing of mMSCs were assayed by NIR815 fluorescence imaging and immunofluorescent staining.

**Fig. 9 (abstract P12).**
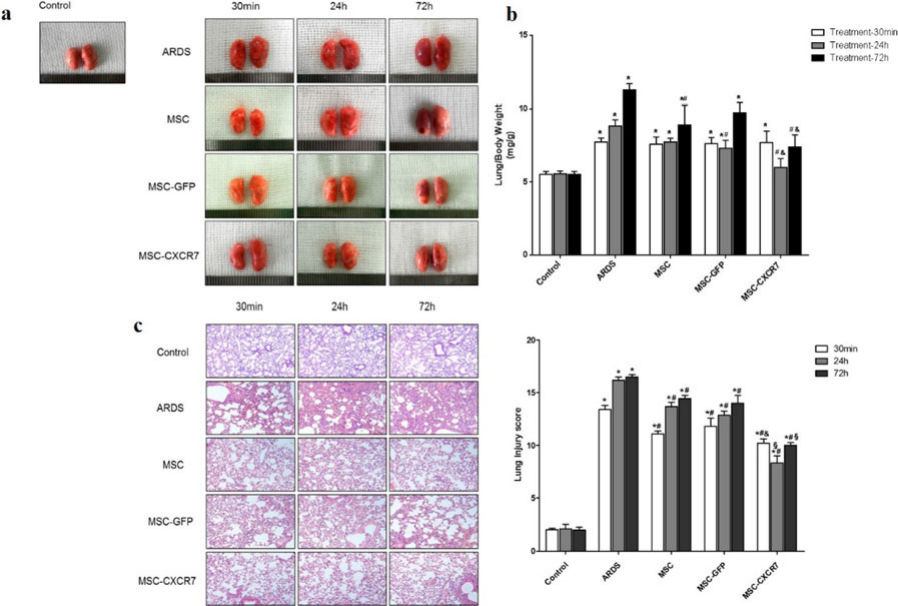
Legend: Histopathology of the effects of MSC-GFP or MSC-CXCR7 in LPS-induced lung injury

**Fig. 10 (abstract P12).**
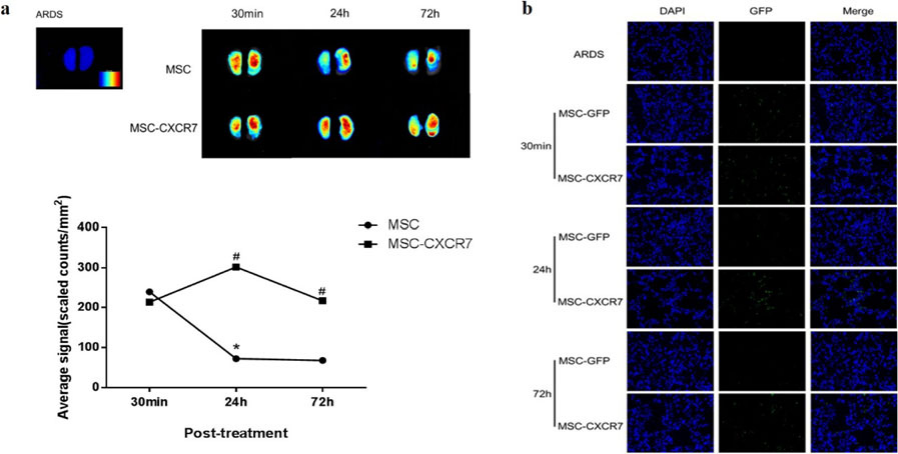
Legend: Effect of overexpressing CXCR7 on the retention of mMSC in the lung after LPS change.

**Results:** The administration of MSC, MSC-GFP and MSC-CXCR7 all improved both lung histopathology (Fig. 9) and lung injury score. Moreover, the MSC-CXCR7 showed lower lung/body weight and lung injury score especially at 24 h compared with MSC-GFP. NIR fluorescence imaging and immunofluorescent staining showed that overexpressing CXCR7 significantly increased the accumulation of mMSCs in the lung especially at 24 h compared to MSC (Fig. 10).

**Conclusion:** It is demonstrated that overexpressing CXCR7 in mMSCs could further promote their homing to injured lung and improve the pathological damage in ARDS mice.

## P13 Independent lung ventilation (ilv) in ICU-forgotten, neglected or useless?

### M. Fister, R. Knafelj

#### Rihard Knafelj, Ljubljana, Slovenia

**Introduction:** ILV remains valuable rescue therapy for refractory hypoxemia in patients with predominantly single sided lung injury (contusion, aspiration, hemorrhage, bronchopleural fistula) especially in centers with no extracorporeal membrane oxygenation (ECMO). Special intubation technique, double lumen endotracehal tube and 2 ventilators are needed.

**Methods:** Single center prospective observational study. Characteristics of consecutive patients assigned to ILV from 2010–2016 are reported.

**Results:** Total 8 patients underwent ILV during observed period (0,9% of all ventilated patients). All patients had paO2/FiO2 < 200, all were ventilated in pressure modes (P-AC, BiLEVEL), ventilators were not synchronized - PEEP, RR, FiO2 and Vt were set individually for each ventilator following blood gas results. Recruitment manuvers and NO were used at physician discretion. In 5 patients oxygenation improved significantly within 2 hours of ILV. 2 patients were upgraded to V-V ECMO (Wegener’s, pneumonia), in 4 contraindication to ECMO was present. 3 patients died (lung carcinoma, emphysema, Wegener’s). All tube placements were confirmed radiologically, 1 intubation with double lumen tube was performed via bougie and proper placement confirmed bronhcoscopically. In all patients left sided double lumen tubes were used only. In one patient carinal decubitus was observed due to double lumen tube’s hook. Patients underwent ILV for median 4 days (range 2–10 days).

**Conclusions:** In severe single sided lung injury necessitating MV, ILV should be considered as it represents valuable adjunct in treating refractory hypoxemia. With lung separation both lungs can be ventilated according to protective ventilation strategy. Special care is needed to prevent tube dislocation, cuffs overinflation and trauma to larynx, trachea and bronchi. Since tubes’ lumens are narrower special attention to possible tube blockage must be paid. Daily reevaluation and conventional tube placement is warranted as soon as possible.

**Fig. 11 (abstract P13).**
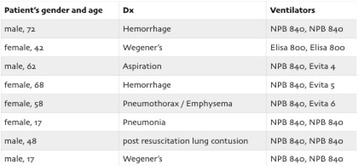
See text for description

## P14 Initiation of mechanical ventilation with the lower threshold of tidal volume in ARDS

### M. A. Suzer^1^, M. E. Kavlak^2^, H. K. Atalan^3^, B. Gucyetmez^4^, N. Cakar^4^

#### ^1^Cankaya Hospital, Ankara, Turkey; ^2^Atasehir Memorial Hospital, Istanbul, Turkey; ^3^Acibadem Fulya Hospital, Istanbul, Turkey; ^4^Acibadem University School of Medicine, Istanbul, Turkey

**Introduction:** We hypothesized that in patients with ARDS, a pH level >7.20 can be achieved by using a tidal volume (TV) of 6 ml/kg. It was demonstrated that low TV (6–8 ml/kg) strategy was related with decreased volutrauma in patients with ARDS (1–3). According to ARDSNet (ARMA) trial, the initial mechanical ventilation (MV) settings should be 8 ml/kg TV, 7–9 L/min minute volume and a respiratory rate up to 35/min to provide pH > 7.20 and it is suggested to reduce the TV to 7 ml/kg and then gradually to 6 ml/kg (4).

**Methods:** The present study was designed in 2015 as prospective observesional. Patients with ARDS who were >18 years old were included in this study. The initial MV was set with TV:6 ml/kg, MV:7–9 L/min as recommended by ARDSNet (ARMA) trial. Blood gas samples were taken at the 1st and 2nd hours of MV. In patients with pH < 7.20, TV was increased by 1 ml/kg. Demographic data, 1st and 2nd hours blood gas values and ventilation parameters were recorded.

**Results:** One hundred and ten patients were included in this study. At the 1st hour, pH values in 86.4% (95) of all patients were above 7.20. In patients with pH < 7.20, while pH-corrected-APACHE II and lactate values were significantly higher; HCO3 and SBE were significantly lower than patients with pH > =7.20 (P = 0.006 and P < 0.001 for others). In patients with pH < 7.20, pH values were increased above 7.20 by increasing TV to 7 ml/kg.

**Conclusions:** In a significant percentage of patients with ARDS, targeted pH value can be achieved by using the lower threshold of recommended initial TV value. In patients with severe hyperlactatemic acidosis, higher initial TV values may be required.

**References**

1. Petrucci N et al. Cochrane Database Syst Rev 2004; 2:CD003844

2. Petrucci N et al. Cochrane Database Syst Rev 2013; 28:CD003844

3. Needham DM et al. Am J Crit Care Med 2015; 191:177–85

4. ARDSNet Study I, ARMA trial 1998.

## P15 Lung protective ventilation: what is the effect of education on tidal volume and p0.1 values?

### D. Weller, A. F. Grootendorst, A. Dijkstra, T. M. Kuijper, B. I. Cleffken

#### Maasstad Hospital, Rotterdam, Netherlands

**Introduction:** Lung protective ventilation may improve patient outcome on ICU’s and clear guidelines are available for ventilator settings. It is unknown whether nurses and doctors adhere to these guidelines. The purpose of this study was to determine the effect of predefined lung-protective ventilation regime in pressure support ventilation (PS) introduced via an educational programme on tidal volumes and airway occlusion pressure.

**Methods:** The study was a case control single-center study. For the intervention group, an education programme was integrated consisting of eight clinical lessons of ±30 min, bed side teaching, four times written instruction by email and twice a dissemination of an educational presentation to the entire ICU team, in the period from May to June 2016. This program set guidelines for mechanical ventilation, with the specific instructions to ventilate patients with a tidal volume of 6–8 ml/kg/ideal body weight (IBW) and airway occlusion pressure (P0.1) of 2.8 to 6.0 cmH2O. Prior to ventilation instruction a control group was obtained from the general ICU population.

Inclusion criteria were: mechanical ventilation (PS) within 7 days after presentation on the ICU, exclusion criteria were: any neurological diagnosis, re-intubation and delirium.

**Results:** In total 14 patients were included in the intervention group versus 17 in the control group (baseline, Table 3).

Thirteen parameters were monitored during the study, primary endpoints where tidal volume ml/kg/IBW and the P0.1. In the intervention group (Table 3) finale tidal volumes were 1.2 ml/kg/IBW lower (p = 0.04). The P0.1 shows no difference between the two groups (p =0.75).

**Conclusions:** Conclusion: Adherence to lung-protective ventilation is increased with education and improves ventilation practice on ICU’s. An educational program on specific guidelines of mechanical ventilation decreases tidal volumes to optimal values between 6–8- ml/kg/IBW. P0.1 values could not be optimized by this regimen. It seems likely that higher pressure support levels lead to lower inspiratory work of breathing.

**Table 2 (abstract P15).** See text for description

	Controle group	Intervention group	P value
	n = 17	n = 14	
Male	10 (58.8)	10 (71.4)	
Age	70.4 ± 7.3	64.9 ± 6.9	0.04
Current weight	73.4 ± 13.5	93.4 ± 27.3	0.02
IBW	63.1 ± 9.4	65.3 ± 9.3	
Cardiac	5 (29.4)	1 (7.1)	
Surgical	8 (47,1)	2 (14.3)	
Medical	4 (23.5)	11 (78.6)	0.01
SOFA score	14.3 ± 2.3	12.3 ± 3.8	

Legend Table 2 Baseline, Mean ± SD (%)

**Table 3 (abstract P15).** See text for description

	Controle group	Intervention group	P value
	n = 17	n = 14	
SpO2 %	96.8 ± 1.3	96.4 ± 1.5	0.45
etCO2 mmHg	33.1 ± 4.0	37.8 ± 8.1	0.07
RR min.	19.4 ± 3.6	22.6 ± 4.8	0.05
AMVe L/min	9,6 ± 1,8	10,2 ± 2,2	0,44
Tve ml	541.7 ± 120.7	476.2 ± 66.8	0.07
PEEP cmH2O	9.2 ± 2.2	10.1 ± 3.1	0.38
Ppeak cmH2O	19.3 ± 3.4	23.8 ± 5.8	0.02
Pmean cmH2O	12 ± 2.1	13.8 ± 3.6	0.1
FiO2	0.4 ± 0.1	0.5 ± 0.1	0.46
P0.1 cmH2O	1.7 ± 0.9	1.8 ± 1	0.75
PS cmH2O	9.3 ± 3.5	13 ± 4	0.01
PF-ratio	217.9 ± 56.1	209.2 ± 57.7	0.68
Tve ml/kg/IBW	8.6 ± 1.7	7.4 ± 1.4	0.04

Legend Table 3 Results, Mean ± SD (%)

## P16 Intra-abdominal pressure adjusted positive end-expiriatory pressure – a pilot study

### A. Regli^1^, B. De Keulenaer^1^, P. Van Heerden^2^

#### ^1^Fiona Stanley Hospital, Perth, Australia; ^2^Hadassah University Hospital, Jerusalem, Israel

**Introduction:** Intra-abdominal hypertension (IAH) is associated with increased morbidity and mortality. IAH reduces lung volumes and oxygenation and increases airway pressures. The optimal level of positive end-expiratory pressure (PEEP) to be applied in such patients remains unknown.

Animal data suggests that high PEEP adjusted for intra-abdominal pressure (IAP) may counteract IAH-induced lung volume and arterial oxygen level reductions.

In this pilot trial, our primary aim was to assess whether high and PEEP, adjusted for IAP, can be safely applied in patients with IAH requiring mechanical ventilation. Our secondary aim was to assess the effect of such PEEP levels on oxygenation.

**Methods:** Prior to enrolment, the patients next of kin were asked for written informed consent. Patients were excluded if deemed unsuitable or if predefined severe cardiovascular dysfunction or severe hypoxemia were present.

Ventilation was standardized. Following a recruitment manoeuvre, the following PEEP levels in cmH2O were randomly applied: 5 (baseline), PEEP = 50% of IAP, and PEEP = 100% of IAP. At each measurement step, we allowed 30 min for equilibration before measuring arterial blood gases and respiratory parameters. For the safety of our patients, predefined stopping criteria were applied.

**Results:** 15 patients were enrolled. The protocol was stopped in one patient (excluded from analysis) due to an unexpected hypertensive episode (drug error unrelated to protocol). Three patients did not tolerate PEEP = 100% IAP due to hypoxemia, hypotension, or ETT cuff leak in one patient each. PaO2/FiO2 ratios (maen and SD) were 234 (68), 271 (99), and 329 (107) at each PEEP level respectively. The difference was significant (p = 0.009) only between baseline and PEEP = 100% IAP.

**Conclusions:** PEEP = 100% was commonly not tolerated and only marginally improved oxygenation in ventilated patients with IAH. Such high PEEP pressures can therefore not be recommended for routine clinical practice.

## P17 Withdrawn

## P18 Withdrawn

## P19 Neurally adjusted ventilatory assist versus pressure support in prolonged mechanical ventilation: a randomised feasibility study

### D. Hadfield^1^, P. A. Hopkins^2^, B. Penhaligon^2^, F. Reid^1^, N. Hart^3^, G. F. Rafferty^1^

#### ^1^King’s College London, London, UK; ^2^King’s College Hospital, London, UK; ^3^Guy’s and St Thomas’ Hospital, London, UK

**Introduction:** Neurally adjusted ventilatory assist (NAVA) is a complex intervention involving diaphragmatic monitoring and a proportional, neurally triggered ventilation mode [1]. Clinical effectiveness has not been demonstrated and feasibility data are required prior to larger definitive randomised controlled trials (RCTs). Although there is no agreement on minimum levels in clinical trials, low protocol compliance will impact on statistical power and interpretation of results in a definitive study [2]. The aim of this trial is to assess feasibility by measuring protocol compliance in the setting of a pragmatic RCT.

**Methods:** A feasibility RCT in 76 ventilated adult ICU patients, currently being conducted in a central London hospital. Patients at high risk of prolonged ventilation are randomly assigned to NAVA or Pressure Support (PSV) in the weaning phase. Feasibility end-points include protocol compliance (time in the NAVA mode), recruitment rate and acceptability. Secondary outcomes include ventilator free days and sedation load. The data from the first 48 patients (n = 25 and 23 in the NAVA and PSV groups respectively) are presented.

**Results:** At 14/11/2016 480 patients had been screened and 55 recruited with a mean recruitment rate of 1.8 patients per month. 71 patients were approached for inclusion with 16 patients (22.5%) excluded due to personal or professional consultee refusal. 68.2% of patients in the intervention group were compliant [CI 47.3–83.6] with a median adherence to the NAVA mode of 87.5% [38.9, 99]. Ventilator free days to day 28 were 18 [8, 24] days in the NAVA group vs. 19 [0, 23] days in the PSV group. There were no adverse events related to the intervention.

**Conclusions:** These data suggest an acceptable protocol compliance of > 65%. Further analysis of the reasons for non-compliance will be conducted; initial data suggest that a mix of technological, clinical and human factors underlie reduced adherence and protocol compliance. This is the first trial to compare NAVA to PSV in prolonged ventilation in the context of a pragmatic RCT. The results will provide guidance for the design of future trials.

**References**

1. Sinderby, C., et al., Neural control of mechanical ventilation in respiratory failure. Nature medicine, 1999. 5(12): p. 1433–6.

2. Chan, A.-W., et al., SPIRIT 2013 Statement: Defining Standard Protocol Items for Clinical Trials. Annals of Internal Medicine, 2013. 158(3): p. 200–207.

**Table 4 (abstract P19).** See text for description

Variable	NAVA	Control
Age, years	68 (53, 74.5)	74(66, 79)
Sex, number male	18 (72%)	15 (70%)
APACHE II	20 (15.5, 25.5)	21 (13, 22)
Chronic Obstructive Pulmonary Disease	9 (36%)	8 (34.8%)
Heart Failure	17 (60.7%)	16 (69.6%)
Acute Respiratory Distress Syndrome	4 (14.3%)	3 (13%)
Protocol compliance, % (95% CI)^a^	68.2% [47.3–83.6]	NA
Adherence with allocated ventilation mode	82.3% (19.7, 97.6) n = 22	100% (100, 100) n = 21
Ventilator free days to D28, days	18 [8, 24]	19 [0, 23]
ICU stay from randomisation (survivors), days	9.1 (6, 21.9) n = 19	12 (7.1, 41.8) n = 16
Hospital stay from randomisation (survivors), days	20.7 (12.3, 35.6) n = 18	31.5 (14.8, 57.6) n = 13
ICU mortality	5 (17.9%)	7 (30.4%)

Legend Table 4 ^a^Protocol compliance defined as >65% adherence to the NAVA mode during weaning

## P20 Effects of prone position and recruitment manoeuvres on gas exchange and regional respiratory mechanics

### G. Grasselli^1^, T. Mauri^2^, M. Lazzeri^3^, E. Carlesso^2^, B. Cambiaghi^4^, N. Eronia^4^, E. Maffezzini^4^, A. Bronco^4^, C. Abbruzzese^1^, N. Rossi^1^, G. Foti^4^, G. Bellani^4^, A. Pesenti^2^

#### ^1^Fondazione IRCCS Ca’ Granda Ospedale Maggiore Policlinico, Milan, Italy; ^2^University of Milan, Milan, Italy; ^3^University of Ferrara, Ferrara, Italy; ^4^University of Milan-Bicocca, Monza, Italy

**Introduction:** Prone position (PP) improves survival and delays the risk of ventilation-induced lung injury in severe ARDS [1]. PP may exploit its benefits in the presence of optimized alveolar recruitment. Thus, we performed prone positioning in combination with recruitment manoeuvres (RMs) to assess the effects on aeration and oxygenation.

**Methods:** Eleven intubated, volume-controlled, ARDS patients (1 severe, 8 moderate, 2 mild) were studied in supine and PP before and after RMs (40 cmH2O airway pressure for 10 breaths). In each phase electrical impedance tomography (EIT) was recorded for 15 minutes to assess global and regional (dependent and non-dependent) respiratory system compliance (Crs), tidal volume (VT) distribution, end-expiratory lung volume changes (ΔEELV). Gas exchange and respiratory mechanics were monitored at the end of each phase.

**Results:** PaO[sub]2[/sub]/FiO[sub]2[/sub] was 159 ± 71 mmHg. Clinical PEEP (13 ± 3 cmH[sub]2[/sub]O), VT (6.6 ± 1.2 ml/kg IBW), RR (25 ± 5 bpm) and FiO[sub]2[/sub] (0.6 ± 0.2) were left unchanged. PaO[sub]2[/sub]/FiO[sub]2[/sub] improved after RMs (p = 0.054) and by interaction between PP and RMs (p = 0.046). ΔEELV significantly increased after RM performed in supine (p = 0.032; 40 ± 53 ml vs. pre-RM), in PP (p = 0.002; 95 ± 78 ml vs. pre-RM) but by similar magnitude (p = 0.089) post-RM supine vs. prone. PP didn’t modify Crs, but dependent Crs significantly improved (p = 0.006) and non-dependent Crs (p = 0.009) decreased. VT distribution was more homogenous during PP and lung homogeneity index improved, albeit non-significantly. Neither regional Crs nor homogeneity were affected by RMs.

**Conclusions:** PP improves regional mechanics and homogeneity; RM performed after proning seems efficacious and might increase PP protective effects.

**References**

1. Valenza F. et al. Crit Care Med. 2005;33:361–7

**Table 5 (abstract P20).** See text for description

	Supine pre-RM	Supine post-RM	Prone pre-RM	Prone post-RM	p-value position	p-value RM	p-vaule interaction
PaO_2_/FiO_2_ [mmHg]	144 ± 63	147 ± 65	172 ± 77	191 ± 92	0.109	0.054	0.046
Homogeneity index	1.79 ± 0.80	1.71 ± 0.77	1.33 ± 0.55	1.28 ± 0.54	0.272	0.183	0.281
Crs [ml/cmH_2_O]	41 [23–46]	37 [22–47]	37 [24–50]	36 [24–48]	0.810	0.811	0.911
Crs non-dep [ml/cmH_2_O]	23 [14–32]	23 [15–33]	13 [9–28]	12 [10–29]	0.009	0.426	0.494
Crs dep [ml/cmH_2_O]	12 [8–19]	9 [8–19]	17 [13–23]	16 [13–20]	0.006	0.106	0.845

Legend Table 5 Main results of our study

## P21 Feasibility of the lateral-trendelenburg vs semi recumbent body position – the GRAVITY-VAP TRIAL

### G. Li Bassi^1^, M. Panigada^2^, O. Ranzani^1^, T. Kolobow^3^, A. Zanella ^2^, M. Cressoni^4^, L. Berra^5^, V. Parrini^6^, H. Kandil^7^, G. Salati^8^, S. Livigni^9^, S. Livigni^10^, A. Amatu^10^, M. Girardis^11^, M. Barbagallo^12^, G. Moise^13^, G. Mercurio^14^, A. Costa ^12^, A. Vezzani^12^, S. Lindau^15^, J. Babel^16^, M. Cavana^17^, A. Torres^1^

#### ^1^Hospital Clinic, Barcelona, Spain; ^2^Policlinico di Milano, Milan, Italy; ^3^National Institutes of Health, Bethesda, USA; ^4^Ospedale San Paolo, Milan, Italy; ^5^Massachusetts General Hospital, Boston, USA; ^6^Ospedale Nuovo del Mugello, Borgo San Lorenzo, Italy; ^7^Gruppo Ospedaliero San Donato, San Donato Milanese, Italy; ^8^Arcispedale S. Maria Nuova, Reggio Emilia, Italy; ^9^Ospedale San Giovanni Bosco, Torino, Italy; ^10^Policlinico San Matteo, Pavia, Italy; ^11^Policlinico di Modena, Modena, Italy; ^12^Azienda Ospedaliero-Universitaria di Parma, Parma, Italy; ^13^Ospedale Citta di Sesto San Giovanni, Sesto San Giovanni, Italy; ^14^Policlinico Gemelli, Roma, Italy; ^15^University Hospital Frankfurt, Frankfurt, Germany; ^16^University Hospital Zagreb, Zagreb, Croatia; ^17^Ospedale Santa Chiara, Trento, Italy

**Introduction:** We recently completed a large randomized clinical trial in critically ill patients to test the LTP vs. SRP in the prevention of ventilator-associated pneumonia (VAP). Here we focus on the feasibility of the study interventions.

**Methods:** We randomized 194 and 201 critically ill patients into LTP and SRP group, respectively. Patients in LTP were placed in semi-lateral (60°) - Trendelenburg position and turned from one side to the other every 6 hours. LTP was encouraged specifically during the first days of mechanical ventilation, in compliance with the patient’s wish. In the SRP group, patients were kept with the head of the bed elevated > = 30°. We assessed interventions feasibility, and nursing feasibility/workload

**Results:** Patients in the LTP and SRP group were kept in the randomized position for 38% and 90% of the study time, respectively (p = 0.001). Median fraction of time in LTP reached up to 51.8% (interquartile range 20.7–79.2) during the first 2 days; yet, it progressively decreased in subsequent days and was inversely related to the level of sedation. Median lorazepam equivalents was 46.7 mg/day (interquartile range, 20.8–120.0) and 58.0 (28.0–124.2) in the SRP and LTP groups, respectively (p = 0.37); propofol dose was 1393 mg/day (627–2345) and 1661 (826–2700) (p = 0.13). Finally, morphine equivalents were 112 mg/day (30–217) in the SRP and 119 (31–234) in the LTP (p = 0.56). Nurses reported greater difficulties in positioning the patient in LTP and higher workload (p < 0.001) vs SRP. However, approximately 50% of the LTP patients were easily or very easily positioned. Finally, these nursing challenges slightly ameliorated through practice, as more patients were enrolled in each center.

**Conclusions:** The LTP was specifically applied during the first days of MV. Of note, higher level of sedation/analgesia was not needed to keep the patient in LTP. At times, the nurses encountered difficulties in executing the LTP.

## P22 Lateral-trendelenburg vs. semi recumbent body position for the prevention of ventilator-associated pneumonia – the GRAVITY-VAP TRIAL

### M. Panigada^1^, G. Li Bassi^2^, O. T. Ranzani^2^, T. Kolobow^3^, A. Zanella^1^, M. Cressoni^4^, L. Berra^5^, V. Parrini^6^, H. Kandil^7^, G. Salati^8^, S. Livigni^9^, A. Amatu^10^, M. Girardis^11^, M. Barbagallo^12^, G. Moise^13^, G. Mercurio^14^, A. Costa^12^, A. Vezzani^12^, S. Lindau^15^, J. Babel^16^, M. Cavana^17^, A. Torres^2^

#### ^1^Policlinico di Milano, Milan, Italy; ^2^Hospital Clinic, Barcelona, Spain; ^3^National Institutes of Health, Bethesda, USA; ^4^Ospedale San Paolo, Milan, Italy; ^5^Massachussets General Hospital, Boston, USA; ^6^Ospedale Nuovo del Mugello, Borgo San Lorenzo, Italy; ^7^Gruppo Ospedaliero San Donato, San Donato Milanese, Italy; ^8^Arcispedale S. Maria Nuova, Reggio Emilia, Italy; ^9^Ospedale San Giovanni Bosco, Torino, Italy; ^10^Policlinico San Matteo, Pavia, Italy; ^11^Policlinico di Modena, Modena, Italy; ^12^Azienda Ospedaliero-Universitaria di Parma, Parma, Italy; ^13^Ospedale di Sesto San Giovanni, Sesto San Giovanni, Italy; ^14^Policlinico Gemelli, Rome, Italy; ^15^University Hospital Frankfurt, Frankfurt, Germany; ^16^University Hospital Center Zagreb, Zagreb, Croatia; ^17^Ospedale Santa Chiara, Trento, Italy

**Introduction:** The semi-recumbent position (SRP) could increase risk of gravity-driven pulmonary aspiration and ventilator-associated pneumonia (VAP) (1–2). We investigated whether the lateral-Trendelenburg position (LTP) vs. the SRP would prevent microbiologically confirmed VAP.

**Methods:** 194 patients were randomized into the LTP group and 201 in SRP, and analyzed in an intention to treat approach. Patients in LTP were turned from one side to the other every 6 hours. Whereas, in the SRP group, patients were kept with the head of the bed elevated > = 30°. Primary outcome was VAP incidence rate, based on quantitative bronchoalveolar lavage fluid culture. Secondary outcomes were duration of mechanical ventilation, intensive care unit (ICU) and hospital stays, and ICU/hospital/28-day mortality.

**Results:** The data safety monitoring board recommended stopping the study at the second interim analysis for low incidence of VAP in the control group, lack of benefits in any major secondary outcome and adverse events in the LTP group. Microbiologically confirmed VAP was 0.5% (1/194 patients) in patients positioned in LTP, and 4.0% (8/201 patients) in patients in SRP, risk ratio (RR) between groups 0.13, 95% confidence interval (CI) 0.02–1.03, p = 0.04. Microbiologically confirmed VAP per 1000 ventilator days was 7.19, 95%CI 3.60–14.37 and 0.88, 95%CI 0.12–6.25 in the LTP and SRP, respectively, RR 0.12, 95%CI 0.01–0.91, p = 0.02. Competing risk analysis, which accounted for the concomitant risk of death and discontinuation of MV on VAP, confirmed lower cumulative probability of VAP in the LTP (cause-specific hazard ratio 0.13, 95% CI 0.02–1.00, p = 0.05). No statistically significant between-group differences were observed in secondary outcomes.

**Conclusions:** Critically ill patients positioned in the LTP had a statistically significant reduction in the incidence of VAP, in comparison with the SRP. Yet, further clinical examinations are mandatory to corroborate our findings, specifically in populations at high risk of VAP.

**References**

1) Li Bassi G et al. Crit Care Med 2014; 42: e620–7

2) Panigada M et al. Crit Care Med 2003; 31: 729–37

## P23 Effects of lateral position and open chest on partitioned respiratory mechanics during thoracic surgery

### M. Umbrello, M. Taverna, P. Formenti, G. Mistraletti, F. Vetrone, A. Marino, G. Vergani, A. Baisi, D. Chiumello

#### Ospedale San Paolo, Università degli Studi di Milano, Milano, Italy

**Introduction:** Airway pressure (Paw) based respiratory mechanics is used to guide ventilation. As an altered chest wall may influence the interpretation of Paw, aim of the study was to investigate the behavior of lung and chest wall during different phases of thoracic surgery

**Methods:** 20 patients undergoing pulmonary resection were enrolled. Double-lung ventilation (DLV) setting: PEEP 8 cmH2O, tidal volume (TV) 8 ml/kg; in one-lung ventilation (OLV) TV was reduced to 5 ml/kg and respiratory rate increased accordingly. Esophageal pressure (Pes) was measured and used to assess partitioned respiratory mechanics (ie. elastance of the respiratory system - Ers, chest wall - Ecw and lung - El). Respiratory mechanics was assessed during DLV in supine and lateral decubitus, OLV in lateral decubitus during closed and open chest

**Fig. 12 (abstract P23).**
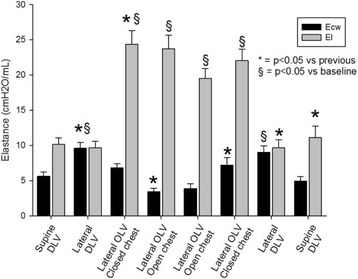
See text for description

**Results:** Supine Ers (cmH2O/L) in DLV was 15.8 ± 4.6; no change in lateral position and DLV (19.3 ± 4.4, p > 0.05); significant increase in lateral position with OLV, with no difference between closed and open chest (31.2 ± 9.6 vs. 27.2 ± 8.9, p > 0.05). Figure 12 shows changes of El and Ecw: Ecw increased in lateral position and decreased when chest was opened. El was unchanged in lateral position but significantly increased during OLV.

**Conclusions:** Paw-based monitoring of respiratory mechanics did not allow to differentiate the cause of an alteration. Partitioned respiratory mechanics gives a deeper insight into the behavior of lung and chest wall.

## P24 Manual versus closed loop control of oxygenation parameters during invasive ventilation: effects on hyperoxemia

### A. G. Garnero^1^, D. N. Novotni^2^, J. A. Arnal^1^

#### ^1^Hôpital Sainte Musse, Toulon, France; ^2^Hamilton medical, Bonaduz, Switzerland

**Introduction:** Hyperoxemia occurs up to 50% of mechanical ventilation days in the ICU [1] and is associated with increased mortality as compared to patients ventilated in normoxemia [2]. INTELLiVENT-ASV is a full closed loop ventilation mode adjusting automatically oxygenation parameters according to SpO2 for passive and spontaneously breathing mechanically ventilated patients. This post-hoc analysis of a monocentric randomized controlled parallel group study compared hyperoxemia (PaO2 > 120 mm Hg or SpO2 > 96%) and hypoxemia (PaO2 < 60 mm Hg or SpO2 < 90%) between INTELLiVENT-ASV and conventional ventilation mode in mechanically ventilated ICU patients.

**Methods:** This randomized controlled trial was performed in the general ICU of Hôpital Sainte Musse, Toulon, France. Eligible participants: adult invasively ventilated for less than 24 h at the time of inclusion with an expected duration of mechanical ventilation of more than 48 h. Exclusion criteria: broncho-pleural fistula, ventilation drive disorder, moribund patients. Patients were allocated to INTELLiVENT-ASV group or to conventional ventilation group (volume assist control and pressure support) using blocked randomization. The post-hoc analysis was performed by the comparison of all arterial blood gases (ABG) performed during the study period: the number of ABG with hyperoxemia and hypoxemia, the median PaO2 and SpO2 for these ABG and FiO2 associated were compared.

**Results:** 60 patients were included, 30 patients in each group.

**Conclusions:** The closed loop control of oxygenation settings provided by INTELLiVENT-ASV decreases significantly the number of blood gas with hyperoxemia as compared to manual oxygenation setting without increasing the risk of hypoxemia.

**References**

[1] Suzuki et al. J Crit Care 28(5):647–654, 2013.

[2] Girardis et al. JAMA 316(15) :1583–1589, 2016.

**Fig. 13 (abstract P24).**
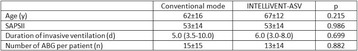
Legend 1: Demographic results

**Fig. 14 (abstract P24).**
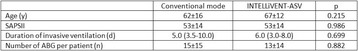
Legend 2: Blood gas results

## P25 Withdrawn

## P26 Predicting the presence of spontaneous breathing in mechanically ventilated patients

### M. Urner, E. Fan, M. Dres, S. Vorona, L. Brochard, N. D. Ferguson, E. C. Goligher

#### University of Toronto, Toronto, Canada

**Introduction:** Databases from large trials are more frequently used to test new hypotheses about the impact of mechanical ventilation. In absence of direct measurement of respiratory effort, it is challenging to know if patients are breathing spontaneously. We sought to establish if the presence of spontaneous breathing can be predicted from readily available clinical variables.

**Methods:** Logistic regression, extreme gradient boosting, and random forest models were built from clinical variables to predict a diaphragm thickening fraction > 0.2 (mean value in healthy subjects breathing at rest [1]) as reference standard for inspiratory effort. Data from 195 patients was randomly split into a training (75%) and a test dataset (25%). The models were derived from the training dataset by cross-validation and validated on the test dataset. All models were implemented in R using the caret package.

**Fig. 15 (abstract P26).**
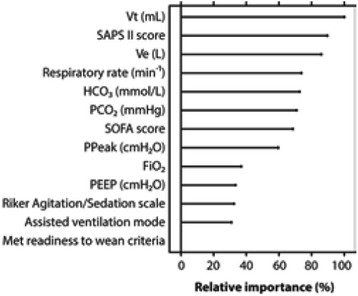
Legend 1: Relative variable importance according to random forest analysis

**Results:** The AUROC for the logistic regression, extreme gradient boosting, and random forest models was 0.65 (0.59 to 0.70), 0.69 (0.63 to 0.72) and 0.68 (0.61 to 0.78). Diaphragm thickening fraction was most closely related to tidal volume, SAPS II score, and minute ventilation (Fig. 15).

**Conclusions:** We present an approach to detect spontaneous breathing during mechanical ventilation. Our models may be useful to identify spontaneously breathing patient subsets in cohort studies, but predictive accuracy is insufficient to replace direct monitoring of individual patients.

**References**

[1] Harper CJ et al., J Orthop Sports Phys Ther. 2013; 43(12):927–31

## P27 Mechanical ventilation training using flipped-classroom with a one-hour face-to-face tutorial

### C. Leung, G. Joynt, W. Wong, A. Lee, C. Gomersall

#### Chinese University of Hong Kong, Sha Tin NT, Hong Kong

**Introduction:** We studied the effectiveness of a flipped classroom approach using e-learning and a one-hour face-to-face (FTF) practical training in teaching medical students to manage basic mechanical ventilation (MV). Teaching MV is challenging due to teacher time limitations.

**Methods:** We performed a prospective cohort evaluation of the level of MV competence of final year medical students who participated in this optional training. The courseware consisted of an e-chapter (to introduce basic MV settings and physiology); online interactive cases and practice tests with a MV simulator (to facilitate scenario-based practice); a one-hour FTF tutorial (1 teacher to 6 students).

Two critical care educators, informed only of the syllabus outline, created the knowledge and skills assessments at a level expected of interns. Knowledge was assessed by pre- and post-course multiple choice questions (MCQs) testing 10 domains of MV. For each student, two MCQs of equal difficulty per domain were randomized to pre- and post-course tests. The two educators conducted 10-minute post-course skills testing of students’ practical competence in setting the correct MV mode, FiO2, tidal volume, respiratory rate (RR), PEEP in a case scenario; performing appropriate action in response to desaturation, pressure or RR alarms; reassessing after setting or adjusting MV. Students’ feedback on the course’s usefulness was surveyed.

**Results:** 179(81%) students consented to participate. Mean MCQ score pre-course was 29% and post-course was 53% - with a mean increment (95% CI) of 32.6(28.7–36.6) in students who completed the course including FTF, 7.0(0.8–13.1) when courseware was used without FTF, and 7.7(−1.6–17.1) in those who did not attempt the course (p < 0.001). MCQ score improved in every domain (McNemar’s test, P-values varied from <0.001 to <0.03) and for all domains combined (McNemar’s test P <0.001). 23 randomly selected students participated in the skills test - median score was 8/10(IQR 6.75–8.5). Students reported the course useful for improving their MV competence. Median score of individual course components was 4–5(IQR 4–5); 4 = agree, 5 = strongly agree.

**Conclusions:** The course is a time-efficient and effective approach to improve students’ MV knowledge and skills. Although FTF appeared to be necessary, we believe that it must be preceded by e-learning as one-hour FTF is insufficient to provide the background and practice skills teaching. Students showed motivation to participate and found the training useful. The courseware is freely available to other educators and therefore only requires clinical teachers to provide one-hour FTF sessions.

## P28 Predictors of prolonged mechanical ventilation after lung transplants: a retrospective cohort study

### S. Poels, M. Casaer, M. Schetz, G. Van den Berghe, G. Meyfroidt

#### UZ Leuven, Leuven, Belgium

**Introduction:** The objective of our study was to identify predictors for prolonged mechanical ventilation (PMV) in patients after lung transplantation (ssLTx).

**Methods:** We performed a retrospective analysis of 36 patients admitted to our ICU after ssLTx, between 2011 and 2014. Baseline characteristics are summarized in Table 6. Cox proportional Hazard model was used to assess the risk of PMV.

**Results:** Of the preoperative risk factors (listed in Table 6), the indication pulmonary hypertension (PHT) was a statistical significant predictor, compared with COPD (P 0.04) and other pathology (P 0,0079) but not compared to pulmonary fibrosis (P 0,0927) when corrected for age, APACHE II >20, BMI < 20, dexmedetomidine or conventional sedation.

After transplantation, the only significant predictor of PMV (P 0,0238) was extracorporeal membrane oxygenation (ECMO), corrected for age, APACHE II > 20, BMI <20, Dexdor or conventional sedation and reperfusion edema.

Limitations of this study are the retrospective and single-center design, and the low sample size.

**Conclusions:** PMV after ssLTx is mainly explained by the indication for lung transplantation (PHT) and the postoperative need for ECMO.

**Table 6 (abstract P28).** See text for description

Preoperative risk factors	
Age med (IOR)	57.5 (43.25/61.75)
BMI mean (SD)	22.96 (+ − 3.71)
APACHE II med (IQR)	19.5 (18/24.75)
COPD N(%)	17 (47)
Pulmonary hypertension N(%)	2 (5.5)
Other (MUCO, Kartagener, Hemangioblastoma)	4 (11)
Lung fibrosis N(%)	13 (36)
Preoperative use of corticosteroids N(%)	15 (41.6)
Preoperative use of oxygen N(%)	16 (44)
Postoperative risk factors	
ECMO N(%)	7 (19.4)
Sedation with Dexmedetomidine	24 (66)
Tracheostomy N(%)	2 (5.5)
Reperfusion edema N(%)	14 (38)

Legend Table 6 Baseline characteristics

**Fig. 16 (abstract P28).**
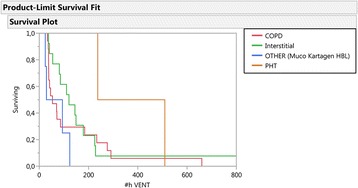
Legend 1: Kaplan Meier Cluster diagnosis

## P29 The effect of hypoxaemia on cognitive outcome in adult patients with severe acute respiratory failure treated with conventional mechanical ventilation or extracorporeal membrane oxygenation: a systematic review of the literature

### B. Holzgraefe^1^, L. B. Von Kobyletzki^2^, A. Larsson^3^

#### ^1^Karolinska Institutet, Stockholm, Sweden; ^2^Lund University, Karlstad University, Lund, Sweden; ^3^Uppsala University, Uppsala, Sweden

**Introduction:** Treatment with extracorporeal membrane oxygenation (ECMO) and/or hypoxaemia in patients with acute respiratory failure (ARF) has been suggested to cause short and long-term cognitive impairment. To explore the evidence for this, we performed a systematic review.

**Methods:** We searched the databases Medline, PsycInfo, Cochrane Library, and Embase to identify publications, i.e. randomised controlled trials, nested case–control studies and cohort studies, reporting the possible association between hypoxaemia and cognitive impairment in patients treated for ARF with or without ECMO.

**Results:** We identified 2606 citations. After eliminating duplicates, two reviewers screened 1825 publications for eligibility and read 30 full text papers. No study fulfilled the inclusion criteria. We identified six studies, mainly case series, which dealt with the study question. One case series reported a correlation between hypoxaemia (haemoglobin oxygen saturation < 90%) and cognitive dysfunction at discharge but not at 1 and 2-year follow-up. In another study which concerned cognitive impairment after ECMO treatment for all causes, no direct correlation was found between ECMO and cognitive impairment. Cognitive impairment was more common after veno-arterial ECMO, which is rarely used in ARF. Mikkelsen et al. reported that a lower PaO2 was significantly associated with cognitive impairment at 12 months follow-up.

**Conclusions:** The evidence is sparse that that ECMO treatment for ARF or hypoxaemia during the course of ARF leads to long-term cognitive impairment. Therefore, any estimation of the actual risk of cognitive impairment as a result of using ECMO or employing hypoxaemia, permissively or inadvertently, in ARF cannot be made. More, high quality studies are needed to explore these clinically important questions.

**References**

Mikkelsen ME et al. American journal of respiratory and critical care medicine 2012; 185(12): 1307–15.

Risnes I et al. The Annals of thoracic surgery 2006; 81(4): 1401–6.

Hopkins RO et al. American journal of respiratory and critical care medicine 1999; 160(1): 50–6.

## P30 Patterns of morpho-functional pulmonary recovery after total structural consolidation

### G. Cianchi^1^, F. Becherucci^1^, S. Batacchi^1^, M. Cozzolino^1^, F. Franchi^2^, S. Di Valvasone^1^, M. C. Ferraro^1^, A. Peris^1^

#### ^1^Careggi Teaching Hospital, Florence, Italy; ^2^University of Siena, Siena, Italy

**Introduction:** Extracorporeal membrane oxygenation (ECMO) can be used in patients with ARDS and life-threatening hypoxia, also to reduce VILI. We described 4 patients requiring ECMO treatment with massive lung consolidation and impaired lung ventilation.

**Methods:** Patient 1, a 63 years old woman, was admitted for ARDS in S. pneumoniae pneumonia with massive bilateral consolidations requiring ECMO treatment; for 4 days she was minimally ventilated (2 mL/kg); after ECMO weaning, protective ventilation was applied for other 5 days. After 16 days she was discharged; CT scan showed residual minimal consolidation of the right lung. She had a good recovery after 6 months. Patient 2, a 50 years old woman, was admitted from another ICU for ARDS in bilateral P. aeuriginosa pneumonia. After 7 days of ECMO and 5 more days of protective ventilation, minimal bilateral consolidation with cystic areas were seen at CT scans; at discharge, after 18 days, she had a good recovery of respiratory function. Patient 3, a 49 years old woman, was admitted for A(H1N1) influenza virus pneumonia evolved in ARDS. Massive lung bleeding and complete pulmonary consolidation occurred; ECMO and minimal ventilation lasted 66 days, mechanical ventilation continued for 10 days after ECMO weaning. She was discharged after 81 days spontaneously breathing, with a reduction of bilateral consolidation at CT. She had a good recovery after 6 months. Patient 4, a 54 years old woman, arrived from another ICU for severe ARDS due to viral pneumonia and massive bilateral consolidations. ECMO treatment was started and for 30 days she was not ventilated for massive bilateral consolidations; weaning from ECMO was not possible for bacterial infection and evolution in pulmonary fibrosis. The patient died in ICU for multi organ failure after 300 days.

**Results:** Massive pulmonary consolidation is not a terminal event in ECMO patients. Pulmonary consolidation can be related to an infection or may depend on massive intra-alveolar bleeding. In our series of 4 ECMO patients, pulmonary consolidation were caused in one case by bleeding, in the others by infectious diseases.

**Conclusions:** We experienced that massive pulmonary consolidation can resolve, provided that patients is kept alive and VILI is minimized by ECMO support. In our series, outcome and long term sequelae seems mainly related to the underling disease, rather than to the extension of pulmonary consolidation. Periods of limited or absent ventilation do not seem to prevent recovery of alveolar function. Bleeding does not seem to exclude a full lung recovery, while infections may cause abscesses and pulmonary disruption with possible long term sequelae and residual lung dysfunction. The authors confirm that written consent for publication had been obtained.

## P31 Initial ECMO experience in a Thai tertiary hospital

### H. Phiphitthanaban, P. Wacharasint, V. Wongsrichanalai, A. Lertamornpong, O. Pengpinij, A. Wattanathum, N. Oer-areemitr

#### Phramonkutklao hospital, Bangkok, Thailand

**Introduction:** Extracorporeal membrane oxygenation (ECMO) may be a lifesaving procedure in patients with severe respiratory and/or circulatory failure (1,2). We present the first 19 adult patients treated with ECMO at medical intensive care unit (MICU) of Phramongkutklao Hospital, a Thai tertiary referral hospital.

**Methods:** A descriptive observational study was performed in 19 patients who received ECMO as a rescue therapy. Initial patient’s characteristics, technical aspects, and ECMO-related complications were described.

**Results:** Our adult ECMO program was started in August 2014. Since then we supported seventeen respiratory failure patients on veno-venous (VV) ECMO, one electrical shock patient on veno-arterial (VA) ECMO, and one cardiac arrest patient on VA ECMO-assisted extracorporeal cardiopulmonary resuscitation (E-CPR). Ultrasound-guided femero-jugular cannulation was percutaneously done, by intensivist, in all VV ECMO patients, while femoro-femoral cannulation was done by cardiothoracic surgeon using open technique in both cases of VA ECMO. The most frequent indication was severe pneumonia with acute respiratory distress syndrome (n = 10). Mean duration on mechanical ventilation before ECMO was 6 days. Before ECMO was initiated, mean Murray score was 3.4, respiratory compliance was 18 mL/cmH2O, PaO2/FiO2 was 102.5, and blood lactate was 5.7 mmol/L. Mean duration of ECMO was 10 days, average length of MICU and hospital stay were 18 and 21 days, respectively. Eight out of nineteen (42%) were decannulated after improvements, while remaining eleven cases who developed multiorgan failure resulting in death while on ECMO. ECMO-related complications were found in eleven cases (58%), which the most complication was active bleeding at the cannulation site.

**Conclusions:** Team organization and meticulous care during ECMO are crucial for minimizing ECMO-related complication. Even number of patients treated with ECMO in Thailand is increasing, a higher ECMO case volume may be required to increase the experience and improve quality of care.

**References**

1. Peek GJ, Mugford M, Tiruvoipati R, Wilson A, Allen E, Thalanany MM, et al. Efficacy and economic assessment of conventional ventilatory support versus extracorporeal membrane oxygenation for severe adult respiratory failure (CESAR): a multicentre randomized controlled trial. Lancet 2009;374:1351–63.

2. Noah MA, Peek GJ, Finney SJ, Griffiths MJ, Harrison DA, Grieve R, et al. Referral to an extracorporeal membrane oxygenation center and mortality among patients with severe 2009 influenza A (H1N1). JAMA 2011;306:1659–68.

## P32 Cannula-related deep vein thrombosis during extracorporeal membrane oxygenation (ECMO) treatment do not affect mortality and length of stay in intensive care unit

### M. Boddi, G. Cianchi, E. Cappellini, M. Ciapetti, S. Batacchi, G. Di Lascio, M. Bonizzoli, M. Cozzolino, A. Peris

#### Careggi Teaching Hospital, Florence, Italy

**Introduction:** Deep vein thrombosis (DVT) continues to be a serious complication in (intensive care unit) ICU patients despite the extensive use of pharmacological and mechanical prophylaxis. During extracorporeal membrane oxygenation (ECMO) treatment, the risk of cannula-related DVT is markedly increased, because the large calipter of cannulas causes endothelium damage and significantly decreases venous flow around the cannula. In this perspective it is possible to assume that during ECMO treatment cannula-related DVT could represent an additional risk of mortality and morbidity for ICU patients. We studied the incidence of cannula-related and not related DVTs in ECMO patients and their potential impact on the mortality and length of stay in this high risk subgroup of ICU patients.

**Methods:** This is a retrospective study on 116 patients, 53 + 19 years old, with ARDS unresponsive to conventional treatments, who fulfilled the criteria for ECMO positioning set by the the Italian Ministry of Health. ECMO configuration included 78 (67.2%) patients with bicaval dual lumen mono-cannulation (J-ECMO), 21 (18.1%) with jugular-femoral cannulation (JF-ECMO) and 17 cases (14.6%) with femoro-femoral cannulation (FF-ECMO). Ultrasound exams for DVT diagnosis at the jugular-subclavian-axillary and the femoral-popliteal and sottopopliteal venous axes were performed within the first 48 hours after ECMO placement, once a week during ECMO treatment and within 48 hours after cannulas removal. During ECMO treatment the heparin infusion was adjusted to maintain aPTT target between 50 and 80 seconds.

**Results:** We diagnosed 45 cannula-related DVT: 39 DVTs were located at the jugular vein and 8 at femoral veins. We found 36 (56.25%) not cannula-related DVTs; 9 cases were central venous catheter (CVC)-related DVTs. No DVT was symptomatic and all were diagnosed by scheduled ultrasound sourvellaince; asymptomatic pulmonary embolism was diagnosed in 5 cases, 3 of which in not cannula-related DVT. In our study, the overall incidence of cannula-related DVT was independent of vascular configuration of ECMO (J; FF; JF). The diagnosis of cannula related DVT was not associated with a higher ICU mortality or longer ICU length of stay; on the contrary Not cannula-related DVT significantly increased ICU mortality and ICU length of stay (p < 0.005).

**Conclusions:** In our ECMO population only DVTs occurring in veins not involved in cannulation were significantly associated with increased mortality and ICU length of stay. These data strongly suggest that different pathophysiologic mechanisms are involved in the occurrence of cannula-related and not related DVT.

## P33 Survival of septic patients with refractory ARDS requiring veno-venous ECMO

### C. Lazzeri, G. Cianchi, M. Bonizzoli, G. Di Lascio, M. Cozzolino, A. Peris

#### Careggi Teaching Hospital, Florence, Italy

**Introduction:** The role of extracorporeal membrane oxygenation (ECMO) remains controversial in adult patients with septic shock and data are so far scare and not univocal. We investigated the outcome of 26 patients with refractory ARDS and septic shock, consecutively admitted to our Intensive Care Unit (which is an ECMO referral center) from January 2013 to December 2015 and treated with venous-venous ECMO (VV-ECMO).

**Methods:** According to our center protocol, all patients were submitted to an echocardiography (transthoracic and/or transesofageal) before VV-ECMO implantation. Mortality during ICU stay was the primary outcome.

**Results:** During the study period, 74 patients with refractory ARDS were consecutively treated with VV-ECMO at our center, among whom 16 patients had concomitant septic shock (35.1%). When compared to patients without septic shock, septic patients showed higher values of SOFA (sepsis: 13.1 ± 3 vs no sepsis: 10.2 ± 2, p < 0.001), lactate values (sepsis: 6.12 ± 5.90 vs no sepsis: 3.09 ± 5.65, p = 0.07) and procalcitonin (sepsis: 76.85 ± 95.37 vs no sepsis: 12.05 ± 30.15, p < 0.01). At echocardiography, no differences was observed between the two subgroups in the incidence of left ventricular dysfunction (chi-square: p = 0.657), right ventricle dilation (chi-square: p = 0.347) and right ventricle dysfunction (chi-square: p = 0.688). The overall mortality rate was 44% (33/74) but it did not differ between patients with and without septic shock (chi-square: p = 0.218).

**Conclusions:** In patients with refractory ARDS requiring VV ECMO, septic shock is quite common. The use of VV-ECMO in these patients is associated with a comparable survival rate when compared to nonseptic patients. According to our data, VV ECMO is not contraindicated in patients with refractory ARDS and septic shock.

## P34 Rescue ecmo therapy in h1n1 induced ARDS – successful experience from Belarus

### M. L. Katsin, M. Y. Hurava, A. M. Dzyadzko

#### Republican Scientific and Practical Center for Organ and Tissue Transplantation, Minsk, Belarus

**Introduction:** The H1N1 strain of Influenza A is associated with a number of complications including acute respiratory distress syndrome (ARDS). Invasive mechanical ventilation (IMV) does not always provide an optimal solution. Extracorporeal membrane oxygenation (ECMO) has been shown to be a rescue treatment of severe H1N1-induced ARDS.

**Methods:** Here we describe first series of successful application of ECMO in these settings in Belarus.

**Results:** From January to April 2016 three patients with positive serodiagnostics of H1N1 requiring IMV for severe ARDS were admitted in different urban hospitals. Despite ARDS-Net recommended approach to IMV, it was not possible to achieve PaO2 > 60 mmHg, SpO2 > 54%. Ad-hoc veno-veinous ECMO with femoral and internal jugular cannulation was initiated shortly. IMV parameters were adjusted as follow: tidal volume reduced to 3–4 ml/kg PBW; PEEP 12–16 cmH2O, FiO2 40–50%. Patients were further transferred to the ICU of our tertiary care center. No adverse effects of transportation were noted. Median time of transfer was less than 60 min. Ultraprotective IMV was applied using APRW mode with Pinsp 20–22 cmH2O, PEEP 12–16 cmH2O, and FiO2 35–45%. Target tidal volume was 3–4 ml/kg PBW, and inspiration/expiration ratio was 1.5:1–2.5:1. Two patients required myoplegia. After 1 to 4 days of IMV an APRW mode was changed to IntelliVent-ASV™. ECMO settings were as following: flow 3–4 l/min (60–80% of minute volume); fresh gas flow 1.5–2.5 (max 9.7) l/min depending on PaCO2; and oxygen fraction 60–100% to achieve SpO2 92–94%.

The durations of ECMO were 9, 11 and 42 days. All patients received transcutaneous tracheostomy on 3–5 day of IMV, ventilator-associated pneumonia prevention bundle. We followed standard ECMO weaning procedure protocol after stabilization of respiratory function and achieving control of SIRS. Spontaneous breathing test was successful in two patients on day two after ECMO weaning. One patient with the longest duration of ECMO developed nosocomial septic shock with lung abscess complicated by hemopneumothorax, hemorrhagic shock and further left lung inferior lobectomy. He required additional 10 days of IMV following ECMO weaning, with successful return to natural airway breathing at day 67 after hospitalization. All patients survived hospitalization.

**Conclusions:** We successfully used ECMO as a rescue therapy in three patients with severe H1N1 induced ARDS that allowed weaning of IMV and recovery.

## P35 Long-term ecmo without anticoagulation in patients with severe thrombocytopenia

### A. Hermann, P. Schellongowski, A. Bojic, K. Riss, O. Robak, W. Lamm, W. Sperr, T. Staudinger

#### Medical University of Vienna, Vienna, Austria

**Introduction:** Severe thrombocytopenia yields a high risk for bleeding thus representing a relative contraindication for anticoagulation and therefore ECMO. We herein report on a series of haematological patients with severe thrombocytopenia undergoing long-term ECMO without anticoagulation.

**Methods:** Retrospective analysis of patient charts undergoing veno-venous gas exchange between 2012 and 2016. Data were extracted from charts and the local ECMO registry. Six patients fulfilling the criteria of severe thrombocytopenia < 50 G/L without anticoagulation for more than 3 days were identified.

**Results:** Patients suffered from acute myelogenous leukemia (n = 3), multiple myeloma (1) and acute lymphoblastic leukemia and consecutive stem cell transplantation (n = 2), respectively. ECMO was performed due to ARDS (n = 4) and graft versus host disease involving the lungs (n = 2). All ECMO systems used were heparin coated. Platelet count was 21 G/L (median, range 1–138), ECMO duration was 31 days (6–262), and ECMO was run without any anticoagulation for 17 days (7–262). Altogether, three clotting events were seen leading to oxygenator changes. Bleeding was common, leading to one fatal intracerebral bleeding, a minor subarachnoidal bleeding, a temporary gastric bleeding and persistent haematuria. Altogether, 27 packed red blood cells per patient (median, range 4–280) or 0,78 per day (0.57–1.25) and 22 platelet concentrates per patient (range 7–207) or 0.8 per day (0.57–1.26) were administered. Three of six patients could be weaned from ECMO, one patient survived ICU.

**Conclusions:** In patients with severe thrombocytopenia, ECMO can be run without anticoagulation even for longer periods. Nevertheless, bleeding is common while clotting events seem to be rare. Given the high mortality rate in this population, however, the indication for ECMO should be scrutinized rigorously.

## P36 Long-term quality of life after extracorporeal membrane oxygenation in ards patients: an Italian tertiary centre experience

### L. Tadini Buoninsegni, M. Bonizzoli, M. Cozzolino, J. Parodo, A. Ottaviano, L. Cecci, E. Corsi, V. Ricca, A. Peris

#### Careggi Teaching Hospital, Florence, Italy

**Introduction:** Patients who develop acute respiratory distress syndrome (ARDS) have high mortality and morbidity rates and survivors could have clinically significant physical and psychological disabilities. Several studies have reported that extracorporeal membran oxygenation(ECMO) may improve survival in severe ARDS but there have been only few studies evaluating long-term outcomes in ECMO-treated ARDS survivors (1). The purpose of the study was to assess intensive care unit (ICU) outcomes and long term outcome and quality of life of patients with ARDS receiving ECMO for refractory hypoxemia.

**Methods:** We conducted a retrospective observational study in adult ARDS patients who had veno-venous-ECMO in a tertiary centre from January 2014 until April 2016. We collected demographic factors, Simplified acute physiology score (SAPS II) at admission, diagnosis, mechanical ventilation and ECMO duration, ICU outcome. We contacted telephonically all ICU-survivors and organized, when possible, a follow-up visits on average 6 months after discharge from ICU. The primary outcome variable was ICU survival and health related quality of life (HRQoL) measured with the Short-Form 36 (SF-36). Long-term quality of life assessment were assessed and compared to normative individual age- matched Italian population.

**Results:** Sixty-four patients (mean age 50.4 ± 14.7 years, 62% males) were studied; 44 patients (69%) were retrieved from external intensive care units (ICUs) by a dedicated ECMO retrieval team. SAPS II at admission was 42.0 ± 15.1 (mean ± DS). Infectious disease were the leading causes of ARDS: 46% bacterial and 21% A(H1N1) influenza pneumonias. Mean duration of vv-ECMO support was 18.8 ± 27.5 days (median of 11 days). 38 patients (59%) survived to ICU discharge; 15 were discharged to other hospitals. Of the 38 ICU-survivors, 25 patients (65.7%) had a follow-up visit after 6 months from ICU discharge. 5 patient died within 6 months and 8 patients were lost/unable to come to visit. HRQoL was evaluated for 21 patients: compared with age-matched controls, our ARDS survivors had significantly lower (p < 0.005) SF-36 physical function and physical role functioning; vitality, bodily pain, general health perceptions, emotional role functioning, social role functioning and mental health were comparable with those of general population.

**Conclusions:** In this ARDS cohort treated with ECMO the ICU-survival and six months after ICU discharge survival rate was similar to other findings (1). Long term survivors had reduced physical health but their psychological domain scores were comparable with those of the general population.

**References**

1. Schmidt M et al.: Intensive Care Med 2013;39:1704–13

## P37 Advance Organ Support (ADVOS) based on albumin dialysis, a new method for CO2 removal and pH stabilization

### A. Perez Ruiz de Garibay, B. Ende-Schneider, C. Schreiber, B. Kreymann

#### Hepa Wash GmbH, Munich, Germany

**Introduction:** Our group has recently developed an Advance Organ Support (ADVOS) system based on albumin dialysis to provide intensive care treatment for patients suffering multiple organ failure including liver, kidney and lung impairments. The system has already shown improved survival in two different animal models as well as safety and efficacy to eliminate water and protein-bound toxins in humans with liver failure [1, 2]. In the present work, the ability of the ADVOS procedure to eliminate CO2 and stabilize blood pH together with the reduction of bilirubin and urea levels has been determined. Results were compared to a conventional renal dialysis machine (NIKKISO DBB-03).

**Methods:** For this purpose an ex vivo model for respiratory acidosis was developed continuously infusing 110 ml/min CO2 into 5 liters swine blood. In addition, liver and kidney detoxification were simulated supplementing blood with bilirubin (275 mg/dl) and urea (30 mg/dl), respectively. Blood was subjected to hemodialysis in the ADVOS system for 4 hours through two dialyzers (2 × 1.9 m^2^) using a blood flow (BF) of 400 ml/min and a dialysate pH of 10. The NIKKISO machine was run through a dialyzer (2.5 m^2^) with a BF of 350 ml/min and a dialysate pH of 8. CO2, pH, bilirubin and urea levels were analyzed pre- and post-dialyzer. Blood was checked for hemolysis at the beginning and the end of the experiments.

**Results:** During the whole hemodialysis time using the ADVOS procedure, an average CO2 removal of 108 ± 4 ml/min was achieved. The ADVOS system was able to maintain pH stable between 7.35 and 7.45 during the experiments, while with the NIKKISO machine pH decreased to 6.60 after one hour of treatment, being thereafter continuously out of the measuring range (hence no further calculations were possible) In the ADVOS system the main fraction was excreted as HCO3- (85%), while 15% was eliminated as dissolved CO2. In addition, post-dialyzer blood pH remained in both systems below 8. Urea was efficiently cleared with both machines (97% removal). Moreover, the ADVOS system reduced bilirubin levels about 3 times as much as conventional hemodialysis (59% vs. 21%). No signs for hemolysis were observed.

**Conclusions:** The ADVOS system, in contrast to normal hemodialysis, was able to efficiently remove CO2, bilirubin and urea while maintaining pH in physiological levels in an ex vivo model for respiratory acidosis simulating additional kidney and liver failure.

**References**

1. Al-Chalabi A et al. BMC Gastroenterol 13: 83, 2013.

2. Henschel B et al. Crit Care 19 (Suppl 1):P383, 2015.

## P38 ECCO2 removal with a membrane oxygenator (Prismalung ®) integrated in a CRRT plataform (prismaflex ®): a feasibility study

### F. Turani ^1^, M. Resta^2^, D. Niro^2^, P. Castaldi^3^, G. Boscolo^4^, G. Gonsales^5^, S. Martini^1^, A. Belli^1^, L. Zamidei^5^, M. Falco^1^

#### ^1^Aurelia and European Hospital, Rome, Italy; ^2^Istituti clinici S Donato MI, Milan, Italy; ^3^Marino Hospital, Cagliari, Italy; ^4^Ospedale dell’ Angelo, Mestre, Italy; ^5^Santo Stefano Hospital, Prato, Italy

**Introduction:** Extracorporeal CO2 removal is used in COPD/moderate ARDS patients to avoid barotrauma and VILI. When renal failure coexists, a combined ECCO2 removal with RRT may be useful to improve CO2 clearance and kidney function in a single treatment.

The aim of this study is to evaluate 1- the clinical feasibility of a CO2 removal device integrated in a CRRT platform.2 - The clinical safety of the system. 3 - The changes of main respiratory and metabolic indices

**Methods:** Ten patients have been enrolled into the study. All patients had renal failure, were hypercapnic and mechanically ventilated, but one.

The patients were connected to a low -flow CO2 removal device (Prismalung ®) integrated into a conventional RRT platform (Prismaflex) by a two single lumen catheters inserted in jugular and femoral vein or by a bilumen catheter in a femoral vein. Pump blood flow was started at 300 ml/min and progressively increased to 400 ml/min. RRT was delivered in a CVHDF mode with an effluent flow of 45 ml/kg/hour. Heparin or citrate infusion (three cases) was used to anti coagulate the circuit. ACT and thromboelastography were used to monitoring the coagulation parameters.

At basal time (T 0), 12 th (T 1), 24 th (T2) hours, the main respiratory parameters and the renal indices were evaluated. Bleeding complications, hemofilter clottings, catheter malfunctions were recorded. All data are expressed as Mean ± SD… ANOVA TEST one way with Bonferroni correction was used to compare the changes of the parameters in the time. P < 0.05 was considered statistically significant.

**Results:** At Table 7 are reported the main results of this study. All patients, but one, survived to the treatment and 4/10 were weaned from the ventilator at the end of ECCO2R. The duration of the treatments was 60 ± 30 hours. There was a clotting of the circuit and two cases of catheter malfunction. No major bleeding episodes were observed.

**Conclusions:** This study confirms previous study of Prismalung on animals.(1) ECCO2 removal with a membrane oxygenator integrated in a CRRT platform is clinically feasible and devoid of major complications. It allows clearance of CO2, with improvement of respiratory acidosis and control of renal failure.

**References**

1) Anaesth Crit Care Pain Med (2015) http://dx.doi.org/10.1016/j.accpm.2014.08.006

**Table 7 (abstract P38).** See text for description

	T0	T1	T2
PH	7.23 ± 0.07	7.34 ± 0.1	7.37 ± 0.1*
PaCO2 mmHg	86 ± 26	56 ± 16**	51 ± 15**
CO2 removal ml/min	53 ± 14	57 ± 15	55 ± 17
Creatinine mg/dL	2.13 ± 2	1.96 ± 0.8	1.41 ± 0.7
Blood flow ml/min	250 ± 28	330 ± 34	330 ± 41

Legend Table 7 *p < 0.05 vs basal time **p < 0.01 vs basal time

## P39 Ultra-low blood flow veno-venous extracorporeal CO2 removal (ECCO2R) with acidification and regional anticoagulation together with hemodiafiltration

### T. Lamas^1^, J. Mendes^2^

#### ^1^Egas Moniz Hosp., Lisboa, Portugal; ^2^Fernando da Fonseca Hosp., Lisboa, Portugal

**Introduction:** The aim of this study was to assess safety and performance of the new ECCO2R device, i.e. Prismaflex® + Prismalung® (Baxter), with citric acid as regional anticoagulant by calcium chelating effect and acidification to displace the dissolved CO2 from HCO3- and removing it through a membrane lung (Prismalung®, PL) with a blood flow (Qb) of 250 ml/min.

**Methods:** This study was conducted on 6 male pigs (68 ± 4.5 kg) separated in two groups, 2 pigs in the control group (Group A) and 4 pigs in the intervention group (Group B). Both groups were submitted to Phase I consisting of 4 hours with citrate anticoagulation (3.3 mmol/L) no CO2 removal (circuit bypass). In Phase II, for 8 hours both groups were submitted to ECCO2R with a FiO2 100% sweep gas flow of 15 L/min; Group A was submitted to the same citrate anticoagulation system as in Phase I while Group B was switched to citric acid anticoagulation (2.7 mmol/L) and acidification (2.0 mEq/min). All pigs were sedated and paralysed and connected to a ventilator (Servoi®, Maquet). The ventilator parameters were fixed mean tidal volume (7.11 ± 0.48 ml/kg) with PEEP 5cmH2O, FiO2 40%, V/min 8.1 ± 1.5 L/min (respiratory rate 18–20).

**Fig. 17 (abstract P39).**
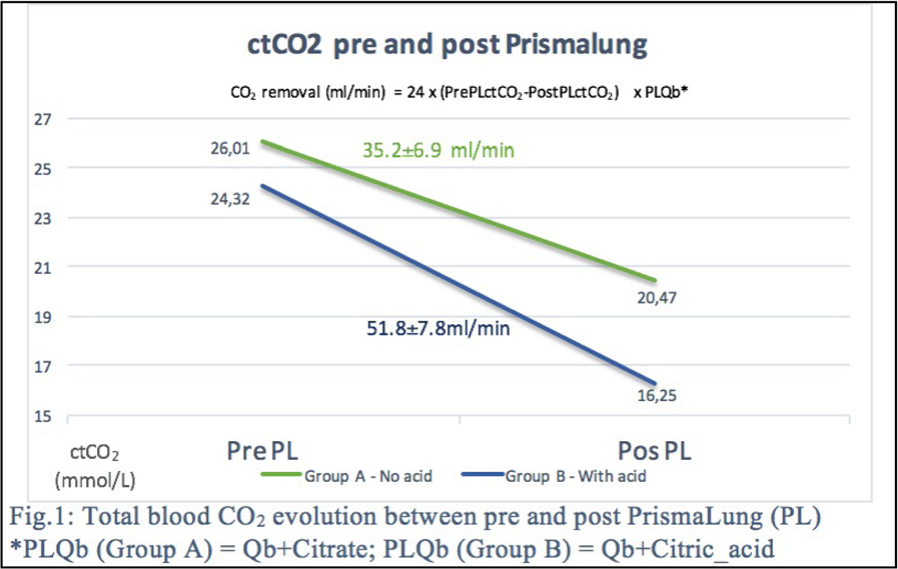
Total blood CO_2_ evolution between pre and post PrismaLung (PL). *PLQb (Group A) = Qb + Citrate; PLQb (Group B) Qb + Citric_acid

**Results:** CO2 content pre and post PL and CO2 removal is depicted in Fig. 17. The systemic pH and pCO2 did not differ statistically between the two groups. The systemic ionized calcium was stable in both groups. During the study no major critical events were recorded (death, cardiac arrest, arrhythmias, etc.). PL CO2 removal in intervention group (51.8 ± 7.8 ml/min) was significantly higher than in control group (35.2 ± 6.9 ml/min).

**Conclusions:** Using citric acid as a regional anticoagulant and acidification during ECCO2R is safe and more effective to remove the CO2 comparing standard ECCO2R.

## P40 Effect of flow and temperature on comfort during high flow nasal cannula

### A. Galazzi^1^, T. Mauri^1^, B. Benco^1^, F. Binda^1^, L. Masciopinto^1^, M. Lazzeri^2^, E. Carlesso^1^, A. Lissoni^1^, G. Grasselli^1^, I. Adamini^1^, A. Pesenti^1^

#### ^1^Fondazione IRCCS Ca’ Granda Ospedale Maggiore Policlinico, University of Milan, Milano, Italy; ^2^Sant’Anna Hospital, University of Ferrara, Ferrara, Italy

**Introduction:** Over the last few years, high flow nasal cannula (HFNC) is increasingly adopted as first line treatment of acute hypoxemic respiratory failure (AHRF) patients [1]. One of the key features of HFNC efficacy seems to be elevated patients comfort, which might critically depend from set temperature and flow. Aim of the study is to assess the comfort of patients undergoing HFNC at different temperatures and flows.

**Methods:** We conducted a prospective, randomized, cross-over study on 18 AHRF patients, admitted to the intensive care unit (ICU) and receiving respiratory support by HFNC as per clinical indication. We randomly applied 2 flows (HFNC 60 l/min and 30 l/min) and 2 temperatures (31 °C and 37 °C) for 15 minutes (four steps per patient). Clinical FiO2 was left unchanged during all steps. We investigated during the last minutes of each step: comfort by numerical scale from 0 (extreme discomfort) to 5 (very comfortable); dyspnea by modified Borg scale form 0 (none) to 10 (unbearable); physiologic respiratory parameters. Data of each step were compared using linear mixed-effects model analysis (parametric or non-parametric, as appropriate) for repeated measures with Bonferroni or Tukey post-hoc test.

**Results:** We enrolled 18 patients, aged 51 ± 14 year-old, 6 females. At enrollment, SpO2 was 97 ± 2% with FiO2 43 ± 13%, the etiology of AHRF was various. Main study results are reported in Table 8. Patient comfort level was significantly higher during HFNC at lower flow and temperature (p = 0.015 and p < 0.001 respectively), but interaction was not significant. Peripheral saturation was significantly increased by higher flow rate (p = 0.026). Interestingly, in this convenience sample, higher HFNC support didn’t modify the respiratory rate likely indicating clinical stability.

**Conclusions:** Lower flow and temperature grant improved patient comfort. Titration of HFNC setting to obtain best comfort might be relevant to exploit clinical efficacy.

**References**

1. Roca O et al. Crit Care 28;20(1):109, 2016

**Table 8 (abstract P40).** See text for description

	HFNC 30 l/min-31 °C	HFNC 30 l/min–37 °C	HFNC 60 l/min–31 °C	HFNC 60 l/min–37 °C	P-value for flow	P-value for temp	P-value for interaction
Comfort scale	5 [4–5]	3 [1–3]	3 [2–4]	2 [0–3]	0.015	<0.001	0.145
Modified Borg scale	3 [2–5]	4 [2–5]	5 [2–6]	5 [2–5]	0.461	0.718	0.283
SpO2 %	97 [94–99]	97 [95–99]	97 [96–99]	98 [96–99]	0.026	0.598	0.190
RR (bpm)	21 [20–25]	23 [20–27]	22 [20–24]	22 [21–25]	0.815	0.248	0.332
FC (bpm)	87 [73–97]	89 [77–98]	86 [74–97]	87 [73–101]	0.858	0.081	0.511
PAS (mmHg)	117 [104–131]	117 [106–132]	115 [105–134]	116 [109–135]	0.805	0.126	0.979

Legend Table 8 Comfort and physiology at different set flows

## P41 High flow nasal cannula prevent reintubation in post-extubated critically ill patients: a systematic review and meta-analysis

### T. Thamjamrassri, J. Watcharotayangul, P. Numthavaj, S. Kongsareepong

#### Mahidol university, Bangkok, Thailand

**Introduction:** Extubation failure and reintubation after planned extubation are associated with high mortality [1]. Multiple factors including impair secretion drainage, hypoxemia or hypercarbia are possible causes of extubation failure [2]. Therefore, giving appropriate oxygen therapy and respiratory care after extubation in critically ill patients should decrease incidence of reintubation. Recently, high flow nasal cannula (HFNC) has been used increasingly in critically ill patients because it has many advantages over conventional oxygen therapy (COT). HFNC provides higher flow rate and more humidity which facilitated secretion drainage. Moreover, PEEP causing from HFNC decreases work of breathing [3]. Previous study shown that HFNC decreased incidence of reintubation in post-extubated critically ill patients [4]. Although there is a meta-analysis compared HFNC with other methods of oxygen therapy, patients who failed extubation criteria were included in the study [5]. Therefore, we conduct a meta-analysis comparing HFNC with other methods of oxygen therapy in preventing reintubation in planned extubated critically ill patients.

**Methods:** MEDLINE, OVID and the Cochrane Library databases were searched. RCTs and cohorts studies comparing HFNC with other methods of oxygen therapy in post-extubated critically ill patients were included. The primary outcome was re-intubation rate. The secondary outcomes were ICU mortality, length of stays (LOS) in ICU and post-extubated respiratory failure.

**Results:** 6 studies were analyzed in the meta-analysis. HFNC significantly reduced re-intubation rate (RR = 0.4, 95% CI 0.26–0.61) and post-extubated respiratory failure (RR = 0.51, 95% CI 0.35–0.73) comparing to COT. However, there were no significant difference in ICU mortality (RR = 0.93, 95% CI 0.47–1.83) and LOS (PMD = −0.14, 95% CI −0.65–0.36)

**Conclusions:** HFNC reduced re-intubation rate and post-extubated respiratory failure compared to COT. There were no difference in ICU mortality and LOS.

**References**

1. Thille AW, et al. Am J Respir Crit Care Med 187:1294–302, 2013

2. Kulkarni AP, et al. Indian J Crit Care Med 12: 1–9, 2008

3. Spoletini G, et al. Chest 148:253–61, 2015

4. Hernández G, et al. JAMA 315:1354–61, 2016

5. Monro-Somerville T, et al. Crit Care Med. 2016 Sep 8. [Epub ahead of print]

## P42 The role of high flow nasal cannula oxygen therapy in the intensive care unit

### J. Higuera, D. Cabestrero, L. Rey, G. Narváez, A. Blandino, M. Aroca, S. Saéz, R. De Pablo

#### Ramón y Cajal University Hospital, Madrid, Spain

**Introduction:** The goal of the study is to analyze the impact and the results of the application of high flow nasal cannula in an intensive care unit of a tertiary, university hospital.

**Methods:** We perform a retrospective study with all patients who have been treated with high flow nasal cannula (HFNC) for their respiratory failure between May 2013 and April 2016.

**Results:** 174 patients were included. Average age: 57.81 ± 15.5 (18–88), male (58.6%). Severity index: SOFA 8.17 ± 4.29 (1–19), APACHE II 19.43 ± 8.3 (3–44) and SAPS II 48.66, average stay 14 days and mortality rate of 22.4%

For a better categorization of patients, diagnostics were tabulated in the following groups. Respiratory 52.3% Haematological 20.7%, Septic 16.1%, Neurological 4.6%, Cardiologic 3.4%, Digestive 0.6%, Oncologic 0.6%

The origin of respiratory failure was primarily: Respiratory (72.4%), Septic (21.3%), and miscellaneous (6.3%)

Patients required high flow nasal cannula therapy for hypoxemic decompensation in 77.6% of the cases. In the 1.7% of the cases the cause were mixed and was hypercapnia in the 20.1% of the cases.

56.9% (99/174) of the patients, required mechanical ventilation. 43.1% of the patients (75/174) didn’t require mechanical ventilation. In 31 up to the 99 patients who require mechanical ventilation, HFNC was used as a support for the scheduled extubation. In 68 up to 99 patients, mechanical ventilation was required after HFNC failure.

The group of patients which needed mechanical ventilation had higher mortality (p < 0.0001).

The mortality rate is considerable higher due to retardation of the intubation, after the high flow oxygen therapy has failed. The patients intubated the first 48 hours after the high flow oxygen therapy failure have a mortality of 44% vs the 61% after 72 hours.

There are significant differences in the mortality rate regarding the type of patient. Is very probable that the most benefit group by HFNC is the one with severe respiratory failure with exclusive respiratory cause and hypoxic decompensation.

**Conclusions:** The use of high flow oxygen therapy has changed the attitude towards the patients with insufficiency respiratory failure and may avoid in some cases the use of mechanical ventilation. The delay in the orotracheal intubation after high flow therapy has failed, increases the rate of mortality. Further controlled studies are needed to display which other patients can be beneficed by the high flow oxygen therapy.

## P43 High flow nasal cannula in immunocompromised patients with acute respiratory failure: a systematic review and meta-analysis

### A. Mohamed, M. Sklar, L. Munshi

#### Sinai Health System, Toronto, Canada

**Introduction:** Our study objectives were to evaluate the impact of high flow nasal cannula (HFNC) in immunocompromised (IC) patients with acute hypoxemia respiratory failure. The role of HFNC compared to conventional oxygen therapy remains controversial in this population.

**Methods:** We performed a systematic review and meta-analysis to evaluate the application of HFNC in an adult IC patients with acute respiratory failure (to August 2016). We included any randomized controlled trials (RCTs) or observational studies and meta-analyzed results where HFNC was compared to face mask oxygen or non-invasive ventilation (NIV). Our primary outcome was the need for mechanical ventilation. A series of secondary outcomes were analyzed.

**Fig. 18 (abstract P43).**
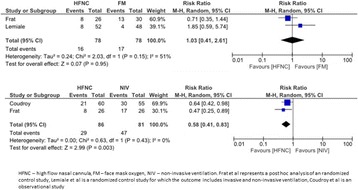
Primary Outcome (Mechanical Ventilation)

**Results:** Of 3099 citations screened, 6 fulfilled our inclusion criteria (3 RCTs, 3 observational studies). Across 428 patients, the definition of IC included patients with pharmacologic IC states or IC due to one’s underlying disease. Of the two trials evaluating the application of HFNC vs face mask, there was no decrease in the rates of mechanical ventilation (RR 1.03, 95% confidence interval (CI) 0.41–2.61, p = 0.95) Fig. 18. Contrary to this, HFNC vs NIV was associated with a decrease in need for invasive mechanical (RR 0.58, 95% CI 0.41–0.83, p = 0.003).

**Conclusions:** Evidence to date is limited on the application of HFNC in this population; therefore, a larger number of high quality studies are needed to corroborate these preliminary findings.

## P44 Setting high flow nasal cannula to maximize physiologic benefits

### T. Mauri^1^, M. Lazzeri^2^, L. Alban^2^, C. Turrini^2^, M. Panigada^3^, P. Taccone^3^, E. Carlesso^1^, C. Marenghi^3^, S. Spadaro^2^, G. Grasselli^3^, C. Volta ^2^, A. Pesenti^1^

#### ^1^University of Milan, Milan, Italy; ^2^University of Ferrara, Ferrara, Italy; ^3^Fondazione IRCCS Ca’ Granda Ospedale Maggiore Policlinico, Milan, Italy

**Introduction:** High-flow nasal cannula (HFNC) is a non-invasive respiratory support associated to several physiologic advantages in acute hypoxemic respiratory failure (AHRF) patients [1]. Previous studies showed that these effects depend on set flow. Aim of the study was to identify the flow at which gas exchange, end-expiratory lung volume (ΔEELV), respiratory rate (RR), inspiratory effort and corrected minute ventilation (MVcorr = MV*PaCO[sub]2[/sub]/40) result in highest improvement.

**Methods:** We performed a prospective, randomized, cross-over study, divided in 4 phases, on AHRF patients (PaO[sub]2[/sub]/FiO[sub]2[/sub] < =300) admitted to intensive care unit. We randomly applied standard facial mask (12 l/min) vs HFNC set at 30, 45 and 60 l/min, each step lasting 20 minutes. Clinical FiO[sub]2[/sub] was left unchanged. We collected gas exchange, lung volumes by electrical impedance tomography, patient’s inspiratory effort by esophageal pressure (Pes) and respiratory rate.

**Fig. 19 (abstract P44).**
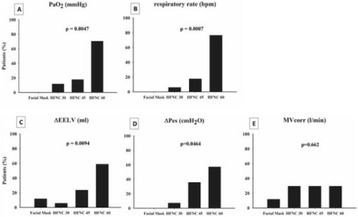
Legend 1: Flow distribution optimizing variables. p chi-squared test among flows

**Results:** We enrolled 17 patients (9 females), aged 62 ± 10 year-old. At enrolment the PaO[sub]2[/sub]/FiO[sub]2[/sub] was 151 ± 60 mmHg. The highest PaO2 value in 71% of patients was reached at HFNC 60 l/min (p = 0.0047). The highest lung volume was obtained for 59% of patients at HFNC 60 l/min (p = 0.0094). The lowest respiratory rate (bpm) in 77% of patients was at HFNC 60 l/min (p = 0.0007) and the lowest value in ΔPes was obtained for 57% of patients at HFNC 60 l/min (p = 0.0464). On the contrary, the percentage of patients with minimum value of MVcorr was equally distributed among the 3 HFNC flows (p = 0.662). Figure 19 shows flows distribution.

**Conclusions:** Our results show that HFNC 60 l/min might be regarded as the optimal flow for most parameters. However, 30–40% of patients obtained best results at lower flows and with absence of “optimum” flow for corrected minute ventilation.

**References**

1. Roca O. et al. Crit Care 28;20:109, 2016

## P45 The role of high flow nasal cannula oxygen therapy in hematological patients with severe respiratory failure

### J. Higuera, D. Cabestrero Alonso, A. Blandino, G. Narváez, L. Rey González, M. Aroca, S. Saéz, R. De Pablo

#### Ramón y Cajal University Hospital, Madrid, Spain

**Introduction:** Our goal is to analyze the impact and the results of the application of high flow oxygen therapy (HFNC) in hematological patients with respiratory failure

**Methods:** We performed a retrospective study.

All hematological patients who were treated with HFNC between May 2013 and April 2016 were included (n = 36)

**Results:** Average age: 51. 67 ± 15. 77 (23–80); 50% male; mean SOFA: 9. 09 ± 5. 15 (2–19), and APACHE II: 22. 61 ± 8. 86 (3–40). The etiology of respiratory failure was hypoxemic in 91. 66% (n = 33) and mixed in 8. 33% (n = 3) of cases.

**Fig. 20 (abstract P45).**
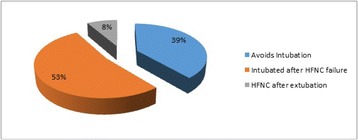
Legend 1: Graphic 1

HFNC avoided intubation and therefore mechanical ventilation (MV) in 38. 9% (n = 14) of the patients. 19 patients required MV after HFNC failure and in the other 3 HFNC was used after extubation. (Fig. 20)

**Fig. 21 (abstract P45).**
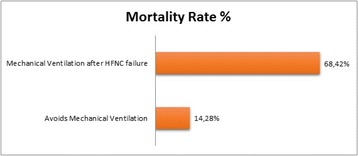
Legend 2: Graphic 2

The mortality rate in the study was 44.4% (n = 16). In the group of patients who avoided MV was 14.28% (2/14). In the ones who required mechanical ventilation after HFNC failure the mortality rate was 68.42% (13/19). 3 Patients required HFNC after extubation, 1/3 died. (Fig. 21)

The group of patients, who need mechanical ventilation, had higher mortality. (p <0.0001) However, the mortality rate was higher in this group due to delay of intubation. The average delay in the patients who died (n = 13) was 2.28 days vs. 1.83 days in the survivors group (n = 6)

**Conclusions:** HFNC oxygen therapy appears to be a safety therapeutic option for the treatment of severe respiratory failure in patients with haematological malignancies. Intubation was avoided in a substantial proportion of patients. Also was related a lower mortality rate in this subgroup of patients. However, the delayed of intubation due to HFNC failure was related to higher mortality.

## P46 Withdrawn

## P47 The advanced airway management in the out-of-hospital setting: a comparison between endotracheal intubation and extra-glottic devices in terms of mortality and outcome

### A. Franci, G. Stocchi, G. Cappuccini, F. Socci, M. Cozzolino, C. Guetti, P. Rastrelli, A. Peris

#### Careggi Teaching Hospital, Florence, Italy

**Introduction:** The study aims to compare endotracheal tube (ETT) and extraglottic devices (EGD) in the out-of-hospital setting, in terms of consequences and outcome. Secondary endpoints were evaluation of parameters that could correlate with survival and neurological outcome and evaluation of the influence of a mechanical ventilation (MV) vs bag valve mask ventilation (BVM) during the transport from the field to the hospital.

**Methods:** This retrospective study examined 404 patients with a out-of-hospital airway management between January 2013 and June 2016. 187 of them were transferred to the Intensive Care Unit (154 managed with ETT and 33 with EGD); they were analyzed for demographic, infective, radiologic and neurologic parameters. Patients with ETT were subsequently divided in 2 subgroups, the ones who received a MV during the transport to the hospital and the ones who were manually ventilated.

**Results:** Our results show that there was no correlation between the use of ETT or EGD and mortality rates 48 hours after the admittance to ICU, but there was a significant difference in survival after 7 days. There was no significant difference between ETT and EGD in terms of neurological and pulmonary outcome when patients were dismissed from ICU. The ventilation technique during the transport revealed to be an accurate parameter of survival after 48 hours, as the MV reduced patients’ mortality when compared to the BVM.

**Conclusions:** The study confirmed the importance of advanced airway management in the out-of-hospital setting, regardless of the device used. Evidence shows that ETT is recommended whenever possible, and most of all the use of MV because this seems to reduce mortality rate, probably reducing pulmonary damage deriving from the impossible titration of volumes and pressures administered to the lung during BVM.

**Table 9 (abstract P47).** See text for description

	Patients with ETT	Patients with EGD	p
BAL POSITIVITY at admittance in ICU (%)	57/154 (37%)	14/33 (42%)	NS
PaO2/FiO2 48H	289 (±99.2)	272 (±127.04)	NS
Lenght Of Stay (days)	8.2 (±7.91)	8.3 (±9.55)	NS
Mortality after 7 Days	37/154 (24.02%)	14/33 (42.42%)	0.047
Survival in MV vs BVM	59/60 (98.33%) vs 82/94 (87.23%)	-	0.033

## P48 Arterial oxygenation and hemodynamics during extrathoracic negative pressure in a porcine model of lung injury

### A. Nestorowicz^1^, J. Glapinski^2^, A. Fijalkowska-Nestorowicz^3^, J. Wosko^4^

#### ^1^Medical Univesity, Lublin, Poland; ^2^Wroclaw University of Technology, Wroclaw, Poland; ^3^Medical University, Lublin, Poland; ^4^SPSK No4, Lublin, Poland

**Introduction:** Recruitment maneuvers have been advocated recently, however their routine use is still under debate [1]. This study attempts to evaluate the impact of extrathoracic negative pressure (eNP) on arterial oxygenation and hemodynamics in pigs with acute lung injury.

**Methods:** The study involved 10 adult Large White pigs weighting 48–60 kg. Under general anaesthesia animals were intubated and ventilated in a volume-controlled mode (Puritan-Bennett 840) with F[sub]I[/sub]O[sub]2[/sub]-1.0, I:E ratio-1:2. Vt −8–10 ml/kg and RR-14–18/min were used to maintain PaCO[sub]2[/sub]- 35–45 mmHg. An infusion of lactate Ringers solution was given iv at a rate of 5–8 ml/kg/h.

Lung injury was induced with bronchoalveolar lavage by warm 0.9% NaCl (30 ml/kg) until PaO[sub]2[/sub]/F[sub]I[/sub]O[sub]2[/sub] remain below 100. Thereafter, using a whole body close-chamber, the eNP was commenced in combination with IPPV.

Heart rate (HR), mean arterial pressure (MAP), mean pulmonary arterial (MPAP) and pulmonary capillary wedge (PCWP) pressures, cardiac output (CO) (Swan-Ganz catheter) as well as arterial oxygen tension (PaO[sub]2[/sub]) were recorded before and after lung injury: at 0 (baseline), −4, −8, −12, −16 cmH[sub]2[/sub]O.

**Fig. 22 (abstract P48).**
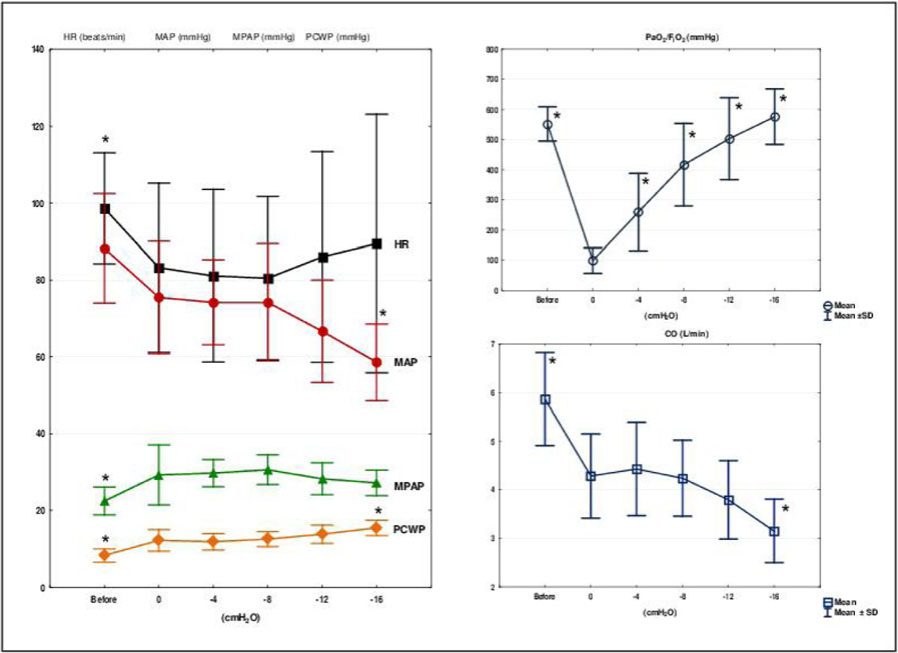
Legend 1: *P < 0.05 vs baseline

**Results:** Significant improvement in PaO[sub]2[/sub] was observed during the eNP when compared with baseline. There were no significant changes in hemodynamic parameters when the negative pressures of −4, −8 and −12 cmH[sub]2[/sub]O were applied (fig. 22).

**Conclusions:** This study demonstrates effectiveness of extrathoracic negative pressure during recruitment of injured lung tissue in terms of arterial oxygenation and hemodynamic stability.

**References**

1. Lovas A. et al.: Biomed Res Int 2015; 2015: 478970.

## P49 Influence of extrathoracic negative pressure on mechanics of atelectatic lung during IPPV

### A. Fijalkowska-Nestorowicz^1^, J. Glapinski^2^, J. Wosko^3^

#### ^1^Medical University, Lublin, Poland; ^2^Wroc^3^aw University of Technology, Wroclaw, Poland; ^3^SPSK No4, Lublin, Poland

**Introduction:** Respiratory mechanics are regularly impaired when atelectasis occurs. There are many different procedures that can be used in order to reopen collapsed lung tissue. The aim of our study was to determine the effects of extrathoracic negative pressure (eNP) on respiratory mechanics of atelectatic lungs.

**Methods:** Ten Large White pigs weighting 52 ± 5 kg were included in the study. Under general anaesthesia all animals were tracheotomized (tube size 8.5–9 mm) and ventilated in a volume-controlled mode (P-B 840) at RR–18/min, with VT– 8–10 ml/kg, I:E ratio −1:2 and F[sub]I[/sub]O[sub]2[/sub]–1.0. Lungs injury was induced by repetitive lavages with 1.5–1.8 l warm 0.9% NaCl until PaO[sub]2[/sub]/F[sub]I[/sub]O[sub]2[/sub] remain stable below 100. After lungs injury was produced, the eNP, using a whole body size-box, was commenced in combination with IPPV. Peak airway pressure (P[sub]a[/sub][sub]w[/sub]peak), airway resistance (R) and dynamic compliance (Cdyn) were recorded, using ventilator software, before lungs injury and after – at 0 (baseline), −4, −8, −12 and −16 cmH[sub]2[/sub]O negative pressures.

**Results:** There were significant differences in P[sub]a[/sub][sub]w[/sub]peak and R but no in Cdyn at negative pressure −8, −12, and −16 cmH[sub]2[/sub]O compared to baseline (tab).

**Conclusions:** Extrathoracic negative pressure constitutes a positive profile of respiratory mechanics of atelectatic lungs and improves blood oxygenation.

**Table 10 (abstract P49).** See text for description

	Before	0 (cmH_2_O)	−4 (cmH_2_O)	−8 (cmH_2_O)	−12 (cmH_2_O)	−16 (cmH_2_O)
P_aw_peak (cmH_2_O)	19.9 ± 2.9*	29.4 ± 3.8	30.5 ± 6	26.2 ± 5.9	24.6 ± 5.9*	25.5 ± 4.6*
Cdyn (ml/cmH_2_O)	30.9 ± 4.5*	21.4 ± 8.9	20 ± 5.7	21.6 ± 5.8	23.1 ± 5.8	21.7 ± 3.9
R (cmH_2_O/l/s)	10.3 ± 2.6	13.3 ± 4.9	10.5 ± 3.8	7.8 ± 1.9*	6.7 ± 1.4*	6.4 ± 1.2*
PaO_2_/F_I_O_2_ (mmHg)	552 ± 56*	98.8 ± 42.5	259 ± 129*	416 ± 137*	503 ± 136*	576 ± 93*

Legend Table 10 *P < 0.05 vs baseline

## P50 Inspiratory negative pressure in simulated spontaneous breathing with a bag valve mask: a bench study

### F. Duprez^1^, T. Bonus^1^, G. Cuvelier^2^, S. Mashayekhi^1^, S. Ollieuz^1^, G. Reychler^3^

#### ^1^Epicura, Hornu, Belgium; ^2^Condorcet, Tournai, Belgium; ^3^UCL, Bruxelles, Belgium

**Introduction:** In emergency care, oxygen therapy can be administered directly with bag valve mask (BVM). In some cases, this method is applied to patient with a spontaneous breathing. The purpose of this study was to evaluate inspiratory negative pressure (INP) during spontaneous breathing through three different bag valve masks.

**Methods:** Three BVM (Ambu®Oval, MR100®, Ambu® Mark IV; without oxygen reservoir bag) were analyzed. Spontaneous breathing was simulated on a bench study. A two-compartment model of adult test lung (Dual Test Lung® DTL, Michigan Instrument) was connected to a Servo i® ventilator. One compartment of DTL was moved by Servo i®, the other as the driving compartment which simulated breathe. Servo i® was set in volume-controlled mode. Three-minute ventilation (MV) (Respiratory frequency (Rf): 10,20,30 cpm with tidal volume (Vt) of .45 L were analyzed. The peep was equal to 0 cm H2O and the Ti/Ttot was equal to 0.33. The compliance of DTL was set to .06 L/cm H2O and the initial resistance set to 5 cm H2O/L/sec. The change in inspiratory pressure was measured by an analog IWorx station/digital IWx/214 LabScribe II ® software. Three consecutive measurements were performed for each MV. Parameters were compared over time using ANOVA for each MV (p < .001).

**Results:** For all MV, no significant statistical differences were raised between Ambu®Oval and Ambu® Mark IV (ANOVA: p < .001).

**Conclusions:** For a same BVM, when MV increases, INP increases. For all MV, MR100® is the BVM is one who offers the lower resistance. This is most likely due to the type of patient valve of the BVMs, because patient valve of AMBUs® is a mushroom valve conception, while patient valve of MR100® is a duckbill valve conception. This last type of valve seems to offer less resistance than mushroom valve.

**Fig. 23 (abstract P50).**
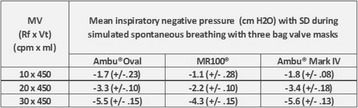
Legend 1: Mean inspiratory negative pressure with SD (cm H2O) during simulated spontaneous breathing with three bag valve masks

## P51 Evaluation of inspiratory negative pressure in simulated spontaneous breathing with a bag valve mask and intubate tube: a bench study

### T. Bonus^1^, F. Duprez^1^, G. Cuvelier^2^, S. Mashayekhi^1^, S. Ollieuz^1^, G. Reychler^3^

#### ^1^Epicura, Hornu, Belgium; ^2^Condorcet, Tournai, Belgium; ^3^UCL, Bruxelles, Belgium

**Introduction:** Bag valve masks (BVM) are frequently used in emergency unit or with intubated patients breathing spontaneously. The mask and the tube of intubation are thought to increase resistances and thus inspiratory resistance. The purpose of the present study is to evaluate:

1) The impact of BVM on the inspiratory negative pressure (INP) during simulated spontaneous breathing with intubate tube.

2) The additional effects of the intubation tube size and minute ventilation on the INP.

**Methods:** In a bench study, a bag valve mask (Ambu® Mark 4, without oxygen reservoir bag) was tested with three intubations tube (Internal Diameter 7,8,9 mm). Spontaneous breathing was simulated with a two-compartment model of adult test lung (Dual Test Lung® DTL, Michigan Instrument). DTL was connected to a Servo i® ventilator in volume-controlled mode. One compartment of DTL was moved by Servo i®, the other as the driving compartment which simulated breathing. Three-minute ventilations (MV) (Respiratory frequency: 10,20,30 cpm with volume tidal = .5 L) were analyzed. The peep was equal to 0 cm H2O and the Ti/Ttot was equal to 0.33. The compliance of DTL was set to 0.06 L/cm H2O and the initial resistance set to 5 cm H2O/L/sec. The change in inspiratory pressure was measured at entry of DTL, by an analog IWorx station/digital IWx/214 LabScribe II ® software. Three consecutive measurements were performed for each MV. Parameters were compared using ANOVA for each MV.

**Results:** For MV (10 × 500 ml): NO Statistical differences were found between A2 and A4, A5 and A1, A6 and A4, A3 and A1, A5 and A3. - For MV (20 × 500) and (30 × 500): Statistical differences were found between all groups ANOVA (p < .001).

**Conclusions:** With a bag valve mask, in spontaneous breathing, when MV increase, INP increase. Moreover, when intubation tube size decrease, the INP increase. For the same intubation tube size, when MV increase, INP increase. For the same MV and same intubation tube size, the addition of a BVM increase INP.

**References**

1) Abdo Khoury. From Mouth-to-Mouth to Bag-Valve-Mask Ventilation: Evolution and Characteristics of Actual Devices—A Review of the Literature. Biomed Res Int. 2014; 2014: 762053.

**Fig. 24 (abstract P51).**
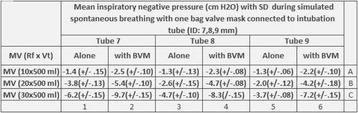
Legend 1: Mean inspiratory negative pressure (cm H2O) with SD during simulated spontaneous breathing with one bag valve masks connected to intubation tube (ID: 7,8,9 mm)

## P52 Withdrawn

## P53 Endothracheal tube cuff pressure assessment: anesthesiologists self–evaluation versus pressure manometer measurement

### I. Kuchyn, K. Bielka, A. Sergienko

#### Institute of Postgraduate Education Bogomolets National Medical University, Kyiv, Ukraine

**Introduction:** The safe level of endotracheal tube (ETT) cuff pressure during anesthesia is between 20 to 30 cm H2O. The over-inflated cuffs may cause different complications, e.g. sore throat, cough, aspiration and trachea injuries, including rupture. The use of cuff pressure manometer was recommended in several studies (1), hovewer it is still not a routine during anesthesia in many hospitals allover the world (2). Often skilled anesthesiologists are sure that they could evaluate normal and high pressure in the cuff by palpating it after intubation.

The aim of this prospestive onservational study was to estimate whether anesthesiologists could evaluate a safe (20–30 cm H2O), high (30–60 cm H2O) and very high cuff pressure (more than 60 cm H2O) by palpation of the external cuff ballon on an ETT.

**Methods:** The study was conducted in two Kyiv city hospitals at the department of anesthesiology and intensive care in 2015–2016. Anesthesiologists were asked to inflate an ETT cuff after intubation using their usual inflation technique and estimate by palpation if the cuff pressure is at the safe level, high level or very high level. After that the actual pressure in the cuff was validated with manometer measurements.

**Results:** ETT cuff pressure were measured in 146 patients by 24 anesthesiologists. The majority self-evaluate ETT cuff pressure as safe (86%), other as high (14%). The actual pressure in the cuff was safe in 26 (18%) of cases, high in 100 (68%) and very high in 20 (14%) of cases. There were no statistical difference between accuracy of ETT cuff pressure self-evaluation from one anesthesiologist to another.

**Conclusions:** Findings from this study shows that only in 18% of cases anesthesiologists could evaluate safe level of ETT cuff pressure by palpation of the ballon after intubation. Most of anesthesiologists underestimated the ETT cuff pressure. So, pressure manometer measurement of ETT cuff pressure should be implemented for safe medical practice.

**References**

1. Harm F, Zuercher M, Bassi M, Ummenhofer W. Prospective observational study on tracheal tube cuff pressures in emergency patients: is neglecting the problem the problem? Scand J Trauma Resusc Emerg Med. 2013;21:83. http://www.ncbi.nlm.nih.gov/pmc/articles/PMC4235018/. Accessed March 26, 2014.

2. Chan S-M, Wong C-S, Cherng C-H. Determining an optimal tracheal tube cuff pressure by the feel of the pilot balloon: a training course for trainees providing airway care. Acta Anaesthesiol Taiwan. 2009;47(2):79–83.

## P54 Evaluation of endotracheal cuff pressure on tracheal mucosal pressure

### H. Jones^1^, C. Day^2^

#### ^1^Plymouth hospitals NHS Trust, Plymouth, UK; ^2^Royal Devon and Exeter Hospital, Exeter, UK

**Introduction:** Despite the use of endotracheal tubes (ETT) with high volume, low pressure cuffs, concerns about tracheal mucosal damage and long term tracheal stenosis following intubation in ICU remain. Estimates of the incidence of laryngeal injury range from 15–94%. It is generally accepted that pressure exerted by the ETT cuff impairs mucosal blood flow and causes ischaemia. [1] We hypothesised that the lack of agreed ‘safe’ cuff pressure or relationship between cuff pressure and complications may be because cuff pressure is not a surrogate for mucosal pressure.

**Methods:** The study was performed using a cadaveric pig trachea. Sheridan endotracheal tubes in sizes 6,7,8 and 9 were used to intubate the trachea. The ETT cuff pressure was measured using a hand held manometer. A strain gauge was inserted under direct vision in a retrograde manor to align with the midpoint of the ETT cuff. The cuff was inflated in 1 ml increments and cuff pressure and applied pressure were recorded.

**Results:** We found no correlation between ETT cuff pressure and applied tracheal mucosa pressure for a size 6 or 7 ETT.

For size 8 and 9 ETT the graph of cuff pressure and applied pressure had an inflection point. Below the inflection point applied pressure remained low. Above the inflection point there was a linear relationship between applied pressure and cuff pressure.

**Conclusions:** We presume that for the smaller ETT, over the volumes tested, there is no circumferential contact between the cuff and trachea. The applied pressure remains independent of the cuff pressure. For size 8 and 9 ETT above the inflection point the cuff becomes in circumferential contact with the tracheal mucosa. The rise in cuff pressure is directly proportional to the applied mucosal pressure.

**References**

[1] Sultan et al. Endotracheal tube cuff pressure monitoring: a review of the evidence. J Perioper Pract. 2011 Nov;21(11):379–86.

**Fig. 25 (abstract P54).**
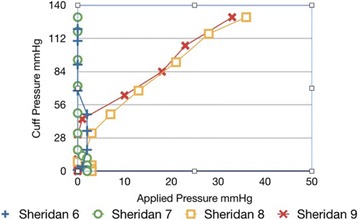
See text for description

## P55 Relation between risk of tracheal tube displacement and postural change of pretracheal tissue depth measured by ultrasound

### S. C. Park, S. R. Yeom

#### Pusan National University Hospital, Busan, South Korea

**Introduction:** The most likely cause of tracheal tube displacement is the increased pretracheal soft tissue thickness related to obesity. If postural change of pretracheal tissue depth increase, it may reduce the intratracheal length of the tube and induce tube displacement. Our purpose was to analyze relationship between difference of pretracheal tissue depth as postural change and risk of tracheal tube displacement.

**Methods:** We performed a prospective clinical trial from June 2014 to September 2015. Patients enrolled in the present study had been admitted to the Department of emergency medicine. We use a linear probe(Sonosite, M-Turbo) to measure each pretracheal tissue depth in neutral and extended neck. Neck extension is accomplished by placing the pillow under patient’s back.

**Results:** Seventy patients (male 49, female 21, mean age 53.9 18 [SD] years) were enrolled in this study. Pretracheal tissue depth difference was positively correlated with neutral depth(rho 0.629) and and BMI(rho 0.326).

**Conclusions:** Because Accidental decannulation is the most common serious complication associated tracheostomy, it is important to predict probability of tube displacement and use extended-length tube for risky patients. Pretracheal tissue depth difference as postural change measured by ultrasound was correlated well with risk factor of tubal displacement, pretracheal tissue depth and BMI. Pretracheal tissue depth difference as neck posture is helpful to predict risk of tracheostomy tube displacement.

**References**

1. Goldenberg D, Bhatti N. Management of the impaired airway in the adult. In: Cummings CW, ed. Otolaryngology Head and Neck Surgery. 4th ed. Philadelphia: Mosby; 2005:2441–53.

2. De Leyn P, Bedert L, Delcroix M, et al. Tracheotomy: clinical review and guidelines. Eur J Cardiothorac Surg 2007;32:412–21. Epub 2007 Jun 27.

3. Kost K. Percutaneous tracheotomy: a prospective evaluation of 500 consecutive cases. Laryngoscope 2005;115:1–.30.

4. McCormick T, Venn R. Recently published papers: Tracheostomy: why rather than when? Obesity: does it matter? And stroke: diagnosis, thrombosis and prognosis. Crit Care 2007;11:127.

5. El Solh AA, Jaafar W. A comparative study of the complications of surgical tracheostomy in morbidly obese critically ill patients. Crit Care 2007;11:R3.

6. Byhahn C, Lischke V, Meininger D, et al. Peri-operative complications during percutaneous tracheostomy in obese patients. Anaesthesia 2005;60:12–5.

7. Darrat I, Yaremchuk K. Early mortality rate of morbidly obese patients after tracheostomy. Laryngoscope 2008;118:2125–8.

8. Szeto C, Kost K, Hanley JA, et al. A simple method to predict pretracheal tissue thickness to prevent accidental decannulation in the obese. Otolaryngol Head Neck Surg 2010;143:223–29.

9. Walker RN, Alexander IJ et al. Anthropometric measurements: Effect of CT depth of pretracheal soft tissue on tracheostomy tube selection. Am J Neuroradiol 2012;33:449–52.

## P56 Safety and efficacy of overnight intubation compared to tracheostomy for postoperative airway management in head and neck cancer patients undergoing surgery

### S. N. Myatra, S. Gupta, V. Rajnala, J. Divatia

#### Tata Memorial Hospital, Mumbai, India

**Introduction:** Tracheostomy has been used traditionally to provide secure airway in patients undergoing radical resections for head and neck cancer. However, recent studies have questioned these practices, emphasizing endotracheal intubations to be equally safe. We planned a study to determine the safety and efficacy of overnight intubation followed by extubation the next morning (ETT group) as compared to tracheostomy (TT group) for postoperative airway management and to audit our airway management practices for major head and neck cancer surgeries.

**Methods:** A prospective observational audit was conducted over one year at Tata Memorial Hospital from August 2015 to July2016. Adult patients undergoing intraoral surgeries were included. Patients undergoing emergency surgery and having a tracheostomy prior to enrolment were excluded. Extent of the disease, type of surgery, demographic details of the patient, anaesthesia details and airway management details were recorded. These patients were followed up in the post-operative period until discharge. Time to extubation was recorded in the ETT group. Time to oral intake, speech, complications until discharge and length of hospital stay were recorded in both groups.

**Results:** We screened 4477 patients and included 720. There were 417 patients in ETT group and 303 patients in TT group. As compared to TT group, ETT group had shorter stay in hospital (7.2 ± 3.7 versus 11.5 ± 7.2 days, p = 0.00), less time to oral intake (5.1 ± 1.6 versus 7.2 ± 2.8 days, p = 0.00), and less time to speech (3.6 ± 1.6 versus 6.1 ± 2.7 days p = 0.00). The overall complications (4.3% versus 22.4%, p = 0.00) and airway related complications (1.68% versus 8.58%, p = 0.00) were lower in the ETT group compared to the TT group.

**Conclusions:** The airway in intra-oral major head and neck cancer surgery can be managed safely with overnight intubation with faster return to oral feeding and speech. Tracheostomy should be performed only in select patients.

**References**

1. Scher, N., Dobleman, T. J. and Panje, W. R. (1989), Endotracheal intubation as an alternative to tracheostomy after intraoral or oropharyngeal surgery. Head Neck, 11: 500–504. doi: 10.1002/hed.2880110605

2. Agnew J(1), Hains D, Rounsfell B, Aust N Z J Surg. 1992 Aug;62(8):652–3. Management of the airway in oral and oropharyngeal resections

3. Randell T, Söderholm AL, Lindqvist C Is nasotracheal intubation safe in surgery for mandibular cancer?Arch Otolaryngol Head Neck Surg. 1992 Jul; 118(7):725–8

## P57 Prognosis of early tracheostomy a patients with severe head trauma (tces)

### J. Villalobos Silva^1^, O. Aguilera Olvera^1^, R. Cavazos Schulte^1^, M. Castañeda Bermudez^1^, L. Pariente Zorrilla^1^, H. Lopez Ferretis^1^, K. Trejo García^2^

#### ^1^Hospital General “Norberto Treviño”, CD. Victoria, Mexico; ^2^Hospital Infantil de Tamaulipas, Cd. Victoria, Mexico

**Introduction:** Treatment of neurocritical patients is complex due to their different types of brain injury, however the realization of early tracheostomy shown to have lower incidence of pneumonia associated with mechanical ventilation (VAP), reduced hospital stay, lower mortality and lower costs hospital.

**Methods:** Prospective observational study corresponding to patients with TCEs admitted to the Intensive Care Unit (ICU) of the General Hospital of Cd. Victoria “Norberto Trevino” over two years (2014–2015). Patients over 18 years were grouped into two groups; “Early” or “late” using as a cutoff 7 days. Tracheostomy We analyzed: age, sex, APACHE II, VM days, incidence of pneumonia associated with VM, days in ICU, ICU mortality. Continuous variables were expressed as mean and standard deviation, categorical variables as absolute value and percentage, test Students were used for independent samples to compare numerical variables and Chi-square test for categorical whether ordinal or nominal, measures of association with OR. SPSS v.23.

**Results:** Early vs late tracheotomy tracheostomy: 62 patients (68% male) with severe head injury, divided into 2 groups they were included. Trach average age <7 days 27.7 ± 5.4 (22–35) vs Trach > 7 days 23.9 ± 10.1 (18–50) p = 0.672, VM days in Trach <7 days 8.5 ± 4.1 (4–13) vs Trach > 7 days 14.0 ± 3.0 (9–21) p <0.05, sedation days Trach <7 days vs 5.0 ± 2.3 Trach > 6.9 ± 3.0 7 days p = 0.21, APACHE in Trach <7-day 16.5 ± 7.4 (10–25) Trach vs > 7-day 15.0 ± 3.2 (10–22) p = 0.53, ICU days in Trach <7 days 10 ± 4.83 (4–14) vs Trach > 7 days 14.9 ± 4.00 (10–26) p <0.04, complications Trach <7-day 27.3% (12n) vs Trach > 7 days 72.7% (32n) p <0.001, mortality Trach <7 days 28.6% (4 N) vs Trach > 7 days 71.4% (10n) p = 0.34. Trach prevalence of VAP with <46% vs 7-day Trach > 7 days 84%. Pneumonias in Trach <7 days OR 0.160 (0.100–0.890).

**Conclusions:** In this study in the Intensive Care Service General Hospital “Norberto Trevino” We conclude that the effectiveness of conducting early days of VM decreases tracheostomy, ICU stay and ventilator associated pneumonia (VAP).

## P58 Withdrawn

## P59 Percutaneous vs surgical tracheostomy: an incidence rate of stoma infection in the neurosurgery ICU of the single tertiary level hospital

### N. Balciuniene, J. Ramsaite, O. Kriukelyte, A. Krikscionaitiene, T. Tamosuitis

#### Lithuanian University of Health Sciences, Kaunas, Lithuania

**Introduction:** One third of all patients undergo open standard tracheostomy (ST) or bedside percutaneous dilatational tracheostomy (PDT) in our 18 bed neurosurgery ICU (NICU). The aim of our study was to evaluate the incidence rate of stoma infection after tracheostomy according to the type of procedure (PDT vs ST) in NICU patients.

**Methods:** We performed a retrospective chart review of 240 patients who underwent tracheostomy during their stay in the NICU at our 2200 bed university teaching hospital Kaunas Clinics from October 2012 to December 2015. The data from 202 patients (140 males) with median age 58.4 (18–80) who met the inclusion criteria were used for further analysis. There were 84 patients in PDT group and 118 patients in ST group.

**Results:** There was no significant difference between two groups in terms of age, sex, timing of tracheostomy, duration of ICU stay, mortality and infectious status before the procedure. The incidence rate of stoma infection was significantly lower in PDT group vs ST group (16.7% vs 65.3%, p < 0.001). We performed the logistic regression adjusting for age, sex, the cause of hospitalization, tracheostomy strategy and timing of the procedure. The use of ST was associated with an increase in the risk of stoma infection (OR = 8.83 95% 4.40–17.71).

**Conclusions:** The incidence rate of stoma infection was significantly higher in ST group compared to PDT group in NICU patients.

## P60 Occurrence of ventilator associated pneumonia using tracheostomy tubes with subglottic secretion drainage

### P. Terragni ^1^, L. Brazzi^2^, D. Falco^2^, L. Pistidda^1^, G. Magni^3^, L. Bartoletti^2^, L. Mascia^3^, C. Filippini^2^, V. Ranieri^3^

#### ^1^University of Sassari, Sassari, Italy; ^2^Città della Salute e della Scienza Torino, Torino, Italy; ^3^Sapienza University of Rome Policlinico Umberto I Hospital, Roma, Italy

**Introduction:** Ventilator-Associated Pneumonia (VAP) represents a cause of morbidity and mortality in critically ill patients who require mechanical ventilation. Subglottic secretions above the endotracheal cuff are associated with bacteria colonization of lower respiratory tract, causing VAP. A preventive strategy to avoid subglottic secretions progression is to remove it by drainage with the use of special tracheal tubes effective in preventing both early onset and late onset VAP.[1] The purpose of this study is to measure VAP incidence in tracheostomized patients with suction above the cuff.

**Methods:** Study design: matched cohort study with historical control in 3 academic italian ICUs.

Procedures and measurements: upon admission to ICU, patients requiring mechanical ventilation were submitted to tracheostomy with a tracheal tube allowing drainage of subglottic secretions (treatment group) to reduce the incidence of VAP. A control group of tracheostomized patients without the ability of suctioning above the cuff was created applying the propensity score matching technique on a dataset including patients enrolled in the previous ELT study [2].

Primary endpoint: occurrence of post-tracheostomy VAP incidence (as determined by clinical pulmonary infection score) at 28-days from intubation. Secondary endpoints: 28-days mortality after tracheostomy; number of ventilator-free days and ICU-free days at 28-days from intubation; time of decannulation, total number of days of inpatient hospital stay; assessment of SOFA score.

**Results:** 125 patients were enrolled in the treatment group from July 2014 to April 2016; 232 patients without suctioning were selected as a control group. Overall incidence of VAP was 10 patients (8%) in treatment group and 45 patients (19.4%) in the control group (p value = 0.004) with OR: 0.361 and CI (0.175; 0.745). In order to balance the two groups for timing of tracheostomy, gender, age, SAPS and SOFA covariates, a propensity score matching was performed: VAP incidence (120 patients) was 8.3% and 21.7% in treatment and control groups respectively (p value = 0.0408) with OR: 0.329 and CI (0.109; 0.990).

**Conclusions:** Subglottic secretions drainage reduces incidence of VAP in critically ill patients requiring ongoing mechanical ventilation via tracheostomy.

**References**

1. Lacherade JC, et al.: Am J Resp Crit Care Med 2010;182:910–7.

2. Terragni PP, et al.: JAMA 2010;303:1483–9.

## P61 Withdrawn

## P62 Withdrawn

## P63 The effect of sepsis in the outcome of spontaneous breathing trial

### A. Kyriakoudi, N. Rovina, O. Koltsida, E. Konstantellou, M. Kardara, E. Kostakou, G. Gavriilidis, I. Vasileiadis, N. Koulouris, A. Koutsoukou

#### ICU, 1st Department of Pulmonary Medicine, “Sotiria” Hospital, Athens Medical School, Athens, Greece

**Introduction:** Spontaneous breathing trial (SBT), a routine procedure during ventilator weaning, entails cardiopulmonary stress for the ventilated patient, which is higher in the patients failing the trial. SBT has been shown to activate an intense inflammatory response, with an increase of cytokines in plasma such as TNF-a, IL-6, IL-1b, and IL-18, a pro-inflammatory cytokine which is being considered as a potential sepsis biomarker. Sepsis has been associated with severe muscle wasting and/or ICU-aquired weakness. Aim of this study was to investigate the effect of sepsis on weaning outcome in critically ill patients.

**Methods:** A total of 55 intubated and mechanically ventilated patients were included in the study (29 with a septic condition, 26 non septic, intubated for other reasons). Patients were assessed during a 30 minute SBT trial SBT. Blood samples were collected at baseline and at the end of the 30 minute trial. IL-18, caspase-3, TLR-2, IL-6, and TNF-á were measured by ELISA and were correlated with markers of inflammation and the weaning outcome.

**Results:** In these preliminary data we included 29 mechanically ventilated patients (53%) with a septic condition and 27 (47%) patients with other than sepsis conditions. Septic patients had a higher percentage of weaning failure compared to non septic ones (38% vs 27%), and this was associated with statistically significantly lower levels of serum albumin (p = 0.026) and CRP (p = 0.05). No statistically significant differences were observed in the cytokine levels between the failure/success groups in septic and non-septic patients, as well as, at the two measurement time points. However, IL-18 levels were higher in septic patients that failed SBT at 30 mins compared to the success group [192 (75–583) vs 147 (12.5–581), median (range)]. In both septic and non-septic patients that failed SBT the stress during the trial led to an increase of IL-18 levels (although, non statistically significant). Finally, IL-18 was correlated at both time points with serum albumine (p = 0.012 and p = 0.011, respectively, and r = −0.344 and r = −0.347, respectively), PCT (p < 0.001, r = 989), and APACHE II score (p = 0.05, r = 0.383)

**Conclusions:** Although the number of patients per group is small in these preliminary data, our results indicate that SBT failure in septic patients was associated with a higher inflammatory profile of the patients. Among all the measured cytokines, IL-18 seems to reflect the severity of sepsis associated with weaning failure.

## P64 Dysphagia management in Dutch intensive care units: a nationwide postal survey

### W. Van Snippenburg^1^, A. Kröner^1^, M. Flim^1^, M. Buise^2^, R. Hemler^1^, P. Spronk^1^

#### ^1^Gelre Hospitals Apeldoorn, Apeldoorn, Netherlands; ^2^Catharina Hospital Eindhoven, Eindhoven, Netherlands

**Introduction:** Dysphagia is a common problem in the intensive care unit (ICU), and has been associated with prolonged ICU length of stay and increased risk of pneumonia, reintubation and death. However, no national guidelines on dysphagia prevention, screening and management exist for ICU patients. Therefore, we performed a national survey to learn what strategies are being used in Dutch ICUs.

**Methods:** A survey was developed based on current literature and experts’ opinions. It comprised both open and multiple choice questions regarding hospital and intensive care characteristics, perceived prevalence and importance of dysphagia, screening strategies including the use of certain diagnostic tests and specialist consultation, modalities used to prevent complications such as aspiration, and interventions used to improve swallowing function and follow-up. It was sent to all 90 non-pediatric ICUs in The Netherlands with two reminders by telephone and e-mail.

**Results:** 67 of 90 addressed ICUs (74%) replied to our survey. A median relevance score of 4 (IQR 4–5) out of 5 was given to the topic of dysphagia. In 22% of ICUs, patients were always screened for dysphagia after extubation, in 45% of ICUs screening was always performed after tracheotomy. The water swallow test was always part of the work-up in 88% of ICUs. Fiberoptic endoscopic evaluation of swallowing (FEES) was used as the gold standard in 60% of ICUs, versus videofluoroscopic swallowing study (VFSS) in 25%. In 49% of ICUs no standardized active rehabilitation protocol for dysphagia existed. In the remaining 51% swallowing exercises supervised by the speech language pathologist were part of standard rehabilitation, occasionally supplemented by electrical stimulation or sEMG biofeedback training in 6% and 10%, respectively.

**Conclusions:** Despite the known possible consequences of dysphagia and it being considered relevant by the majority of respondents, most Dutch ICUs do not regularly screen for dysphagia after extubation or tracheotomy and almost half do not have a treatment or rehabilitation protocol. The diagnostic tests and therapies used vary between hospitals, including some of unproven validity. A well-defined screening and treatment algorithm based on an evidence-based guideline could provide more standardized approach to this problem, thus improving quality of care.

## P65 The respiratory pressure – abdominal volume curve in a porcine model

### A. Regli^1^, B. Noffsinger^2^, B. De Keulenaer^1^, B. Singh^2^, L. Hockings^3^, P. Van Heerden^4^

#### ^1^Fiona Stanley Hospital, Perth, Australia; ^2^SCGH, Perth, Australia; ^3^The Alfred Hospital, Melbourne, Australia; ^4^Hadassah University Hospital, Jerusalem, Israel

**Introduction:** Intra-abdominal hypertension can increase airway pressures and impair ventilation. Little is known about the relationship between increasing intra-abdominal volume (IAV) and airway pressures. We asseseds the effect of increasing IAV on airway and intra-abdominal pressures (IAP) in a porcine model

**Methods:** Seven pigs (41.4 +/−8.5 kg) received standardized anesthesia and mech. ventilation. A latex balloon in the abdomen was inflated in one liter steps until IAP exceeded 40 cmH2O. At each step, we measured peak airway pressure (pPAW) and IAP.

**Results:** Raising IAV led to an exponential increase of pPAW and IAP while decreasing both abdominal and respiratory system compliance. pPAW increased by approx. 40% of the increase of IAP.

**Conclusions:** The exponential nature of IAV on pPAW and IAP implies that small reductions in IAV may lead to significant reductions in airway and abdominal pressures in the presence of high grades of intra-abdominal hypertension. Treatment to reduce IAP may be more effective at high grades of IAH.

**Fig. 26 (abstract P65).**
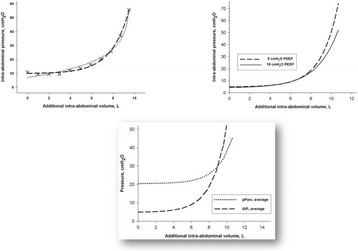
Legend 1: Pressure, volume and PEEP relationships in intra-abdominal hypertension

## P66 Distribution of tidal ventilation in potential lung donors: a pilot observational study

### C. Spina^1^, A. Bronco^2^, F. Magni^2^, C. Di Giambattista^1^, A. Vargiolu^2^, G. Bellani^1^, G. Foti^1^, G. Citerio^1^

#### ^1^University of Milano Bicocca, Monza, Italy; ^2^San Gerardo Hospital, Monza, Italy

**Introduction:** Lung donation occurs in a limited number of potential donors due to the deterioration of lung function after brain death and the strict eligibility criteria. “Protective” ventilator strategy markedly increased lung donation rate [1]. However, to our knowledge no studies have investigated individualization of PEEP and recruitment maneuvers in this setting. Thus, we explored the use of electrical impedance tomography (EIT) in a hypothesis-generating study.

**Methods:** Single center observational study. Potential lung donors underwent a 10 minutes evaluation by EIT (PulmoVista 500 Dräger Medical) after neurologic determination of death. Potential donors were ventilated with a “protective” protocol. Gas analysis and respiratory system compliance (Crs) were assessed. Two regions of interest were defined: non-dependent lung zones (ROIn-dep) and dependent lung zones (ROIdep). We measure the Vt distending each region (VtROIn-dep, VtROIdep); the heterogeneity of Vt distribution (VtH) and regional values of compliance (CrsROInon-dep, CrsROIdep). Results are expressed as median (Q1; Q3).

**Results:** 5 subjects were enrolled. Vt was 7.4 (7.1; 7.7) ml/Kg IBW and PEEP was 8 (8; 8) cmH20. PF ratio was 358 (47; 560) and Crs was 43 (35; 76) ml/cmH2O. Vt was preferentially distributed in non-dependent lung zones because of higher CrsROInon-dep 26 (20; 43) ml/cmH2O vs. CrsROIdep 18 (13; 32). For clarity, VtROInon-dep was 252 (196; 285) ml while VtROIdep was 166 (143; 197) ml and VtH was 1.35 (1.09; 2.02).

**Conclusions:** EIT monitoring showed that ventilation is preferentially distributed in ventral lung zones when the protective ventilator strategy is employed in potential organ donors. Next step will be to verify whether PEEP titration and recruitment maneuver based on EIT findings contribute to enhance respiratory performance and suitability for lung transplantation.

**References**

[1] Mascia, 2010 JAMA 304:2620–7

**Fig. 27 (abstract P66).**
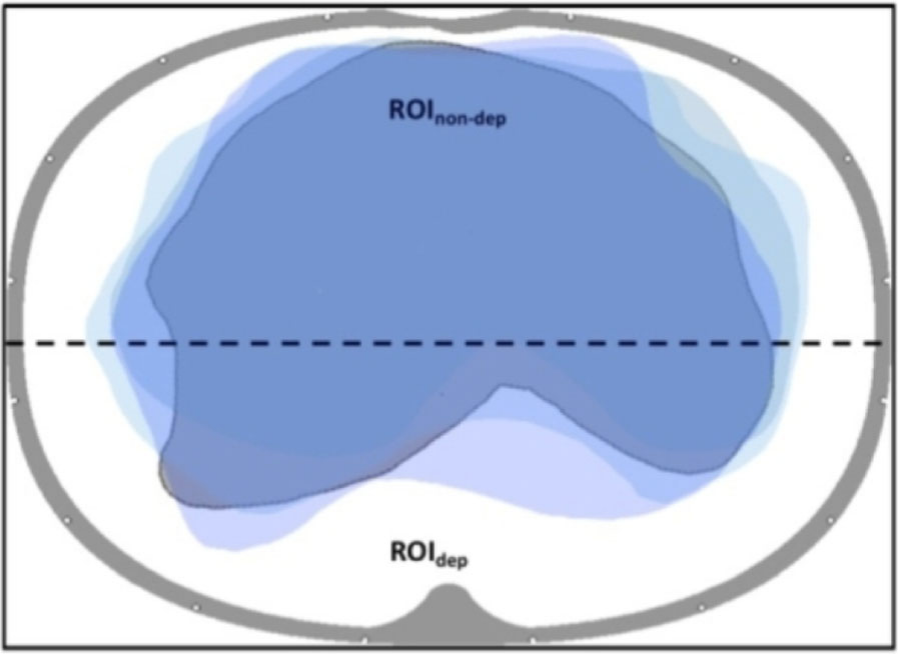
Legend 1: Tidal ventilation in each individual subject

## P67 Pixel-level pressure-volume curves predict lung recruitability: pilot study on electrical impedance tomography (EIT) in acute respiratory failure (ARF) patients

### G. Scaramuzzo^1^, S. Spadaro^1^, A. D. Waldmann^2^, S. H. Böhm^2^, R. Ragazzi^1^, C. A. Volta^1^

#### ^1^University of Ferrara, Ferrara, Italy; ^2^Swisstom AG, Landquart, Switzerland

**Introduction:** Recruitment Maneuvers (RM) typically consist of high airway pressures for prolonged periods of time to open closed lung units. Despite potentially positive effects, RM can cause hypotension, desaturation, or pneumothorax. Different methods have been proposed to identify patients who can benefit from a RM but conclusive recommendations are still lacking. We hypothesized that the shape of a pixel-level pressure-volume curve (PVpx) could predict recruitability defined as Recruited Volume (RV) after a RM at rising PEEP levels.

**Fig. 28 (abstract P67).**
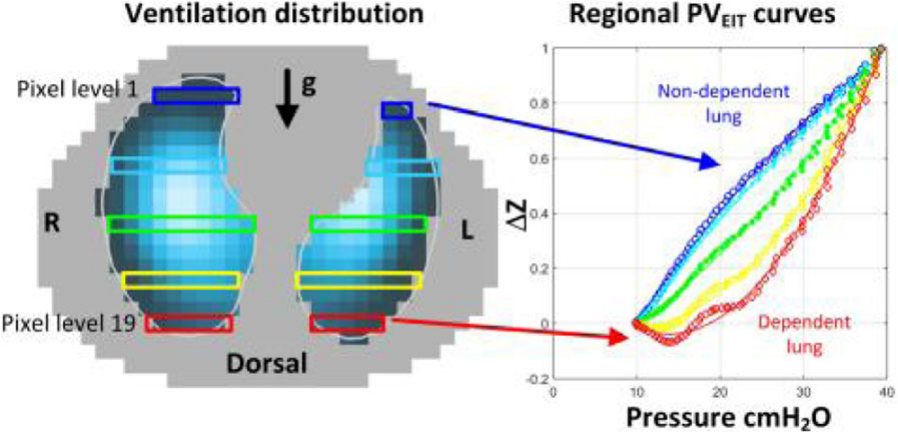
Legend 1: Pixel-level pressure-volume curves

**Methods:** 12 ARF patients (P/F < 300 mmHg) were mechanically ventilated with TV of 6–8 ml/kg. A 5-step PEEP trial - increasing (5,10,15 cmH[sub]2[/sub]O) and decreasing (10,5 cmH[sub]2[/sub]O) limb - was performed with a RM (inspiratory hold,40 cmH2O CPAP for 40s) between PEEP10 and PEEP15. At each step, lung mechanics and EIT data were recorded during a quasi-static pressure-volume curve (PV) maneuver. The PV before the RM was adapted to an isogravitational pixel level plotting the variation of impedance in each pixel row and the variation of pressure in the respiratory system (fig. 28); 19 pixel-level PV curves (PVpx) were obtained [1] and fitted in the equation V = a + b•Pao + c•Pao^2^[2]. The “c” factor derived from the fitting (C) indicates the shape of the curve: positive C is related to a compliance increase during the inflation, while negative C to its reduction. We correlated the RV after the RM with the C before the RM at each pixel level, from non-dependent (pixel 1) to dependent lung (pixel 19).

**Results:** The C had a significant positive correlation with RV (ml/kg/PBW) for pixel levels 13–17,19 (dependent lung); positive PVpx values predicted recruitability of the dependent lung. In the central lung, no correlation was founded. A negative (non-significant) correlation was founded in non-dependent lung indicating that a preexisting non-dependent overinflation could be inversely correlated with the effects of the RM.

**Conclusions:** The C factor from PVpx predicts lung recruitability at the bedside and could help to identify patients who might benefit from lung RM.

**References**

1. Kunst PW et al.: Crit Care Med 2000 Jan;28(1):178–83

2. Ranieri MV et al.: Am J Respir Crit Care Med. 1994 Jan; 149(1):19–27

## P68 Regional distribution of alveolar collapse and overdistension assessed by electrical impedance tomography in ARDS patients

### S. J. Heines, U. Strauch, M. C. Van de Poll, P. M. Roekaerts, D. C. Bergmans

#### University Hospital Maastricht, Maastricht, Netherlands

**Introduction:** Aim: determine the effect of PEEP on alveolar collapse (CL) and overdistension (OD) in ARDS.

Lung recruitment decreases CL but may induce regional OD. PEEP may be optimal when the balance between CL and OD is optimal. The distribution of this balance and changes in local ventilation distribution at varying PEEP levels throughout different lung fields in ARDS is unknown. Regional OD, CL and centre of ventilation (COV, a measure of homogeneity of ventilation distribution) can be visualized using Electrical Impedance Tomography (EIT).

**Methods:** Ten ARDS patients (P/F-ratio 170, SD 53) were studied. Ventral and dorsal OD and CL (% overdistended or collapsed alveoli) and COV were measured by EIT during an incremental and decremental PEEP trial (8–20 cmH2O). COV was expressed as dorsal to ventral ventilation distribution. A COV of 50% indicates an equal distribution between ventral and dorsal lung fields. COV >50% indicates a shift of ventilation distribution towards ventral. We estimated the balance between overdistended and collapsed alveoli by subtracting CL from OD (ODCL). An ODCL of 0% indicates an optimal balance between CL and OD. Data are expressed as mean ± SEM. 1 or 2-way ANOVA was used as appropriate.

**Fig. 29: (abstract P68).**
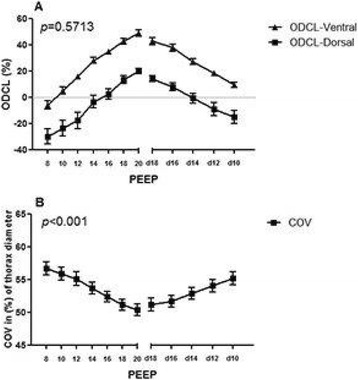
See text for description

**Results:** At 8 cmH2O, dorsal ODCL (−30% ±6) was significantly lower than ventral ODCL (−6% ±3) (p < 0.001). During the increment in PEEP, ODCL significantly increased to +20% ±2 dorsally and +49% ±2 ventrally (p < 0.001), the magnitude of this increase was similar in both lung fields (p = 0.6, Fig. 29a). The COV shifted dorsally from 57% ±1 to 50% ±1 (p < 0.001, Fig. 29b).

**Conclusions:** The balance between CL and OD at varying PEEP levels differs between ventral and dorsal lung fields. Lung recruitment reduces CL and increases ventilation in dorsal lung fields and a dorsal shift of the centre of ventilation. However simultaneously ventral OD occurs. When determining optimal PEEP settings, variations in the balance between CL and OD between lung fields should be taken into account. Visualization of local ventilation by EIT appears to be a useful tool for this.

## P69 A calibration technique for the estimation of lung volumes in non intubated subjects by electrical impedance tomography

### S. Sosio, S. Gatti, E. Maffezzini, V. Punzi, A. Asta, G. Foti, G. Bellani

#### University of Milano Bicocca, Milan, Italy

**Introduction:** Electrical Impedance Tomography (EIT) is a bedside monitoring technique of regional ventilation that measures the changes in the impedance within the thorax. The tight correlation between variation of impedance (ΔZ) and variation of lung volumes (Vt) is known. Unless the Vt is measured by an external reference (e.g. spirometry) its absolute value (in ml) cannot be determined, however measurement of Vt would be useful in non intubated subjects.

**Methods:** We performed a prospective study on thirteen healthy volunteers (>18 years) without obesity, pregnancy, chest circumference <75 cm and history of pulmonary or medical disorders. The subjects were connected to the EIT monitor.

Calibration phase: the subjects breathed 10 times, from functional residual capacity, fully distending a non-elastic bag with a volume of 1680 ml. In this way the conversion factor between Vt and ΔZ (K[sub]Vt/ΔZ[/sub]) was found for each subject.

Validation phase: tidal volumes were estimated (estVt = ΔZ* K[sub]Vt/ΔZ[/sub]) from impedance variations; the accuracy was assessed measuring tidal volumes (Vt) with a ventilator through which the subjects breathed by a mouthpiece. Four different ventilator settings were used changing pressure support ventilation (PSV) and positive end expiratory pressure (PEEP).

**Fig. 30 (abstract P69).**
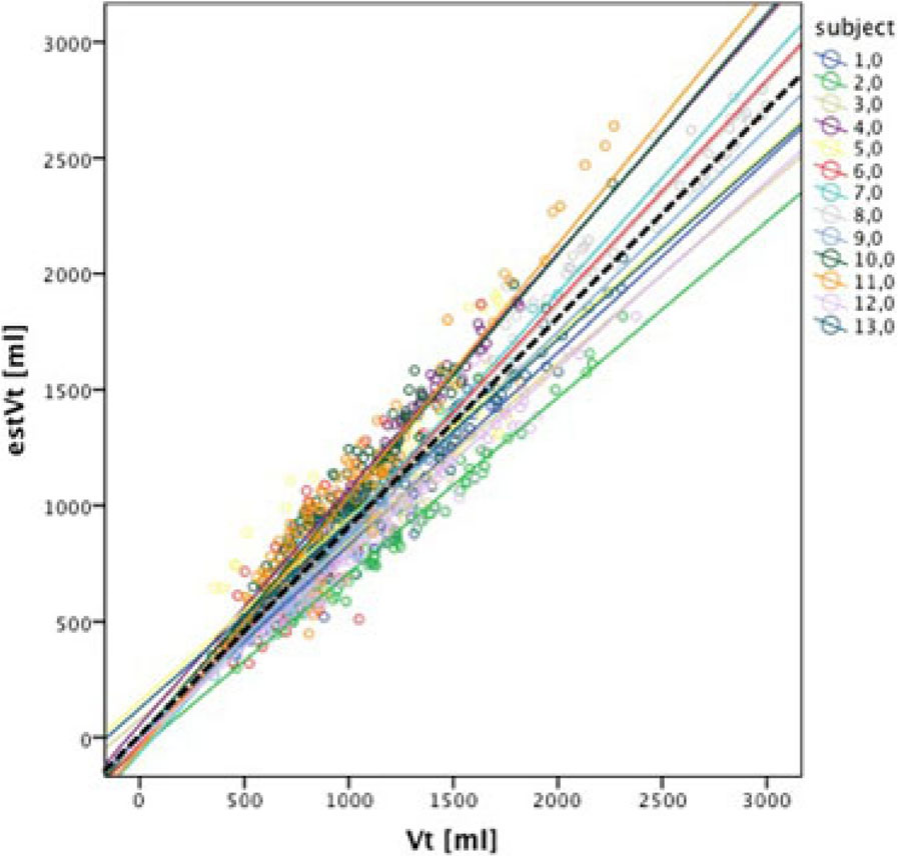
See text for description

**Results:** The correlation between Vt and estVt was tight (r^2^ = 0.89) with a within-subject mean r^2^ of 0.91 ± 0.07. The fit equation was estVt = 0.9 * Vt + 10.1 (Fig. 30). The highest correlation was found at PEEP 0 (mean: estVt = 0.93 * Vt) vs PEEP 8 (mean: estVt = 0.8 * Vt), p = 0.01. No differences were found between PSV 0 (mean: estVt = 0.97) and PSV 8 (mean: estVt = 0.93 * Vt), p = 0.50. The Bland-Altman plot showed a systematic bias of −95,5 ml (−10%) and 95% CI of -396 ml (−40%); 205 ml (20%).

**Conclusions:** A simple and fast technique of calibration of the EIT can allow a reliable and non-invasive measure of Vt in non intubated subjects.

## P70 Low frequency forced oscillation technique in lung recruitment maneuver monitoring

### J. Glapinski ^1^, J. Mroczka^1^, A. Nestorowicz^2^, A. Fijalkowska-Nestorowicz^2^

#### ^1^Wroclaw University of Science and Technology, Wrocław, Poland; ^2^Lublin Medical Univesity, Lublin, Poland

**Introduction:** The recruitment maneuver (RM) is a part of clinical strategy of respiration supporting, The subject of the research was to monitor the phenomena occurring during RM when the artificial ventilation is applied at the mouth of animal ARDS model, using modified low frequency forced oscillation technique (FOT).

**Methods:** The phenomenon of the opening alveoli was modelled according to the estimations made in the complex mathematical model of respiratory system. The complex model structure was reduced into its simpler, identifiable analog of nonlinear resistance and capacitance of the lung.

A Large White pig weighing 50 ± 5 kg were included into the study. Under general anaesthesia the animal was tracheostomised, intubated and ventilated in a volume-controlled mode (Puritan Bennett 840) at RR–18/min, with VT– 8–10 ml/kg, I:E ratio −1:2 and FIO2–1.0. Lungs injury was induced by repetitive lavages with 1.5–1.8 l warm 0.9% NaCl.

The low frequency (0,5Hz) sinusoidal flow oscillations were induced at the intubation tube by means of mechanically controlled syringe. The flow and pressure changes were monitored by pneumotachograph (Hans Rudolph) while the external negative pressure (eNP) in the whole body size-box was changing. For the estimation of time-varying mechanical parameters (Rest and Cest) of the lung the Kalman filter method of data analysis was used.

**Fig. 31 (abstract P70).**
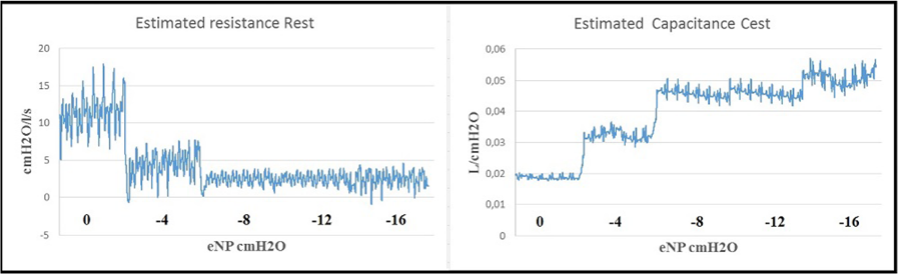
See text for description

**Results:** Application of the proposed method of the identification of the parameters of lung during the vacuum changes in the chamber results as in the example figure (Fig. 31). There were significant differences in Rest and in Cest at the body size-box negative pressure 0, −4, −8, −12, and −16 cmH2O.

**Conclusions:** The modified low frequency forced oscillation technique allows observation of changes in the parameters typical for recruited lungs and assess the possibility of monitoring of changes of physiological parameter values when the RM is applied in artificially ventilated lungs.

## P71 Evaluation of ventilation-associated lung injury, respiratory mechanics and work of breathing by tracheal and esophageal pressure monitoring in pressure support ventilation

### A. I Yaroshetskiy^1^, N. A. Rezepov^2^, I. A. Mandel^3^, B. R. Gelfand^1^

#### ^1^Pirogov Russian National Research Medical University, Moscow, Russia; ^2^City Hospital#67, Moscow, Russia; ^3^Federal research and clinical center for special methods of healthcare and medical technology of FMBA, Moscow, Russia

**Introduction:** There is limited data on safe tidal volume and pressure setting in pressure support ventilation (PSV).

**Methods:** We included 59 mechanically ventilated patients in SICU with PSV mode. We used monitoring of airway pressure, tracheal pressure, and esophageal pressure, plotted dynamic «tracheal pressure-volume» (Ptr-Vt) and «esophageal pressure-volume» (Pes-Vt) loops. We measured tidal volume (Vt), patient’s work of breathing (WOBp), delta esophageal pressure (ΔPes), plateau pressure (Pplat), transpulmonary plateau pressure (Ptp plat), transpulmonary pressure at PEEP (Ptp PEEP), dynamic compliance of respiratory system (Cdyn), dynamic lung compliance (Clung dyn), dynamic chest wall compliance (Ccw dyn), delta transpulmonary pressure (ΔPtp = Ptp plat - Ptp PEEP), tracheal pressure at PEEP level (PEEPtr), minimal tracheal pressure during triggering (Ptrig tr), and calculated difference between PEEP tr and Ptrig tr. After that we plotted loops and estimated its shape. We collected data at 6 steps: (1) at baseline PS and PEEP level, (2) at PS + 4 mbar level and baseline PEEP, (3) at PS-4 mbar level and baseline PEEP, (4) at PEEP + 4 mbar level and baseline PS, (5) at PEEP set by end-expiratory transpulmonary pressure level (PEEPtp0) and baseline PS, and then (6) at PEEP-4 mbar level and baseline PS.

**Results:** We discovered 3 typical shapes of inspiratory part of «tracheal pressure–time» (Ptr-t) curve: triangle, square and S-shape. We found significant differences between different shapes of inspiratory part of «Ptr-t» curve in WOBp (p < 0.0001), ΔPes (p < 0.0001), PEEPtr-Ptrig tr (p < 0.0001), and PS-ΔPtp (p = 0.002). We discovered 4 types of dynamic «Ptr/Vt» loops: inverted (n = 41), classical (n = 7), linear (n = 7), and S-shaped (n = 4). We found significant differences between different shapes of dynamic «Ptr/Vt» loops in ΔPtp (p = 0.05) и Clung dyn (p = 0.020), which allowed to estimate delta transpulmonary pressure without esophageal pressure monitoring. We found significant differences between different shapes (v-,u-,w- and v + −shaped) of inspiratory part of «Pes-t» curve in WOBp (p = 0.002), PEEPtr-Ptrig tr (p = 0.05), PS-ΔPtp (p = 0.034), and Ccw dyn (p = 0.014). Vt was more than 6 ml/kg of ideal body weight (IBW)(7.8 (6.9–9.1)ml/kg) in 88.1% of patients at baseline while ΔPtp was 16.3 (12.1–19.7) mbar, and «safe» Vt (in which ΔPtp <15 mbar) had 46.2% of patients.

**Conclusions:** Monitoring of «Ptr-t» curve and dynamic «Ptr/Vt» loop allow for estimation of the lung injury and WOBp without Pes an Ptp monitoring.

## P72 The effect of respiratory circuit system on development of ventilator associated pneumonia

### E. Ozen, E. Karakoc, A. Ayyildiz, S. Kara, S. Ekemen, B. Buyukkidan Yelken

#### Eskisehir Osmangazi Uni. Faculty of Medicine, Eskisehir, Turkey

**Introduction:** Evaqua technology allows the humidity in the expiratory limb to diffuse through the permeable membrane of the expiratory limb wall before it has an opportunity to condense into liquid water within the circuit limb. As the membrane is not permeable to pathogens it seems to reduce the risk of pathogen transfer to the patient. With conventional circuits, condensate can form inside the expiratory limb when humidity in the expiratory gas flow touches cooler surfaces along the gas path. In this study, we aimed to evaluate the effect of respiratory circuit systems on the development of ventilator associated pneumonia (VAP).

**Methods:** We evaluated 41 patients retrospectively who were admitted to intensive care unit (ICU) and mechanically ventilated using 2 different circuit systems between May, 2013-August, 2015 (Group 1: Adult dual heated wire ventilator circuit, Group 2: Adult ventilator circuit dual heated with Evaqua technology).

**Fig. 32 (abstract P72).**
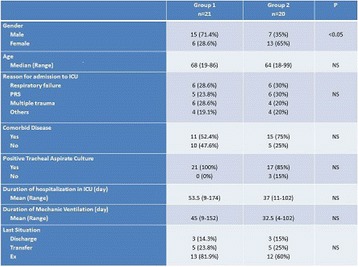
See text for description

**Results:** Patients’ characteristics are showed in Fig. 32. There was statistically difference only in gender status between two groups. Respiratory failure (28.6 vs 30%), Postresuscitation Syndrome (PRS) (23.8 vs 30%) and multiple traumas (28.6 vs 20%) were the main reasons for admission to ICU. Mean duration of hospitalization in ICU and duration of mechanical ventilation were similar in two groups (53.5 (9–174) vs 37 (11–102) days, 45 (9–152) vs 32.5 (4–102) days, respectively). Positive result in tracheal culture was obtained 100% and 85% in Group 1 and 2. Acinetobacter Baumannii, Pseudomonas Aeruginosa and Klebsiella Pneumoniae were the most detected agents in the weekly tracheal culture results. Mean duration time to positive tracheal culture was similar in two groups (10.1 ± 5.9 vs 13.6 ± 9.1 day).

**Conclusions:** We could not find any significant advantage on development of VAP in favor of Evaqua technology. Well-designed prospective studies with more patients are necessary for better understanding of the effect of respiratory circuit system on development of VAP.

## P73 Comparison of minute ventilation to respiratory rate measurements in the post-operative period

### W. Saasouh^1^, J. Freeman^2^, A. Turan^1^

#### ^1^Cleveland Clinic Foundation, Cleveland, OH, USA; ^2^Respiratory Motion, Inc, Waltham, MA, USA

**Introduction:** Opioids are commonly used for postoperative pain management but often decrease respiratory drive and can cause opioid-induced respiratory depression. Current respiratory monitoring in non-intubated patients relies on late indicators of respiratory depression, such as pulse oximetry or point of care measurements of RR [1,2]. Here, we assess the effectiveness of respiratory rate (RR) alone to detect respiratory depression by using a non-invasive respiratory volume monitor (RVM) which accurately measures minute ventilation (MV), tidal volume (TV), and RR [3].

**Methods:** Impedance-based RVM (ExSpiron, Respiratory Motion, Waltham MA) was used to non-invasively collect MV, TV and RR measurements from 104 patients (55 males, BMI: 27.2 ± 5.0 kg/m2) recovering from elective major abdominal surgery. MV, TV and RR were calculated from 30-second respiratory segments for up to 48 hours following surgery. Predicted MV (MVPRED) was calculated for each patient based on body surface area. LowMV was defined as MV < 40% MVPRED and LowRR was defined as RR < 6 breaths/min. RR rate values were compared to MV measurements and sensitivity and specificity of LowRR as a predictor of LowMV were calculated.

**Results:** Patients were monitored for an average of 34.5 ± 14.7 hours in the PACU and general hospital ward. Analysis of all 417,850 paired MV and RR measurements revealed that although MV is a function of RR (MV = TV*RR), there was poor correlation between a given MV measurements and its corresponding RR measurements (r = 0.35). 56.4% of MV recordings were below 80% MVPRED and 19.1% were below 40% MVPRED while only 4.0% were below 6 breaths/min. A variety of RR alarm conditions (4–8 breaths/min (bpm)) were explored which showed that a substantial fraction of low MV measurements remain undetected. Specifically, with a RR cutoff of 8 bpm, 74% of all MV measurements < 40% MVPRED would be missed. Decreasing the RR cutoff to 4 bpm misses 93% of MV measurements <40% MVPRED. Overall, LowRR was a poor predictor of LowMV with a sensitivity of 15.0% and specificity of 98.6%. Furthermore, 29% of all low RR events were associated with adequate MV, indicating that patients were adequately ventilated even in the presence of LowRR.

**Conclusions:** Our data suggest that LowRR alone does not accurately reflect episodes of LowMV, and is not sufficient for accurate assessment of respiratory status. The tidal volume of each breath is at least equally critical to ensure respiratory sufficiency.

**References**

1. British Journal of Anaesthesia, 108:872–875, 2012.

2. Anesthesia & Analgesia, 117:69–75, 2013.

3. Anesthesia & Analgesia, 117:91–100, 2013.

## P74 Ventilatory weaning failure of cardiac origin and the variation of hemoglobin and protidemia

### Z. Hajjej, W. Sellami, M. Bousselmi, W. Samoud, H. Gharsallah, I. Labbene, M. Ferjani

#### Military Hospital of Tunis, Tunis, Tunisia

**Introduction:** Weaning failure of cardiac origin can be diagnosed by the elevation of the left ventricular (LV) filling pressure. This hydrostatic pulmonary edema is associated with hemoconcentration du to hypooncotic fluid movement from the vascular compartiment to the interstitium. The aim of this study was to search for a correlation between the protidemia and the hemoglobin elevation during the weaning test and the presence of LV filling pressure elevation.

**Methods:** This prospective observational study was conducted during two years (between January 2014 and January 2016). Every patient with weaning failure was included. The variation of the biological and ultrasound criteria between the periods before and after the ventilator weaning was analysed.

**Results:** We included 56 patients which failed during the first weaning test. 20 patients (35,7%) had an elevation of the LV filling pressure during the second weaning test with E/A > 0.95 and E/Ea > 8.5 at the end of the test period and within these 20 patients, 12 (60%) failed this second weaning test. When compared to the 36 patients who didn’t present a pulmonary edema, these 12 patients required additional delay for the weaning: 2.5 + 3.7 days versus 0.75 + 2.4 days (P = 0.023). the unique predictive factor associated with the occurrence of pulmonary edema was positive body weight difference between the admission in the ICU and the inclusion in the study (4.6 + 5.6 versus 3.5 + 7.3, p = 0.004). the was no significant variation of the hemoglobin and protidemia during the weaning for the patients who presented a pulmonary edema.

**Conclusions:** During this study we didn’t find a correlation between the hemoglobin or the protidemia variation during the weaning period and the ultrasound criteria of th LV filling pressure elevation. The difference of body weight between the admission in the ICU and the inclusion, which reflect a positive fluid balance, was the unique factor associated with weaning failure of cardiac origin. Its control could allow a more frequent weaning success and the diminution of the morbidity and mortality due to the diminution of the mechanical ventilation duration

## P75 Diaphragm dysfunction in intensive care unit: prevalence and effects of the non invasive ventilation

### L. Vetrugno^1^, F. Barbariol^1^, F. Forfori^2^, I. Regeni^1^, G. Della Rocca^1^

#### ^1^University-Hospital, Udine, Italy; ^2^University-Hospital of Pisa, Pisa Italy

**Introduction:** The prevalence of diaphragmatic dysfunction (DD) in patients admitted to Intensive Care Units (ICUs) has often been underestimated, even though it occurs quite frequently.1,2 The first aim of our study is to estimate its prevalence in patients with acute respiratory failure admitted to our ICU; the second aim is to show if Non Invasive Ventilation (NIV) can be helpful in these court.

**Methods:** We enrolled patients with acute respiratory failure admitted to our ICU, scheduled to perform NIV. To measure the diaphragm excursion, a trans-thoracic ultrasound (US) examination was performed before (T0) and after 30 minutes of NIV (T1).3 NIV was performed through a full-face mask with a pressure support ventilation of 6 to 10 cmH2O and a Positive End Expiratory Pressure (PEEP) of 5 cmH2O. Inspired oxygen fraction was set to obtain an SpO2 > = 92–93%.

**Results:** In our general ICU, the overall prevalence of DD was 55% (11 over 20 pts). A subgroup analysis revealed that 90% (9 over 10 pts) of the post-operative patients had DD, compared to only 20% (2 over 10 pts) of medical origin. All patients with normal diaphragmatic functionality were NIV responder. The sensitivity and specificity of DD in predicting NIV failure was, respectively, 100% and 60%; negative predictive value was 100% while positive predictive value was 46%.

**Conclusions:** The main finding of this study is that DD showed a high prevalence: 55% of our mixed population (surgical and medical). The presence of a normal diaphragmatic functionality seems to be an indicator of good response to NIV.

**References**

1. Kim SH et a. Anesth Analg. 2010; 110(5):1349–54

2. Kim WY et al. Crit Care Med 2011; 39: 2627–30

3. Boussuges A et al. Chest 2009; 135: 391–400

**Fig. 33 (abstract P75).**
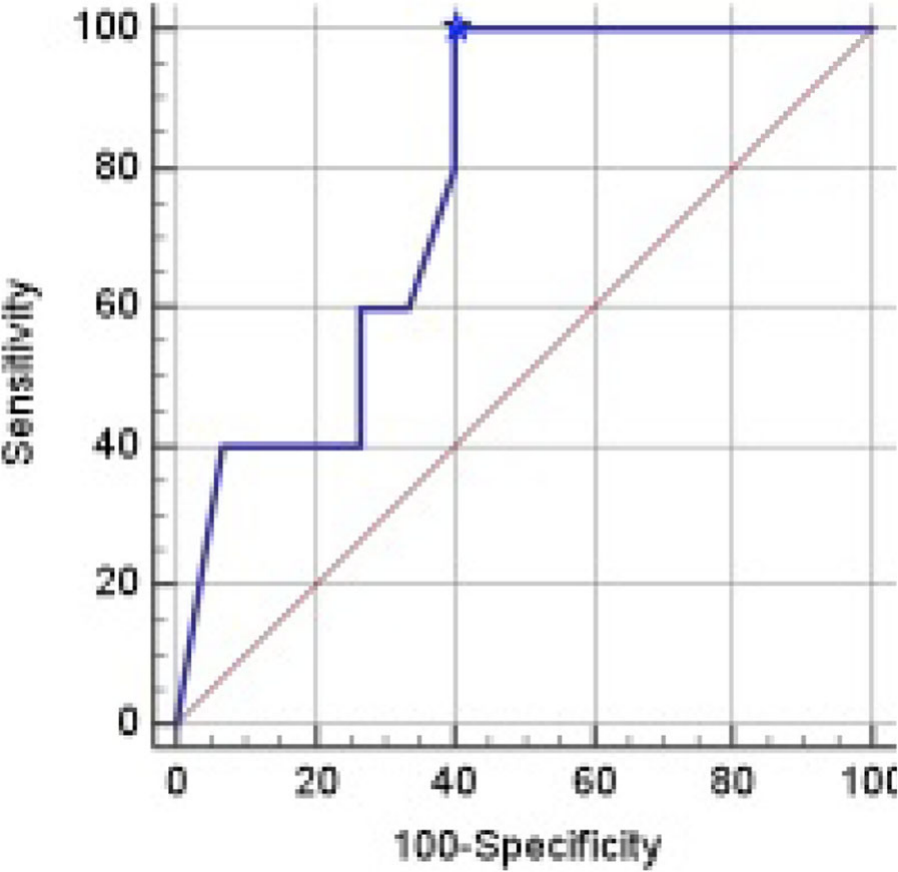
Legend 1: ROC-curve of the diaphragm excursion to predict the non-response to NIV

**Fig. 34 (abstract P75).**
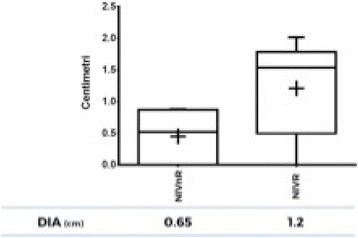
Legend 2: NIVnR and NIVR with the corresponding mean value. P = 0.028

## P76 Reliability of diaphragm neuromuscular efficiency index in mechanically ventilated ICU patients

### D. Jansen^1^, A. Jonkman^2^, J. Doorduin^1^, L. Roesthuis^1^, J. Van der Hoeven^1^, L. Heunks^2^

#### ^1^Radboudumc, Nijmegen, Netherlands; ^2^VU Medical Center, Amsterdam, Netherlands

**Introduction:** Mechanical ventilation unloads the respiratory muscles in order to prevent development of muscle injury and patient discomfort. On the other hand, over-assist may lead to diaphragm inactivity associated with disuse atrophy and patient-ventilator asynchrony. Clinicians are often unable to recognize diaphragm inactivity based on pressure and flow signals on the ventilator. In recent studies an index was described, the neuromuscular efficiency (NME), in which the electrical activity of the diaphragm (EAdi) was used to determine the respiratory muscle effort. However, conversion of EAdi (in microvolt) into pressure has not been very well validated today. The aim of the current study was to assess the repeatability coefficient (RC) of the NME (dynamic and during occlusion).

**Methods:** We included 31 mechanically ventilated adult ICU patients with a dedicated naso-gastric feeding tube for assessing diaphragmatic EMG activity. NMEoccl was calculated by measuring the change in airway pressure (delta Paw) divided by the EAdi during an end expiratory occlusion; repeated 5 times with a 1-minute interval at inclusion and 12, 24, 72 hours respectively. NMEdyn was calculated by dividing the peak inspiratory effort of the diaphragm (Pmus = chestwall compliance + esophageal pressure) by the corresponding EAdi during regular tidal ventilation.

**Results:** The repeatability coefficient of NMEoccl was 81.7%, which means that the difference between two repeated measurements lies between this ratio with a 95% confidence interval. For example, with a calculated NME of 0.93cmH2O/μV and a repeatability coefficient of 81.7% it is expected that 95% of the subsequent measurements will be between 0.17–1.69 cmH2O/μV. Both the Eadi peak as the Paw peak showed a wide variation in which just a moderate correlation was found (p = 0.44). Also after additional analysis, the RC did not improve. The repeatability coefficient of NMEdyn was 125,7%.

**Conclusions:** The measurements of the NME is feasible in daily practice, but the repeatability coefficient of both NMEoccl and NMEdyn seems too high for clinical use.

## P77 Surface electromyography compared to ultrasound for the assessment of diaphragm activity

### S. Arrigoni Marocco^1^, M. Bottiroli^1^, R. Pinciroli^1^, V. Galanti^1^, A. Calini^1^, M. Gagliardone^1^, G. Bellani^2^, R. Fumagalli^1^

#### ^1^Milano Niguarda, Milano, Italy; ^2^Ospedale San Gerardo, Monza, Italy

**Introduction:** A preserved respiratory muscle function is essential for the liberation of patients from mechanical ventilation. Evidence suggests that a prolonged time of ventilation leads to diaphragm atrophy and dysfunction. Bedside diaphragmatic function monitoring may allow for a more complete evaluation of the patient’s respiratory effort. Surface Electromyography (sEMG) is a non-invasive technique for the assessment of diaphragmatic electrical activity.[1] Diaphragm Ultrasound (US) provides a reliable evaluation of its motion.[2] Aim of the present study was to assess, through US, the motility corresponding to sEMG-derived electrical activity signals of the healthy diaphragm.

**Methods:** 12 healthy volunteers underwent a standard breathing protocol including three different respiratory patterns: Quiet Breathing (QB), Deep Breathing and Voluntary Sniffing (VS). Analysis of sEMG recordings was performed to assess baseline and maximum electrical activity (sEMG max, sEMG delta), and the Area Under the Curve (sEMG AUC). Simultaneously we assessed baseline (Tmin) and maximum muscle thickening (Tmax) with a linear 10 Mhz probe positioned at the 9th intercostal space in the mid-axillary line. We calculated Thickening Fraction (TF) as: 100(Tmax-Tmin)/Tmin. We then assessed Diaphragm Excursion (DE) with a convex 7.5 Mhz probe placed below the right costal margin.

**Fig. 35 (abstract P77).**
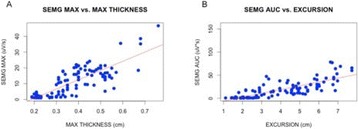
See text for description

**Results:** In healthy volunteers the simulation of different respiratory patterns led to statistically significant differences in terms of both sEMG and US parameters. TF differed among QB (24,8 ± 11,4%), DB (154 ± 40,8%), and VS (96,9 ± 33,3%, p < 0.0001). As well, differences in sEMG delta could be measured (QB: 0.99 ± 0.5 μV; DB: 16.8 ± 9.43 μV; VS: 13.03 ± 5.87 μV, p < 0.0001). A significant correlation could be identified between several parameters obtained by the two techniques. Particularly, sEMG max vs. Tmax (r = 0.81, 95%CI: 0.73–0.87, p < 0.0001, Fig. 35A), and sEMG AUC vs. DE (r = 0.79, 95%CI: 0.71–0.85, p < 0.0001, Fig. 35B).

**Conclusions:** SEMG provides reliable and reproducible information about the electrical activity of diaphragm, showing a direct relationship with the resulting muscle motility, as assessed by ultrasonography.

**References**

[1] Bellani et al. Submitted data

[2] Goligher AJRCCM Nov1;192(9):1080–8,2015

## P78 Evaluation of diaphragmatic thickness with ultrasound and computed tomography in mechanically ventilated patients: a prospective observational study

### S. Gatti^1^, C. Abbruzzese^2^, D. Ippolito^3^, V. L. Sala^4^, V. Meroni^4^, A. Bronco^4^, G. Foti^5^, G. Bellani^5^

#### ^1^University of Milano-Bicocca, Milano, Italy; ^2^Fondazione IRCCS Ca’ Granda Ospedale Maggiore Policlinico, Milano, Italy; ^3^San Gerardo Hospital, Monza, Italy; ^4^Hospital San Gerardo, Monza, Italy; ^5^University of Milano-Bicocca, San Gerardo Hospital, Monza, Italy

**Introduction:** In ICU the dysfunction of respiratory muscles, during mechanical ventilation is a relevant problem which may lead to prolonged ventilation and difficult weaning. Some data suggest that ventilator induced diaphragmatic dysfunction (VIDD) can be aggravated by neuromuscular blockers and high doses corticosteroids. Aims of this study are to evaluate the coherence between ultrasound (US) and computed tomography (CT) measurements of diaphragmatic thickness in mechanically ventilated patients, and their relationship with clinical features and outcomes.

**Methods:** We enrolled intubated or tracheotomized patients undergoing mechanical ventilation for at least 48 hours undergoing chest CT scan for clinical reasons. Patients under the age of 18 years, with neuromuscular diseases, phrenic nerve lesions or air leakage were excluded. In enrolled patients thickness of the right hemidiaphragm was evaluated with US as described by Goligher [1] (within 12 hours prior to or after CT scan), while thickness of right and left diaphragm (anterior, posterior pillars, domes) was evaluated with CT scan. At the time of enrollment clinical data were collected.

**Fig. 36 (abstract P78).**
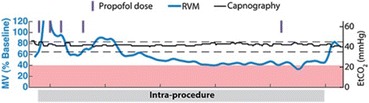
See text for description

**Results:** We enrolled 24 patients in whom 35 CT and US were performed. US evaluation of the right hemidiaphragm was feasible in all patients, and with CT scan in all patient except one. CT measurements of thickness in different portions were tightly related (r = 0,631 ± 0,142). Consistency between US and CT is supported by significant correlations between measurements, in particular posterior diaphragmatic pillars evaluated with CT scan showed the highest correlation coefficient with US, r = 0,439;p-value = 0,009 and r = 0,573;p-value < 0,001 respectively for right and left. Finally we found significant correlations between thickness of posterior pillars and duration of controlled mechanical ventilation (Fig. 36 - r = −0,433;p-value = 0,01), of neuromuscular blocking (r = −0,434;p-value = 0,010) and fraction of total time spent on assisted ventilation on (r = 0,351;p-value = 0,045) before enrollment.

**Conclusions:** Evaluation of diaphragmatic thickness is feasible with both US and CT scan. These techniques may be both useful to evaluate effects of mechanical ventilation and administration of neuromuscular blockers on diaphragmatic structure.

**References**

[1] Goligher et al. Am J Respir Crit Care Med 192(9): 1080–1088, 2015

## P79 Ventilator induced diaphragmatic dysfunction during spontaneous breathing trials assessed by ultrasonography and its impact on weaning outcome

### M. Elbanna, Y. Nassar, A. Abdelmohsen, M. Yahia

#### Cairo University, Giza, Egypt

**Introduction:** Ventilator induced diaphragmatic dysfunction (VIDD leads to difficulties in weaning.

We aimed to evaluate the presence of VIDD during spontaneous breathing trials (SBT) of patients who required mechanical ventilation > =72 hrs and monitor its impact on weaning.

**Methods:** The study was conducted in the Critical Care department Cairo university hospital between march 2014 and march 2015. All consecutive patients who required mechanical ventilation > =72 hrs and were ready for SBT were prospectively recruited. Exclusion criteria: Any history of aminoglycosides, paralyzing agent, central or neuromuscular disease, chemotherapy, cachexia, severe electrolytes imbalance and Intra-abdominal pressure (IAP) > 7

At the start of a 1-hr SBT, each hemidiaphragm was evaluated by M-mode Sonography with the patient in the supine position. Six measurements were recorded and averaged for each side. Ultrasonographic Diaphragmatic Dysfunction (DD) was diagnosed if Diaphragmatic Excursion (DE) was <10 mm or negative paradoxical movement. Patients were classified into two groups: Non Diaphragmatic Dysfunction (NDD Group) and Diaphragmatic Dysfunction (DD Group). Patients were monitored for Rapid shallow breath index (RSBI), Spontaneous minute volume (Spont MV), Airway Occlusion pressure (PO.1), weaning time and outcome.

**Results:** Fifty patients (100%) were studied. DD group included 24 (48%) while NDD group included 26 (52%) patients. There was no statistically significant difference regarding Age, Sex, weight and comorbidities between the two groups (p > 0.05).DE median, was higher [14.4(1.9–40) vs. 9.2 (6.6–35.1), p 0.01] in successfully weaned vs. failed weaning patients.

Successful weaning was higher [18/26(69%) vs.13/24(54.2%), p 0.02)], Weaning time was shorter [29 (11–72) hrs vs 43 (10–192) hrs, p = 0.02], RSBI was lower (58.6 ± 29.9 vs. 72.5 ± 30.5, p = 0.02), PO.1 was lower (1.3 ± 0.8 vs 1.7 ± 0.88, p 0.05) and Spont MV was higher (12.35 L/min ±4.22 vs. 9.45 L/min ±2.28, p 0.017) in NDD Group vs. DD Group respectively.

There was no differences between application of RT. DE <14 mm and the famous RSBI <105 as a cut off in predicting weaning outcome (p > 0.05), ROC was 0.84 of DE < =14 mm and 0.71 of RSBI <105.

**Conclusions:** DD is present in nearly half of our ICU patients on MV > =72 hrs. DE as a morphometric index is as good as traditional volumetric respiratory indices in predicting weaning outcome.

## P80 A combined ultrasound approach to weaning from mechanical ventilation: preliminary results

### S. Mongodi^1^, F. Mojoli^1^, G. Via^1^, G. Tavazzi^1^, F. Fava^1^, M. Pozzi^1^, G. A. Iotti^1^, B. Bouhemad^2^

#### ^1^Fondazione IRCCS Policlinico S. Matteo, University of Pavia, Pavia, Italy; ^2^CHU Dijon, Dijon, France

**Introduction:** Weaning failure (WF) from mechanical ventilation (MV) may be due to lung derecruitment, cardiac dysfunction and respiratory muscles weakness. Transthoracic echocardiography (TTE) [1], lung ultrasound (LUS) [2] and diaphragm ultrasound (DUS) [3] have shown their value in early identification of the failing patients separately. A combination of TTE, LUS and DUS could improve the identification of failing patients and of WF etiology [4]. We aimed to estimate the value of a combined ultrasound assessment (heart-lung-diaphragm) to early identify patients at high risk of WF from MV.

**Methods:** Prospective observational multicenter international study including all patients undergoing a 30’ spontaneous breathing trial (SBT) before extubation. Patients with neuromuscular diseases and MV <48 hours were excluded. TTE and LUS were performed before SBT and at its end; DUS at beginning and end of SBT. TTE included: MAPSE (mitral annulus plane systolic excursion), EF% (ejection fraction), E/A, E/Ea. We computed LUS score (0–36) [2]. DUS assessed diaphragm excursion. Extubation was considered as failed in case of reintubation, non-invasive ventilation or death within 48 hours. We used classical criteria for SBT failure. Extubation was decided by an independent operator.

**Results:** We enrolled 18 patients (age 77.7 ± 11.4 yrs, BMI 29.9 ± 7, SAPSII 54.4 ± 17.4, MV length 9.5 ± 7.3 days). 6 patients (33%) failed the SBT and were not extubated. 12 patients (67%) were extubated; 5 failed. Two populations were identified: WF (failed SBT or extubation) and weaning success (WS). LUS: SBT-LUS was higher than MV-LUS in the whole population (17 ± 4 vs. 12 ± 3; p = 0.019); this was more remarkable in WF (20 ± 3 vs. 11 ± 3; p = 0.0004). SBT-LUS was higher in WF vs. WS (20 ± 3 vs. 13 ± 4; p = 0.03). SBT-LUS predicted SBT and weaning failure (respectively AUC 0,877 and AUC 0,883). TTE: E/A and E/Ea (both MW and SBT) did not predict WF, extubation failure nor SBT-failure. MV-MAPSE predicted WF (AUC 0,833); if < =10 mm it predicted WF with sensitivity 0.67 and specificity 1. SBT-MAPSE predicted extubation failure (AUC 0.833). DUS: No correlation between diaphragm excursion and WF was identified.

**Conclusions:** LUS and MAPSE seem to be the most useful parameters to predict weaning failure.

**References**

1. Caille et al., Crit Care 2010

2. Soummer et al., Crit Care Med 2012

3. Kim et al., CCM 2011

4. Mongodi et al., Crit Care Med 2013

Grant

The research project received the ESICM Clinical Research Award 2015.

## P81 Respiratory work of breathing during CPAP trial on weaning from mechanical ventilation

### F. Ruiz-Ferron^1^, J. Serrano Simón^2^, M. Gordillo-Resina^1^, V. Chica-Saez^1^, M. Ruiz Garcia^1^, R. Vela-Colmenero^1^, M. Redondo-Orts^1^

#### ^1^Complejo hospitalario de Jaen, Jaen, Spain; ^2^Hospital Reina Sofía, Cordoba, Spain

**Introduction:** Tidal volume and respiratory rate are the usual variables to follow the weaning from mechanical ventilation. Although different studies show controversies; because these parameters are influenced by several factors on critically ill patients, and could do not reflect with accurately the work of breathing. Our objective was to study the relationship between respiratory pattern and variables related with the work of breathing.

**Methods:** We studied 11 patients ready to maintain spontaneous breathing after recovering form acute respiratory failure from different causes. Respiratory flow (V’), airways and esophageal pressure (Paw, Pes) were registered for ulterior analysis of these signals. Respiratory rate (RR), delta Pes and pressure time product (PTP) were measured from Pes. The respiratory mechanics during control mechanical ventilation, dynamic elastance (Ers) and total resistances (Rrs), were calculated by multiple linear regression techniques.

**Results:** Age 44 ± 18 y, ventilated since 1 to 70 days (23 ± 22). Ers 26 ± 9 cmH2O/l, Ecw 10 ± 6 cmH2O/l, Rrs13 ± 7 cmH2O/l/s, PEEPi 4 ± 6 cmH2O. On CPAP (5 ± 4 cmH2O), tidal volume 0.350 ± 0.185 l, Inspiratory V’ 0.68 ± 0.16 l/s, RR 24 ± 14 bpm. Variables related with respiratory effort were: PTP 7 ± 4 cmH2O/l•s, PTPm 189 ± 108 cmH2O/l•s/min, delta Pes 10 ± 5 cmH2O. These variables were related with tidal volume (r 0.8;0.5;0.7), however respiratory rate was unrelated (0.05;0.5;0.3).

**Conclusions:** The respiratory effort may not be reflected in the global ventilatory pattern during trial of spontaneous breathing, with risk of respiratory muscle injury and fatigue. Therefore it is necessary to analyze other breathe components by additional monitoring.

## P82 Ultrasound assessment of lung edema correlates with lung injury severity in patients with acute respiratory distress syndrome

### C. Gontijo-Coutinho, T. Ozahata, P. Nocera, D. Franci, T. Santos, M. Carvalho-Filho

#### Unicamp, Campinas, Brazil

**Introduction:** Lung ultrasound (LUS) is useful to assess lung edema in many clinical situations. We hypothesized that LUS might be a good tool to assess gas exchange defect (GED) in patients with the Acute Respiratory Distress Syndrome(ARDS).

**Methods:** This was a prospective observational study at an academic intensive care unit. We Included adult septic patients with ARDS. A simplified lung edema scoring system (SLESS) was used, and 6 thoracic regions were evaluated. Four LUS patterns were considered, from normal aeration to consolidation. To evaluate the GED, the SLESS was compared with the PaO2/fraction of inspired oxygen ratio (PaO2/FiO2) and the partial pressure of carbon dioxide (PCO2).

**Fig. 37 (abstract P82).**
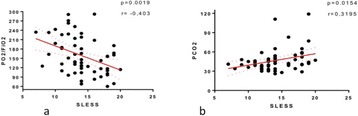
Legend 1: Correlations between SLESS vs. PO2/FiO2 (a) and SLESS vs PCO2 (b).

**Fig. 38 (abstract P82).**
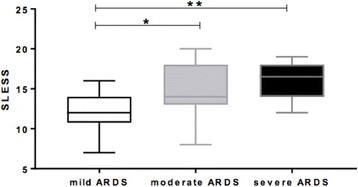
SLESS differences among the severity stages of ARDS

**Results:** Fifty-seven patients were enrolled. Clinical characteristics are presented in Table 11. Figure 37.a shows correlation between SLESS and PaO2/FiO2; Figure 37.b shows the correlation between the SLESS and PCO2 levels. Figure 38 shows a stepwise increase in SLESS among ARDS stages (mean SLESS = 12.21; 14.32 and 16.22, respectively, for mild, moderate and severe ARDS; p < .05 for * and **).

**Conclusions:** The SLESS seems to be useful to assess the gas exchange deffect in patients with ARDS. It also seems to correlate with ARDS severity stages.

**Table 11 (abstract P82).** See text for description

	Survivors (n = 27)	Non-survivors (n = 30)	Total (N = 57)
Age	41.9 ± 16.6	51.3 ± 15.6	46.8 ± 16.6
Female	12	9	21
Male	15	21	36
ARDS severity			
Mild	7	7	14
Moderate	16	18	34
Severe	3	6	9
Clinical data			
Respiratory Rate (bpm)	17.8 ± 5.0	17.7 ± 4.2	17.7 ± 4.5
Mean blood pressure (mmHg)	86.3 ± 10.5	85.3 ± 15.6	85.8 ± 13.3
PCO2	47.3 ± 18.6	46.2 ± 16.3	46.7 ± 17.3
Plasma lactate	1.9 ± 1.5	2.8 ± 3.9	2.4 ± 3.0
Severity scores			
SOFA	8.26 ± 3.31	9.7 ± 3.7	
SAPS3	62.9 ± 13.7	71.97 ± 14.51	

Legend Table 11 Clinical characteristics of study population

## P83 A modified lung ultrasound score to assess aeration in infants: comparison with CT scan

### O. Fochi^1^, S. Gatti^2^, M. Nacoti^1^, D. Signori^2^, A. Bronco^3^, D. Bonacina^1^, G. Bellani^4^, E. Bonanomi^1^

#### ^1^Papa Giovanni XXIII Hospital, Bergamo, Italy; ^2^University of Milano-Bicocca, Milano, Italy; ^3^San Gerardo Hospital, Monza, Italy; ^4^Università degli studi Milano Bicocca, San Gerardo Hospital, Monza, Italy

**Introduction:** In critically ill patients ultrasonography has gained widespread acceptance for lung monitoring of ventilated patients [1]. Lung Ultrasound Score (LUS) has been demonstrated to be a valid tool to monitor semi-quantitatively lung aeration using four patterns corresponding to increasing loss of air content [2]. Computed tomography (CT) scan remains the reference method, nonetheless X-ray exposure is a relevant concern, particularly in children. We developed a modified pediatric lung ultrasound score (pLUS) to assess aeration in infants. Aim of this study was to compare pLUS with CT scan.

**Methods:** Mechanically ventilated children < = 1 years old who underwent CT-scan for clinical purposes received a lung ultrasound examination. Each hemithorax was divided in six regions according a systematic protocol examination [1]. For each explored region, the worst finding was scored as follows: normal: 1, single B-lines: 2; multiple non-coalescent B-lines: 3; coalescent B-lines: 4; white lung: 5; consolidation: 6. A cumulative pLUS was calculated as the sum of each examined region in order to obtain a comprehensive picture of the lung. CT scans were analyzed determining, for each lung region the median density and percentage of aerated lung (fraction of voxels with a density < −500 Hounsfield Units).

**Fig. 39 (abstract P83).**
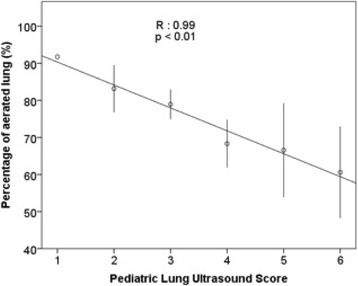
See text for description

**Results:** Nine infants (median age 86 days [36–242]) were enrolled between September 2015 and September 2016 (18 lungs, 108 regions). Regional pLUS and cumulative pLUS of each lung showed a good correlation with median CT-density (R = 0.83; p < 0.05 and R = 0.71; p < 0.01, respectively). Moreover, regional pLUS had a closed correlation with fraction of aerated lung (R = 0.99; p < 0.01) (Fig. 39). Cumulative pLUS showed a significant correlation with fraction of aerated lung (R = 0.54; p < 0.05).

**Conclusions:** According to these preliminary data, pLUS seems to be a reliable method to assess lung aeration in infants showing a close correlation with aeration lung determined by CT scan.

**References**

[1] Bouhemad B, et al. Anesthesiology 122(2):437–47, 2015

[2] Bouhemad B, et al. Am J Respir Crit Care Med. 183(3):341–7, 2011

## P84 Different probes for lung ultrasound – impact on pleural length visualization

### S. Mongodi^1^, E. Bonvecchio^1^, A. Stella^1^, E. Roldi^1^, A. Orlando^1^, M. Luperto^1^, B. Bouhemad^2^, G. A. Iotti^1^, F. Mojoli^1^

#### ^1^Fondazione IRCCS Policlinico S. Matteo, University of Pavia, Pavia, Italy; ^2^CHU Dijon, Dijon, France

**Introduction:** Lung ultrasound (LUS) is a useful tool for lung diseases’ assessment and monitoring [1]. A lung aeration score can be computed on the basis of type and number of visualized artefacts per scan [2]; this allows semiquantification of lung aeration. LUS score is affected by the length of visualized pleura, significantly different in different scans (longitudinal (Long) vs. transversal (Transv)) [3]. The choice of the probe may also affect the length of visualized pleura, and therefore type and number of artefacts useful for lung assessment and LUS score computation. No clear indications on the most appropriate probe are reported in recommendations [4] and different kinds of probe (microconvex-Mi, linear-Li, phased-array-Pa, convex-Co) can be found in literature [5–8], limiting the possibility to standardize the exam. We prospectively compared 4 probes.

**Methods:** Prospective observational monocenter study. In each patient, we scanned 6 standard areas per lung (anterior, lateral, posterior, each divided in superior and inferior) with 4 probes (Pa 2.5 MHz, Co 4 MHz, Li 10 MHz, Mi 10 MHz), in Long (craniocaudal orientation) and Transv (aligned with intercostal space) scan. The length of visualized pleura was measured by a caliper.

**Results:** We enrolled 6 patients (2 males, age 63 ± 23 yrs, BMI 24.7 ± 2.0 kg/m2), corresponding to 72 areas and 288 scans. In all probes, Long has poorer performances than Transv scan: it visualizes significantly shorter pleura with higher pleural length variance (Table 12). In Transv, the length of visualized pleura shows significant differences among different probes. Co visualizes the longest pleura, but also has the highest pleural length variance.

**Conclusions:** Transv visualizes longer and more constant pleura with any probe; it’s confirmed to be a better approach to assess the lung. The length of visualized pleura and its variance differ among probes; linear probe seems to offer the best combination of high visualized pleura and low pleural length variance.

**References**

1. Bouhemad B et al., Anesthesiology 2015,122(2):437–47

2. Bouhemad B et al., Crit Care Medicine 2008;38(1),84–92

3. Mongodi S et al., Eur J Ultrasound 2016; In press

4. Volpicelli et al., Intensive Care Med 2012;38:577–91

5. Soummer A et al., Crit Care Med 2012, 40(7):2064–72

6. Bouhemad B et al., Am J Resp Crit Care Med 2011;1;183(3):341–7

7. Corradi F et al., Chest 2016;150(3):640–51

8. Lichtenstein D et al., Chest 2009;136:1014–1020

**Table 12 (abstract P84).** See text for description

Probe	Linear	Phased-Array	Convex	Micro-convex
Pleural length Transv (cm)	3.68 ± 0.31#	3.21 ± 0.83*	7.18 ± 1.07*	3.97 ± 0.91#
Pleural length Long (cm)	1.69 ± 0.46	2.05 ± 0.48	2.02 ± 0.58	1.74 ± 0.45
Difference in pleural length Long vs. Transv	p < 0.0001	p < 0.0001	p < 0.0001	p < 0.0001
Pleural length variance Transv	0.10	0.68	1.14	0.84
Pleural length variance Long	0.21	0.23	0.34	0.20
Difference of variance Long vs. Transv	P = 0.0025	P < 0.0001	P < 0.0001	P < 0.0001

Legend Table 12*p < 0.0001 vs. all other probes #p < 0.0001 vs. Pa and Co, =0.0187 vs. Li or Mi

## P85 Quantitative assessment of pleural effusion with ultrasound in intensive care unit

### D. Trunfio^1^, G. Licitra^2^, R. Martinelli^2^, D. Vannini^2^, G. Giuliano^2^, L. Vetrugno^3^, F. Forfori^2^

#### ^1^Univesity Hosptial, Pisa, Italy; ^2^Univesity anesthesia and intensive care unit, University of Pisa, PISA, Italy; ^3^Azienda Ospedaliero Universitaria di Udine, Udine, Italy

**Introduction:** Chest ultrasonography is commonly performed in the ICU to identify pleural fluid and to guide thoracic drainage. Aim of this study was to compare the accuracy of two ultrasound methods of estimating the volume of pleural effusions(PEV) with sonography in intubated ICU patients

**Methods:** The first method used to measure PEV was V = 20xSep [1]. Sep was the maximal distance between parietal and visceral pleura, recorded in end-expiration at the posterior axillary line in supine position with trunk elevation at 15°. The compared method multiplied PE paravertebral length (LUS), assessed between the apical and the caudal limits in supine patients, by its cross-sectional area at mid length (AUS) [2]. Moreover the estimated PEV was compared to the volume drained. Patients with incomplete drainage, suspect of empyema, high bleeding risk or interpleural distance less than 10 mm were excluded. *T*-test, Bland-Altman were used to compare the results.

**Fig. 40 (abstract P85).**
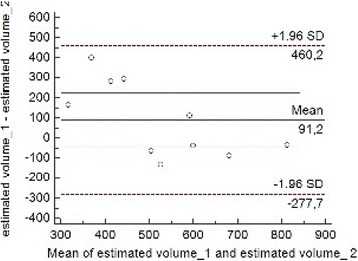
See text for description

**Results:** 10 patients (average age 63,2 ± 12,5) were recruited. Bland-Altman analysis showed no differences between the two methods, which can therefore be regarded as interchangeable (fig. 40). Both ultrasound approaches resulted highly correlated with drained PEV (respectively R0,75 R20,56 P0,012, and R0,92 R20,86 P0,001), and so accurate in estimating it. Coefficients of correlation were totally similar to those reported in previous publications. Ultrasound was performed after a 5-h professional training. These results underlined the simplicity of execution and learning of the ultrasound methods. The time necessary to measure ultrasound parameters was less than 4 min. No patients developed complications after thoracentesis

**Conclusions:** Ultrasound quantification of pleural effusion volume was found to be equally rapid, easy and precise using both methods, and could become a fundamental part of the clinical decision to perform thoracentesis.

**References**

1. Balik, M., et al., Intensive Care Med, 2006. 32(2): p. 318–21.

2. Remerand, F., et al., Intensive Care Med, 2010. 36(4): p. 656–64.

## P86 Accurate and rapid determination of arterial oxygen saturation using photo plethysmography on the sternum

### E. Näslund^1^, L. G. Lindberg^2^, I. Lund^1^, A. Larsson^1^, R. Frithiof^3^

#### ^1^Karolinska Institutet, Stockholm, Sweden; ^2^Linköping University, Linköping, Sweden; ^3^Uppsala University, Uppsala, Sweden

**Introduction:** The aims of this study were to evaluate if:

1) Arterial oxygen saturation can be determined using Photo Plethysmography (PPG) recordings obtained from a probe placed on the sternum.

2) Only the DC-component of the PPG-signal can be used to calculate arterial oxygen saturation.

3) A change in arterial oxygen saturation can be detected faster when monitored centrally over the sternum as compared to other standard non-invasive monitoring sites.

Peripheral vasoconstriction markedly increases the time to detect hypoxia with pulse oximetry [1]. The sternum bone has a central anatomical location and is highly vascularized. Monitoring arterial oxygen saturation in the sternum could circumvent effects of peripheral vasoconstriction and shorten time for detecting hypoxia.

**Methods:** A hypoxia study was conducted with healthy male volunteers (n = 16) that breathed different gas mixtures with gradually decreasing oxygen content to successively lower their arterial oxygen saturation to approximately 60%. A research prototype PPG-probe (RespiHeart) using reflection mode was used to monitor the arterial oxygen saturation in the sternum. Simultaneous sampling of arterial blood gases and recordings of pulse oximetry from an earlobe and a finger were performed. Arterial oxygen saturation from the probe (S[sub]RH[/sub]O[sub]2[/sub]%) was calculated via the quotient of DC-components from infrared (810 nm) and red (660 nm) lights.

**Results:** The observed association between S[sub]RH[/sub]O[sub]2[/sub]% and arterial oxygen saturation measured in blood gases were found to be significant (r^2^ 0.9736, p < 0.05) for every gas mixture used. The sternum probe was in average 28.7 s (95% CI 20.0–37.4 s) faster than the finger probe and 6.6 s (95% CI 5.3–7.8 s) faster than the ear probe to detect desaturation defined as the lowest observed oxygen saturation.

**Conclusions:** Based on the results in the current study we conclude that:

1) It is possible to accurately detect arterial oxygen saturation changes with reflection PPG-recordings based on sternal blood flow on an individual level.

2) Arterial oxygen saturation can be calculated using only the DC-component of the PPG-signal from infrared and red lights.

3) Arterial oxygen saturation changes are detected markedly faster on the sternum compared to more distal sites of measurement.

**References**

1. Mannheimer et al. Minerva Anestesiol 68:236–9,2002

## P87 Comparison of non-invasive monitoring techniques during intravenous propofol-based anesthesia: respiratory volume monitoring vs. capnography

### A. Nichols^1^, J. Freeman^2^, S. Pentakota^1^, B. Kodali^1^

#### ^1^Brigham & Women’s Hospital, Boston, MA, USA; ^2^Respiratory Motion, Inc, Waltham, MA, USA

**Introduction:** Capnography (EtCO2) is the current standard of care for monitoring ventilation in patients under moderate & deep sedation. It assists in making decisions regarding airway interventions. In non-intubated patients, EtCO2 monitoring is often challenging or impossible. Thus, clinicians often rely on pulse oximetry, a late indicator of respiratory depression, or subjective assessments. A recently developed non-invasive respiratory volume monitor (RVM) provides accurate & continuous monitoring of minute ventilation (MV), tidal volume (TV) & respiratory rate (RR) [1–2]. Here we compared RVM & EtCO2 monitoring in patients receiving propofol-based sedation.

**Methods:** Continuous RVM (ExSpiron, Respiratory Motion, Inc.) & capnography data (Capnostream 20, SmartCapnoLine, Covidien) were collected from 17 patients undergoing colonoscopies (6 males; age: 53.1 ± 14.4 yrs; BMI: 27.2 ± 4.9 kg/m2). Baseline MV was established during normal breathing pre-sedation. Monitored anesthesia care was provided using a combination of propofol with midazolam and/or fentanyl. Clinicians were blinded to RVM data. MV, TV, RR, & EtCO2 measurements were compared before & after a cumulative dose of 100 mg of propofol using paired t-tests.

**Fig. 41 (abstract P87).**
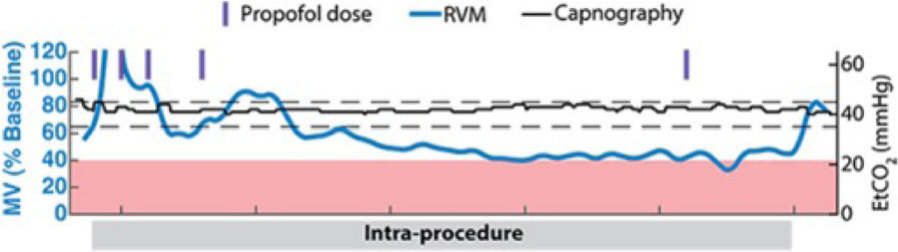
See text for description

**Results:** After administration of propofol, MV & TV decreased significantly (p < 0.05) while EtCO2 remained unchanged (p > 0.5). RR, measured by the RVM, & EtCO2 were similar & not significantly affected by propofol. Figure 41 shows a typical course where MV decreases due to propofol, while EtCO2 shows little change.

**Conclusions:** In non-intubated patients, the RVM reflects expected changes in ventilation with propofol & demonstrates reduction in TV & MV during obstruction & hypoventilation. The RVM provides reliable measurements when capnography is unavailable & is not subject to the EtCO2 limitations of nasal cannula placement, dilution with O2, mouth breathing, & during upper endoscopies. The RVM shows expected decreases in respiratory status in response to anesthetics, while there is little change EtCO2. The RVM provides useful data similar to that provided by capnography & may be a useful alternative.

**References**

1. Voscopoulos C et al.: Anesth Analg 2013; 117: 91–100 2. Voscopoulos C et al.: J Clin Monit Comput 2015; 29: 223–30

## P88 Time evolution of sublingual microcirculatory changes in recreational marathon runners

### A. Pranskunas^1^, I. Kiudulaite^1^, J. Simkiene^1^, D. Damanskyte^1^, Z. Pranskuniene^1^, J. Arstikyte^1^, D. Vaitkaitis^1^, V. Pilvinis^1^, M. Brazaitis^2^

#### ^1^Lithuanian university of health sciences, Kaunas, Lithuania; ^2^Lithuanian Sports University, Kaunas, Lithuania, Kaunas, Lithuania

**Introduction:** Marathon race transiently elevates the probability of sudden death. Also during long-distance run may occur various gastrointestinal symptoms with range from mild nausea to hemorrhagic stool. However microcirculatory nature of this disturbances is not clear. Microcirculation of sublingual mucosa is part of interest, because it is easy and noninvasively accessible, changes have relation with mortality and it is part of the upper digestive tract. Here, we evaluate changes in sublingual microcirculation induced by a marathon race.

**Methods:** Thirteen healthy male controls and 13 male marathon runners volunteered for the study. We performed sublingual microcirculation, using a Cytocam-IDF device (Braedius Medical, Huizen, The Netherlands), and systemic hemodynamic measurements four times on the marathon runners: 24 hours prior to their participation in the Kaunas Marathon (distance: 41.2 km), directly after finishing the marathon, 24 hours after the marathon and one week after the marathon.

**Results:** The marathon runners exhibited a higher functional capillary density (FCD) and total vascular density of small vessels at the first visit compared with the controls. Overall, we did not find any changes in sublingual microcirculation in the marathon runners at any of the visits. However, in a subgroup of marathon runners with a decreased FCD after finishing the marathon race compared to increased FCD had shorter running time (190.37 ± 30.2 vs. 221.80 ± 23.4 min, p = 0.045), ingested less fluids (907 ± 615 vs. 1950 ± 488 ml, p = 0.007) during the race and lost much more weight (−2.4 ± 1.3 vs. -1.0 ± 0.8 kg, p = 0.041).

**Conclusions:** Recreational marathon running is not associated with an alteration of sublingual microcirculation. However, faster running and dehydration may be crucial for further impairing microcirculation.

## P89 Can sublingual microcirculation predict microvascular and tissue responsiveness to usual resuscitation in a porcine model of sepsis?

### R. Pool^1^, H. Haugaa^2^, A. Botero^3^, D. Escobar^4^, D. Maberry^1^, T. Tønnessen^2^, B. Zuckerbraun^1^, M. Pinsky^1^, H. Gomez^1^

#### ^1^University of Pittsburgh, Pittsburgh, PA, USA; ^2^Oslo University Hospital, Oslo, Norway; ^3^Staten Island University Hospital, New York, NY, USA; ^4^Bronx-Lebanon Hospital, New York, NY, USA

**Introduction:** We hypothesized that that sublingual (SL) microcirculatory parameters 1. Can predict microvascular response to resuscitation during sepsis; and 2. Are associated with tissue level lactate, lactate/pyruvate ratio (L/P) and tissue to blood lactate gradient (T-BLac).

**Methods:** Lipopolysaccharide (LPS) was administered to 23 anesthetized Yorkshire-Durock pigs for 45 minutes. Thirteen animals received late (90 min after LPS) and 10 early (immediately after LPS) resuscitation. Five animals per group had available data for this opportunistic study. Sublingual microcirculatory parameters (microvascular flow index (MFI) and perfused vessel density (PVD)) were collected. Tissue level lactate and pyruvate were measured using microdialysis catheters inserted in the liver, kidney and tongue at baseline and pre-/post-resuscitation (PreR, PostR). Resuscitation was driven by MAP and SvO2 as targets and using SVV for fluid responsiveness. Data are shown in median (interquartile range). Non parametric statistics were used whenever appropriate.

**Fig. 42 (abstract P89).**
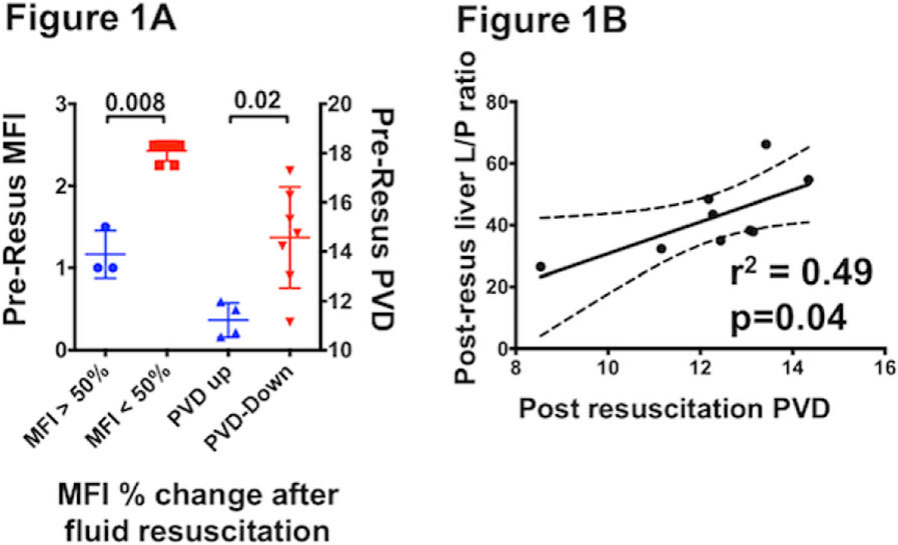
Legend 1: MFI/PVD predict microvascular response (1A) and correlate with liver L/P (1B)

**Results:** Pre-R MFI correlated with Post-R MFI (r2 = 0.562, p = 0.008). Microvascular ‘responsive’ animals (i.e. increase in MFI > 50% or any increase in PVD) had lower Pre-R MFI and PVD (Fig. 42A). Pre-R PVD correlated with Pre-R SL lactate (r2 = 0.4, p = 0.08). A higher Pre-R MFI (>2.5) was associated with lower SLT-BLac (−0.42 (0.74) vs. 3.49 (0.34), p = 0.008). At the Post-R time point, PVD was associated with liver L/P (Fig. 41B), and MFI with renal L/P (r2 = 0.31, p = 0.09). Animals with increased Post-R renal T-BLac had lower Pre-R MFI (1.25 (0.25) vs. 1.75 (0.75), p = 0.07).

**Conclusions:** Pre-resuscitation MFI and PVD may predict post-resuscitation microvascular responsiveness. Importantly, pre- and post-resuscitation MFI and PVD were associated with local metabolic changes in the tongue as well as in vital organs, particularly the kidney and the liver.

## P90 Retrospective study over a 12-month period looking at the national early warning score as a screening tool for patients with sepsis admitted to intensive care

### H. Lyons, A. Trimmings

#### East Sussex Healthcare, East Sussex, UK

**Introduction:** The definition of sepsis has changed recently, pushing us to redefine and examine how we screen for sepsis [1] [2].

The bedside clinical score termed quickSOFA (qSOFA) has been introduced as an additional tool to identify patients with suspected sepsis who are likely to have a prolonged ICU stay or to die in hospital. The trigger score for qSOFA correlates with a NEWS of either 4 or 5 depending on which qSOFA parameters are abnormal. We have recently updated the sepsis screening tool at our institution, amending that published by the UK Sepsis Trust with their permission. We decided to use a NEWS score of 5 as a trigger by local ‘sepsis steering group’ consensus. Whilst reaching this consensus decision, however, we also wanted to look at NEWS for our local population admitted to critical care with sepsis.

**Methods:** Our Trust is a District General Hospital comprising 19 medical and surgical critical care beds across two separate sites. All patients admitted to critical care with a diagnosis of sepsis as per ICNARC coding method from April 2015 until March 2016 were identified using ‘Wardwatcher software’ (Critical Care Audit Ltd, West Yorkshire, UK). This period was before the introduction of the updated sepsis screening tool. We then looked retrospectively at highest NEWS prior to admission to critical care using early warning score electronic records (VitalPac, The Learning clinic, London, UK). Admissions to critical care were from the emergency department, general medical and surgical wards.

**Results:** 200 patients were admitted to critical care with a diagnosis of sepsis during the study period. 47 patients were excluded as NEWS data was not available for these patients. Overall mortality was 34%. 17.6% had a NEWS between 0 and 4. This cohort consisted mainly of young patients with single organ dysfunction who had the lowest mortality (6% of all deaths).

The majority of patients fell into the middle category – 47.8% scored NEWs 5–9 The remaining score a NEWS of greater or equal to 10, this accounted for 34.6% of selected patients. The mortality in this group was high as was the length and complexity of the stay.

**Conclusions:** In our local population NEWS score of 5 is a reasonably sensitive screening trigger for identifying the septic patient at risk of significant deterioration. The patients with a NEWS less than 5 and admitted to ICU with sepsis had a lower morbidity and mortality than those with a NEWS score over 5.

**References**

[1] Singer M et al.; JAMA. 2016;315(8):801–810

[2] Churpek et al.; Am J Respir Crit Care Med. 2016 Sep 20.

## P91 Microcirculatory daily monitoring in polytraumatic patients

### R. Domizi, C. Scorcella, E. Damiani, S. Pierantozzi, S. Tondi, V. Monaldi, A. Carletti, S. Zuccari, E. Adrario, P. Pelaia, A. Donati

#### Università Politecnica delle Marche, Ancona, Italy

**Introduction:** Microvascular alteration is associated with organ dysfunction and adverse outcome in several subsets of critically ill patients. We performed a daily monitoring of sublingual microcirculation to evaluate the association between microcirculatory alterations and the development of organ dysfunction in polytraumatic patients.

**Methods:** This is a subgroup analysis of a perspective observational study on a mixed Intensive Care Unit (ICU) population. 36 polytraumatic patients. Sublingual microcirculation was monitored with Sidestream Dark Field (SDF) imaging daily from admission to discharge/death. Parameters of vessel density and flow quality were calculated with a dedicated software. Organ function was observed and SOFA score was recorded daily.

**Fig. 43 (abstract P91).**
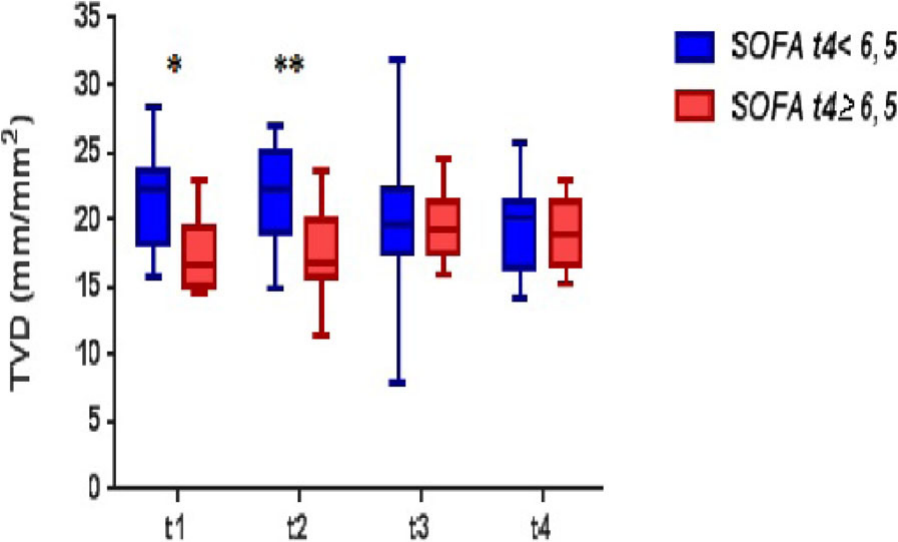
Legend 1: Relationship between TVD and SOFA at day 4

**Fig. 44 (abstract P91).**
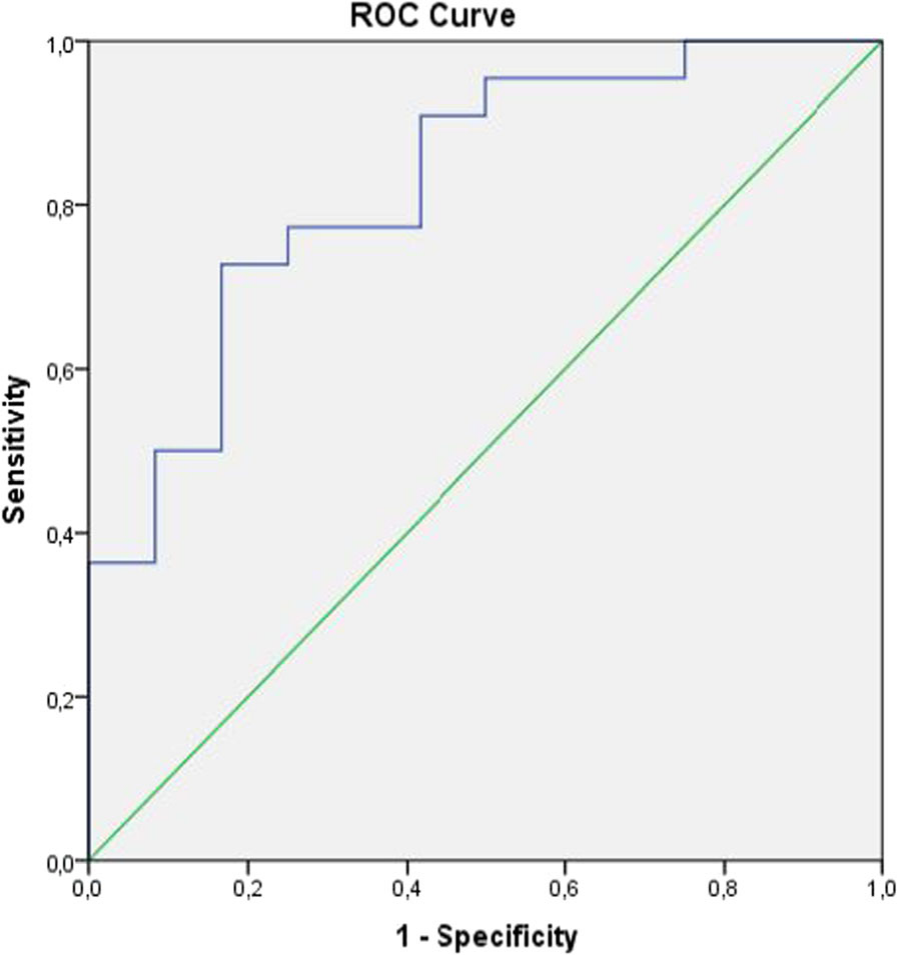
Predictive capacity of TVD day 1 towards SOFA at day 4

**Results:** Patients who had a SOFA ad day 4 < 6.5 showed a higher Total Vessel Density (TVD) at day 1 and 2 (Fig. 43). TVD at day one was also a good predictor of SOFA > = 6.5 at day 4 (Area Under the Receiver Operating Characteristic Curve 0.826; p = 0.002; 95%CI = 0.681–0.970) (Fig. 44). Analogue results were obtained for the Perfused Vessel Density (AUC 0.p99773; p = 0.009; 95%CI = 0.604–0.942). Microvascular Flow Index did not show any predictive value towards development of organ dysfunction.

**Conclusions:** The presence of altered microcirculatory vessel density and perfusion in the first 48 hours from the ICU admission resulted to be associated with the development of organ dysfunction in polytraumatic patients.

## P92 Skin oxygenation and relative hemoglobin concentration measured by hyperspectral imaging based method as prognostic indicators in septic shock

### S. Kazune^1^, A. Grabovskis^2^, K. Volceka^2^, U. Rubins^2^

#### ^1^Hospital of Traumatology and Orthopaedics, Riga, Latvia; ^2^University of Latvia, Riga, Latvia

**Introduction:** Skin mottling is extreme manifestation of microcirculatory changes in septic shock. Skin discolouration is thought to be due to heterogenous changes in oxygen saturation and volume of microcirculatory blood which results in alterations in the amount of reflected light in various parts of the spectrum. These alterations can be captured using hyperspectral imaging. We aim to describe mottled knee skin relative total hemoglobin concentration and microcirculatory saturation values in survivors and non survivors of septic shock and assess their prognostic information.

**Methods:** Hyperspectral imaging was performed in 62 intensive care patients with septic shock enrolled within 24 hours of admission in single centre observational study to obtain relative oxy/deoxyhemoglobin concentration maps and numerical values from mottled area around the knee. Images were processed to identify areas with purple blue discolouration. Nonlinear fitting of optical density spectra was used to calculate relative oxy/deoxy haemoglobin concentration and obtain total hemoglobin concentration (a.u.) and microcirculatory oxygen saturation values (%). In addition, demographic (age, sex, primary site of infection), hemodynamic (mean arterial pressure, dose of vasopressor agents) and disease severity (SAPS II, SOFA score) data were collected.

**Results:** It was possible to obtain analysable images in 55 patients, 26 survivors and 29 non survivors. There was no statistical difference in terms of age (60 (53–73) vs 70 (62.5–82)yrs), gender (42.3 vs 55.2% male), severity of disease (SAPS II 44.5 (32.0–50.0) vs 44.0 (36.0–57.5)) and dose of noradrenaline (0.02 (0–0.09) vs 0.08 (0–0.12 mcg/kg/min) between those who survived the episode of septic shock and those who died. In non survivors relative total hemoglobin concentration in mottled areas was significantly higher (267.6 ± 249.7 a.u. vs 72.3 ± 36.7 a.u.; P < 0.001) and skin microcirculatory oxygen saturation values were significantly lower (29.7 ± 36.1% vs 76.3 ± 30.7%;P < 0.001). Skin microcirculatory oxygen saturation of more than 22% provided 72% specificity and 69% sensitivity for predicting survival. Increase of relative skin total haemoglobin concentration (RR 1.11 (1.05–1.20)) and decrease of skin oxygen saturation by 5 units (RR 1.19 (1.09–1.30)) was associated with increase in hospital mortality.

**Conclusions:** We used a non contact hyperspectral imaging system to quantify total haemoglobin and oxygen saturation in dermal microcirculation of septic shock patients. Increase in total dermal hemoglobin and decrease in oxygen saturation was strongly predictive of 28 day mortality.

## P93 Intra operator variability in visualization of microcirculation in ICU patients using sidestream dark field imaging

### M. Bol, M. Suverein, T. Delnoij, R. Driessen, S. Heines, T. Delhaas, M. Vd Poll, J. Sels

#### MUMC, Netherlands, Netherlands

**Introduction:** There is a growing interest in the state of the microcirculation during critical illness. Sidestream Dark Field Imaging (SDFI) is widely used to study microcirculation in vivo. One such SDFI device is the Glycocheck®, which estimates the thickness of the endothelial glycocalyx by measuring the thickness of the Perfused Boundary Region (PBR). Though single measurements are current clinical practice, intra observer variability is unknown.

**Methods:** Microcirculation was assessed in a pilot study of 49 mechanically ventilated mixed ICU patients using SDFI (Glycocheck®). Each patient was assessed three times consecutively within 30 minutes by the same experienced operator. The operator was unaware of the results of the measurements until all three measurements were completed. The Glycocheck® calculated a PBR for each of the measurements. The intra-observer agreement was calculated using an intra-class correlation coefficient (ICC) using SPSS.

**Results:** The mean (SD) PBR was 2,02 (0,26) μm. The ICC for single measures was 0,362, indicating a poor intraobserver agreement for single measures. ICC for average measures was 0,630 indicating reasonable reliability of the averaged value of 3 consecutive measurements.

**Conclusions:** Intraobserver variability in this pilot study was higher than expected. Averaging the results of three consecutive measurements improved data quality. Whether intraobserver agreement was limited by patient factors such as hemodynamic instability or whether it is inherent to the method itself remains unclear. Reliability of measurement of PBR by SDFI should be increased by averaging (at least three) multiple consecutive measurements. A single measurement is insufficient to yield reliable data on PBR diameter assessed by glycocheck.

## P94 Effect of two levels of mean arterial pressure on microcirculatory reserve in septic shock patients

### M. Jozwiak, M. Chambaz, P. Sentenac, C. Richard, X. Monnet, J. L. Teboul

#### Hôpitaux universitaires Paris-Sud, Hôpital de Bicêtre, Inserm UMR S_999, Univ Paris-Sud, Le Kremlin-Bicêtre, France

**Introduction:** To compare the microcirculatory reserve at two levels of mean arterial pressure (MAP) in septic shock patients.

**Methods:** In 22 septic shock patients receiving norepinephrine with a MAP > 75 mmHg (so-called “high MAP”) within the first six hours of resuscitation, we decreased the norepinephrine dose in order to target MAP to 65–70 mmHg (so-called “low MAP”). We measured muscle tissue oxygen saturation (StO2) at the thenar eminence by using near-infrared spectroscopy and the StO2 recovery slope after a vascular occlusion test at “high MAP” and “low MAP”. Transpulmonary thermodilution cardiac index was also measured at the both MAP levels.

**Results:** On average, MAP was decreased from 81 ± 3 to 67 ± 3 mmHg. Cardiac index did not change (3.26 ± 1.09 vs. 3.12 ± 1.05 L/min/m^2^, respectively). The decrease in MAP was associated with a decrease in mean StO2 recovery slope (3.00 ± 1.40 vs. 2.61 ± 1.46 units/sec, respectively, p < 0.05). StO2 recovery slope decreased by more than 5% in 16 patients and increased by more than 5% in 5 patients. Decreasing MAP from “high” to “low” values was not associated with any changes in mean StO2 (81 ± 8 vs. 81 ± 9%, respectively). StO2 decreased by more than 1% in 11 patients and increased by more than 1% in 8 patients. There was no difference in baseline cardiac index between patients with a decrease or an increase in StO2 or in StO2 recovery slope. Changes in StO2 or in StO2 recovery slope were not different between patients with (n = 17) and without (n = 5) a past medical history of arterial hypertension. There was no correlation between changes in StO2 and changes in StO2 recovery slope.

**Conclusions:** Decreasing MAP from “high” to “low” values in septic shock patients was associated with a decrease in the mean StO2 recovery slope without any changes in mean StO2. Nevertheless, there was a large interindividual variability with two opposite behaviours: the StO2 recovery slope seems to be greater at the high MAP level in the majority of patients but greater at the low MAP level in a smaller group of patients. Thus, it could make sense to monitor the microvascular reserve in septic shock patients in order to individualize the MAP level to target above 65 mmHg.

## P95 B-lines on chest ultrasound predicts elevated left ventricular diastolic pressures

### Z. Bitar^1^, O. Maadarani^2^, R. Al Hamdan^2^

#### ^1^Hôpitaux universitaires Paris-Sud, Hôpital de Bicêtre, Inserm UMR S_999, Univ Paris-Sud, Le Kremlin-Bicêtre, France; ^2^zouheir bitar, Fahahil, Kuwait

**Introduction:** We investigated the relationship between the ultrasonic B profiles and Spectral tissue Doppler echocardiography (E/E’ ratio), a non-invasive surrogate for left ventricular diastolic pressures, in patients presenting with suspicion of acute pulmonary edema

**Methods:** This is a prospective observational study of 61 consecutive patients presenting with acute pulmonary edema and B - profile detected by echocardiography with a 5 MHz curvilinear probe. The Filling pressure of the left ventricle considered high when E/E’ is equal or > 15 or when value between 9 and 14 with ultrasound chest B pattern. The filling pressure is considered normal if E/E’ is equal or below 8 or the value between 9 and 14 with A-line pattern (1).

**Results:** Sixty-one participants were included (49.2% male, with a mean age 66.8). The mean E/E’ level in the patients with B-profile was (20.8), compared with the mean level in the patients with an A-profile of (8.2) (p = 0.003). Based on the value of E/E’, the sensitivity and specificity (including the 95% confidence interval) were determined and are shown in Table 13. The systolic function in the subjects with a B-profile was below 50% in 74.3% of the subjects. All the subjects with B profile and systolic function > 50% had elevated NT-proBNP and E/E’ > 15.

**Conclusions:** Detecting the B-profile in lung ultrasound is highly sensitive and specific for elevated left ventricular diastolic pressures in patients with acute pulmonary oedema.

**References**

Nagueh SF, Appleton CP, Gillebert TC, et al.: Recommendations for the evaluation of left ventricular diastolic function by echocardiography, J Am Soc Echocardiogr 22:107–133, 2009.

**Table 13 (abstract P95).** See text for description

Thoracic ultrasound profile	High E/E’	Normal E/E’	Total
B- Profile	46	1	47
A -profile	4	10	14
total	50	11	61
Variable	Value	95% confidence interval	
sensitivity	0.92	0.812 to 0.968	
Specificity	0.91	0.623 to 0.98	
Positive predictive value	0.97	0.889 to 0.996	
Negative predictive value	0.714	0.454 to 0.883	

Legend Table 13 Chest ultrasound profiles based Spectral tissue Doppler echocardiography E/E’

## P96 Association of extravascular lung water (EVLW) to biometric data: a meta-analysis based on original data by the elwi-star-investigators

### W. Huber^1^, M. Malbrain^2^, M. Chew^3^, J. Mallat^3^, T. Tagami^4^, S. Hundeshagen^1^, S. Wolf^5^

#### ^1^Klinikum rechts der Isar; Technical University of Munich, Munich, Germany; ^2^University Hospital, Ghent, Belgium; ^3^Centre Hospitalier, Lens, France; ^4^Nagayama Hospital, Tokyo, Japan; ^5^Charité, Campus Virchow-Klinikum, Berlin, Germany

**Introduction:** EVLW is a marker of pulmonary oedema associated to morbidity and mortality. To adjust for individual patients, EVLW is indexed to biometric data (EVLWI). Several studies suggest that predicted bodyweight BW_pred is superior to actual bodyweight BW_act for indexation, in particular in obese patients. However, BW_pred is an unspecific formula based on height and gender. BW_act has been introduced to define a gender-adjusted normal weight, but not to specifically adjust haemodynamic parameters.

Therefore, we analyzed the original data of 9 databases of 6 centers regarding the association of EVLW to age, height, gender and BW_act.

**Methods:** Spearman correlation and multivariate regression analysis regarding the association of unindexed EVLW to biometric data. Analyses were performed for all measurements, the first measurements and the individual means Statistics: IBM SPSS 23. ELWI-STAR: EVLW-Study and Research-group

**Results:** 20226 thermodilutions in 1760 adult ICU or peri-operative patients (1049 male and 711 female).

In all measurements, EVLW was univariately associated to male gender (r = 0.264; p < 0.001), height (r = 0.249; p < 0.001), BW_act (r = 0.172;p < 0.001), but not to age (r = −0.013; p = 0.068). In multivariate analysis (R^2^ = 0.047) EVLW was independently associated to male gender (t = 13.919), height (t = 7.169), BW_act (t = 8.471) and age (t = 5.823) with p < 0.001 for all variables.

First measurements of EVLW were univariately associated to male gender (r = 0.261; p < 0.001), height (r = 0.112; p < 0.001), age (r = 0.064; p = 0.009), but not to BW_act (r = 0.034; p = 0.181). In multivariate analysis (R^2^ = 0.054), EVLW was independently associated to male gender (t = 9.205; p < 0.001) and age (t = 2.188; p = 0.029), but neither to height nor to BW_act.

Individual means of EVLW were univariately associated to male gender (r = 0.272; p < 0.001) height (r = 0.146; p < 0.001), BW_act (r = 0.068; p = 0.006), but not to age (r = −0.031; p = 0.193). In multivariate analysis (R^2^ = 0.057) EVLW_mean was only associated to male gender (p < 0.001).

**Conclusions:** EVLW is predominatly associated to height and male gender, whereas BW_act and age have at best limited impact on EVLW. The small amount of R^2^ of about 0.06 suggests that in adult critically ill patients EVLW is mainly driven by pathology (i.e. pulmonary oedema), whereas the impact of biometrics seems to be low, resulting in a high “signal to noise ratio” of EVLW.

## P97 Personalized haemodynamic monitoring: context-sensitive indexation of haemodynamic parameters – a database feasibility analysis

### W. Huber, S. Mair, R. Schmid

#### Klinikum rechts der Isar; Technical University of Munich, Munich, Germany

**Introduction:** Haemodynamic parameters are measured as unindexed “raw values” and in part adjusted to unspecific biometric data. We hypothesized that 1.) Indexation to unspecific biometrics is inappropriate. 2.) Haemodynamic parameters might be associated not only to biometrics, but also to “contexts” such as mechanical ventilation MV, position of the CVC used for transpulmonary thermodilution TPTD and (patho)physiological contexts (e.g. heart rate HR, heart rhythm Rh).

**Methods:** Database analysis (10936 TPTDs; 608 patients). Multivariate regression regarding independent association of GEDV, stroke volume SV, SVV, EVLW and CVP to age A, gender G, weight W, height H, HR, Rh, MV and CVC.

**Results:** 1.) All haemodynamic parameters were independently associated to biometrics and contexts (Table 14).

2.) R^2^-values for the combined models between 0.074 and 0.444 suggest that up 44.4% of the unindexed values can be explained by biometrics and contexts.

**Conclusions:** The ratio of the unindexed value divided by the value expected according to the individual regression-formula (“context-sensitive indexation”) could replace normal ranges, since the individual normal value is 1 (100%).

**Table 14 (abstract P97).** See text for description

	Biometry	R^2^	Context	R^2^	Combined model	R^2^
GEDV	A, G, W, H	0.306	HR, Rh	0.040	A, G, W, H, Rh, CVC	0.345
SV	A, W, H	0.281	HR, Rh, MV	0.194	A, W, H, HR, Rh, MV	0.444
SVV	A	0.014	HR, Rh, MV	0.225	A, W, HR, Rh, MV	0.244
EVLW	A, H	0.039	HR, CVC	0.016	A, H, HR, CVC	0.059
CVP	W, H	0.02	MV	0.039	A, W, H, Rh, MV	0.074

Legend Table 14 Independent association of haemodynamics to biometrics and contexts

## P98 Haemodynamic monitoring – awareness and clinical exposure

### J. Aron^1^, M. Adlam^1^, G. Dua^2^

#### ^1^St Georges Hospital, London, UK; ^2^GSTT, London, UK

**Introduction:** We aimed to run a training day focused on practical use of haemodynamic monitoring (HM). Methods of HM have rapidly expanded, trainees’ knowledge of, and exposure to, all of them is likely to be variable. HM is of increasing importance which has been mirrored with an increasing array of devices. Trainees undergo assessment of their knowledge of these devices during examinations. Practical application however is learnt by apprenticeship. To encourage a wider and more detailed practical understanding we undertook a HM training day.

**Methods:** We conducted a training day consisting of didactic lectures followed by small group practical sessions. We conducted pre and post course surveys assessing subjective confidence of setting up and interpreting data, and objective assessment via multiple choice questions.

**Fig. 45 (abstract P98).**
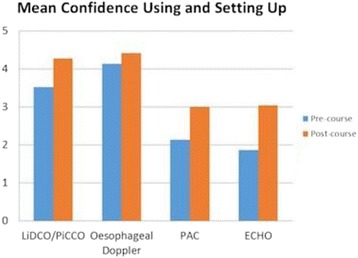
Legend 1: Confidence in setting up and using devices

**Fig. 46 (abstract P98).**
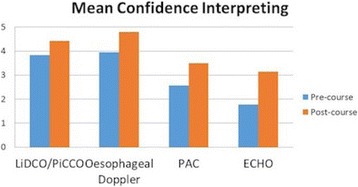
Legend 2: Confidence interpreting devices

**Results:** There was an increase in confidence across all four monitoring techniques in both setting up and interpreting (fig. 45 and 46). The MCQ showed a pre and post course mean of 74% and 78% respectively.

**Conclusions:** The survey has demonstrated inconsistent knowledge of and exposure to the many haemodynamic monitors available. The course has improved both subjective and objective measures. There was a proportionately greater increase in confidence with ECHO and PAC, which may be indicative of the lower overall prior exposure. The MCQ showed minimal increase, and this may reflect the aims of the course were to focus on hands on experience and understanding rather the exam based knowledge.

## P99 Forecasting tachycardia in the ICU

### L. Mu^1^, L. Chen^1^, J. Yoon^2^, G. Clermont^2^, A. Dubrawski^1^

#### ^1^Carnegie Mellon University, Pittsburgh, PA, USA; ^2^University of Pittsburgh, Pittsburgh, PA, USA

**Introduction:** Tachycardia events observed in the Intensive Care Unit (ICU) could lead to cardiorespiratory instabilities and significant morbidity and mortality [1]. We apply machine learning to predict whether and how soon tachycardia will occur based on continuous hemodynamic monitoring data.

**Methods:** An episode of tachycardia is defined as heart rate over 130 for at least 10% of a 5 minute interval. Based on heart rate, respiratory rate, blood pressure and plethysmography from data in MIMIC-II [2], we extracted 42 statistical features over 30-minute time windows right before the onset of tachycardia. We trained two random forest classification models. The first used the first occurrences of tachycardia during patient stay as cases, and randomly selected controls from patients without tachycardia. The second model used the same cases as above but controls were sampled from tachycardia patients data way ahead of its onset.

**Fig. 47 (abstract P99).**
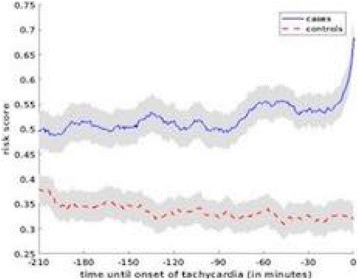
See text for description

**Fig. 48 (abstract P99).**
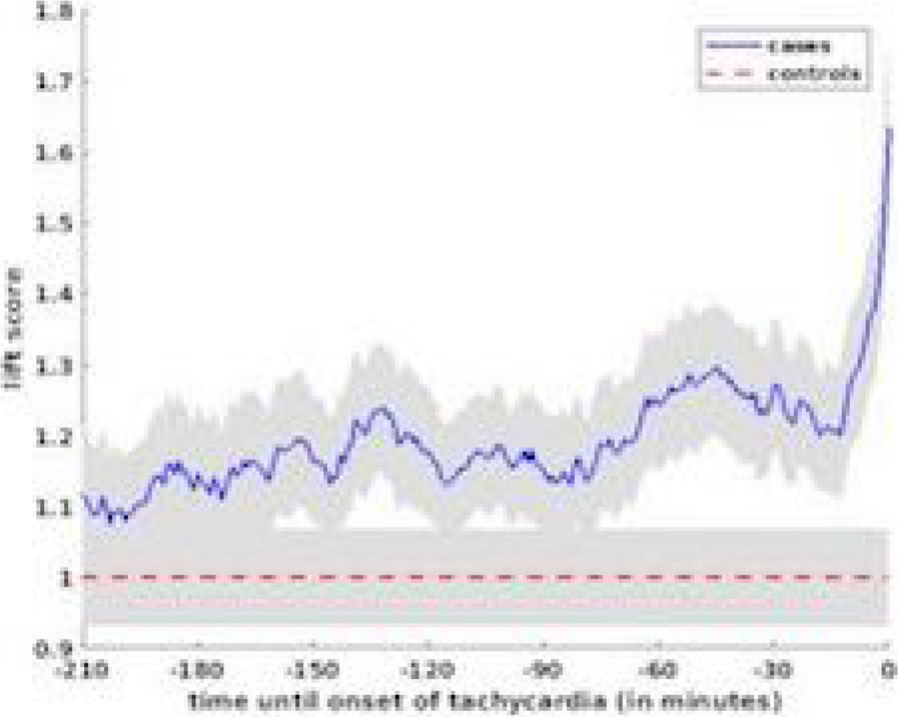
See text for description

**Results:** We evaluated the models using 10-fold cross validation, and tracked predictions made over the entire stays of test patients. Fig. 47 summarizes predicted risk scores and 95% confidence intervals for cases (solid) and controls (dashed) over 3.5 hours leading to tachycardia. Fig. 48 depicts the ratio of risk scores (lift) for cases during 3.5 hours leading to the onset (solid) vs. the risk computed for the same patient during the first few hours of their ICU stay (dashed).

**Conclusions:** Estimated risks scores for cases and controls differ significantly throughout the test period suggesting potential utility of the first proposed model in triage as it helps identify who is going to develop tachycardia with high confidence, and could inform monitoring and care resource allocation in the ICUs. The second model applied to patients at risk reliably signals the upcoming episode more than 1 hour ahead of its onset, allowing preemptive treatment. The results suggest improvements of quality of care and patient outcomes, and mitigation of costs.

**References**

1. Park S et al. Journal of critical care, 2011

2. MIMIC-II. physionet.org/mimic2/

## P100 Qt dispersion in critical illness and relationships with serum lactate

### Z. Duhailib, K. Al Assas, A. Shafquat, N. Salahuddin

#### Zainab Al Duhailib, Riyadh, Saudi Arabia

**Introduction:** Several predictive models and scores were developed to predict critical care disease severity and prognosis. QT dispersion, one of the proposed measures of heterogeneity of ventricular repolarization. It was studied extensively in cardiology and there was a trend toward increased cardiac adverse outcome as well as mortality in normal subjects with a prolonged QTd. We aimed to clarify the relationship between QTd and serum lactate clearance along with ICU mortality and vasopressors requirements in critically ill patients.

**Methods:** A prospective, observational study was performed on inpatients that required a Rapid Response Team (RRT) consultation. 12 lead EKG and serial measurements of physiological and biochemical data were made. QT dispersion was measured as the difference between maximum QT interval and minimum QT interval on the baseline EKG. The study was performed as a nested cohort study within a larger study (Research Ethics Committee approval No. 2151069).

**Results:** Seventy patients were evaluated with a mean age 49.9 ± 22.3 years; mean APACHE II score 23.5 ± 7.3 and a mean SOFA score 9.1 ± 4.9, 64% patients had presumed sepsis. The mean QT dispersion was 68.1 ± 26.5. Significant negative correlations were observed between QT dispersion and mean arterial blood pressure, p =0.03 and serum lactate, p =0.04. QT dispersion was also significantly associated with changes in serum lactate, i.e.: delta lactate at 4 hours, p = 0.04 and at 6 hours, p =0.015. No associations were observed with ICU mortality, 28-day mortality, vasopressor requirement, and QT dispersion.

**Conclusions:** Increased QT dispersion appears to reflect perfusion in patients with critical illness.

## P101 Mortality associated with tachycardia in an intensive care population

### J. Donaghy, P. Morgan

#### East Surrey Hospital, Redhill, UK

**Introduction:** It is recognised that both a prolonged tachycardia and a high daily mean heart rate are associated with an increase in Intensive Care patient mortality [1]. This study aimed to look at the highest recorded heart rates during Intensive Care Unit (ICU) admission and the associated rate of survival to discharge from hospital.

**Methods:** A retrospective analysis looking at a total patient population of 11,685 which included consecutive patients admitted to a mixed medical and surgical ICU over a 22-year period. We assessed the highest heart rate recorded during the first 24 hours of ICU admission. Patients were grouped in incremental sets depending on highest heart rate. The defined outcome was survival to discharge from hospital. Fischer’s exact test was applied to determine two-tailed p values for the dataset.

**Fig. 49 (abstract P101).**
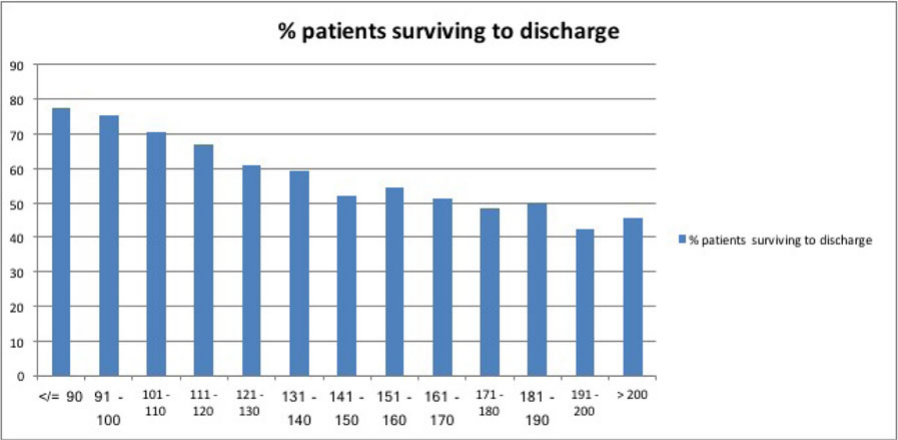
See text for description

**Results:** Of those patients with a highest recorded heart rate of 90 beats per minute (bpm), or less, 77.4% survived to discharge from hospital. This decreased to 70.6% in patients with a high heart rate of between 101 and 110 bpm (p value < 0.0001) and to 50% or less in patients with a high heart rate of more than 170 bpm (p value < 0.0001) (see Fig. 49). The difference in mortality between those with a maximum heart rate of 90 bpm (77.4%) and those with a maximum heart rate of 100 bpm (75.5%) was not statistically significant (p = 0.172).

**Conclusions:** The occurrence of tachycardia of greater than 100 bpm during an ICU admission is associated with a reduced rate of survival to discharge when compared with those patients with a high heart rate of 90 bpm or less.

**References**

1. Park S et al.: J Crit Care. 2011 Oct; 26 (5): 534

## P102 Minimally invasive cardiovascular monitoring is associated with good outcome: results of a multicentre study

### L. Valeanu, M. Stefan, S. Provenchere, D. Longrois

#### Hopital Bichat, Paris, France

**Introduction:** Adult cardiac surgery and perioperative care underwent continuous improvement, with decline in mortality and morbidity. We present data from a prospective, observational study, regarding current clinical practice in cardiac surgery.

**Methods:** With approval from the Ethics and Research Committee, 32 centres in France included cardiac surgery patients from 2 November to 20 December 2015, excluding transcatheter aortic valve replacement, extracorporeal life support, congenital cardiac surgery, wound infections or pericardial drainage.

**Results:** Comparative results in Table 15 present data collected globally and from our centre.

Populations are similar in preoperative risk and comorbidities. Significant differences concern the type of surgery, mean extracorporeal circulation (ECC) and cross clamping (CC) time.

All centres rely on less invasive monitoring, our centre showing remarkable preference for minimally invasive monitoring. While no patient had invasive intraoperative CO monitoring, this was used for 2% of patients in ICU. 36% of patients were monitored intraoperatively by transesophageal echocardiography (TEE), with all patients being monitored by TEE or transthoracic echocardiography (TTE) in the ICU.

All centres used preferentially norepinephrine and dobutamine, while milrinone and levosimendan remain peripheral in clinical practice.

Mortality is low, with no significant differences in postoperative complications or ICU stay.

**Conclusions:** Data shows that in our centre low rates of mortality and morbidity can be maintained using minimally invasive monitoring and relying on echocardiography. If this can be a change of paradigm or an isolated report is to be determined.

**Table 15 (abstract P102).** See text for description

	Bichat number	Total number
Included patients	115 (%)	3111 (%)
Mean Euroscore II	3.89+/−2.36	3.75+/−0.5
Mean ECC/CC time*	67+/−33/53+/−24	97+/−48/70+/−35
Monitoring		
TEE intraoperative/ICU	42 (36)/6 (5)	1139 (36)/250 (8)
TTE ICU*	110 (95)	1327 (42)
CO monitoring intraoperative*/ICU*	0/3 (2.6)	387 (15)/338 (10)
Postoperative complications		
Death*	0	96 (3)
All complications confounded	52 (45)	1338 (43)
ICU stay (days)	4+/−4.8	4.1+/−4.8

*p < 0.05

## P103 Pulmonary artery catheter (PAC) use is associated with improved clinical outcomes after adult cardiac surgery

### A. Shaw^1^, M. G. Mythen^2^, D. Shook^3^, D. Hayashida^4^, X. Zhang^4^, S. H. Munson^4^

#### ^1^Vanderbilt University Medical Center, Nashville, TN, USA; ^2^UCL, London, UK; ^3^Brigham and Womens Hospital, Boston, MA, USA; ^4^Boston Strategic Partners, Boston, MA, USA

**Introduction:** The utility of the PAC has been called into question over recent years as studies have demonstrated varying outcomes in different patient populations. Despite this, sales of PACs have remained robust, suggesting that users of the device derive value from it. To investigate this apparent paradox, we used propensity score methodology to control for observable bias in a retrospective study of adult cardiac surgical patients who were managed with and without a PAC, and whose data were recorded in a North American electronic health record (EHR) database (Cerner Health Facts). The study protocol and analysis plan were approved by the VUMC IRB prior to data abstraction and the study was registered in ClinicalTrials.gov (NCT02964026).

**Methods:** From the Cerner Healthfacts database we identified a study cohort of 6,844 adult cardiac surgery patients: 3,422 PAC patients matched 1:1 with 3,422 non PAC patients via propensity score (PS), developed from patient and hospital demographics, operation, EuroSCORE II, and Elixhauser algorithm. For each patient the APS was calculated on ICU admission and used to control for severity of illness in the outcomes models. Primary outcomes included hospital mortality, Major Adverse Cardiac Events (MACE), Length-of-stay (LOS) and unplanned readmissions. Secondary outcomes included individual organ function outcomes.

**Fig. 50 (abstract P103).**
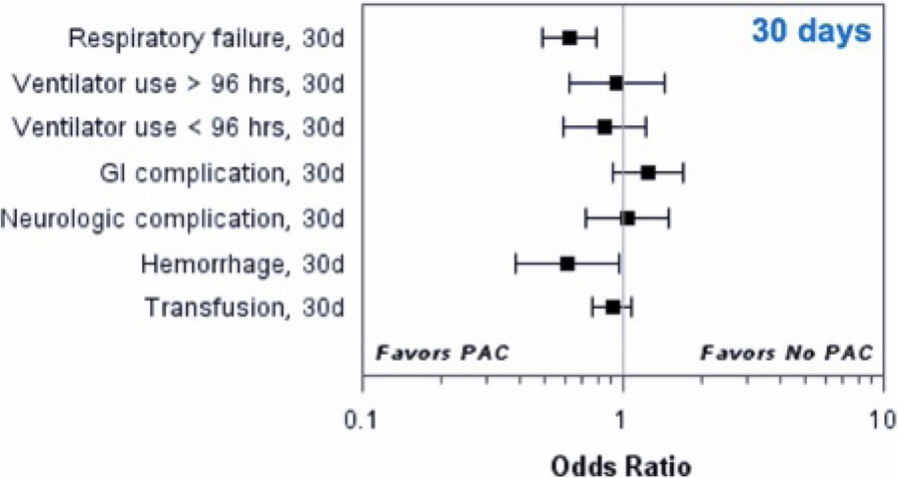
Legend 1: Morbidity Outcomes at 30 days

**Results:** There was no statistically significant difference in the primary endpoint; although 60 and 90 day all-cause readmission rates were significantly lower in PAC patients. Patients who were managed with a PAC experienced significantly fewer complications due to new onset heart failure (index visit OR [95%CI] 0.70 [0.59–0.84], respiratory failure (index visit OR [95%CI] 0.66 [0.53–0.84] and hemorrhage through 30 days post-discharge OR [95%CI] 0.62 [0.39–0.97] (Fig. 50). Patients managed with a PAC experienced some increased infections and abnormal coagulation labs.

**Conclusions:** Use of a PAC in adults undergoing cardiac surgery is associated with fewer cardiopulmonary and bleeding complications, more infections but no increased length of hospital stay, readmissions, or mortality.

## P104 Management of retained guidewires: the ‘suck out technique’

### A. Sawyer, M. Mariyaselvam, M. Blunt, P. Young

#### Queen Elizabeth Hospital, Kings Lynn, UK

**Introduction:** Accidental guidewire retention following central venous catheter (CVC) insertion is the second commonest reported retained foreign object in the UK and is a never event [1]. If recognised early, part of the guidewire may be retained in the catheter lumen. A common technique for guidewire removal is to clamp the catheter at the skin and pull the assembly back. However, if the guidewire has passed beyond the skin level, this is likely to be ineffective. We tested standard techniques against a novel ‘suck out technique’ for guidewire retrieval.

**Methods:** Following institutional approval, a 10x5cm piece of pigskin and subcutaneous tissue (2 cm depth) was cannulated at 45 degrees using the Seldinger technique and a CVC (Blue FlexTip, Arrow, Reading, PA, USA) was placed at a depth of 15 cm to the skin. The J tipped guidewire was intentionally retained, with the straight end at 5 cm above or below the skin level. Minor perpendicular traction was placed on the guidewire, which was in a water trough under a 100 g weighted sponge. Three techniques were assessed to test guidewire retrieval: simple catheter removal, clamping of catheter at skin level and the ‘suck out technique,’ where a 50 ml syringe is attached to the distal lumen and strong suction is continuously applied by retracting the plunger during catheter removal. All three techniques were tested 10 times. A Fishers exact test was used for statistical analysis, with a pre-determined primary end point comparing the two standard with the ‘suck out technique,’ for guidewire retention below the skin.

**Results:** The ‘suck out technique’ was successful in all scenarios (n = 60, p < 0.01).

**Conclusions:** It may be impossible to know the guidewire position, when loss is recognised. Guidewire position may be difficult to see on x-ray, and delays or patient manipulation, may cause further migration. All techniques with the guidewire above the skin were successful, however, as the position is unknown, it is safest to assume the guidewire is below the skin and use the technique most likely to retrieve it in this situation at the earliest opportunity – the ‘suck out technique.’

**References**

1. NHS England National Learning and Reporting Database.

**Table 16 (abstract P104):** See text for description

	Wire end above skin	Wire end below skin
Simple removal	100%	0%
Clamped removal	100%	10%
Suck out removal	100%	90%

Legend Table 16 Success rate of guidewire retrieval techniques

## P105 A comparison of left ventricular systolic function index provided by volumeview/ev1000™ and left ventricular ejection fraction by echocardiography in septic shock patients

### N. Nakwan^1^, B. Khwannimit^1^, P. Checharoen^2^

#### ^1^Division of Critical Care Medicine, Hat Yai, Thailand; ^2^Division of Cardiology, Prince of Songkla University, Hat Yai, Thailand

**Introduction:** The aim of our study was to compare the left ventricular systolic function index provided by VolumeView/EV1000 (Cardiac function index (CFI) and Global ejection fraction (GEF)) with the left ventricular ejection fraction (LVEF) by echocardiography in septic shock patients

**Methods:** A prospective study was conducted in the medical intensive care unit. We simultaneously measured transpulmonary thermodilution and LVEF (biplane Simpson method).

**Fig. 51 (abstract P105):**
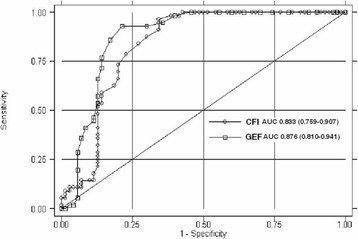
Legend 1: Receiving operating characteristics curves for the prediction of LVEF > 60%

**Results:** A total of 22 septic shock patients were enrolled. There were 126 pairs of systolic function index and LVEF. Mean LVEF was 57.4 ± 17.2%, whereas mean CFI, GEF and cardiac index (CI) were 5.4 ± 2.5 L/min/m2, 21.5 ± 8.2% and 3.4 ± 1.2 L/mim/m2, respectively. CFI was significantly correlated with GEF (r = 0.82, P < 0.001). The area under receiving operating characteristic (AUC) of CFI and GEF were similar for predicting LVEF by more than 35%, 40% and 50% (0.98 vs. 0.99, 0.97 vs. 0.98 and 0.87 vs. 0.88, respectively). However, the AUC of GEF was statistically greater than CFI for predicting LVEF by more than 60% (0.88 vs. 0.83, P = 0.02) Fig. 51. CFI > 3.2 and GEF > 14 estimated LVEF > = 35% with sensitivities of 96.2% and 94.3% and specificities of 95% and 94.5%. Moreover, CFI > 4.4 and GEF > 21 allowed diagnosing an LVEF value > = 60% with sensitivities of 96.4% and 92.9% and specificities of 65.7% and 78.6%.

**Conclusions:** VolumeView/EV1000-derived CFI and GEF provide a reliable estimation of LV systolic function in septic shock patients.

## P106 Methodological issues for the determination of mean systemic filling pressure at the end of life

### D. Berger^1^, P. Moller^2^, S. Bloechlinger^1^, A. Bloch^1^, S. Jakob^1^, J. Takala^1^

#### ^1^Inselspital, Bern University Hospital, University of Bern, Bern, Switzerland; ^2^Institute of Clinical Sciences at the Sahlgrenska Academy, University of Gothenburg, Sahlgrenska University Hospital, Gothenburg, Sweden

**Introduction:** Mean systemic filling pressure (MSFP) is the elastic recoil pressure of the systemic circulation at zero blood flow and it reflects the stressed volume [1, 2]. Clinical estimation models have been developed [1] and values reported from dead ICU patients [2]. The time course of vascular pressure equilibration at the end of life has not been reported.

**Methods:** In eight pigs, HES was infused to expand estimated blood volume (6% body weight) by 15% [1]. Pressures were measured in the aortic arch and both venae cavae (PSVC and PIVC). MSFPRAO was estimated by occlusion of the right atrium with an inflatable balloon and averaged from 9 to 12 seconds into the venous pressure plateau. The animals were then killed with potassium chloride in deep anesthesia without relaxation [1]. Circulatory standstill was defined as loss of pulmonary flow (ultrasonic flow probe). MSFPearly was visually identified at a venous plateau similar to MSFPRAO, MSFPequil at the meeting point of aortic and central venous pressures and MSFPlate after complete cessation of pressure drift. Pressures were averaged over 3 seconds from PIVC and PSVC. Data are average (range) and analyzed by repeated measurements ANOVA.

**Results:** MSFPRAO was 16.4 (11.5–20.9), MSFPearly 13.3 (9.7–16.7), MSFPequil 19.6 (15.7–23.8), MSFPlate 14 (9.6–20.1) mmHg (p = 0.007). The time from standstill to MSFPearly [8.5 (2–19) sec] to MSFPequil [104 (21–91) sec] and MSFPlate [277 (94–506) sec, p < 0.001] was accompanied by an aortic pressure drop of 14.9 (5.3–29.5) mmHg.

**Conclusions:** The venous standstill pressure after death changes dynamically with clinically relevant magnitude over a considerable time. From initial standstill, it increases to an early venous plateau, then further increases to arterio-venous equilibrium, after which all pressures decline together. The equilibrium pressure is not stable. This should be taken into account when MSFP is estimated after cardiac arrest [2].

**References**

1. Berger D et al. Effect of PEEP, blood volume, and inspiratory hold maneuvers on venous return. American journal of physiology Heart and circulatory physiology 2016, 311(3):H794–806.

2. Repesse X et al. Value and determinants of the mean systemic filling pressure in critically ill patients. American journal of physiology Heart and circulatory physiology 2015, 309(5):H1003–1007.

## P107 Critical closing pressure in human endotoxemia and sepsis

### J. M. Van den Brule, R. Stolk, E. Vinke, L. M. Van Loon, P. Pickkers, J. G. Van der Hoeven, M. Kox, C. W. Hoedemaekers

#### Radboud UMC, Nijmegen, Netherlands

**Introduction:** The aim of this study was to determine the change in critical closing pressure (CrCP) during experimental endotoxemia (Lipopolysaccharide, LPS) and sepsis. In addition, we determined the effect of vasopressors on the vasomotor tone during experimental endotoxemia.

**Methods:** We performed a prospective observational study, at the intensive care department (ICU) of a tertiary care university hospital the Netherlands, in 40 healthy subjects during experimental human endotoxemia and in 10 patients with severe sepsis or septic shock.

Subjects in the LPS trial were randomized to receive either a 5 hour infusion of 0,05 μg/kg/min noradrenaline (n = 10, “LPS-nor”), 0,5 μg/kg/min phenylephrine (n = 10, “LPS-phenyl”), 0,04 IU/min vasopressin (n = 10, “LPS-vaso”) or placebo (n = 10, “LPS-placebo”). In patients with sepsis, fluid resuscitation and vasopressor use was performed at the discretion of the medical team, aiming at normovolemia and a mean arterial pressure (MAP) >65 mmHg, using noradrenalin.

The mean flow velocity in the middle cerebral artery (MFVMCA) was measured by transcranial Doppler (TCD) with simultaneously recordeding of heart rate, arterial blood pressure, respiratory rate and oxygen saturation. CrCP was determinded by a cerebrovascular impedance model.

**Results:** The CrCP decreased significantly in the LPS-placebo group from 52.6 [46.6–55.5] mmHg at baseline to 44.1 [41.2–51.3] mmHg at 270 min (P = 0.0281). Infusion of phenylephrine before LPS injection increased the CrCP significantly from 46.9 [38.8–53.4] to 53.8 [52.9–60.2] mmHg (P = 0.0161). Norepinephrine or vasopressin prior to LPS had no effect on the CrCP. The decrease in CrCP after a LPS bolus was similar in all treatment groups. The CrCP in the sepsis patients was 35.7 [34.4–42.0] mmHg and significantly lower compared to values in the LPS-placebo subjects from baseline until 90 min after LPS (P < 0.0001).

**Conclusions:** The human endotoxemia model decreases cerebral blood flow velocity. This is accompanied by a decrease in CrCP, due to a loss of vascular resistance of the arterial bed. Vasopressors could not prevent this change in MFVMCA and did not reverse the decrease in CrCP. Values in patients with sepsis were comparable to those found in subjects after LPS injection.

This means that patients with sepsis, despite treatment with vasopressors, have a risk for low cerebral blood flow and ischemia.

**Fig. 52 (abstract P107):**
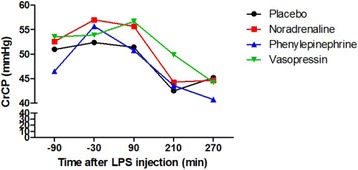
Legend 1: CrCP after LPS injection in LPS-placebo, LPS-nor, LPS-phenyl and LPS-vaso

## P108 Right atrial pressure as back-pressure to venous return

### P. Werner-Moller, S. Jakob, J. Takala, D. Berger

#### Inselspital, University Hospital of Bern, Bern, Switzerland

**Introduction:** Guyton’s graphic analysis integrates vascular and cardiac function. Questions remain whether right atrial pressure (RAP) acts as the back-pressure for venous return (VR) with mean systemic filling pressure (MSFP) as up-stream pressure, and if the model, formulated for steady states, can be applied in dynamic settings [1].

**Methods:** To examine the immediate response of VR (Q[sub]VR[/sub]; sum of caval vein flows) to changes in RAP and pump function we used a porcine closed chest, central cannulation, heart bypass preparation (n = 10) with veno-arterial extracorporeal membrane oxygenation (ECMO). MSFP was determined by clamping ECMO tubing with an opened arterio-venous shunt at [i]Reference[/i], [i]Volume Expansion[/i] (after infusing 9.75 mL/kg of hydroxyethyl starch) and [i]Hypovolemia[/i] (after bleeding 19.5 mL/kg). We analyzed the behavior of RAP and Q[sub]VR[/sub] in maneuvers with variable pump speed at constant airway pressure (P[sub]AW[/sub]) and constant pump speed at variable P[sub]AW[/sub].

**Results:** Data is mean (SD) or range, [i]p[/i]-values reflect one-way repeated measures ANOVA. RAP was inversely proportional to pump speed (linear regression: [i]r[/i]^2^ 0.859–0.999). Q[sub]VR[/sub] was inversely proportional to RAP ([i]r[/i]^2^ 0.594–0.991). Within a volume state, regardless of whether RAP was altered by pump speed or P[sub]AW[/sub], the immediate hemodynamic response operated on a single VR line ([i]r[/i]^2^ 0.594–0.991). Open shunt MSFP (n = 9) was 10.3 (0.5), 12.3 (1.2) and 8.7 (1.0) in [i]Reference, Volume Expansion[/i] and [i]Hypovolemia[/i] ([i]p[/i] <0.0005). Changing RAP via P[sub]AW[/sub] caused immediate, transient, directionally opposite changes in Q[sub]VR[/sub] (n = 9, [i]p[/i] < = 0.002 for RAP, [i]p[/i] < = 0.001 for Q[sub]VR[/sub]), proportional to the change in VR driving pressure as predicted from a stable MSFP.

**Conclusions:** We conclude from these findings that RAP acts as back-pressure to VR and that Guyton’s model can be applied in dynamic conditions.

**References**

1. Brengelmann GL. Letter to the editor: Why persist in the fallacy that mean systemic pressure drives venous return? [i]American Journal of Physiology - Heart and Circulatory Physiology[/i] 311: H1333-H1335, 2016.

## P109 Right ventricular-vascular coupling: a single-beat noninvasive approach in the critically ill

### P. Bertini^1^, F. Guarracino^1^, D. Colosimo^2^, S. Gonnella^1^, G. Brizzi^1^, G. Mancino^1^, R. Baldassarri^1^, M. R. Pinsky^3^

#### ^1^Azienda Ospedaliero Universitaria Pisana, Pisa, Italy; ^2^Meyer Children Hospital, Florence, Italy; ^3^University of Pittsburgh, Pittsburgh, PA, USA

**Introduction:** Right ventricular function (RVF) has been recently reviewed as having a major role in the development of hemodynamic alteration in the critically ill ^1^. Measuring RVF is not easy because of RV geometry and limitations in monitoring techniques, nevertheless, single-beat (SB) approaches to measure RV efficiency and its relationship with the pulmonary vasculature has been described using invasive approaches ^2^ or MRI ^3^

**Methods:** Using max pressure extrapolation from tricuspid regurgitation Doppler flow we designed a completely noninvasive approach to measure RV coupling (RVAC). We aimed to test it in a critically ill ventilated patient. To design end-systolic pressure volume relationship (ESPVR), we defined maximal isovolumic RV pressure (RVPmax) as (dp/dt*tau)/π + CVP as previously described ^4^. We used tricuspid regurgitation flow to assess dp/dt noninvasively. We then calculated mPAP, an approximation of end systolic pressure ^2^, by adding mean tricuspid regurgitation gradient to CVP, and SV using continuity equation as commonly used. Effective pulmonary arterial elastance (RVEa) has been defined as mPAP/SV, right ventricular elastance (RVEes) is the result of the equation (RVPmax-mPAP)/SV ^2^. Their ratio is RVAC. We compared SB invasive technique using a pulmonary artery catheter and completely noninvasive echocardiographic method in a ventilated septic shock patient using 10 seriated measurements modulating afterload condition by changing 4 levels of PEEP (Zeep, 5 cmH20, 10 cmH20, 15cmH20) and 10 others by varying preload conditions using passive leg raising (PLR)

**Results:** New SB noninvasive technique performed well under every load condition except with levels of PEEP above 10

**Conclusions:** In this preliminary first case we describe a new method of measuring RVAC in a completely noninvasive way. We plan further investigations to validate such approach in larger series before assessing the impact of this technique in clinical practice

**References**

1. Pinsky MR The right ventricle: interaction with the pulmonary circulation. Crit Care. 2016 10;20:266.

2. Brimioulle S et al. Single-beat estimation of right ventricular end-systolic pressure-volume relationship Am J Physiol Heart Circ Physiol. 2003;284(5):H1625–30

3. Vanderpool RR et a. RV-pulmonary arterial coupling predicts outcome in patients referred for pulmonary hypertension Heart. 2015;101(1):37–43

4. Abel FL Pmax end systolic elastance and Starling’s law of the heart. Shock 2001;15(1):56–9

## P110 Right ventricular ejection fraction: new non volume-based method

### P. Bertini, S. Gonnella, G. Brizzi, G. Mancino, D. Amitrano, F. Guarracino

#### Azienda Ospedaliero Universitaria Pisana, Pisa, Italy

**Introduction:** Poor right ventricular ejection fraction (RVEF) is an important predictor of mortality in the patient with ischemic cardiomyopathy^1^. Accurate measurement of RVEF foresees precise RV volume assessment as this can be obtained from cardiac MRI. Moreover, RVEF can be estimated using thermodilution (TD) using PAC, but this method can underestimate the correct value under several condition ^2^

**Methods:** Using max pressure extrapolation from tricuspid regurgitation Doppler flow, we measured right ventricular elastance (RVEes) and pulmonary artery elastance (RVEa) so designing a new method to measure RVEF.

To calculate RVEF, we used the previously validated formula ^3^ RVEF = RVEes/(RVEes + RVEa). We then designed end-systolic pressure volume relationship (ESPVR) using maximal isovolumic RV pressure (RVPmax) defined by sine extrapolation of systolic and diastolic portions of RV pressure curve as described ^4^, but the RV pressure curve was assumed from tricuspid regurgitation Doppler flow using the Bernoulli’s. Effective pulmonary artery elastance (RVEa) was then calculated as mPAP/SV and right ventricular elastance (RVEes) as the result of the equation (RVPmax-mPAP)/SV. mPAP can be calculated by adding mean tricuspid regurgitation gradient to CVP, and SV can be measured via continuity equation. We then tested this method against TD and surrogates of RV function as tissue doppler S’ curve and TAPSE at several preload condition using passive leg raising and fluid adminisitration.

**Results:** New single-beat technique was effective in measuring RVEF in every load condition (Delta increase of EF was 11% after PLR, 5% after subsequent fluid challenge). RVEF absolute values were higher than the ones measured by TD, as expected ^2^, but their respective trends correlates well as they are concordant with surrogates measurements.

**Conclusions:** A new method of measuring RVEF independent of RV volumes is here described. Further investigation is needed to assess the real impact of this technique in clinical practice.

**References**

1 Sabe MA et al. Predictors and Prognostic Significance of Right Ventricular Ejection Fraction in Patients With Ischemic Cardiomyopathy. Circulation. 2016 Aug 30;134(9):656–65

2 Starling RC et al. Thermodilution measures of right ventricular ejection fraction and volumes in heart transplant recipients: a comparison with radionuclide angiography. J Heart Lung Transplant. 1992 Nov-Dec;11(6):1140–6

3 Robotham JL et al. Ejection fraction revisited. Anesthesiology. 1991 Jan;74(1):172–83

4 Abel FL. Pmax, end systolic elastance, and Starling’s law of the heart. Shock. 2001 Jan;15(1):56–9

## P111 Treatment of right heart thrombi in pulmonary embolism

### T. Goslar, D. Stajer, P. Radsel

#### University center Ljubljana, Ljubljana, Slovenia

**Introduction:** Mobile right heart thrombi are rare finding in patients with pulmonary embolism, described in up to 4% of cases, but are associated with increased mortality. The optimal treatment strategy is unknown.

**Methods:** We performed a retrospective review of patients charts who were admitted to intensive care unit at University medical center Ljubljana with intermediate or high risk pulmonary embolism between January 1, 2008 and September 20, 2016. 315 patients were screened and in 31 cases right heart thrombus was reported on echocardiographic examination. The primary end point was survival to out of hospital discharge and comparison between different treatment strategies. Data were analyzed using one-way ANOVA statistical test. P of 0,05 was considered to be statistically significant.

**Results:** 31 patients aged 66 ± 15 years, 17 (55%) of whom were male, had right heart thrombus identified on echocardiography. Overall survival at hospital discharge was 65% (20 patients). All the 31 patients received unfractionated heparin (UFH). 9 patients (29%) received only UFH, 17 patients (55%) received thrombolytic therapy in addition to UFH, 2 patients (6%) had thromboaspiration in addition to UFH and 3 patients (10%) had surgical embolectomy in addition to UFH, survival to hospital discharge was 4 (out of 9; 44%), 12 (out of 17; 71%), 1 (out of 2; 50%) and 3 (out of 3; 100%) respectively. There was no statistically significant difference in survival between groups as determined by one-way ANOVA (F = 1,217, p = 0,323).

In three cases the initial treatment strategy was inefficient and they had additional intervention. In two cases thrombolysis did not achieve haemodynamic stabilization so one patient had additional thromboaspiration and the second one had surgical embolectomy. One patient after surgical embolectomy had an additional thromboaspiration. All three patients who had additional interventions survived to hospital discharge.

**Conclusions:** Finding of right heart thrombus in patient with pulmonary embolism is associated with increased mortality. No treatment strategy, UFH alone, UFH + thrombolytic therapy, UHF + thromboaspiration or UHF + surgical embolectomy, was associated with statistically significant survival benefit. It is possible to combine two of treatment strategies if the one attempted first is unsuccessful.

**References**

Stavros V Konstantinides et all.: European Heart Journal 2014; 35: 3033–3080

## P112 Massive pulmonary embolism: a potential lethal complication in patients with autoimmune haemolytic anaemia

### R. De Vos, N. Bussink-van Dijk

#### Academic hospital Maastricht, Maastricht, Netherlands

**Introduction:** Autoimmune haemolytic anaemia (AIHA) is a rare disorder in which red blood cells are destroyed by haemolysis by either IgG or IgM auto-antibodies against red cell membrane antigens. We report a case of a 49-year-old woman who had been diagnosed with AIHA since 2 years. The therapy she received didn’t include any thromboprophylaxis. During her last admission for exacerbation of haemolysis she developed a massive pulmonary embolism (PE) with cardiovascular collapse. In this case the patient died despite intensive care treatment, appropriate cardiopulmonary resuscitation and fibrinolytic therapy (alteplase). Thereafter we performed a literature search to assess the prevalence of venous thromboembolism (VTE) in patients with AIHA.

**Methods:** PubMed was searched for studies that focus on the prevalence of thromboembolic complications in patients with AIHA. The following key words were used: ‘autoimmune haemolytic anaemia’, ‘pulmonary embolism’ and ‘venous thromboembolism’. A total of 32 studies were found. After screening the titles and abstracts only 6 articles were retained. The full text of these articles was analysed.

**Results:** The review of literature showed 3 case reports similar to our case of pulmonary embolism associated with AIHA. Although our search showed a lack of studies concerning this topic, one retrospective review reported an incidence up to 20% of thromboembolic events in patients with AIHA [1]. Two other reviews did not determine an exact percentage but showed that AIHA is an independent risk factor for thromboembolic complications and these patients may need to be considered for thromboprophylaxis [2,3].

**Conclusions:** By reporting this case we want to raise awareness for thromboembolic events in patients with AIHA. VTE and PE are frequent, serious and potential lethal complications in this patient group. Early recognition and treatment of VTE and venous thromboprophylaxis are advised to prevent disastrous outcome. Further studies are needed in order to determine more precisely the prevalence of these complications in the selected patient group.

**References**

1. Lecouffe-Desprets M et al. Autoimmun Rev. 14(11):1023–8, 2015

2. Ramagopalan SV et al. BMC Med. 9(1), 2011

3. Hendrick AM et al. Hematology. 8(1):53–6, 2003

## P113 Cardiac output monitoring: comparison between a non-invasive and a minimally invasive device

### G. Stringari, G. Cogo, A. Devigili, M. Ceola Graziadei, E. Bresadola, P. Lubli, S. Amella, F. Marani, E. Polati, L. Gottin

#### AOUI Verona, Verona, Italy

**Introduction:** Hemodynamic monitoring plays a pivotal role in the management of acutely ill patients. Many monitoring systems are available but they differ from the degree of invasiveness [1]. Aim of this study was to compare a non invasive hemodynamic monitoring system (ClearSight–Edwards Lifesciences) and a minimally invasive system (Vigileo FloTrac–Edwards Lifesciences) in a population of patients undergone coronary revascularization surgery.

**Methods:** This prospective study was conducted in a 12 beds Cardiothoracic Surgical ICU in patients with preoperative normal left ventricular function undergone coronary bypass surgery. Exclusion criteria were age <18 and >79 years, arrhythmias, hemodynamic instability requiring vasopressors or mechanical assistance, heart valves disease. In the immediate postoperative period patients were monitored with Vigileo FloTrac System and ClearSight System in the contralateral arm. The following parameters were monitored every hour till patient discharge and after fluid challenges if needed: Systolic, Diastolic and Mean Arterial Pressures (SP, DP, MAP), Stroke Volume (SV), Stroke Volume Variation (SVV), Cardiac Index (CI). Fluid challenges were performer by the administration of crystalloid solution 500 ml in 20 minutes. Analysis of data was performed with MedCal Software. The agreement between the devices was described by the Bland-Altmann plot.

**Fig. 53 (abstract P113).**
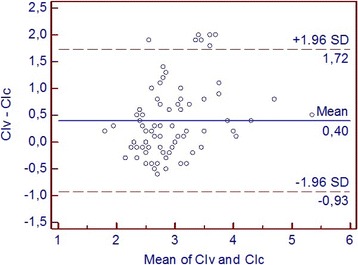
See text for description

**Fig. 54 (abstract P113).**
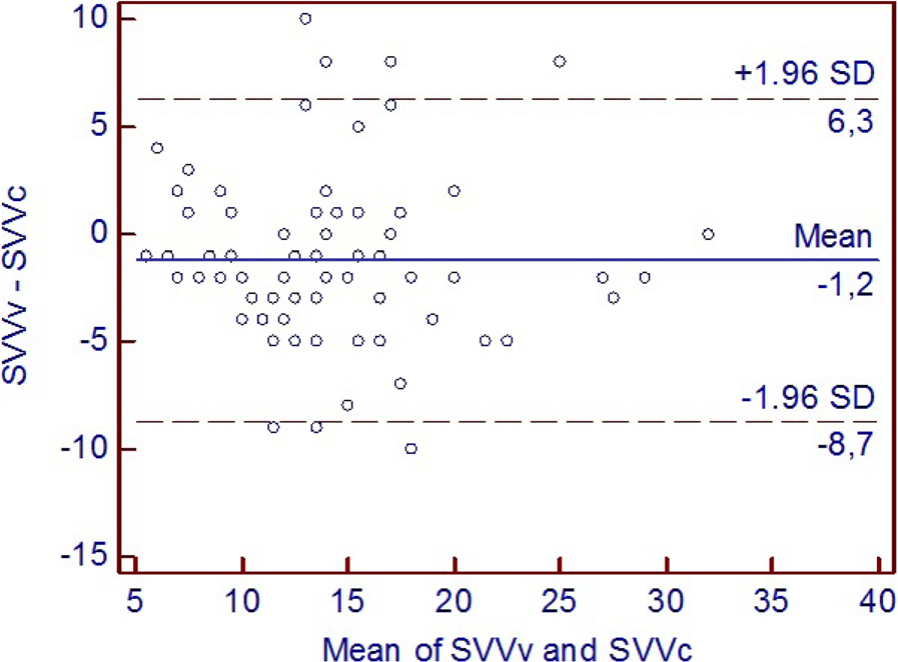
See text for description

**Results:** 15 patients with age ranging from 57 to 76 years were enrolled from June 1st and June 30th 2016. Analysis of the data are reported in Table 17. All the parameters showed a significant difference between the two devices. In fig. 53 and 54: Bland-Altmann plots regarding CI and SVV are reported. All the parameters analyzed and their response to fluid challenge showed agreement even if absolute values were different.

**Conclusions:** The two monitoring systems showed significant agreement. However the mean value of the parameters were significantly different between the two devices.

**References**

1. Vincent JL, et al. Crit Care 2011;15:229

**Table 17 (abstract P113).** See text for description

Parameter	Median	25–75 P	p
CIc	2.7	2.4–3.0	
CIv	3	2.6–3.5	<0.001
DPc	60	54.0–67.0	
DPv	55	48.5–63.0	<0.001
MAPc	72,5	66,0–81,0	
MAPv	76,5	70.0–85.0	<0.001
SPc	96,5	86.0–112.5	
SPv	121	104.5–134.0	<0.001
SVVc	13,5	10.0–17.0	
SVVv	11,5	9.0–16.0	0.0012

## P114 Echocardiographic measurement of cardiac output through modified subcostal window: consistency analysis

### L. Colinas ^1^, G. Hernández^1^, R. Vicho^2^, M. Serna^3^, A. Canabal^1^, R. Cuena^1^

#### ^1^Virgen de la Salud Hospital, Madrid, Spain; ^2^Quironsalud Palmaplanas Hospital, Palma de Mallorca, Spain; ^3^Marina Salud Denia Hospital, Denia, Spain

**Introduction:** Echocardiography has shown to be a feasible and accurate alternative in the measurement of cardiac output (CO).

There are some limitations when using left ventricular outflow tract (LVOT). We can measure the velocity time-integral (VTI) in the right ventricular outflow tract (RVOT).

**Methods:** We included 100 consecutive critically ill patients undergoing transthoracic echocardiography for both initial diagnosis of shock or subsequent hemodynamic monitoring.

LVOT VTI by the apical view and RVOT VTI by the transthoracic view and by the modified subcostal view were measured. Results were analysed with predictive performance test and the consistency analysis. Interobserver reproducibility (ICC) analysis was assessed.

**Results:** The comparison between LVOT VTI and RVOT VTI modified subcostal view revealed a consistency of .52 (95% CI .29 to .69, p < .001); between LVOT VTI and RVOT VTI transthoracic of .44 (95% CI .13 to .67, p = .004). The ICC observed was .92.

**Conclusions:** The consistency between LVOT VTI and RVOT VTI modified subcostal view and RVOT VTI transthoracic view were moderate.

**Fig. 55 (abstract P114):**
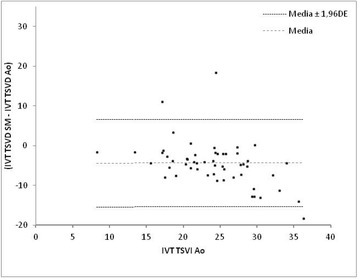
See text for description

**Fig. 56 (abstract P114).**
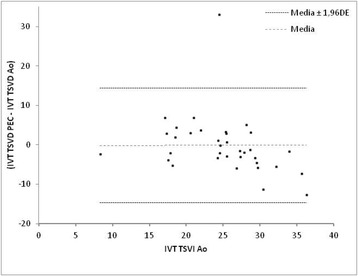
See text for description

## P115 Precision of measurements with transthoracic echocardiography in critically ill patients

### M. Jozwiak, J. Gimenez, J. L. Teboul, P. Mercado, F. Depret, C. Richard, X. Monnet

#### Hôpitaux universitaires Paris-Sud, Hôpital de Bicêtre, Inserm UMR S_999, Univ Paris-Sud, Le Kremlin-Bicêtre, France

**Introduction:** We aimed at answering two questions: how many measurements should be averaged within an echocardiographic examination for measuring the velocity time integral (VTI) of the subaortic flow, in other words what is the within-examination precision? What is the least significant change (LSC) of various variables (VTI, left ventricular ejection fraction (LVEF), E wave of the mitral flow, e’ wave of the external mitral annulus, left ventricular end-diastolic area (LVEDA)) between two measurements performed at different times, either when one or when two operators perform these examinations, in other words what is the between-examination precision?

**Methods:** We included 100 hemodynamically stable patients (age 67 ± 16 y.o., SAPSII 52 ± 19, 54% mechanically ventilated, 16% with atrial fibrillation). Three successive examinations were performed by two different board certified operators, the first and the third examinations by one operator and the second examination by the other operator. Within each examination, we performed three successive measurements for each variable at end-expiration, without moving the probe. For every echocardiographic variable, we calculated the LSC, either from the series of measurements performed within one examination (for the within-examination precision) or from the series of repeated echocardiographic examinations, each one considering the average of three measurements (for the between-examination precision).

**Results:** Regarding within-examination reliability, LSC of VTI was 13 ± 11% when examination included one single measurement. It dropped to 8 ± 6% if three measurements were averaged. Regarding between-examination precision, if two different operators performed the two successive examinations, the LSC of VTI, LVEF, E, e’ and LVEDA was 14 ± 13%, 10 ± 8%, 12 ± 12%, 24 ± 23% and 15 ± 11%, respectively. If the same operator performed the two successive examinations, the LSC for VTI, LVEF, E, e’ and LVEDA was 19 ± 17%, 11 ± 10%, 14 ± 12%, 28 ± 27% and 18 ± 15%, respectively.

**Conclusions:** Within each echocardiographic examination, averaging three measurements is enough for obtaining an acceptable LSC for the VTI measurement. When examinations are repeated, the LSC of echocardiographic measurements is slightly better when the two examinations are performed by the same than by two different operators.

## P116 Withdrawn

## P117 The contribution of (pvco2-paco2)/(cao2-cvo2) ratio level as an indicator of anaerobic metabolism variation in patients with septic shock

### Z. Hajjej, W. Sellami, K. Sassi, H. Gharsallah, I. Labbene, M. Ferjani

#### Military Hospital of Tunis, Tunis, Tunisia

**Introduction:** Blood lactate level in patient with septic shock is dependent on various factors that their interpretation can be difficult. CTA: (PvCO2-PaCO2)/(CaO2-CvO2) superior to 1.4 was described as parameter of anaerobic metabolism’s evaluation. The aim of this study is to describe the correlation between fast hemodynamic status ‘s variation and CTA and if this parameter is related to blood lactate level in patients with septic shock.

**Methods:** We included all patients with septic shock who are intubated, ventilated, sedated, with stable hemodynamic status requiring noradrenalin and with cardiac catheterization. Noradrenalin infusion was reduced in patients who had PAM superior to 85 mmhg under noradrenalin and this to develop a PAM at 65 mmhg. Cardic output was measured with heart ultrasound. Hemoglobin, arterial, venous blood gases were measured before and after noradrenalin modifications.

**Results:** 30 patients with documented septic shock were included. The mean age of patients was 60 yaers olds [20–82]. Blood lactate levels was 3.5 mmol/l(2–8.5) .23 patients had an elevated CTA at 1.4.

The falling of PAM resulted in the rise of CTA from 6% to 130% in 20 cases and the falling of CTA between −40% et −10% in 10 cases. In 26 patients, blood lactate level remained unchanged while 23 patients had a CTA variation from 6% to 130%. The absence of CTA variation is always associated with an absence of lactate level variation. There was no correlation between variation of CTA and variation of cardiac output.

**Conclusions:** Hemodynamic status’s modification in patient with septic shock put under noradrenalin can result in modification of anaerobic metabolism detected with CTA variation and still indetected with lactate level variations.

## P118 Accuracy and precision of ScvO2 measured with the CeVOX-device: a prospectice study in patients with a wide variation of ScvO2-values

### A. Herner, R. Schmid, W. Huber

#### Klinikum rechts der Isar; Technical University of Munich, Munich, Germany

**Introduction:** ScvO2 reflects the relation of oxygen delivery and consumption. Furthermore, ScvO2 has been suggested as therapeutic goal for resuscitation and as a basic parameter of haemodynamic monitoring. Several devices offer continuous monitoring features, including the CeVOX (Pulsion Medical Systems SE, Germany). Despite its use for more than a decade, there are only few studies available that prove validity and clinical usefulness. Some validation studies suggest that accuracy and precision might depend on the value of ScvO2, with lower values resulting in imprecision compared to the gold-standard of blood gas analysis (BGA).

Therefore, we performed a validation study in 24 patients equipped with the CeVOX device which measures ScvO2 using a fibre-optic probe inserted through a conventional CVC. To increase the yield of lower ScvO2-values, 12 of the patients had a femoral CVC.

**Methods:** 24 patients, 21/24 on mechanical ventilation, 9/24 with vasopressors. Baseline cardiac index 4 ± 1.6 L/min/m^2^ (transpulmonary thermodilution with the PiCCO). CVC in the jugular vein (12 patients) or in the femoral vein (12 patients). Baseline BGA and calibration of the CeVOX at 0 h; no further calibrations. Documentation of CeVOX-derived ScvO2 and comparison to simultaneous BGA (without re-calibration) 6 min, 1 h, 4 h, 5 h and 8 h after the initial calibration. Bland-Altman analysis, multiple regression analysis regarding the amount of the bias |ScvO2_CeVOX – ScvO2_BGA|. IBM SPSS 23.

**Results:** n = 120 comparisons. In patients with jugular CVC (primary endpoint), bias, lower and upper limits of agreement (LLOA; ULOA) and percentage error (PE) were acceptable with 0.45%, −12.0%, 12.7% and 15.6%, respectively.

As supposed, ScvO2 was lower in the femoral compared to the jugular measurements (69.5 ± 10.7 vs. 79.4 ± 5.8%; p < 0.001). While the bias (0.68%) was still acceptable, LLOA (−20.5%), ULOA (21.9%) and PE (30.3%) were substantially higher for femoral assessment of ScvO2 by the CeVOX.

Multiple regression analysis demonstrated that the amount of the bias |ScvO2_CeVOX – ScvO2_BGA| independently increased with lower values of ScvO2_BGA and increasing time after calibration, but not with the CVC-site (jugular or femoral), the individual patient or the CI at baseline.

**Conclusions:** Both decreased values of ScvO2_BGA and increasing time to the last calibration increase bias and imprecision of ScvO2_CeVOX. Therefore, in case of low ScvO2_BGA, more frequent re-calibrations of the CeVOX might improve the precision of ScvO2_CeVOX

## P119 Role of veno-arterial co2 gap in patients with sepsis and its correlation with other markers of hyppoperfusion

### N. Abded, Y. Nassar, M. Elghonemi, A. Monir

#### University of Cairo, Cairo, Egypt

**Introduction:** Venous-to-arterial carbon dioxide differences (PCO2 V-A Gap) were reported during shock states. An inverse relationship between PCO2 V-A Gap and cardiac output was described, highlighting the importance of blood flow on venous CO2 accumulation. We performed the study to assess the role of veno-arterial CO2 gap (Pco2 V-A) in septic patients and it’s correlation with other markers of perfusion and hemodynamics.

**Methods:** We studied 50 patients diagnosed with sepsis and/or septic shock.. Arterial and venous blood gas samples were analyzed at different time intervals (time 0,6 hours,12 hours and 24 hours). Venoarterial Co2 gap (PCo2 V-A) was calculated. Routine labs including lactate and base deficited were reported at the same time intervals. Hemodynamic monitoring was done using non-invasive bioimpedence(ICON).

**Results:** Pco2 V-A gap was higher in non survivors versus survivors at all times (basal: 12.2 v 3.3. 6 hours :13.4 vs 1.7, 12 hours: 13.9 vs 1.8, 24 hors :13.9 vs 0.1). cut off value of Pco2 V-A gap for prediction of death was 8 mmhg on admission, 7 mmhg at 6 hours,6.5 mmhg at 12 hours and 6 mmhg at 24 hours. Pco2 V-A gap showed positive correlation with SOFA score at day 3 (r = 0.5), lactate at 24 hours (r = 0.3) and base deficit at 24 hours (r = 0.6). Pco2 V-A gap showed negative correlation with SVR at 24 hours (r = −0.28) and SCvo2 at24 hours (r = −0.5).

**Conclusions:** Pco2 V-A correlated with SVR, lactate. Base deficit, Scvo2 and SOFA score. Pco2 V-A can be used as a prognostic tool in septic patients for prediction of death.

**References**

1-Van Beest PA, van Ingen J and Boerma EC. No agreement of mixed venous and central venous saturation in sepsis, independent of sepsis origin. Crit Care Med 2010, 14:R219.

2-Hernandez G, Pena H, Cornejo R, Rovegno M, Retamal J, Navarro JL, Aranguiz I, Castro R, Bruhn A. Impact of emergency intubation on central venous oxygen saturation in critically ill patients: a multicenter observational study. Crit Care Med 2009, 13:R63.

3-Van Beest PA, Lont MC, Holman ND, Loef B, Kuiper MA and Boerma EC. Central venousarterial pCO2 difference as a tool in resuscitation of septic patients. Intensive Care Med 2013, 39:1034–1039

## P120 Tissue perfusion marker responses to fluid resuscitation? search for an ideal marker

### J. Nikhilesh, T. Apurv

#### CHL Hospitals, Indore, India

**Introduction:** Background:

Sepsis patients form a major chunk of ICU admissions and there have been various guidelines and consensus statements extolling virtues of early fluid resuscitation in these subsets of patients. However, defining an accurate marker in terms of fluid resuscitation targets eludes us till date.

Objective:

To compare responses of tissue perfusion markers to an initial fluid bolus as a component of management of sepsis patients

**Methods:** Setting: A 35 bedded multidisciplinary CCU of a tertiary care unit.

Study Module:

Consecutive patients getting admitted to CCU with sepsis were included. Data was collected with focus on demographics, SOFA scores. All these patients had a baseline measurement of Lactates (L1), Central venous oxygen saturations (Cvo2 1) and base excess (BE 1) values. All were given fluid boluses of 1500 ml of balanced crystalloid and the variables were measured again post fluid resuscitation(L2, CvO2 2, BE2). Patients with severe left ventricular dysfunction, right ventricular dysfunction, known chronic renal failure, history of COPD, hepatic failure (acute/chronic) and out of hospital cardiac arrests were excluded. Statistical analysis was done with a paired *t* test to compare variations in values pre and post resuscitation

**Results:** Thirty nine patients were enrolled (n = 39, M:F-24:15) for a study duration dated March 2016-Nov 2016. SOFA scores were 13.9 + 2.5. Baseline L1, CvO2 1 and BE1 values were 11.6 + 4.1,63.3 + 6.2 and −9.7 + −3.6 respectively. Post resuscitation values for L2, CvO2 2 and BE2 were 9.8 + 3.7, 63.9 + 4.8 and −7.1 + −3 respectively. On statistical analysis for post resuscitation values while Lactate and Base excess had p values of 0.00001(S) central venous oxygen saturations had value of 0.234(NS).

**Conclusions:** While targets for perfusion markers remain variable in terms of end points for fluid resuscitation in sepsis patients our study demonstrated poor correlation of central venous oxygen saturations for variation post fluid resuscitation while lactate levels and Base excess were non inferior. However, this observation requires further validation across various subsets of sepsis to extrapolate on larger cohorts.

## P121 Relationship between initial lactate and the need of ICU-specific interventions in patients with suspected infection presenting to the emergency department

### A. U. Uber, A. Grossestreuer, A. Moskowitz, P. Patel, M. J. Holmberg, M. W. Donnino

#### Beth Israel Deaconess Medical Center, Boston, MA, USA

**Introduction:** The objective of this study was to better characterize the relationship between initial emergency department (ED) lactate and need for ICU-Specific intervention amongst patients with suspected infection. Elevated serum lactate has been shown to be a predictor of mortality in septic populations. To date, however, the relationship between serum lactate and requirement for Intensive Care Unit (ICU)-Specific interventions has not been explored. As need for ICU-Specific intervention is a more proximal and actionable endpoint than all-cause mortality, understanding its relationship to lactate may be useful in assigning patient disposition from the ED.

**Methods:** We performed a retrospective chart review of all patients with a suspected infection presenting to the ED of a single urban tertiary care center between January 2010 to December 2014. Suspected infection was defined as receiving antibiotics and having a culture drawn within 24 hours of ED presentation, consistent with criteria used in the derivation of the Sepsis III definition. ICU interventions were defined as needing intravenous insulin, a central venous catheter, arterial catheter, urgent hemodialysis, pulmonary artery catheterization, intubation, non-invasive ventilation, and/or vasopressors. We excluded those with missing lactate values. The sensitivity and specificity of lactate > 4 for predicting need for ICU-intervention were calculated.

**Results:** Out of the 20,092 patients who met criteria for suspected infection, 48.3% were female and the mean age was 63.8 +/− 17.9. Of the sample, 990 (4.9%) had an initial lactate >4 and 4,309 (21.5%) required an ICU-Specific intervention. Patients with a lactate > 4 had 8 times higher odds of needing an ICU-Specific intervention than those with a lactate < =4 (OR: 8.0 [95% CI: 6.9–9.1]). A lactate > 4 had 15% sensitivity, 98% specificity, 65% positive predictive value, and 81% negative predictive value for requiring an ICU-Specific intervention.

**Conclusions:** In a population of patients with suspected infection, a lactate > 4 was highly specific in predicting need for ICU level care, therefore suggesting the majority of these patients should be admitted to the ICU. This is the first study to use ‘ICU-Specific intervention’ as an endpoint for prediction, which we believe to be a more practical end-point as compared to the traditionally used’all-cause mortality.’

## P122 Agreement between capillary and venous poct lactate in emergency department patients

### CA Graham^1^, K Hung^1^, R Lo^1^, LY Leung^1^, KH Lee^2^, CY Yeung^1^, SY Chan^1^

#### ^1^The Chinese University of Hong Kong, Hong Kong, Hong Kong; ^2^ Prince of Wales Hospital, Hong Kong, Hong Kong

**Introduction:** Increase in lactate levels (hyperlactatemia) is a sensitive marker in early identification of patients who are critically ill. Capillary lactate measurement by handheld lactate devices may allow for rapid determination of test results and extend the possible use in the pre-hospital arena. Thus, the aim of this paper is to study the agreement of handheld lactate analyzers for the measurement of capillary lactate as compared with reference venous blood lactate level assessed using a blood gas analyzer in the Emergency Department (ED).

**Methods:** Two hundred and forty patients triaged as ‘urgent’ (Category 3 of the five category triage scale), aged 18 or above, who presented to the ED in 2016 were recruited. Venous and capillary blood samples were collected for lactate analysis. Venous lactate levels were measured by blood gas analyzer were used as reference (VL-Ref). Capillary lactate level were measured using two handheld analyzers (Nova StatStrip Xpress Lactate Meter and Lactate Scout + Analyzer) (CL-Nova and CL-Scout+). Venous lactate measurements were also performed using two handheld analyzer (VL-Nova and VL-Scout+). Agreement of handheld lactate analyzers with blood gas analyzer will be determined by using Bland-Altman agreement analysis.

**Results:** Two hundred and forty patients (mean age 69.9 years; 54.2% males) were recruited. Of 240 patients, The result of VL-Ref ranged from 0.70–5.38 mmol/L, with a mean of 1.96 mmol/L. 63.75% and 36.25% showed lactate level (VL-Ref) <2 mmol/L and > = 2 mmol/L respectively. Regarding capillary lactate measurements, Bland-Altman agreement method showed bias values of −0.22 mmol/L and 0.46 mmol/L between VL-Ref and CL-Scout + and CL-Nova, with 95% limits of agreement were being −2.17 to 1.73 mmol/L and −1.08 to 2.00 mmol/L respectively. Regarding venous blood lactate level, the results showed bias values of 0.22 mmol/L and 0.83 mmol/L between VL-Ref and VL-Scout + and VL-Nova, with 95% limits of agreement being −0.46 to 0.90 mmol/L and −0.01 to 1.66 mmol/L respectively.

**Conclusions:** Capillary lactate POCT appears to be a promising tool for screening for hyperlactatemia. An overall low systemic bias were observed in CL-Scout + (bias: −0.22 mmol/L) and VL-Scout + (bias: 0.22 mmol/L), suggesting a potential clinical utility of Scout + handheld analyzer. However the wide range of limit of agreement suggests poor precision, and caution the interpretation of results outside of the normal values. Thus, further studies are required to improve the precision of the handheld analyzers.

## P123 The evaluation of peripheral chemoreflex sensitivity in patients with chronic heart failure

### N Trembach, I Zabolotskikh

#### Kuban State Medical University, Krasnodar, Russia

**Introduction:** Assessing the sensitivity of the peripheral chemoreflex (SPCR), we can predict the likelihood of developing respiratory and cardiovascular disorders during the treatment of these patients, during surgery and general anesthesia, to predict the course of the disease and its outcome. At present, there is sufficient evidence that SPCR is often increaseв in chronic heart failure and it is one of the markers of disease progression and even a prognostic marker [1]. However, the existing methods for SPCR assessing are difficult to use in routine clinical practice due to their complexity. Breath-holding test performed well in this regard, in healthy people, and the result of this test is inversely correlated with peripheral receptor sensitivity to carbon dioxide [2]. The aim of the study was to compare the breath-holding test to single-breath carbon dioxide test in the evaluation of the sensitivity of the peripheral chemoreflex in subjects with chronic heart failure.

**Methods:** The study involved 42 patients with stable chronic heart failure due to left ventricular systolic dysfunction (56 ± 12 years). In all participants, breath-holding test was performed in the morning before breakfast: voluntary breath-holding duration was assessed three times, with 10 min intervals. After inspiration of a volume equal to 2/3 of the vital lung capacity, the participant was asked to hold their breath and the duration of voluntary apnea was measured from the beginning of the voluntary inspiration until reflex contractions of the diaphragm were noted by palpation. A mean value of the duration of the three samples was calculated. The single-breath carbon dioxide test [3] was performed the next day. The study was approved by the local ethics committee. All subjects provided signed informed consent to both tests. The reported study was funded by RFBR, research project No. 16-34-60147 mol_a_dk.

**Results:** The average sensitivity of peripheral chemoreflex measured with single-breath carbon dioxide test was 0.31 ± 0.13 L/min/mm Hg., the average breath-holding duration was 41 ± 12 seconds. During the correlation analysis a significant negative correlation between the results of two tests was noted (−0.83, p <0.05).

**Conclusions:** A breath-holding test reflects the sensitivity of the peripheral chemoreflex defined by the single-breath carbon dioxide test in patients with chronic heart failure.

**References**

1. Giannoni A et al., J. Am. Coll. Cardiol. 53: 1975–80, 2009

2. Trembach N et al., Resp. Physiol. And neurobiol. 235: 79–82, 2017

3. Chua TP et al., Eur. J. Clin. Invest. 25: 887–92, 1995

## P124 Cerebral blood flow autoregulation in ischemic heart failure

### J Caldas^1^, R Panerai^2^, L Camara^1^, G Ferreira^1^, J Almeida^1^, G Queiroz de Oliveira^1^, J Jardim^1^, E Bor-Seng-Shu^1^, M Lima^1^, R Nogueira^1^, F Jatene^1^, S Zeferino^1^, F Galas^1^, T Robinson ^2^, LA Hajjar^1^

#### ^1^University of Sao Paulo, Sao Paulo, Brazil; ^2^University of Leicester, Leicester, United Kingdom

**Introduction:** Patients with ischemic heart failure (iHF) have a high risk of neurological complications such as cognitive impairment and stroke. We hypothesized that iHF patients have a higher incidence of impaired dynamic cerebral autoregulation (dCA).

**Methods:** Adult patients with iHF and healthy volunteers were included. Cerebral blood flow velocity (CBFV, transcranial Doppler, middle cerebral artery), end-tidal CO2 (capnography), and arterial blood pressure (Finometer) were continuously recorded supine for five minutes at rest. Autoregulation index (ARI) was estimated from the CBFV step response derived by transfer function analysis using standard template curves.

**Results:** Fifty-two iHF patients and 54 age-, gender-, and BP-matched healthy volunteers were studied. Echocardiogram ejection fraction was 40 (20–45) % in iHF group. iHF patients compared to control subjects had reduced EtCO2 (34.1 ± 3.7 vs. 38.3 ± 4.0 mmHg, p < 0.001) and lower ARI values (5.1 ± 1.6 vs. 5.9 ± 1.0, p = 0.012). ARI < 4, suggestive of impaired CA, was more common in iHF patients (28.8% vs. 7.4%, p = 0.004).

**Conclusions:** IHF patients are more likely to have impaired dCA in comparison with age-matched controls. The relationship between impaired dCA and neurological complications in iHF patients deserves further investigation.

**References**

Panerai RB. Cerebral autoregulation: from models to clinical applications. Cardiovasc. Eng. 8: 1: 42–59, 2008.

de Bruijn RF, Portegies ML, Leening MJ, Bos MJ, Hofman A, van der Lugt A, Niessen WJ, Vernooij MW, Franco OH, Koudstaal PJ and Ikram MA. Subclinical cardiac dysfunction increases the risk of stroke and dementia: the Rotterdam Study. Neurology 84: 8: 833–840, 2015.

**Fig. 57 (abstract P124):**
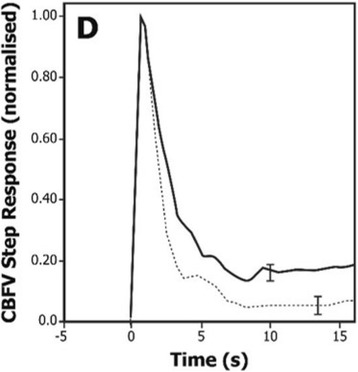
Legend 1: CBFV Step Response

## P125 Cerebral hemodynamic in high-risk cardiac patients undergoing cardiac surgery with cardiopulmonary bypass: the role of intra-aortic balloon

### J Caldas^1^, R Panerai^2^, G Ferreira^1^, L Camara^1^, S Zeferino^1^, J Jardim^1^, E Bor-Seng-Shu^1^, M Oliveira^1^, R Norgueira^1^, R Groehs^1^, L Ferreira-Santos^1^, F Galas^1^, G Oliveira^1^, J Almeida^1^, T Robinson^2^, F Jatene^1^, L Hajjar^1^

#### ^1^University of Sao Paulo, Sao Paulo, Brazil; ^2^University of Leicester, Leicester, United Kingdom

**Introduction:** To assess the effects of intra-aortic balloon pump (IABP) on cerebral hemodynamics in high-risk patients undergoing cardiac surgery with cardio-pulmonary bypass (CPB).

**Methods:** A prospective, randomized, single-center, pilot study was performed in a surgical ICU of a university teaching hospital. Sixty-seven high-risk patients undergoing coronary artery bypass surgery was included, and were randomized in a 1:1 ratio to surgery with or without IABP. Cerebral blood flow velocity (CBFV, transcranial Doppler) and blood pressure (BP, Finometer or intra-arterial line) were continuously recorded over 5 minutes preoperatively (T1), after 24 h (T2) and 7 days after surgery (T3). Autoregulation index (ARI) was estimated from the CBFV response to a step change in BP derived by transfer function analysis. Diagnosis of delirium was based on the Confusion Assessment Method for ICU. Two cognitive scales were applied before and 6 months after surgery.

**Results:** No significant differences were found in the IABP group in comparison with controls for CBFV or ARI. The incidence of delirium or cognitive decline was similar for both groups.

**Conclusions:** IABP does not affect cerebral hemodynamic or rates of post-surgical delirium and cognitive decline after cardiac surgery with CPB. These results suggest that IABP per se is unlikely to contribute to the occurrence of early or late neurological complications of cardiac surgery.

**References**

Panerai RB, White RP, Markus HS, et al.: Grading of cerebral dynamic autoregulation from spontaneous fluctuations in arterial blood pressure. Stroke 1998;29:2341–2346

Tiecks FP, Lam AM, Aaslid R, et al.: Comparison of static and dynamic cerebral autoregulation measurements. Stroke 1995;26:1014–1019

**Fig. 58 (abstract P125):**
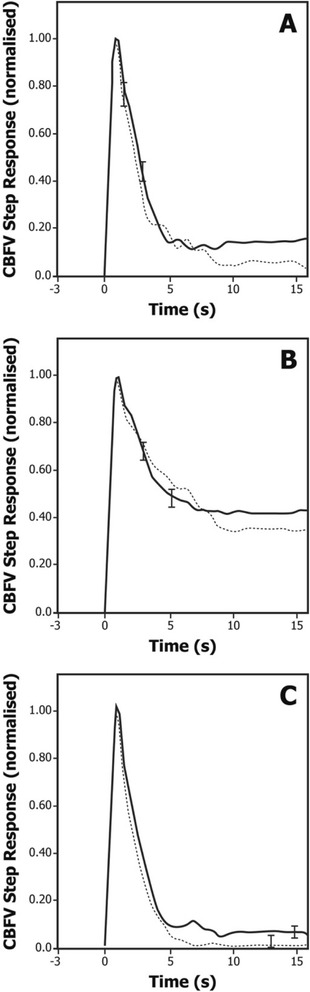
Legend 1: CBFV Step Response in different times and group

**Fig. 59 (abstract P125):**
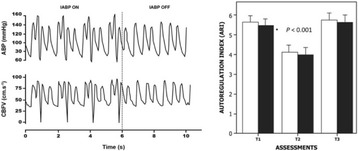
Legend 2: ARI for control group (white) and IABP group (black).10’ continuous recording

## P126 Effect of preoperative intra-aortic balloon pump on outcomes following cardiac surgery in high-risk patients: a prospective randomized clinical trial

### G Ferreira, J Ribeiro, F Galas, F Gaiotto, L Lisboa, J Fukushima, S Rizk, J Almeida, F Jatene, E Osawa, R Franco, R Kalil, L Hajjar

#### University of Sao Paulo, Brazi, Sao Paulo, Brazil

**Introduction:** Intra-aortic balloon pump (IABP) is the most common used device in the setting of cardiac surgery. It improves cardiac output, reduces systemic vascular resistance and decreases afterload. In high-risk patients, the prophylactic use in cardiac surgery might reduce postoperative complications, such as cardiogenic shock and myocardial ischemia. However, in the last years, the evidence regarding the prophylactic use of IABP is controversial, mainly based in retrospective data. This study aims to evaluate the role of prophylactic IABP in reducing complications in high-risk patients undergoing cardiac surgery.

**Methods:** A prospective randomized controlled trial that evaluated 181 patients undergoing coronary artery bypass at the Heart Institute of the University of Sao Paulo from 2014 April to 2016 June to receive or not intraaortic balloon pump after anesthesia induction just before skin incision. Inclusion criteria were left ventricular ejection fraction (LVEF) < = 40% and/or EuroSCORE > = 6. Eligible patients were randomly assigned, in a 1:1 ratio, to IABP group (n = 90) or control group (n = 91). The primary outcome was the composite endpoint of mortality and major morbidity in 30 days after cardiac surgery (cardiogenic shock, stroke, acute renal failure, mediastinitis, prolonged mechanical ventilation and need for reoperation.

**Results:** The primary outcome was observed in 47.8% in the IABP group and 46.2% in the control group (P = 0.456). Patients from the IABP group had a greater duration of inotrope use (51 hours [32–94] vs 39 hours [25–66], P = 0.007) and longer intensive care unit length of stay (five days [3–8] vs four days [3–6], P = 0.035). The length of hospital stay was similar (13 days [9–18] vs 11 days [8–17], P = 0.302) between groups.

**Conclusions:** Preoperative IABP use did not reduce 30-day major complications in high-risk patients undergoing cardiac surgery.

**References**

1. Bignami E, Tritapepe L, Pasin L, Meroni R, Corno L, Testa V, Landoni G, Guarracino F, Zangrillo A: A survey on the use of intra-aortic balloon pump in cardiac surgery. Ann Card Anaesth 2012; 15: 274–7

2. Biondi-Zoccai G, Lotrionte M, Landoni G, Modena MG: The rough guide to systematic reviews and meta-analyses. HSR Proc Intensive Care Cardiovasc Anesth 2011; 3: 161–73

3. Boning A, Buschbeck S, Roth P, Scheibelhut C, Bodeker RH, Bruck M, Niemann B: IABP before cardiac surgery: clinical benefit compared to intraoperative implantation. Perfusion 2013; 28: 103–8

4. Christenson JT, Badel P, Simonet F, Schmuziger M: Preoperative intraaortic balloon pump enhances cardiac performance and improves the outcome of redo CABG. Ann Thorac Surg 1997; 64: 1237–44

5. Christenson JT, Licker M, Kalangos A: The role of intra-aortic counterpulsation in high-risk OPCAB surgery: a prospective randomized study. J Card Surg 2003; 18: 286–94

## P127 Clinical evaluation of the volemic status in critically ill non-septic cardiac patients- the role of central venous pressure

### M Chlabicz, B Sobkowicz, K Kaminski, R Kazimierczyk, W Musial, A Tycińska

#### Medical University of Bialystok, Bialystok, Poland

**Introduction:** Critically ill cardiac patients consist a heterogeneous population, but usually are congestive. Among the variety of methods used to evaluate volemic status in Intensive Cardiac Care Unit (ICCU) none of them has been checked in a prospective study.

**Methods:** Twenty-two consecutive non-septic critically ill cardiac patients (mean age 67.1 ± 14.8 years, mean APACHE II 14.6 ± 5.6 points) were prospectively evaluated. Among them 11 (50%) were admitted due to non-ischemic acute heart failure (AHF), 6 (27%) with ischemic AHF, 3 (14%) with acutely decompensated pulmonary hypertension and 2 (9%) because of cardiac tamponade. At admission clinical as well as routine biochemical assessments were performed, CVP was measured and transthoracic echocardiography (TTE) was performed with the measurements of right atrium area (RAA), tricuspid regurgitation peak gradient (TRPG), tricuspid annular plane systolic excursion (TAPSE) and left ventricle ejection fraction (LVEF). Moreover, vena cava inferior (VCI) during inspiration (VCIi) and expiration (VCIe) was measured.

**Results:** During mean 6 ± 3 days of hospitalization 7 (32%) patients died. Admission CVP was 12.1 ± 5.9 mmHg and not significantly lower values in the group of deaths as compared to survivors were found (11.0 ± 4.8 vs. 12.6 ± 6.5 mmHg, p = 0.6). Among patients who died significantly lower central venous oxygen saturation, higher high-sensitivity troponin I, higher TAPSE and higher iv fluids infusion with significantly negative fluid balance within the first 24 hours were found (p < 0.05). CVP positively correlated with VCI- both inspiration (VCIi r = 0.5, p = 0.02) and expiration (VCIe r = 0.6, p = 0.002), RAA (r = 0.4, p = 0.05) and the dose of furosemide used in the first 24 hours (r = 0.6, p = 0.002). RAA correlated with both VCI dimensions (VCIe r = 0.42, p = 0.05 and VCIi r = 0.44, p = 0.04, respectively) and VCI negatively correlated with fluid balance (r = −0.56, p = 0.006). At the same time TAPSE positively correlated with iv fluid infusion (r = 0.41, p = 0.04).

**Conclusions:** CVP values were compatible with imaging variables in the evaluation of the volume status in patients hospitalized in ICCU. Patients who died instead of higher iv fluid therapy within the first 24 hours had negative fluid balance which, together with higher TAPSE, might suggest paradoxical dehydratation. At the same time false interpretation of CVP encouraged clinicians to the incorrect use of high doses diuretics. In this aspect, among other assessed parameters, TAPSE seems to be a better tool to guide the balance between the dose of diuretics and iv fluid therapy in critically ill non-septic cardiac patients.

## P128 Comparation between invasive and non invasive haemodynamic volume predictors in surgical patients on mechanical ventilation with i and ii degree abdominal hypertension-preliminary study

### M Siranovic, A Gopcevic, ZG Gavranovic, AH Horvat, H Krolo, B Rode, L Videc

#### Clinical hospital center, Zagreb, Croatia

**Introduction:** In this cohort observational preliminary study we aggregate hemodynamic parameters of some standard invasive metodes (PiCCO and LiDCO) with noninvasive obtain by ultrasound (US). Comparation in volume prediction was obtained among indexed intrathoracic blood volume (ITBVI) in PiCCO and LiDCO standard measurements versus noninvasive inferior vena cava pulsatility index (IVCPI) and velocity time variation (VTI var.) on aortic valve processed by US transthoracic approach after passive leg rising (PLR) manoevre. Referent metode was salina volume challenge and stroke volume (CI) change measured by PiCCO and LiDCO.

**Methods:** In this cohort study we included 19 patients (11 m and 8f) with I and II degree abdominal hypertension. APACHE II grade for this observed group was between12 and 20 . Laparotomy for abdominal surgical indications was done. All patient were mechanical ventilated. Abdominal pressure was measured trough the sistem located in urinary bladder. Invasive hemodynamic monitoring was processed with PiCCO and LiDCO concentratio dilution and thermodilution metods. Non invasive monitoring included ultrasound measurements using doppler for aortic VTI var. and M mode for IVCPI and caval diameter size. Former ultrasound aproach for VTI aortic variation was measured in five chamber view by pulsed wave doppler. For second US measurement subcostal M mod aproach for inferior vena cava was obtained. Statistic was performed using Med Calc. program ROC analysis for performed metodes.

**Results:** In comparation of static and dynamic parameters using ROC operating curve analysis with cut of margin of 80 percent area IVCPI (88%) and aortal VTI variation (89%) was better indicators than central venous tension (CVT) (58%) and intrathoracic blood volume (ITBVI) (66%) in this group of surgical patients using volume challange and cardiac output variation like a gold standard in volume response evaluation.

ROC curve for IVCPI

Area under the ROC curve = 0.885

ROC curve for VTI_var

Area under the ROC curve = 0.896

ROC curve for ITBVI

Area under the ROC curve = 0.667

ROC curve for CVT

Area under the ROC curve = 0.583

**Conclusions:** In this measurements of volume response evaluation using different methodes in surgical patients with I and II dgr. of abdominal hypertension we found good capability of dynamic methodes IVCPI and aortic VTI variations in prediction of volume response. This non invasive methodes have acceptable prediction of volume demand in comparation with standard methode.

## P129 Ultrasonic stroke volume variation by passive leg raising and fluid responsiveness in medical icu patients

### A Trifi, S Abdellatif, K Ben Ismail, A Bouattour, F Daly, R Nasri, S Ben Lakhal

#### University hospital center of La Rabta, Tunis, Tunisia

**Introduction:** the evaluation of the fluid responsiveness (FR) in ICU patients is a common dilemma for intensivists. It is believed that the static preload indices have a moderate interest in FR prediction. The passive leg rising (PLR) is an endogenous test to predict whether the stoke volume increases with volume expansion. By transferring a volume of about 300 ml of venous blood from the lower body to the right heart, the PLR mimics a volume expansion test.

The aim was to determine whether the ultrasound measurement of SV coupled with PLR can be used as a predictive tool for FR.

**Methods:** prospective cohort study of ICU patients requiring volume expansion. The measurement of SV was performed using a transthoracic-doppler echocardiography device. Four measurements were obtained: before and 90 minutes after PLR, before and after expansion of 500 ml of crystalloids over 15 minutes. Subsequently, patients were classified according to their hemodynamic response to volume expansion: VE (endogenous or exogenous). Responders were defined by an increase in SV of at least 15% in response to VE. Non-responders had an increase in SV <15%. The ROC curve was used to measure the predictive values of ultrasound variation of SV after PLR and VE. The Spearman test was used to study the correlation between two-SV variations.

**Results:** 30 VE were studied. Included patients had an average age of 48 years, sex ratio of 1.3 and SOFA score at 5.5. 23 patients (77%) were ventilated. The requiring of VE was justified by sepsis or septic shock in 24 cases (80%), diabetic ketoacidosis (5 cases) and acute adrenal insufficiency (1 case). The use of norepinephrine was noted in 12 patients (40%) and epinephrine in 1 patient. An increase in SV > 15% in response to PLR was reported in 21 cases. Elevation of SV > 15% in response to PLR was followed by a FR in 18/21 of cases with sensitivity at 94.7% and specificity at 72.7%. The SV variation after exogenous VE was significantly correlated with the SV variation in response to PLR with a Spearman score at 0.701 and p value = 0.01.

**Conclusions:** Transthoracic echocardiography-Doppler measurement of SV in response to PLR was shown to be a good predictor of FR. The interest of this test is mainly due to its sensitivity to 94.7% to predict the FR and its significant correlation with the SV variation after an EV.

## P130 Accuracy of the passing leg raising test in patients with intra-abdominal hypertension

### A Beurton^1^, JL Teboul^2^, V Girotto^2^, L Galarza^2^, C Richard^2^, X Monnet^2^

#### ^1^Hôpital de Bicêtre, Le Kremlin Bicêtre, France; ^2^Hôpital de Bicêtre, Hôpitaux universitaires Paris-Sud, Assistance publique – Hôpitaux de Paris, Le kremlin bicêtre, France

**Introduction:** The passing leg raising (PLR) test predicts preload responsiveness by inducing a reversible shift of venous blood from the lower part of the body toward the cardiac cavities. Intra-abdominal hypertension may impede the PLR-induced increase in cardiac preload by compressing the inferior vena cava. The aim of this study was to test the reliability of the PLR test to detect fluid responsiveness in patients with intra-abdominal hypertension.

**Methods:** At this stage of the study, we have included 13 patients where intra-abdominal pressure (IAP) was > =12 mmHg. We measured the changes of cardiac index (CI, pulse contour analysis, PiCCO2 device) during a PLR test and during a volume expansion (500 mL saline). We also measured IAP (bladder pressure) during PLR test and volume expansion.

**Results:** In 10 patients (“responders”), CI increased > =15% (22 ± 8%) during volume expansion. The IAP at baseline was 21 ± 4 mmHg in responders and 17 ± 1 mmHg in non-responders (p = 0.10). The PLR test increased CI in responders (11 ± 16%) but not in non-responders. The PLR test was positive (PLR-induced increase in CI > =10%) in 3 responders (true positives) and negative on 7 responders (false negatives). The PLR test was negative in 2 non-responders (true negatives) and positive in 1 non-responders (false positives). Thus, the sensitivity of the PLR test was 30% (95% confidence interval: 7–65%) and the specificity was 67(9–99)%. The positive predictive value was 75(19–99)% and the negative predictive value was 22(3–60)%. The PLR test decreased IAP in all patients (by 37 ± 16%). The baseline IAP was not different in false negatives than in true negatives.

**Conclusions:** Intra-abdominal hypertension is responsible for false negatives to the PLR test, although the PLR test reduces IAP. These are preliminary results and the study is ongoing.

## P131 The accuracy of the perfusion index to detect the variation of the cardiac index

### A Beurton, JL Teboul, V Girotto, L Galarza, C Richard, X Monnet

#### Hôpital de Bicêtre, Le Kremlin Bicêtre, France

**Introduction:** At best, the effects of volume expansion must be assessed through a direct measurement of cardiac index (CI). Nevertheless, measuring CI often requires invasive and costly techniques. The perfusion index (PI) is the ratio of the pulsatile infrared signal (systole) and the non-pulsatile signal (diastole) that is calculated from the oxygen saturation plethysmographic waveform. We made the hypothesis that the variations of PI could detect and track the variations of CI, especially when induced by a passing leg raising (PLR) test and a volume expansion (VE).

**Methods:** In 65 patients who were monitored for CI (PICCO2 device) and PI (Masimo), we measured and compared the simultaneous changes in CI and PI observed during a PLR test and VE (500 mL of saline)..

**Results:** The PLR test was positive in 27 cases (increase in CI ≥ 10%). In these cases, CI increased by 20 ± 10% and PI increased by 54 ± 37%. In patients with negative PLR test, neither CI nor PI significantly changed. If PI increased >9%, a positive response of CI to PLR could be diagnosed with a sensitivity of 100% (88–100%) and a specificity of 76% (59–88%) (area under the receiver operating characteristic curve: 0.93 (95% confidence interval: 0.83–0.97, p < 0.0001)). Volume expansion was performed in 22 of the 27 PLR responders. CI increased by ≥15% in 21 of these cases with volume expansion. During volume expansion, CI increased to 24 ± 12% and PI increased by 57 ± 55%. Taking into account all changes together (induced by PLR and VE), the correlation between the changes of CI and of PI was r = 0.67 (p < 0.001).

**Conclusions:** PI could detect the variations of CI during PLR and volume expansion. Monitoring PI may be a non-invasive alternative to CI monitoring to guide and assess fluid therapy.

## P132 Can carotid and femoral doppler assess the effects of passive leg raising?

### V Girotto^1^, JL Teboul^2^, A Beurton^2^, L Galarza^2^, T Guedj^3^, X Monnet^2^

#### ^1^Hôpital de Bicêtre, Le Kremlin-Bicêtre, France; ^2^Service de réanimation médicale, Inserm UMR S_999, Université Paris-Sud, Le Kremlin-Bicêtre, France; ^3^Hôpital de Bicêtre, Hôpitaux universitaires Paris-Sud, Assistance publique – Hôpitaux de Paris, Le Kremlin-Bicêtre, France

**Introduction:** For the moment, a reliable assessment of the hemodynamic effects of volume expansion and passive leg raising (PLR) requires a direct measurement of cardiac output, which is often invasive. However, it has been suggested that changes in common carotid and common femoral artery blood flows and in their velocities may reflect the variations in cardiac output. We tested whether these changes measured by Doppler could accurately follow the changes in cardiac output during PLR and volume expansion.

**Methods:** In 34 ICU patients, before and during PLR and before and after volume expansion, we measured cardiac index (pulse contour analysis, PiCCO2) and carotid and femoral blood flows, which were calculated from arterial diameter and velocity time integrals (for femoral arteries) or time average velocities (for carotid arteries). Peak systolic velocity was also recorded. We performed 53 PLR tests. Volume expansion was then performed in 23 cases where cardiac index increased > = 10% during PLR.

**Results:** Taking into consideration measurements performed during PLR and volume expansions (n = 76), cardiac index increased by 14,6 ± 13,7%, carotid blood flow by 15,3 ± 35,4% and femoral blood flow by 21,1 ± 36%. The correlation between changes in cardiac index and in carotid blood flow and between changes in cardiac index and in femoral blood flow were not significant (p = 0.60 and p = 0.69, respectively). In 27 patients, PLR was positive (increase in cardiac index > = 10%). During PLR in these patients, cardiac index increased by 19 ± 11%, carotid blood flow increased by 13 ± 38% and femoral flow increased by 3 ± 12%. Neither the changes in carotid blood flow (area under the ROC curve: 0.55 ± 0.10) nor the changes in femoral flow (area under the receiver operating characteristic (ROC) curve: 0.57 ± 0.16) could detect a positive PLR test. The changes in peak systolic velocity during PLR also failed to detect a positive PLR test, for carotid as for femoral blood flows.

**Conclusions:** The assessment of carotid and femoral artery blood flow as well as the measurements of their peak systolic velocity are not reliable methods to assess a PLR test in ICU patients.

## P133 Could bioreactance assess the effects of passive leg raising in critically ill patients?

### L Galarza, P Mercado, JL Teboul, V Girotto, A Beurton, C Richard, X Monnet

#### Hôpital de Bicêtre, Le Kremlin-Bicêtre, France

**Introduction:** Until now, reliable continuous monitoring of cardiac output (CO) in the intensive care unit has been achieved only by invasive methods. The bioreactance is a non-invasive method for CO monitoring based on the measurement of frequency modulation and signal phase shift of an electrical current crossing the thorax. Previous studies have shown different results with this technique. With a modified software version, where the time over which the device averages CO values was reduced, we tested if the bioreactance device (NICOM®, Cheetah Medical) was able to track the changes in cardiac index (CI) during passive leg raising (PLR) test and to predict fluid responsiveness when compared with transpulmonary thermodilution and pulse contour analysis device (PICCO®, Pulsion Medical Systems).

**Methods:** We included all patients with a PICCO® device where a PLR test was planned. We recorded CI with both devices before and during PLR. If a volume expansion was given (500 ml of saline during 10 minutes), we also recorded CI before and after. The PICCO ® system was calibrated with thermodilution before PLR and volume expansion.

**Results:** Thirty-eight PLR and 16 volume expansions were performed in 26 patients (60% septic shock; 77% under vasoactive support, noradrenaline median dose 0.18 mcg/kg/min (IQR 0.32); 80% sedated and under volume control ventilation). Regarding the agreement between absolute values, we included every pair of CI during PLR and volume expansions (n = 146). The median bias was 0.06 litre min^−1^ m^−2^ and limits of agreement were −1.89 and 2.01 litre min^−1^ m^−2^. The percentage of error was 60%. The 4-quadrant plot showed an 89% rate of concordance of Nicom® for tracking changes in CI induced for PLR and volume expansion with a 15% of exclusion zone. The PLR test was positive (increase in CI > =10%) in 15 “preload responsive” cases. The ability for detecting this change with Nicom® had a sensitivity of 87% and a specificity of 70% (AUC 0.77 ± 0.17). In case of volume expansion, for detecting an increase in CI > =15%, the sensitivity of Nicom® was 87%.

**Conclusions:** A software version of NICOM® device, where the averaging time of CO had been reduced, showed an acceptable ability for tracking changes in CI induced for PLR and volume expansion in ICU patients.

## P134 Reliability of lung ultrasound and inferior vena cava collapsibility index in volume status estimation in ICU patients during early postoperative period

### M Karaman Iliæ^1^, L Sakic^1^, V NN^1^, L Stojcic^2^

#### ^1^Clinical Hospital Sveti Duh, Faculty of Medicine, University of Osijek, Zagreb, Croatia; ^2^University of Zagreb, School of Medicine, Zagreb, Croatia

**Introduction:** A liberal approach to volume replacement therapy is commonly seen during surgical procedures. An excess of administrated fluid may result in an increase of Extra Vascular Lung Water (EVLW) and can cause gas exchange deterioration.

The aim of this study was to investigate whether Lung Ultrasound, a noninvasive bedside method, can be used in volume status estimation.

**Methods:** Sixty patients without known cardiac or pulmonary diseases admitted to the Intensive care Unit (ICU) after elective abdominal or vascular surgical procedure, were included in the study sample. Inferior Vena Cava collapsibility index (IVC cl) and PaO2/FiO2 ratio were measured and the occurrence of B-lines was monitored at the patients’ admission to the ICU (0 time), and after six, twelve and twenty-four hours. The appearance of B- lines < =7 mm together with IVC cl > = of 40% was taken as a sign of fluid overload and the rise of Extra Vascular Lung Water (EVLW). The value of the PaO2/FiO2 ratio lower than 200 was a sign of tissue oxygenation impairment.

**Results:** In 18/60(30%) of patients there were no signs of fluid overload. In 42/60(70%) of patients fluid overload was detected. The comparison of the appearance of B-lines < = 7 mm coupled with IVCcl > =40% to PaO2/FiO2 < 200 showed no difference in the time when fluid overload was detected (Wilcoxon test, P = 0.0113). Relations between individual measures (B-Lines vs PaO2/FiO2 and IVCcl vs PaO2/FiO2) that were tested at the given times, showed a statistically significant association between B lines and PaO2/FiO2(Chi-square test, P < 0.001 for all four times of measurement). The relation between IVCcl and PaO2/FiO2 was not statistically significant (Chi-square test, P = 0.071 or higher for all).

**Conclusions:** Our study showed that Lung Ultrasound had the same sensitivity as the PaO2/FiO2 ratio in the detection of fluid overload, and that Lung Ultrasound could be used for EVLW detection in the ICU patients during early postoperative period. These findings should be further verified in studies involving larger samples.

**References**

1. Lichtenstein DA. BLUE-protocol and FALLS-protocol: two applications of lungultrasound in the critically ill. Chest. 2015 Jun;147: 1659–70.

2. Bouhemad, M Zhang, Q Lu, JJ Rouby. Clinical review: bedside lung ultrasound in critical care practice. Crit Care 2007; 11: 205

## P135 Predicting fluid responsiveness by using combined end-expiratory and end-inspiratory occlusion tests with echocardiography

### M Jozwiak, F Depret, JL Teboul, J Alphonsine, C Lai, C Richard, X Monnet

#### Hôpitaux universitaires Paris-Sud, Hôpital de Bicêtre, Inserm UMR S_999, Univ Paris-Sud, Le Kremlin-Bicêtre, France

**Introduction:** The effects of end-expiratory occlusion on cardiac output were shown to reliably predict fluid responsiveness in patients under mechanical ventilation. Nevertheless the threshold value of 5% which was found requires that the method of measurement of cardiac output is of high precision, what is doubtful when echocardiography is used. We aimed at assessing whether 1) fluid responsiveness can be predicted by the effects of end-expiratory occlusion on the velocity-time integral (VTI) of the left ventricular outflow measured by echocardiography and 2) adding the effects of end-inspiratory to those of end-expiratory occlusions on VTI can predict fluid responsiveness with the same reliability as end-expiratory occlusion alone but with a higher threshold value, more compatible with the precision of echocardiography, which is assumed to be close to 5%.

**Methods:** In 30 patients, we measured pulse contour analysis-derived cardiac index (PiCCO2) and VTI during the 5 last seconds of 15-second end-inspiratory and end-expiratory occlusions, separated by 1 minute, and after infusing 500-mL saline. Patients where volume expansion increased cardiac index (transpulmonary thermodilution) > = 15% were defined as “responders”.

**Results:** End-expiratory occlusion increased VTI more in responders than in non-responders (11 ± 5% vs. 3 ± 1%, respectively, p < 0.0001) and end-inspiratory occlusion decreased VTI more in responders than in non-responders (12 ± 5% vs. 5 ± 2%, respectively, p = 0.0002). When adding the absolute values of changes in VTI observed during both occlusions, VTI changed by 23 ± 9% in responders and by 8 ± 3% in non-responders. Fluid responsiveness was predicted by the end-expiratory occlusion-induced change in VTI with an area under the receiver operating characteristic (ROC) curve of 0.938 (0.785–0.989) and a threshold value of 4% increase in VTI. Fluid responsiveness was predicted by the sum of absolute values of changes in VTI during both occlusions with a similar area under the ROC curve (0.973 (0.838–1)) and with a threshold of 13% change in VTI. In this case, the sensitivity was 93% (95% confidence interval: 68–100%) and the specificity was 93% (68–100%).

**Conclusions:** If consecutive end-inspiratory and expiratory occlusions change VTI > =13% in total, fluid responsiveness is accurately predicted. This threshold is more compatible with the precision of echocardiography.

## P136 Changes in ftc with peep titration, but not absolute value of ftc, predicts fluid responsiveness in patient with septic shock

### N Tapanwong, P Chuntupama, P Wacharasint

#### Phramongkutklao hospital, Bangkok, Thailand

**Introduction:** In septic shock, fluid optimization is an important feature in hemodynamic resuscitation. Corrected flow time (FTc) is one hemodynamic parameters derived from esophageal doppler monitoring. Even low FTc commonly results from hypovolemia, high FTc may results from decreased afterload conditions such as sepsis (1,2). Whether absolute value of FTc can predict fluid responsiveness in sepsis is not clarified. We hypothesized that, using the heart-lung interaction, changes in FTc following positive end-expiratory pressure (PEEP) titration from 5 to 15 cmH[sub]2[/sub]O (dFTcPEEP) may predicts fluid responsiveness in patients with sepsis better than absolute value of FTc.

**Methods:** We performed a prospective study in 15 patients with septic shock who were mechanically ventilated and had respiratory compliance above 30 mL/cmH[sub]2[/sub]O. Patients were categorized into 2 groups (based on changes in their cardiac output (CO) following 500 mL of fluid bolus), which were 1) fluid responder (increased of CO [>=]15% from baseline) and 2) fluid non-responder (increased of CO <15% from baseline). All patients were monitored for CO as well as FTc using esophageal doppler at the PEEP 5 cmH[sub]2[/sub]O, 15 cmH[sub]2[/sub]O, and immediately after 500 mL of fluid bolus with PEEP of 5 cmH[sub]2[/sub]O.

**Results:** There were 11 fluid responders and 4 fluid non-responders. Compared to fluid responders, fluid non-responders had a significantly lower dFTcPEEP (−19 vs 7 msec, p = 0.02) while there was no significant difference in absolute value of baseline FTc between two groups of patients (306 vs 328 msec, p = 0.6). We found significant correlation between dFTcPEEP and changes in CO after fluid bolus (r^2^ 0.51, p = 0.003). Analysis using ROC curve, we also found that dFTcPEEP [<=]-2 msec can predict fluid responsiveness with a sensitivity of 75%, and specificity of 82% (AUC 0.90, 95%CI 0.70–1.00, p = 0.02).

**Conclusions:** Changes in FTc following PEEP titration, but not absolute value of baseline FTc, can predict fluid responsiveness in patients with septic shock.

**References**

1. Singer M, Allen MJ, Webb AR, Bennett ED. Effects of alterations in left ventricular filling, contractility, and systemic vascular resistance on the ascending aortic blood velocity waveform of normal subjects. Crit Care Med. 1991;19:1138–45.

2. Singer M. The FTc is not an accurate marker of left ventricular preload. Intensive Care Med. 2006;32:1089.

## P137 Adjustment of stroke volume variation svv to biometric data and other contexts: a database analysis with an independent validation study

### W Huber, J Hoellthaler, T Lahmer, R Schmid

#### Klinikum rechts der Isar; Technical University of Munich, Munich, Germany

**Introduction:** SVV is a predictor of fluid responsiveness FR in patients with sinus rhythm (SR) and controlled mechanical ventilation (CMV). A number of confounders preclude the use of SVV in a substantial number of patients. We hypothesized that adjustment of SVV to some of these confounders and other parameters independently associated to SVV might improve its predictive capabilities regarding FR.

**Methods:** Analysis of a prospectively maintained database on SVV (10936 measurements in 608 patients). Spearman correlation of SVV to weight, height, gender and age as well as to heart rate HR, mechanical ventilation (MV) and heart rhythm (Rh: SR or atrial fibrillation). Primary endpoint: Multivariate regression regarding SVV in each patient. IBM SPSS 23.

**Results:** SVV was univariately associated to older age (r = 0.100; p = 0.023), HR (r = 0.334; p < 0.001), atrial fibrillation (r = 0.303; p < 0.001) and spontaneous breathing (r = 0.140; p = 0.002). Multivariate analyses regarding biometrics (R^2^ = 0.012) or contexts (R^2^ = 0.225) demonstrated independent association of SVV to older age (p = 0.008), HR (p < 0.001), atrial fibrillation (p < 0.001) and spontaneous breathing (p < 0.001). The R^2^-values suggest a much stronger association of SVV to contexts compared to biometric data. A combined multivariate analysis (R^2^ = 0.244) regarding biometric data and contexts showed an independent association of SVV to age (p = 0.002), weight (p = 0.010), HR (p < 0.001), atrial fibrillation (p < 0.001) and spontaneous breathing (p < 0.001). Based on these data, SVV_estimated can be predicted as −9.995 + 0.133*HR [/min] + 5.881 [for atrial fibrillation] - 2.642 [for mechanical ventilation] + 0.074*age [years] + 0.042*weight [kg]. Division of measured SVV by SVV_estimated results in the ratio “SVV_context-sensitive”. To validate SVV_context-sensitive we compared its predictive capabilities regarding FR defined as an increase in CI [>=]15% in 106 volume challenges in an independent group of patients with controlled (n = 28) or assisted ventilation (n = 39) or spontaneous breathing (n = 39). While SVV (ROC-AUC 0.573; p = 0.069) and CVP (AUC 0.388; p = 0.139) were not predictive, SVV_context-sensitive (AUC 0.670; p = 0.025) and GEDVI (AUC = 0.352; p = 0.05) significantly predicted FR.

**Conclusions:** SVV is independently associated to HR, Rh, mode of ventilation, old age and weight. Adjustment for these potential confounders might improve prediction of FR by SVV particularly in patients without CMV or SR.

## P138 Stroke volume guided resuscitation in severe sepsis and septic shock decreases time on pressors and ICU stay

### H Latham, CD Bengtson, L Satterwhite, M Stites, SQ Simpson

#### University of Kansas Medical Center, Kansas City, Kansas, United States

**Introduction:** The purpose of this study is to evaluate if a targeted volume resuscitation strategy aimed at optimizing stroke volume via non-invasive bioreactance monitoring would result in a decreased fluid balance and improved secondary outcomes in ICU patients with severe sepsis and septic shock.

**Methods:** Retrospective chart review of patients diagnosed with severe sepsis or septic shock treated in the medical or transplant ICUs at the University of Kansas Hospital from April 1-September 1, 2014. The study test group consisted of patients with severe sepsis or septic shock who underwent stroke volume targeted fluid resuscitation guided by NICOM (Cheetah-Medical, Newton Center, MA), during their ICU course. The comparison group consisted of matched consecutive patients with a diagnosis of severe sepsis or septic shock treated in the same ICU during the same time period, but who had fluid resuscitation guided by usual care at the discretion of the provider.

**Results:** The test group consisted of 100 identified patients compared with 91 patients in the usual care group. Medical histories were similar between the two groups, including mean SAPS II (p = 0.87). Fifty-three percent of patients who received treatment guided by NICOM measurement were found to be fluid responsive as determined by a change in the SVI of > 10 percent. When fluid balance over the entire ICU stay was compared between the two groups, patients in the test group exhibited a significantly decreased fluid balance (1.77 + 0.60 L) compared to 5.36 + 1.01 L in the usual care group (p = 0.002). There was no between group difference in the need for vasopressors (p = 0.25) or number of vasopressors required (p = 0.19). However, patients requiring vasopressors in the test group received this therapy for significantly less time (32.08 + 5.22 hours) compared to the usual care group (64.86 + 8.39 hours; p = 0.001). Importantly, patients in the test group exhibited a decreased ICU length of stay (5.98 + 0.68 days) compared to usual care (8.87 + 1.18 days; p = 0.03).

**Conclusions:** Optimization of stroke volume guided by NICOM in patients with severe sepsis and septic shock was associated with reduced fluid balance, and decreased duration of vasopressors. Reduced time on vasopressors was likely a major contributing factor to the reduced ICU length of stay in the test group.

## P139 Stroke volume guided resuscitation in severe sepsis and septic shock decreases need for mechanical ventilation

### H Latham, CD Bengtson, L Satterwhite, M Stites, SQ Simpson

#### University of Kansas Medical Center, Kansas City, Kansas, United States

**Introduction:** The purpose of this study is to evaluate if a targeted volume resuscitation strategy aimed at optimizing stroke volume via non-invasive bioreactance monitoring would result in a decreased fluid balance and improved secondary outcomes in ICU patients with severe sepsis and septic shock.

**Methods:** Retrospective chart review of patients diagnosed with severe sepsis or septic shock treated in the medical or transplant ICUs at the University of Kansas Hospital from April 1-September 1, 2014. The study test group consisted of patients with severe sepsis or septic shock who underwent stroke volume targeted fluid resuscitation guided by NICOM (Cheetah-Medical, Newton Center, MA), during their ICU course. The comparison group consisted of matched consecutive patients with a diagnosis of severe sepsis or septic shock treated in the same ICU during the same time period, but who had fluid resuscitation guided by usual care at the discretion of the provider.

**Results:** The test group consisted of 100 identified patients compared with 91 patients in the usual care group. Medical histories were similar between the two groups, including mean SAPS II (p = 0.87). Fifty-three percent of patients who received treatment guided by the NICOM measurements were found to be fluid responsive as determined by a change in the SVI of > 10 percent. When fluid balance over the entire ICU stay was compared between the two groups, patients in the test group exhibited a significantly decreased fluid balance (1.77 + 0.60 L) compared to 5.36 + 1.01 L in the usual care group (p = 0.002). Importantly, patients in the test group were less likely to require mechanical ventilation (relative risk, 0.51; CI 0.36 to 0.72; p = 0.0001).

**Conclusions:** Optimization of stroke volume guided by NICOM in patients with severe sepsis and septic shock was associated with a reduced fluid balance, and decreased need for mechanical ventilation. With less administered volume in the test group, it is logical that patients exhibited less pulmonary and peripheral edema and therefore would be less likely to progress to respiratory failure requiring mechanical ventilation. Importantly, the finding that 53% of patients in the test group were fluid responsive is consistent with other publications indicating that only half of critically ill patients are fluid responsive.[1]

**References**

1 Cavallaro F et al. Diagnostic accuracy of passive leg raising for prediction of fluid responsiveness in adults: systematic review and meta-analysis of clinical studies. Intensive Care Medicine. 2010;36(9):1475–1483.

## P140 Measurement of the inferior vena cava as a method of evaluating the fluid balance of mechanically ventilated patients

### T Skladzien, M Cicio, J Garlicki, W Serednicki, J Wordliczek

#### Szpital Uniwersytecki, Cracow, Poland

**Introduction:** Assessment of circulating volume and the requirement for fluid replacement are fundamental to resuscitation but remain largely empirical. The aim of the study was to investigate whether in mechanically ventilated patients the change of ratio of the diameter of inferior vena cava to the diameter of the thoracic aorta (IVC/Ao) depends on the daily balance of fluids. In addition, we evaluated the relationship measuring the central venous pressure (CVP) from the daily balance of fluids and the daily change of ratio of IVC/Ao and CVP.

**Methods:** The study included 42 mechanically ventilated patients treated in Intensive Care. In all patients was assessed using transthoracic ultrasound width of the inferior vena cava, and the thoracic aorta for the following five days. Recorded daily differences in the amount of fluid intake and lost. CVP measurement was performed.

**Results:** The mean IVC/Ao was 0.95 ± 0.21. The difference in intake and lost fluids within 24 hours was −231 ± 1214 ml. The average score of CVP was 9.7 ± CVP 4,6 cmH2O. There were no significant correlation between IVC/Ao within 24 h and the balance of fluid intake and lost (p = 0.62) and a change in CVP and the balance of fluid intake and lost (p = 0.87). However, there was statistical correlation between the daily change in the average value of CVP and the difference IVC/Ao within 24 h (p <0.001).

**Conclusions:** Daily IVC/Ao change does not correlate with daily fluid change in mechanically ventilated patients. That is why IVC/Ao could not be a good predictor of fluid request in mechanically ventilated patients.

## P141 Negative fluid balance is associated with a reduction in airway driving pressure in critically ill patients with fluid overload

### P Vargas, A Salazar, P Mercado, M Espinoza, J Graf

#### Clinica Alemana, Santiago, Chile

**Introduction:** Fluid overload (FO) has been associated with adverse outcomes in critically ill patients. Once reanimation was completed we routinely removed fluid excess with a protocol using diuretics or hemofiltration. We examined the effects of this strategy on respiratory system mechanics.

**Methods:** We included critically ill patients with a positive fluid balance >5 L after fluid resuscitation and no evidence of hypoperfusion. The protocol aimed a reduction of 50% of the FO in 24 h and 25% of the FO in the subsequent 24 h. Body weight (BW) and ventilatory parameters were registered at baseline, 24 and 48 h.

**Fig. 60 (abstract P141):**
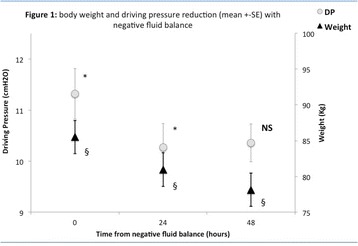
Body weight and driving presure reduction (mean + −SE) with negative fluid balance

**Results:** We studied 40 patients; 31 had ARDS, 12 had moderate ARDS. Characteristics of the population are presented in Table 18. The application of the protocol resulted in a mean BW reduction of 4.2 ± 2.5 Kg at 24 h and additional 2.7 ± 2.3 Kg at 48 h (8.3 ± 3.3% of initial BW reduction in 48 h). Despite no change in ventilator parameters, negative fluid balance was associated with a reduction in inspiratory plateau pressure (Pplat) and airway driving pressure (DPaw) from 20 ± 4 to 18.8 ± 4 and from 10.9 ± 3 to 9.7 ± 2.9 cmH2O at 24 h, respectively (p < 0.05 for both). These reductions were maintained at 48 h (fig. 60). The reduction in DPaw was most noticeable in those with the highest values at the beginning of the protocol; DPaw reduction of 2.8 ± 3.2 cmH2O (p < 0.05) for the highest quartile vs. 0.38 ± 2.3 cmH2O (NS) for the remainder at 24 h.

**Conclusions:** Negative fluid balance is associated with a reduction in Pplat and DPaw in critically ill patients with FO. Fluid removal could therefore be and adjunct to protective mechanical ventilation in reducing ventilator induced lung injury risk. This hypothesis should be tested in a randomized trial.

**Table 18 (abstract P141).** See text for description

Age	62 ± 15
APACHE II	20.4 ± 6.4
SOFA	8.7 ± 3.6
Septic shock (%)	78
Pneumonia (%)	33
PaO2/FiO2	250 ± 77
PEEP (cmH2O)	8.7 ± 3.4
Tidal volume (ml/Kg PBW)	6.3 ± 1.2

## P142 Positive cumulative fluid balance during the first week of acute respiratory distress syndrome as a predictor of 28-day mortality

### N Kongpolprom, N Sanguanwong

#### Chulalongkorn University, Bangkok, Thailand

**Introduction:** Acute respiratory distress syndrome (ARDS) is characterized by pulmonary edema, caused by an increase in pulmonary capillary permeability. Conservative fluid management significantly improves oxygenation and increases the number of ventilator free days. However, conflicting mortality-benefit exists in the literature.

We aimed to evaluate variables, particularly fluid balance, which could predict 28-day mortality in ARDS patients.

**Methods:** Retrospective study was conducted to identify predictors of 28-day mortality in ARDS patients. Data of ARDS patients admitted in medical department in our hospital between 2010 and 2014 were analyzed.

**Results:** Two hundred and ten ARDS patients were included in our study. The majority of their ARDS severities were moderate to severe. Overall, mortality at 28th day was 67%, which was associated with age, APACHE II, PaCO2, cumulative fluid balance in the first 3 days and the first 7 days. After multivariate analysis, only APACHE II and the first 7-day cumulative fluid balance were the independent predictors for 28-day mortality. Moreover, the risk of death increased with increasing 7-day fluid balance (Table 19).

**Conclusions:** The study demonstrated the more positive cumulative fluid balance during the first week of ARDS, the higher 28-day mortality. The first 7-day cumulative fluid balance threshold for increased risk of 28-day mortality in ARDS patients was −1,000 ml. However, further studies are needed to confirm these results.

**Table 19 (abstract P142).** See text for description

Cut off values of 7-day fluid balance	Odds ratio	95% CI	p value
− 5000 ml	2.25	0.54 to 9.28	0.264
− 1000 ml	2.95	1.24 to 7.02	0.014
0 ml	2.95	1.31 to 6.65	0.009
+1000 ml	3.99	1.95 to 8.19	<0.001
+5000 ml	3.90	2.10 to 7.24	<0.001
+10000 ml	4.98	2.21 to 11.22	<0.001
+15000 ml	10.58	1.39 to 80.64	0.023

Legend: The cumulative fluid balance in 7 days and risk of 28-day mortality

## P143 The association of intravenous fluid administration on patient outcomes in critical care

### S Jonnada^1^, C Gerrard^2^, N Jones^2^

#### ^1^University of Cambridge, Cambridge, United Kingdom; ^2^Papworth Hospital, Papworth, United Kingdom

**Introduction:** It is well known that fluids are vital to patient management after cardiac surgery. The question is, how much do you give? Some of the latest research suggests that that the previously held belief of “the more fluid, the better” might not hold true.^1,2^.

**Methods:** To help answer our question of how much, we carried out a retrospective observational analysis of patients admitted to an intensive care unit at Papworth Hospital, a cardiothoracic centre, between 01/01/2014 and 31/12/15. After extracting data from an electronic database, patients were divided into four quartiles, determined by daily IV fluid administration (FA). Parameters analysed included mortality, CAM-ICU status, alkaline phosphatase score and bilirubin score. These two scores were defined by the number of times they were higher than the upper limit of normal for the parameter, using the highest value during the patient’s stay. Each quartile was compared to Quartile 1 (Q1) for a variety of different adverse outcomes, using relative risk (RR) and 95% confidence intervals (CI).

**Results:** Although 5519 patients were found at first, only 4222 qualified for the study as 945 patient records contained missing data. Each quartile was therefore composed of 1055 or 1056 individuals. A statistically significant relationship was found between higher FA and unfavourable patient outcomes in patients in Q4 compared to Q1 for mortality (RR = 109.1 (CI 6.7–1764.3)), CAM-ICU status (RR = 7.4 (CI 5.6–9.6)), an alkaline phosphatase score >3 (11.1 (CI 2.6–46.7)) and a bilirubin score >3 (RR = 6.0 (CI 3.0–12.1)). The results found here are similar to those found in other studies^1,2^.

**Conclusions:** The correlations found here between increased FA and mortality and morbidity are significant, suggesting fluid overload can have serious negative consequences. However, further statistical analysis is needed to determine whether these are independent associations, and if so, this may then support a more restrictive approach..

**References**

1. Pradeep, A., et al. High volumes of intravenous fluid during cardiac surgery are associated with increased mortality. HSR Proceedings in Intensive Care and Cardiovascular Anesthesia 2 (2010): 287–296.

2. Lee, J., et al. Association between fluid balance and survival in critically ill patients. Journal of internal medicine 277.4 (2015): 468–477.

## P144 Restrictive fluid therapy in septic shock – is peripheral noradrenaline the answer?

### T Morley, PT Thorburn, A Trimmings

#### East Sussex Healthcare NHS Trust, Eastbourne, United Kingdom

**Introduction:** Fluid resuscitation is central to the management of septic shock[1]. The topic has attracted much attention over the last decade with a raft of contradictory results. In 2011 a study published in the NEJM was stopped early because aggressive fluid resuscitation in children with sepsis had significantly worse outcomes [2]. This led many to question whether fluid is actually a good thing and focus has shifted to restrictive fluid regimes.

At our institution we are considering the use of peripheral noradrenaline to enable earlier vasopressor therapy in septic shock. We first wanted to quantify how much fluid was being administered to those with septic shock before noradrenaline was started. This was the period after “adequate fluid resuscitation” in areas outside the ICU and before noradrenaline was started. We hypothesised that delay in placement of central venous catheter (CVC) may delay vasopressor administration and potentially increase unnecessary fluid administration.

**Methods:** Our Trust is a DGH consisting of 19 mixed surgical and medical critical care beds across two separate sites. We retrospectively analysed all ICU admissions with ICNARC coding of “sepsis” between April 2015 and April 2016. Diagnosis of septic shock was also confirmed by interrogation of the notes and requirement for vasopressor support after adequate fluid resuscitation outside the ICU. Patients taken directly from theatre and re-patriated were excluded. We looked at the time taken to place CVC, start noradrenaline and the amount of fluid administered on ICU before noradrenaline was started.

**Results:** 79 patient were identified as having septic shock and were included in the analysis. The mean time taken to place a CVC was 2 hours 48 minutes and mean time to commencing noradrenaline was 4 hours 53 minutes. The mean volume of fluid administered prior to starting vasopressors was 1174 ml. This did not include fluid resuscitation on the ward.

**Conclusions:** Our results show that large amounts of fluid are being administered to patients with septic shock on admission to ICU before vasopressor initiation. It appears from the data that placement of a CVC represents a barrier to the timely initiation of vasopressors. Peripheral administration may represent a means of overcoming this delay.

**References**

1. M. Singer et al., The Third International Consensus Definitions for Sepsis and Septic Shock (Sepsis-3). JAMA 315, 801–810 (2016).

2. K. Maitland et al., Mortality after fluid bolus in African children with severe infection. N Engl J Med 364, 2483–2495 (2011).

## P145 The impact of perioperative fluid balance on postoperative complications

### T Musaeva, I Zabolotskikh

#### Kuban State Medical University, Krasnodar, Russia

**Introduction:** Fasting, anaesthesia and surgery affect the body’s physiological capacity not only to control its external fluid and electrolyte balance but also the internal balance between the various body fluid compartments. Conversely, abnormalities of fluid and electrolyte balance may adversely affect organ function and surgical outcome. The aim of the study -to determine association between fluid administration during the perioperative period and complication development after major abdominal operations.

**Methods:** A retrospective study of the perioperative period after major abdominal operations in 560 patients was performed. The physical condition of patients corresponded to 3 class of ASA. The median age was 46.0 (38,0–62,0) years. The duration of the operations was more than 180 minutes. All patients received standart fluid management according to the ICU rules and were divided into 2 groups according to complication development during postoperative period: 1 - with complicated postoperative period (n = 169), without complications (n = 391).

**Results:** Patients with complications had significantly greater cumulative positive fluid balance, than patients without complications on postoperative day 1 (34,5 (19,5–44,3) and 21,9 (7,1–31,3) ml/kg; p > 0.05), day 3 (80,4 (61,1–106,2) and 38,2 (21,2–58,7) ml/kg; p <0.03), day 5 (115,2 (79,2–118,5) and 40,6 (17,7–58,6)ml/kg; p <0.01), day 7 (134,4 (107,9–166,8) and 42,2 (24,3–61,9)ml/kg; p <0.01), day 9 (135,3 (118,8–158,4) and 47,5 (29,4–68,6)ml/kg; p <0.01). Multivariable regression analysis demonstrated that cumulative fluid balance in day 3 (risk ratio =2.12, 95% CI =1.36–3.51, P-value =0.001) was independent risk factor for postoperative complications.

**Conclusions:** It is necessary to count the cumulative balance, along with the daily balance as accumulated fluid can be significant. Positive fluid balance in postoperative day 3 was a significant risk factor for complications in patients after major abdominal operations.

**References**

Zabolotskikh IB. et al. European Journal of Anaesthesiology 2015; 32(53): 260.

## P146 Early protocolized post-resuscitation fluid overload removal is safe

### A Salazar, P Vargas, P Mercado, M Espinoza, J Graf

#### Clinica Alermana, Santiago, Chile

**Introduction:** Fluid overload has been associated with adverse outcomes in critically ill patients. We examined the feasibility and safety of a protocol for early and fast post-resuscitation fluid overload removal.

**Methods:** We prospectively included mechanically ventilated critically ill patients with a positive fluid balance (PFB) >5 L after fluid resuscitation, edema, no evidence of hypoperfusion and stable vasopressor dose for >6 hours. Absence of hypoperfusion was defined by lack of skin mottling, blood lactate level <2.2 mM/L and central venous oxygen saturation (SvcO2) >70%. Negative fluid balance was induced with furosemide or hemofiltration (CVVHF), with a target of removing 75% of the PFB in 48 h. We registered body weight (BW), renal, metabolic, perfusion and hemodynamic variables at baseline and 48 h. The protocol was stopped if the patient showed evidence of hypoperfusion or doubled the initial vasopressor dose. Parenteral potassium and water were added in patients with furosemide infusion.

**Results:** We included 40 patients mean age 61 ± 15 years, 22 male, APACHE II 20.4 ± 6.4, SOFA 8.7 ± 3.6. Main diagnoses were sepsis (n = 31) and trauma (n = 5). PFB was 10.3 + 3.6 L. Fluid removal was started 2.5 ± 1.7 days after ICU admission with furosemide in 30 patients and CVVHF in the remainder. In 3 patients the protocol was stopped due to hypoperfusion. The application of the protocol resulted in a BW reduction of 6.9 ± 2.6 Kg at 48 h (8.3 ± 3.3% of initial BW). There was no change in renal function or electrolyte disturbances. Mild metabolic alkalosis and a drop in cardiac output (CO) without hypoperfusion were evident at 48 h (Table 20).

**Conclusions:** An early post-resuscitation fluid overload removal protocol is safe without inducing renal failure, electrolyte disturbances or hypoperfusion.

**Table 20 (abstract P146).** See text for description

	Baseline	48 h	p
BUN (mg/dL)	29.5 ± 18.9	31.4 ± 14.3	0.59
Creatinine (mg/dL)	1.10 ± 0.59	0.99 ± 0.41	0.09
Na (mEq/L)	142 ± 4	139 ± 3	<0.01
K (mEq/L)	4.2 ± 0.5	4.4 ± 0.7	0.04
HCO3 (mEq/L)	24.3 ± 2.8	27.6 ± 3.1	<0.01
CO (L/min)	6.5 ± 2.5	5.7 ± 1.3	<0.01
SvcO2 (%)	78 ± 7	76 ± 5	<0.01
Lactate (mM/L)	1.6 ± 0.6	1.4 ± 0.4	0.04
va pCO2 gap (mmHg)	4.6 ± 1.9	5.0 ± 3.7	0.70

## P147 The impact of resuscitation fluid bag size availability on the volume of fluid administration in the ICU (the tiring study)

### S Horst^1^, M Lipcsey^1^, R Kawati^1^, A Pikwer^2^, J Rasmusson^3^, M Castegren^4^

#### ^1^Department of Surgical Sciences, Uppsala, Sweden,^2^Eskilstuna County Hospital, Eskilstuna, Sweden,^3^Gävle County Hospital, Gävle, Sweden,^4^Karolinska University Hospital Solna, Stockholm, Sweden

**Introduction:** Iatrogenic fluid overload is associated with increased mortality in the intensive care unit (ICU). Decisions on fluid therapy in ICU patients are, at times, not based on physiological endpoints. 1 and Wwe hypothesized that that psychological factors such as larger volume available in fluid bags would lead to a more generous fluid therapy.

**Methods:** We performed a prospective interventional cross-over study at 3 Swedish ICUs by replacing the standard resuscitation fluid bag of Ringers Acetate 1000 ml available at each unit with 500 ml bags for 5 separate months (interventional group) comparing it to 5 months with 1000 ml bag size as controls. The primary endpoint was the amount of resuscitation fluid per patient during ICU stay.

**Fig. 61 (abstract P147):**
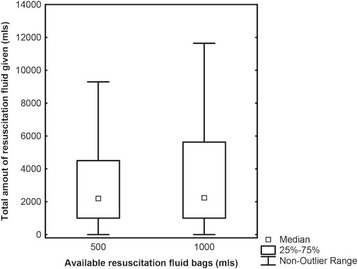
Resuscitation fluid bag size vs. total amount of fluid given

**Results:** 437 ICU adult patients, 231 in the interventional group and 206 in the control group, were included. There were no difference in the amount of resuscitation fluid per patient in the interventional group vs the control group (2200 (1000–4500) vs 2245 (1000–5630) ml (Fig. 61)s; median (IQR)), in CRRT rate (14 vs 12%), 90 day mortality (19 vs 18%) or in cumulative fluid balance (1005 (−50–2502) vs 931 (−103–2333) ml) in the in the interventional vs control groups.

**Conclusions:** We concluded that the amount of resuscitation fluid administered to ICU patients is not affected by the size of the fluid available in the fluid bags.

**References**

1. Cecconi M, Hofer C et al., Fluid challenges in intensive care: the FENICE study, Intensive Care Med (2015) 41:1529–1537.

## P148 Characteristics of patients with elevated troponin I level and prompt coronary arteries

### A Shilova, A Yafarova, M Gilyarov

#### Moscow Clinical City Hospital #1 named after N. Pirogov, Moscow, Russia

**Introduction:** Pathophisiology, management, and outcomes of tacotsubo (stress) cardiomiopathy and MI with intact coronary arteries remain unclear.

**Methods:** We have analyzed respectively 39 cases of elevated troponin I level and prompt coronary arteries, divided in two groups depending on final diagnosis. Of 39 patients, 14 cases of tacotsubo (stress) cardiomiopathy, and 25 cases of myocardial infarction (MI) with no significant coronary artery lesions, hospitalized in Moscow Civil Hospital #1 in Moscow in year 2016. Patients in two groups were age- and sex-matched.

**Results:** Of 14 patients with takotsubo cardiomiopathy, 85.5% were women (mean age 68.75 ± 2.34). Of 25 patients with MI with intact coronary arteries 16 were men (mean age 52.8 ± 1.57). Emotional triggers were more common, than physical in tacotsubo patients (50% vs 35%, p < 0.05), in 15% no evident triggers were found. In 28% patients, tacotsubo cardiomopathy caused acute heart failure in early postoperative period, which was significantly higher, then in MI group. In 50% of patients with tacotsubo psychiatric abnormalities (severe dementia, delirium or schizophrenia) were found, which was significantly higher when compared with patients with MI (13%). Mean levels of hs troponin I were higher in patients with MI (0.74 ng/dl vs 0.14 ng/dl; p < 0.05). Rates of severe heart failure (Killip 3–4) were markedly lower in MI patient (8% vs 57%; p < 0.001). Mortality rate was similar in both groups (p = 0.7). High troponin levels, low EF, anemia and delirium on admission were independent predictors for severe in-hospital complications.

**Conclusions:** Patients with Tacotsubo cardiomiopathy were more likely to suffer from neurogical and psychiatric disorders compared with those with MI on intact coronary arteries. It seems like postsurgical patients are more likely to suffer from tacotsubo cardiomiopathy and to be the main cause of troponin elevation. Nature and late outcomes of both conditions are to be further investigated.

## P149 Perioperative effects of remifentanil vs fentanyl on cardiovascular and humoral response in elderly patients undergoing total gastrectomy

### DL Loncar Stojiljkovic

#### Special Gynecological Hospital, Jevremova, 11000, Serbia

**Introduction:** Upper abdominal operations demand higher doses of opioids. The aim of the study was to investigate whether the ulrashort-acting opioid remifentanil provide better cardiovascular and humoral response control than fentanyl during and after total gastrectomy in elderly parient.

**Methods:** A total of 60 patients aged over 65 years (ASA II calssification) were randomised in two equal groups to receive remifentanil (0.1 μ g/kg continuous iv infusion) or fentanyl (1.5 μ g/kg iv bolus), as analgesic part of general balanced anaesthesia and continued postoperatively. The values of cardiovascular parameters – blood pressure and heart rate and level of cortisol, glicemia was measured at six points in time: baseline value-before inducion in anaesthesia (0), after intduction (1), two minutes after intubation (2), after manipulation with abdominal organs (3), after extubation (4), after 12 h and 24 h postoperatively (5,6).

**Results:** After induction of anaesthesia, a greater decrease of systolic pressure was found in the remifentanil group (32% ± 5 vs. 25 ± 4%). However, in the same group after the intubation of trachea a smaller increase of systolic pressure was registered (16% vs. 33%).

Systolic blood pressure was less incressed during the phase of extubation in fentanyl group (24%). Diastolic pressure was significantly higher during the phase of extubation in fentanyl group. After induction in anaesthesia, a significant fall in diastolic blood pressure was found in remifentanil group

Levels of cortisol were increased after extubation, but significantly more in the remifentanil group. Level was significantly increased after 12 h of the operation in the both groups. After 24 h was increased but not significantly. Glycaemia was without changes after intubation. After first incision and during procedure. Glucose level was significantly increased in phase of tracheal extubation only in remifentanil group. During the postoperative period was within the normal values in both groups.

**Conclusions:** Fentanyl assured better cardiovascular and humoral stability than remifentanil in elderly gastrectomised patients.

## P150 Clevidipine versus sodium nitroprusside as adjunct therapy to esmolol in aortic dissection

### A Ulici, S Reidt, T Lam, J Jancik

#### Hennepin County Medical Center, Minneapolis, MN, United States

**Introduction:** The purpose of this study was to compare clevidipine (CLEV) vs sodium nitroprusside (SNP) as adjunct agents to esmolol (ESM) for blood pressure (BP) management in aortic dissection. Intravenous (IV) vasodilators are commonly added to beta-blocking agents to reach BP goals in aortic dissection [1]. Our institution has transitioned to adding CLEV instead of SNP to standard therapy ESM for initial BP management. CLEV has been proven efficacious in lowering BP in both pre- and post-operative cardiac procedures and hypertensive emergencies [2, 3]. To our knowledge, this is the first study to evaluate CLEV use in acute aortic dissection.

**Methods:** A single-center retrospective chart review evaluated all patients diagnosed with aortic dissection from September 2010 through September 2016. Included patients were over 18 years old with new diagnosis of aortic dissection at presentation, were initiated on ESM as their primary beta blocker, received CLEV or SNP adjunct therapy and had complete hemodynamic data. Excluded patients were initiated on IV beta-blockers other than ESM, received other concomitant IV anti-hypertensive therapies, did not reach primary endpoint prior to surgical management, or were pregnant or breastfeeding. The primary endpoint was defined as time to reach patient specific systolic blood pressure (SBP) goals after ESM initiation. The secondary endpoint was defined as efficacy of CLEV and SNP for maintaining BP within patient specific goals using area under the curve (AUC) analysis of both positive and negative excursions until 24 hours post agent initiation or time of surgical management, whichever was less. Statistical analyses were conducted using R statistical software.

**Results:** Fourteen patients were included in final analyses: CLEV = 8, SNP = 6. The majority of patients were male (78.5%) diagnosed with Stanford Type-B aortic dissection (54.1%). Median SBP immediately prior to initiation of ESM was 162 mm Hg vs 160.5 mm Hg for CLEV and SNP groups, respectively. SBP goal was reached in a median 1.68 hours vs 1.03 hours for CLEV and SNP, respectively (p = 0.99). Median AUC was lower for patients treated with CLEV (206.9 mm Hg x min/hr vs 538.9 mm Hg x min/hr; p = 0.1079).

**Conclusions:** CLEV administration during initial aortic dissection medical management showed similar efficacy to SNP when used as adjunct therapy to ESM. CLEV is a reasonable alternative to SNP in acute aortic dissection based on our institution’s evaluation.

**References**

1. Erbel R et al. European Heart Journal. 22:1642–81, 2001

2. Powroznyk AV et al. European Journal of Anaesthesiology. 20:697–703, 2003

3. Pollack CV et al. Annals of Emergency Medicine. 53:329–38, 2009

## P151 Continuous infusion of furosemide versus intermittent boluses in acute decompensated heart failure: non-invasive evaluation of the effect on thoracic fluid content

### D Ragab, K Taema, W Farouk, M Saad

#### Cairo University, Cairo, Egypt

**Introduction:** The administration of loop diuretics in the management of acute decompensated heart failure (ADHF) whether IV boluses or continuous infusion is still controversial. We intended to evaluate differences between the two administration routes on the thoracic fluid content (TFC) and the renal functions.

**Methods:** Sixty patients with ADHF admitted to the critical care medicine department (Cairo University, Egypt) were initially enrolled in the study. Twenty patients were excluded due to EF > 40%, myocardial infarction within 30 days, and baseline serum creatinine level > 4.0 mg/dL. Furosemide (120 mg/day) was given to the remaining 40 pts who continued the study after 1:1 randomization to either continuous infusion (group-I, 20 pts) or three equal intermittent daily doses (group-II, 20 pts). Subsequent dose titration was allowed after 24 hours, but not earlier, according to patient’s response. No other diuretic medications were allowed. All patients were daily evaluated for NYHA class, urine output, TFC, body weight, serum K+, and renal chemistry.

**Results:** The median age (Q1-Q3) was 54.5 (43.8–63.8) years old with 24 (60%) males. Apart from TFC which was significantly higher in group-I, the admission demographic, clinical, laboratory and co-morbid conditions were similar in both groups. There was statistically insignificant tendency for increased urine output during the 1st and 2nd days in group-I compared to group-II (p = 0.08). The body weight was decreased during the 1st day by 2 (1.5–2.5) kg in group-I compared to 1.5 (1–2) kg in group-II, (p = 0.03). These changes became insignificant during the 2nd day (p = 0.4). The decrease of TFC was significantly higher in group-I than in group-II [10 (6.3–14.5) vs 7 (4–10)1/kΩ during the first day and 8 (6–11) vs 6 (4–9)1/kΩ during the second day in groups-I&II respectively, P = 0.02 for both]. There was similar NYHA class improvement in both groups (p = 0.7). The serum creatinine was increased by 0.2 (0.1–0.5) vs 0 (−0.1–0.2) mg% and the eGFR was decreased by 7.4 (4.5–12.3) vs 3.1 (0.2–8.8)ml/min/1.73 m2 in groups-I&II respectively (p = 0.009 and 0.02 respectively).

**Conclusions:** We concluded that continuous furosemide infusion in ADHF might cause greater weight loss and more decrease in TFC with no symptomatic improvement and possibly with more nephrotoxic effect.

## P152 In vitro ubiquinol increases cellular oxygen consumption in peripheral blood mononuclear cells from patients with metabolic stress

### X Liu, MJ Holmberg, A Uber, S Montissol, M Donnino, LW Andersen

#### Beth Israel Deaconess Medical Center, Boston, MA, United States

**Introduction:** The objective of the current study was to investigate the effects of in vitro ubiquinol (coenzyme Q10) administration on cellular oxygen consumption in peripheral blood mononuclear cells (PBMCs) from patients undergoing coronary artery bypass grafting. Ubiquinol (Coenzyme Q10) is a mitochondrial molecule that is essential for adequate aerobic metabolism. This population was evaluated to capture pre- and post blood sampling for patients in a state of severe metabolic stress.

**Methods:** Patients scheduled for coronary artery bypass grafting with cardiopulmonary bypass were enrolled at Beth Israel Deaconess Medical Center, a tertiary care center in Boston, USA, between January 2015 and July 2015. Blood was drawn and PMBCs were isolated from the patient before and after surgery. Cells were then randomized to either treatment with placebo or 1 μ g/mL ubiquinol. The complete mitochondrial respiration profiles were measured using XF Cell Stress Mito Kit (Seahorse Bioscience) to reveal the key parameters of cellular oxygen consumption. Wilcoxon Signed Rank test was used to analyze differences in oxygen consumption rate between groups.

**Results:** Basal cellular oxygen consumption was available on 23 patients pre-operatively and 17 patients post-operatively. The mean age was 71 (SD: 7), and 22/26 (85%) were male. We found a significant difference in post-operative relative basal (1.1 mL/min/mg difference [0.9, 1.6], p < 0.001) oxygen consumption and maximal (4.2 mL/min/mg difference [0.3, 7.0], p = 0.01) oxygen consumption between the ubiquinol and placebo group. There were no significant differences in pre-operative basal (1.0 mL/min/mg [−0.9, 2.2], p = 0.08) or maximal (0.5 mL/min/mg [−4.3, 7.3], p = 0.56) cellular oxygen consumption between the ubiquinol and placebo.

**Conclusions:** In a sample of cardiac surgery patients, in vitro administration of ubiquinol enhanced post-operative cellular oxygen consumption. These findings suggest ubiquinol may have potential as a mitochondrial resuscitator in states of metabolic stress, akin to typical physiology for critically ill patients in a state of shock.

## P153 Lazaroid (u-74389 g) prevents lung ischemia-reperfusion injury caused by thoracoabdominal aortic occlusion

### F Perlikos^1^, M Lagiou^2^, A Papalois^3^, C Kroupis^2^, I Toumpoulis^4^

#### ^1^Evangelismos Hospital, Athens, Greece; ^2^Attikon Hospital, Athens, Greece; ^3^ELPEN Research and Experimental Center, Athens, Greece; ^4^Department of Cardiac Surgery, Attikon Hospital, Athens, Greece

**Introduction:** Lung ischemia-reperfusion injury after thoracoabdominal aortic occlusion represents a major complication, which increases morbidity and mortality. In the present study we hypothesized that lazaroid U-74389G intravenous administration protects from lung ischemia-reperfusion injury through lipid peroxidation inhibition.

**Methods:** A total of 24 pigs were randomized in three groups. Group I (n = 8) underwent sham operation, group II (n = 8) underwent thoracoabdominal aortic occlusion for 45 min and received placebo and group III (n = 8) received 3 doses of lazaroid (3 mg/kg) 60 and 30 min before thoracoabdominal aortic occlusion and at 30 min of thoracoabdominal aortic occlusion (duration 45 min). Aortic occlusion was performed with aortic balloon-catheters under fluoroscopic guidance. All animals were sacrificed at the 7th postoperative day and lung specimens were received for molecular analysis.

**Results:** mRNA levels of leukotrienes LB4, LC4 and nitric oxide synthase isoforms including eNOS, nNOS and iNOS were determined with real-time RT-PCR. Nitric oxide can either induce (iNOS) or inhibit (eNOS and iNOS) lipid peroxidation based on its specific isoform origin. Group III showed significantly reduced levels of both LB4 (−63.7%) and LC4 (−35.9%) when compared with group II (P < 0.05). Isoform nNOS was not detected in lung specimens of all three groups. iNOS was significantly reduced (−60.2%) in Group III when compared with group II (P < 0.05). Finally, eNOS was slightly increased (+2.1%) in group III when compared with group II (P = 0.467).

**Conclusions:** Lazaroid U-74389G may represent an effective pharmacologic intervention in reducing lung ischemia-reperfusion injury following thoracoabdominal aortic occlusion.

This study has been partially funded by a research grant from the Hellenic Thoracic Society and was conducted at ELPEN Experimental Research Center.

**References**

1. den Hengst W.A, Gielis J.F, J.Y. et al. Lung ischemia-reperfusion injury: a molecular and clinical view on a complex pathophysiological process. Am J Physiol Heart Circ Physiol November 2010;299:(5) 1283–1299

2. Kuwaki K, Komatsu K, Sohma H, et al. The effect of various doses of lazaroid U74389G on lung ischemia reperfusion injury. Thorac Cardiovasc Surg. 1999 Apr;47(2):67–72.

3. Fisher A.B, Dodia C, Tan Z.T, et al. Oxygen-dependent lipid peroxidation during lung ischemia. J Clin Invest. 1991 August; 88(2): 674–679.

## P154 Nitric oxide in cardiac surgery. A meta-analysis of randomized trials

### E Osawa^1^, D Carter^1^, S Sardo^1^, J Almeida^1^, F Galas^1^, S Rizk^1^, R Franco^1^, L Hajjar^1^, G Landoni^2^

#### ^1^Heart Institute, Sao Paulo, Brazil; ^2^San Raffaele, Milan, Italy

**Introduction:** Nitric oxide may be used in many medical conditions, such as primary pulmonary hypertension and acute pulmonary embolism. In the setting of cardiac surgery, its role is unknown. The aim of this study was to to investigate the efficacy and safety of perioperative administration of nitric oxide in cardiac surgery.

**Methods:** The authors conducted a systematic review of randomized, controlled, parallel-group trials in accordance with a previously registered protocol (International Prospective Register of Systematic Reviews registration no. PROSPERO 2016:CRD42016032702), from inception to March 2016. Primary outcome was intensive care unit (ICU) stay, secondary outcomes were mortality, duration of mechanical ventilation, reduction of mean pulmonary artery pressure.

**Results:** The study included 17 RCTs comprising 760 patients. We calculated the pooled odds ratio (OR) and the mean difference (MD) with Random-Effects model. Quantitative synthesis of data demonstrated a significant reduction in the length of ICU stay (mean difference −0.33 days, CI [−0.59, −0.07] p = 0.01) and of mechanical ventilation duration (MD −4.88 hours, CI [−8.07, −1.69] p = 0.003) when compared to controls with no differences in mortality.

**Conclusions:** Nitric oxide might be beneficial in patients with pulmonary hypertension undergoing cardiac surgery. Large randomized trials are needed in order to further assess its effect on survival.

**References**

1. Copenhagen: The Nordic Cochrane Centre, The Cochrane Collaboration: Review Manager (RevMan). 2014

2. Greco T, Biondi-Zoccai G, Gemma M, Guérin C, Zangrillo A, Landoni G: How to impute study-specific standard deviations in meta-analyses of skewed continuous endpoints? World J Meta-Anal 2015; 3:215–224

**Fig. 62 (abstract P154).**
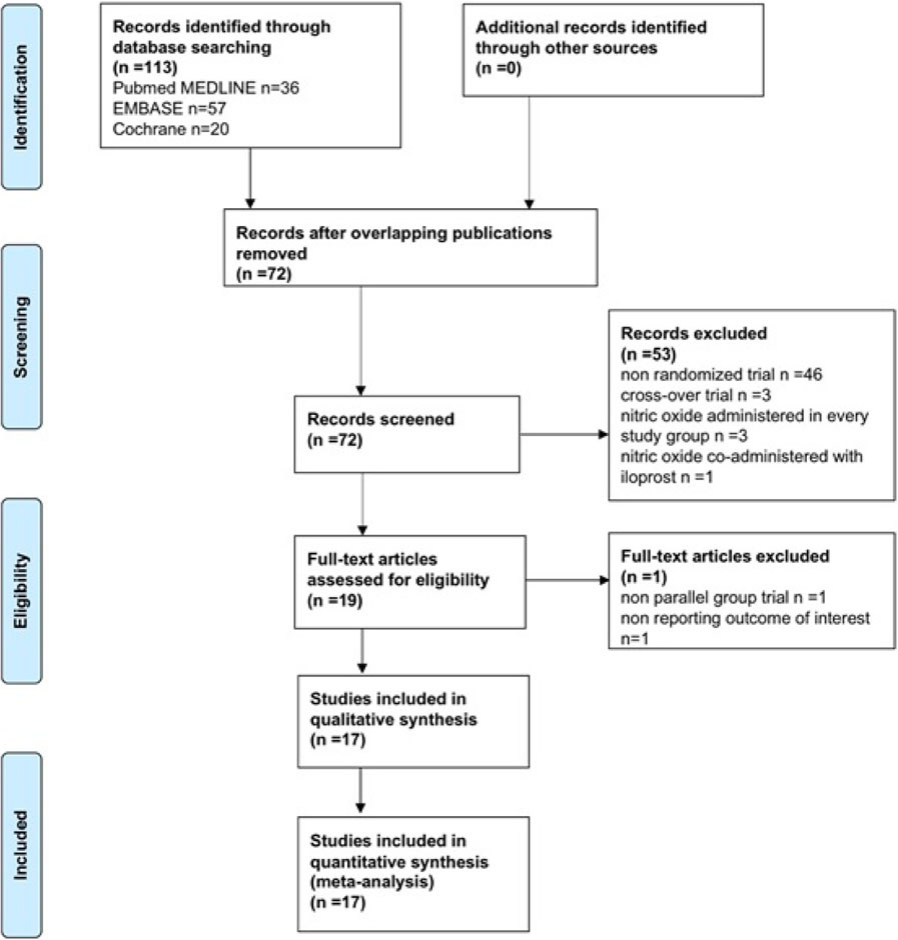
PRISMA flow diagram

**Fig. 63 (abstract P154).**
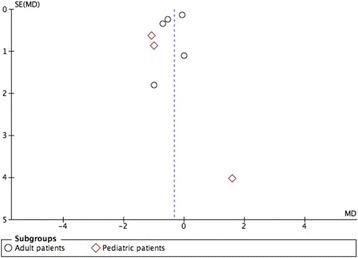
Funnel plot of ICU stay outcome for comparison nitric oxide versus all comparato

## P155 Preoperative beta-blocker & vasodilator combined with neuraxial block increase risk of perioperative cardiac complication in patients admitting to the general surgical ICU

### S Kongsayreepong, R Sungsiri, P Wongsripunetit

#### Siriraj Hospital, Mahidol University, Bangkok, Thailand

**Introduction:** Increase serious cardiac complications in patient receiving preoperative high dose beta-blocker and neuraxial block1. Anyhow with the perioperative benefit of neuraxial block especially the combined thoracic epidural and general anesthesia that could reduce stress & decrease ventilator hours including ICU length of stay. So questions were asked which could be the causes of these serious cardiac complication associated with neuraxial block. So the aim of this study was to study factors associated with perioperative cardiac morbidity in sick surgical patient receiving neuraxial block admitting to the general surgical ICU.

**Methods:** This was a part of prospective ICU database of general surgical ICU of Siriraj Hospital, Mahidol Univeristy. The data from 80 surgical patients receiving neuraxial block admitting to this general surgical ICU postoperative & stay in ICU > 24 hours. Studied information included: age, sex, BW, BMI, co-morbidity (stroke, TIA, coronary artery disease, NewYork Heart Classification, arterial vascular disease, DM), ASA classification, preoperative medication (beta-adrenergic blocking agent, vasodilator, ACEI), neuraxial block technique, preoperative hydration, type & duration of anesthesia/surgery, ventilator hours & ICU length of stay. Perioperative cardiac complications included intraoperative hypotension (MAP < 65 mmHg > 25 mins that needed fluid resuscitation and/or vasopressor), early perioperative myocardial ischemia/infarction, early perioperative arrhythmias, cardiac death. Neuraxial block in this study included: spinal, combined lumbar or thoracic epidural anesthesia.

**Results:** The result of this study showed that perioperative cardiac complications that associated with neuraxial block significant associated with preoperative beta-blocking agent, vasodilator, hypovolemia, high dose local anesthetic agent, high level thoracic epidural combined with general anesthesia, not using continuous technique, poor cardiac function (EF < 40%) (p < 0.05). As the sample size was not enough to do the multivariate analysis, so this part of the result was not be reported.

**Conclusions:** Despite the benefit of perioperative use of neuraxial block. Caution should be taken into consideration when using this neuraxial block in combind with perioperative beta-adrenergic blocking agent, vasodilator or in dehydration condition to prevent serious cardiac complications associated with this neuraxial block.

**References**

Leslie K, et al. 2013

## P156 Levamisole, a cocaine adulterant, potentiates the contractile response to endothelin-1 in rabbit carotid artery

### P Marchio, S Guerra-Ojeda, M Gimeno-Raga, MD Mauricio, SL Valles, C Aldasoro, A Jorda, M Aldasoro, JM Vila

#### University of Valencia, Valencia, Spain

**Introduction:** Acute vascular complications of cocaine abuse are mainly mediated by the inhibition of presynaptic reuptake of noradrenaline (NE), exacerbating the sympathetic response. In addition, cocaine exerts vasoconstrictive effects by stimulating α1-adrenergic receptors in arterial smooth muscle, and by decreasing nitric oxide and increasing endothelin-1 (ET-1) plasma levels [1]. Levamisole, an antihelminthic drug limited to veterinary use for its adverse effects in humans such as agranulocytosis and vasculitis, is currently used as cocaine adulterant [2]. Levamisole blocks the reuptake of NE [3, 4], but its effects on ET-1 are unknown. Thus, the purpose of this work was to evaluate the direct effects of levamisole and cocaine on contractile response to ET-1 on rabbit carotid artery.

**Methods:** Rabbit carotid rings were mounted for isometric tension recording in organ baths containing Krebs-Henseleit solution. Concentration-response curves to ET-1 (10^−11^-10^−7^M) were obtained in the absence and presence of levamisole (10^−5^-10^−3^M), cocaine (10^−5^-10^−4^M) and the combination of both drugs. To determine which receptor subtype was involved in ET-1 response, a second set of experiments were performed in the presence of ET-1 selective blockers BQ-123 (3 × 10^−7^M) for ETA and BQ-788 (10^−6^M) for ETB in the absence and presence of levamisole (10^−3^M).

**Results:** ET-1 produced a concentration-dependent contraction that was reduced in the presence of BQ123 (pD2 = 8.3 ± 0.1 vs 7.5 ± 0.1, p < 0.05), indicating that the contraction is mediated by activation of ETA receptors. Cocaine (10^−4^M) did not modify the maximal contractile response to ET-1 (Emax = 130 ± 3 vs 130 ± 11%, p > 0.05) whereas levamisole (10^−3^M) enhanced it (Emax = 130 ± 3 vs 152 ± 6%, p < 0.05). BQ123 prevented the potentiation of ET-1 response induced by levamisole. Co-incubation of cocaine (10^−4^M) and levamisole (10^−3^M) did not modify the potentiation induced by levamisole on ET-1 curve.

**Conclusions:** Levamisole exerts direct effects on vascular tone in the carotid artery, enhancing the contractile response to ET-1 through the activation of ETA receptors. This effect is independent of cocaine actions, suggesting that levamisole could potentiate the deleterious effects of cocaine on vascular tone.

**References**

[1] Zimmerman JL. Crit Care Clin. 28:517–26, 2012.

[2] Larocque A et al. Clin Toxicol. 50:231–41, 2012.

[3] Vanhoutte PM et al. J Pharmacol Exp Ther. 200:127–40, 1977.

[4] Hofmaier T et al. Neurochem Int. 73:32–41, 2014.

## P157 Effect of volume vs. vasoconstriction on regional oxygen saturation during hemodynamic management

### UB Borg, AM Neitenbach

#### Medtronic, Boulder, CO, United States

**Introduction:** The objective of this animal study was to determine effects of volume infusion versus vasoconstriction on regional oxygen saturation. Near infrared technology (NIRS) is used in cardiac surgery to monitor cerebral oxygen saturation (CrSO2) as an indicator of adequate perfusion. The most common intervention in reaction to decreasing CrSO2 is to increase mean arterial blood pressure (MAP). The question is whether volume or vasoactive drugs is the correct path. The chosen path may influence the incidence of organ insufficiency such as acute kidney injury. Organ perfusion, other than brain is not easily assessed with INVOS in adults, and may be compromised

**Methods:** In a porcine study, approved by the local animal use committee, we investigated the effect on CrSO2 and perirenal rSO2 (RrSO2) (INVOS™ 5100C, Medtronic, Boulder, USA) of restoring MAP with volume or vasoconstriction. Seventeen animals were put under general anesthesia, intubated and ventilated to normocapnia. MAP, heart rate, cardiac output CrSO2 and RrSO2 were continuously monitored. Hypovolemic state was achieved by removing 50% of the animals calculated blood volume. The shed blood or vasoconstriction using norepinephrine (NE) was used to return CrSO2 to baseline values. The shed blood was returned to the animal in steps of 200 ml/step and NE infusion was stepwise increased until CrSO2 was returned to baseline.

**Results:** Table 21 show mean values and SD for baseline, hypovolemia, effect of blood infusion or NE. Both blood infusion and NE returned the CrSO2 to baseline. Only blood infusion returned RrSO2 to baseline and there was a significant difference (p < 0.0002) between RrSO2 blood vs. NE even though a significantly (p < 0.0001) higher MAP was restored. A significantly (p < 0.0001) higher MAP was required to reach baseline CrSO2 with NE.

**Conclusions:** NIRS monitoring is often used in an attempt to monitor and prevent oxygen desaturation of the brain. In this study we demonstrated that restoring CrSO2 may result in unintended consequences for other organs. When CrSO2 was restored using NE the RrSO2 showed no improvement thus possibly indicating that the renal perfusion may have been compromised despite “normal” CrSO2 and MAP. The clinical implications of these results should be elucidated by clinical studies in patients.

**Table 21 (abstract P157).** Effect on regional oxygen saturation

	CrSO2 (%)	RrSO2 (%)	MAP (mm Hg)
Baseline values	59.9 ± 5.4	62.8 ± 8.6	74.8 ± 10.7
Hypovolemia	44.8 ± 5.8	57.0 ± 9.6	38.3 ± 8.7
After blood infusion	56.1 ± 6.6	63.4 ± 8.2	75.6 ± 8.4
After NE	59.4 ± 6.4	57.4 ± 4.9	90.2 ± 8.0

## P158 Dynamic arterial elastance reflects pressure-flow uncoupling in an experimental endotoxic septic shock

### M García^1^, P Guijo González^2^, M Gracia Romero^2^, P Saludes Orduña^2^, A Gil Cano^2^, A Rhodes^1^, RM Grounds^1^, M Cecconi^1^

#### ^1^St. George’s Healthcare NHS Trust and St George’s University of London, London, United Kingdom,^2^Hospital SAS de Jerez, Jerez de la Frontera, Spain

**Introduction:** Dynamic arterial elastance (Eadyn), the ratio between pulse pressure variation (PPV) and stroke volume variation (SVV), has been suggested as a functional parameter of arterial load.

We aimed to determine the effects of an endotoxic septic shock on Eadyn and its components (PPV and SVV), and the impact of hemodynamic resuscitation, considering both macro (arterial and cardiac factors) and microcirculatory influences, and the relationship with cardiac energetics.

**Methods:** 18 New Zealand rabbits. Animals received placebo (SHAM, n = 6) or lipopolysaccharide (LPS) with or without (EDX-R, n = 6; EDX-NR, n = 6) hemodynamic resuscitation (fluids and norepinephrine). Animals were monitored with an indwelling arterial catheter and an esophageal Doppler. The arterial load was evaluated by a 3-element Windkessel model. Cardiac influence on Eadyn was assessed by heart rate and the profile of the aortic blood flow. External work generated by the heart is the sum of the pressure energy (PE) and kinetic energy (KE), and represents the driving force for the circulation. Tissue oxygen saturation (StO2) was assessed using near-infrared spectroscopy

**Fig. 64 (abstract P158).**
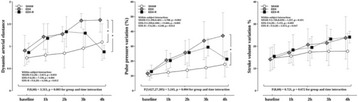
See text for description

**Results:** LPS infusion resulted in a hyperdynamic profile with an increase in CO and a reduction in blood pressure and arterial load. LPS administration led to a sustained decrease in StO2, while resuscitation produced a modest increase. Eadyn, PPV and SVV increased in both EDX and EDX-NR groups, but not in SHAM animals (Fig. 64). Even if PPV and SVV were related to arterial and cardiac factors, only blood flow pattern (velocity and ejection time) were associated with Eadyn. Cardiac energetics were associated with Eadyn. So, the higher the percentage of work performed by the heart to accelerate blood flow (KE), or the lower the ability to produce PE, the higher the Eadyn.

**Conclusions:** In this experimental model, Eadyn reflected the pressure-flow impairment during endotoxic septic shock. Hemodynamic resuscitation restored this relationship but a higher cardiac energetic cost. Eadyn could be a potential index of ventriculo-arterial coupling.

## P159 Validation of hypotension probability algorithm on university of California, Irvine surgical patients

### C Lee^1^, F Hatib^1^, Z Jian^1^, J Rinehart^2^, J De Los Santos^2^, C Canales^2^, M Cannesson^3^

#### ^1^Edwards Lifesciences, Irvine, CA, United States,^2^UC Irvine School of Medicine, Irvine, CA, United States,^3^UCLA David Geffen School of Medicine, CA, Los Angeles, United States

**Introduction:** Patients in surgical settings are often at risk of developing hypotension, which can lead to poor outcomes such as acute kidney and myocardial injury [1]. We have developed a hypotension probability indicator (P(↓BP)) for real-time prediction of intraoperative hypotension using features of the arterial pressure waveform. We assessed the performance of P(↓BP) on independent data being collected at UCI Medical Center (UCIMC).

**Methods:** We studied patients undergoing surgery for a wide range of procedures, including spinal, abdominal, and cardiac. 993 patients consented to this study, and 155 patients with arterial lines were analyzed to assess the accuracy of P(↓BP). A hypotensive event was defined as any time period where MAP < 65 mmHg for > = 1 minute. An ROC analysis was performed to assess AUC of P(↓BP) to identify and predict an event. Time to event, P(↓BP) and MAP values when P(↓BP) > 51 prior to event were also assessed. Clinical records were reviewed for any vasopressor, inotrope, crystalloid, colloid, or blood product interventions within 10 minutes before or after the start of an event.

**Fig. 65 (abstract P159).**
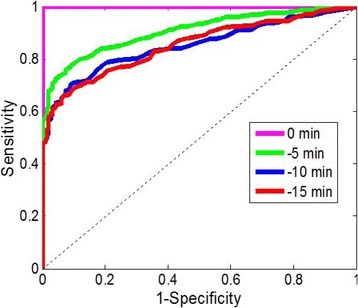
ROC curves for P(↓BP). AUC at 0 min = 1; −5 min = 0.91; −10 min = 0.85; −15 min = 0.85

**Results:** 123 of the 155 patients had at least one hypotensive event, totaling 12,212 hypotensive events. P(↓BP) accurately detected an event up to 15 minutes prior to event start (Fig. 65). In addition, using a threshold of 51, P (↓BP) warned of hypotension ~20 minutes prior (Table 22). Of all hypotensive events, only 5,665 (46.4%) events were treated. An intervention was started −0.5 [−2.5–1.8] from start of an event, a value < 0 indicating prior to event. Of the treated events, 3516 (62.1%) were with vasopressor, 1061 (18.8%) with crystalloid, 568 (10.0%) with colloid, 261 (4.6%) with blood, and 259 (4.6%) with inotrope.

**Conclusions:** P(↓BP) is capable of early detection of hypotensive events in independent validation data.

**References**

[1] Salmasi V et al. Anesthesiology 126:00–00, 2017

**Table 22 (abstract P159).** Table of P(↓BP) time to event and P(↓BP) and MAP values as median [IQ]

Threshold	Time to Event (min)	P(↓BP) at Threshold Crossing (%)	MAP at Threshold Crossing (mmHg)
P(↓BP) ≥ 51	19.7 [8–62.7]	61 [56–91]	72.7 [68–76.2]
P(↓BP) = 100	0 [0–0]	100 [100–100]	59.2 [55.5–61.9]

## P160 Pre-emptive protocol based on hypotension probability indicator: preliminary clinical results

### MI Monge García

#### Hospital SAS de Jerez, Jerez de la Frontera, Spain

**Introduction:** Hypotension often represents the first sign of an acute decompensated cardiovascular system that precedes organ hypoperfusion. Thus, predicting arterial hypotension could be a relevant aspect in hemodynamic management of critically-ill patients. In this regard, a new hemodynamic parameter based on the analysis of the arterial pressure waveform features has been proposed to predict hypotensive episodes: hypotension probability indicator (HPI). The aim of this study was to report the preliminary results of the application in real clinical practice of the performance of the HPI™ for predicting arterial hypotension.

**Methods:** Patients had an indwelling radial arterial catheter connected to a FloTracIQ™ sensor (Edwards Lifesciences, Irvine, CA). A hypotensive episode was defined as MAP < 65 mmHg for at least 1 minute. ROC curve analysis was performed to determine sensitivity/specificity and the area under the ROC curve of the HPI to predict a hypotensive event 5 minutes prior to the start of event.

**Fig. 66 (abstract P160).**
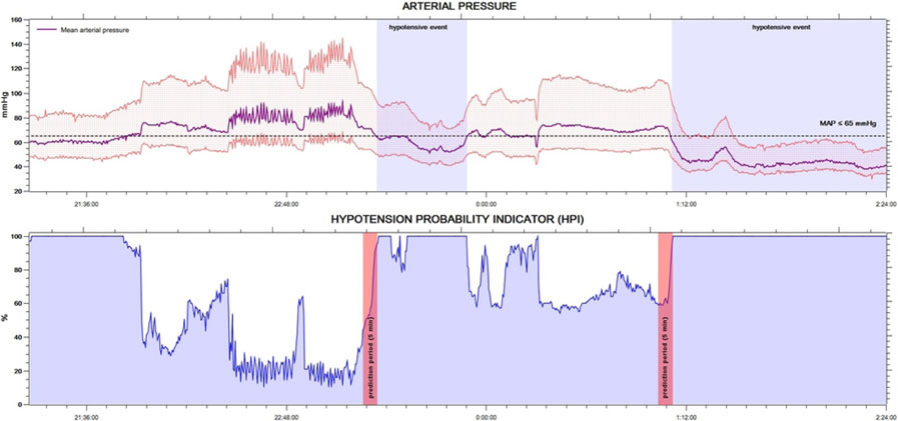
Example of evolution of arterial pressure and HPI in one patient

**Fig. 67 (abstract P160).**
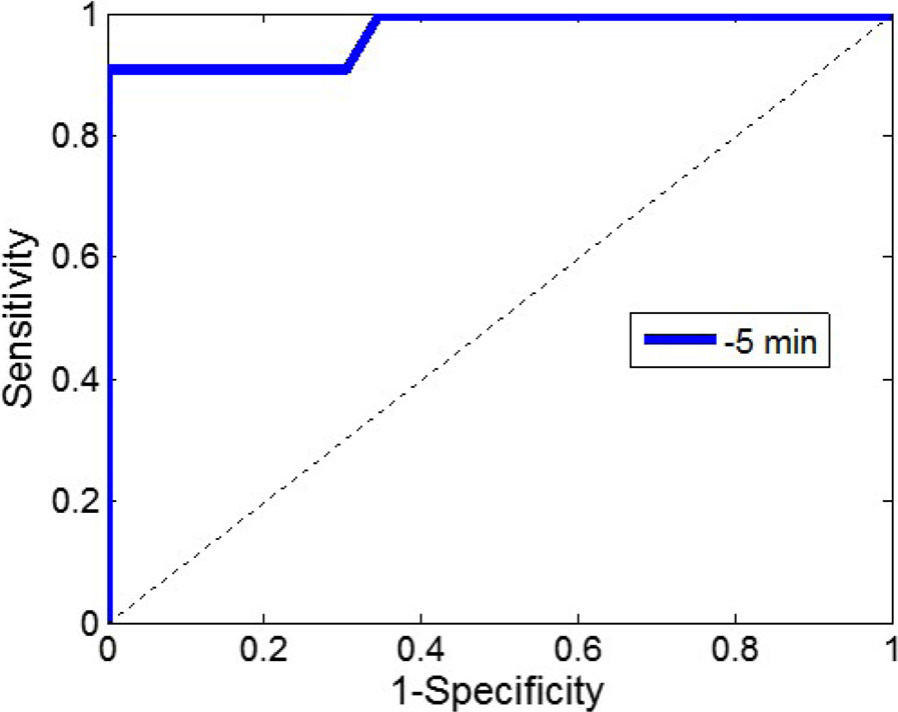
ROC curve for testing the ability of HPI to predict a hypotensive event

**Results:** Six patients (3 surgical, 1 blunt chest trauma, 1 intracerebral haemorrhage and 1 variceal upper gastrointestinal bleeding) were evaluated (3 Female, 3 Male, 45 ± 15 years, 77 ± 15 kg, 168 ± 10 cm). There were 21 hypotensive episodes (Fig. 66). On average, each patient had 3.5 ± 4.5 episodes and each episode had a duration of 10 ± 20 minutes. The average monitoring time was 835 ± 732 minutes. Three patients (50%) have at least one hypotensive episode. Each patient was in hypotension for 36 ± 61 minutes, representing 9.5 ± 19% of monitoring time. ROC analysis shows HPI was able to predict hypotension with a sensitivity of 91% and specificity of 91%, at 5 minutes prior to hypotensive episodes. The AUC for HPI was 0.97 (Fig. 67).

**Conclusions:** Although further confirmation with a larger sample size is required, our preliminary results showed that HPI predicted hypotension with high reliability in real clinical practice and could be a valuable information during hemodynamic management of critically-ill patients.

## P161 Prediction of hypotension in porcine model

### F Hatib^1^, Z Jian^1^, T Scheeren^2^

#### ^1^Edwards Lifesciences, Irvine, CA, United States; ^2^University Medical Center Groningen, Groningen, Netherlands

**Introduction:** Hypotension occurs frequently in intraoperative and critical care settings and is associated with an increased incidence of complications. We have recently developed a hypotension probability indicator (HPI™) to predict hypotension based on machine learning techniques. The objective here is to test the accuracy of HPI in predicting artificially induced hypotension in a porcine model.

**Fig. 68 (abstract P161).**
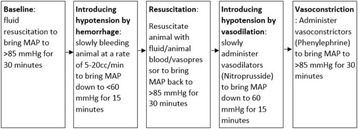
Animal study protocol flow chart

**Methods:** With IRB approval, hypotension is induced in animals both by hemorrhage and by vasodilation using the protocol described in fig. 68. Radial arterial pressure waveforms were monitored invasively by FloTrac (Edwards Lifesciences) to calculate HPI. ROC analysis was performed to assess the performance of HPI.

**Fig. 69 (abstract P161).**
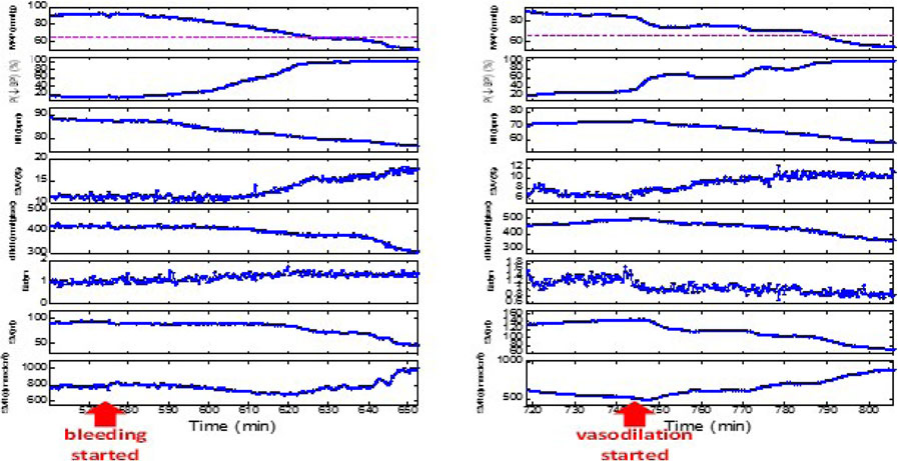
Examples of hemodynamics in hemorrhage (left) and vasodilation models (right)

**Results:** 5 pigs were studied. 12 hypotensive episodes occurred: 6 by hemorrhage and 6 by vasodilation. Typical examples of animal’s hemodynamic variables are shown in fig. 69: HPI increases with the introduction of hypotension, indicating there is higher probability for hypotension to occur. ROC analysis shows HPI can predict hypotension with a sensitivity and specificity of 100% and 100%, respectively, at 5 minutes prior to hypotensive episodes. The area under the curve is 1. Changes can be found in stroke volume variation as a preload measure, dynamic arterial elastance as a functional measure of afterload, and dP/dt as a measure of LV contractility.

**Conclusions:** Our study suggests that HPI can predict both hemorrhage and vasodilation induced hypotension 5 minutes before its occurrence with high sensitivity and specificity. Information about preload, contractility or afterload may help to take appropriate therapeutic measures.

## P162 Prevalence of hypotension and prediction of hypotension in intensive care unit

### Z Jian^1^, F Hatib^1^, M Pinsky^2^

#### ^1^Edwards Lifesciences, Irvine, CA, United States; ^2^University of Pittsburgh, Pittsburgh, PA, United States

**Introduction:** Critically ill patients are often at risk of developing hypotension, which could lead to end organ ischemia/dysfunction, increased morbidity and mortality. The objective here is two-fold: 1) to determine the prevalence of hypotension in ICU patients, defined as mean arterial pressure (MAP) < 65 mmHg for at least 1 minute; and 2) to test the accuracy of HPI™ parameter in predicting hypotension. HPI is a hypotension probability indicator Edwards Lifesciences recently developed based on machine learning techniques.

**Methods:** Patient data were randomly selected from the MIMIC II Database and a proprietary Edwards clinical Database. Radial arterial pressure waveforms were passed through FloTrac algorithm (Edwards Lifesciences) to calculate MAP and HPI. The MAP was then used for the evaluation of hypotension prevalence, while an ROC analysis was performed to assess the performance of HPI to predict hypotension.

**Results:** 753 patients were evaluated (254 Female, 499 Male, 63 ± 15 years, 82 ± 23 kg, 170 ± 10 cm), including 181 MIMIC II patients (69% cardiac recovery and care unit; 28% medical; 3% medical + surgical), and 572 Edwards patients (36% sepsis; 9% cardiogenic; 26% cardiac; 10% liver transplant; 19% others).

The average monitoring time is 57 ± 57 hours. 632 (or 84%) patients have at least one hypotensive episode. On average each patient was in hypotension for 671 ± 1549 minutes, representing 18 ± 25% of monitoring time. There are 34,271 hypotensive episodes, on average each patient has 45 ± 67 episodes and each episode has a duration of 15 ± 186 minutes.

**Fig. 70 (abstract P162).**
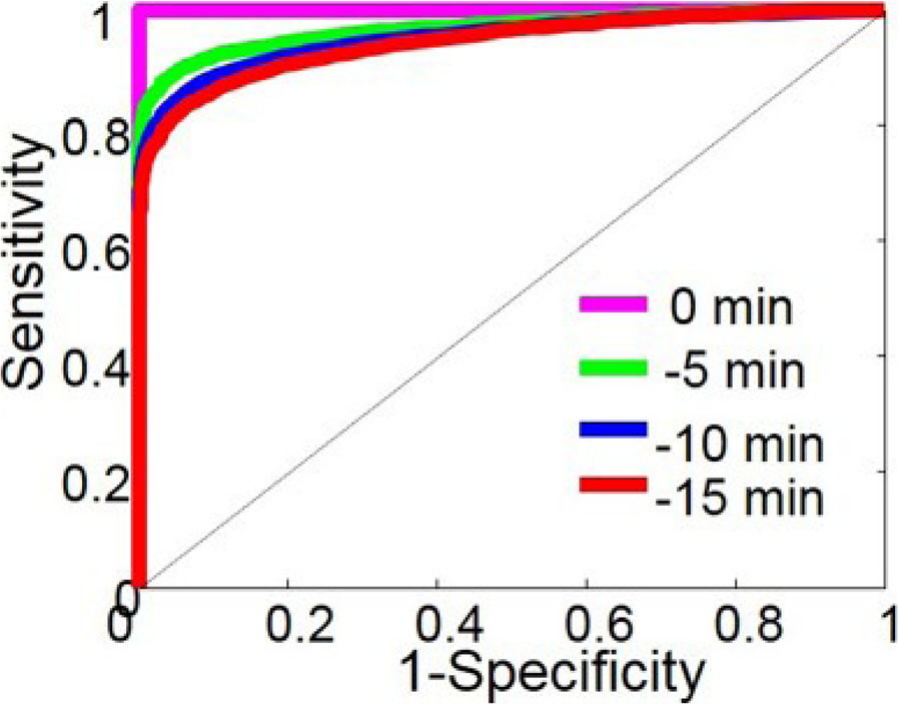
ROC curve for HPI to predict hypotension

ROC analysis shows HPI was able to predict hypotension with a sensitivity/specificity of 91%/92%, 89%/89%, and 88%/87%, at 5, 10, and 15 minutes prior to hypotensive episodes, respectively. The corresponding area under the curve is 0.97, 0.95, and 0.94, respectively (Fig. 70).

**Conclusions:** Those data demonstrate that hypotension occurs quite often in ICU patients, and HPI can predict hypotension with high sensitivity and specificity. HPI may serve as a useful addition in the care of critically ill patients with the potential to reduce the occurrence of hypotension.

## P163 A novel method of ultrasonic assessment of bilateral radial artery stiffness characteristics

### V Chantziara^1^, A Vassi^1^, G Michaloudis^1^, E Sanidas^2^, S Golemati^3^

#### ^1^Saint Savvas Hospital, Athens, Greece; ^2^Cardiology Dep, Laiko General Hospital, Athens, Greece; ^3^Medical School, National Kapodistrian University of Athens, Athens, Greece

**Introduction:** The radial artery is often catheterized in the intensive care unit (ICU), allowing for direct measurement of local blood pressure. Along with ultrasonic estimates of arterial displacements, such measurements can be used to estimate local tissue stiffness and its changes in different conditions. However, because of the presence of the catheter, ultrasound and pressure measurements cannot be made on the same side.

**Methods:** Image sequences of left and right radial arteries of 5 normotensive subjects (38.5 ± 3.10 yo) were acquired at ~30Hz using B-mode ultrasound and a linear array transducer. Arterial diameters were derived from displacement waveforms produced from image sequences using adaptive block matching. Diastolic and systolic blood pressures were measured at the brachial artery using sphygmomanometry. Tissue properties were quantified through radial strain, distensibility and compliance coefficients and Peterson’s elastic modulus (Table 23).

**Fig. 71 (abstract P163).**
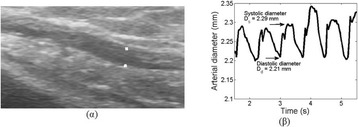
See text for description

**Results:** Figure 71 shows an ultrasound image of the radial artery and its diameter waveform. Table 23 shows average ± std values for the estimated parameters. Tissue stiffness of the left side was similar to that of the right, based on a Wilcoxon rank sum test (p- value > 0.05). The observed large variabilities were attributed to intersubject anatomical differences reflected in different diameters and blood pressures.

**Conclusions:** These preliminary findings suggest that blood pressure measurements in one radial artery can be used to reliably derive stiffness indices in the contralateral side.

**Table 23 (abstract P163).** Average ± std values for diastolic (*D*_*d*_) and systolic (*D*_*s*_) diameters, radial, strain (ε)

	Left side	Right side	*p*-value
***D***_***d***_, mm	1.66 ± 0.54	1.63 ± 0.67	0.94
***D***_***s***_, mm	1.73 ± 0.53	1.72 ± 0.69	0.96
$\varepsilon$, %	5.04 ± 3.67	5.68 ± 3.94	0.78
***DC***, kPa^−1^*10^−3^	21.90 ± 17.88	19.74 ± 11.24	0.81
***CC***, m^2^/kPa*10^−3^	5.60E-6 ± 4.56E-6	6.06E-6 ± 6.43E-6	0.89
***E***_***P***_, kPa	87.18 ± 76.28	72.13 ± 57.31	0.71

$\varepsilon =\frac{D_s-{D}_d}{D_d},\kern0.5em  D C=\frac{A_s-{A}_d}{A_s\cdotp PP},\kern0.5em  C C=\frac{A_s-{A}_d}{ P P},{E}_P=\frac{A_{avg}\cdotp PP}{A_s-{A}_d}$ where *A*_*s*_ denotes lumen area at systole, *A*_*d*_ lumen area at diastole, *A*_*avg*_ average lumen area, and PP pulse pressure

## P164 Non-invasive measurement of arterial flow velocity in the dorsalis pedis artery under control and septic conditions

### RM Bateman

#### University of Western Ontario, London, Canada

**Introduction:** Non-invasive measurement of hemodynamic parameters and microvascular reactivity are increasingly reported and recommended for assessment of critically ill patients [1–4]. In this proof of concept study, objectives were 1) assess non-invasive measurement of peripheral arterial flow velocity in the Dorsalis Pedis artery using a novel ultrasound sensor [5], 2) analyze velocity waveforms and 3) assess response under a variety of control and pathological conditions including 1) resting control state, 2) Valsalva maneuver, 3) hyperemic response and 4) in active sepsis with norepinephrine treatment.

**Methods:** In age matched male subjects in the basal position (135 degrees), the sensor was positioned above the left Dorsalis Pedis artery.

**Fig. 72 (abstract P164).**
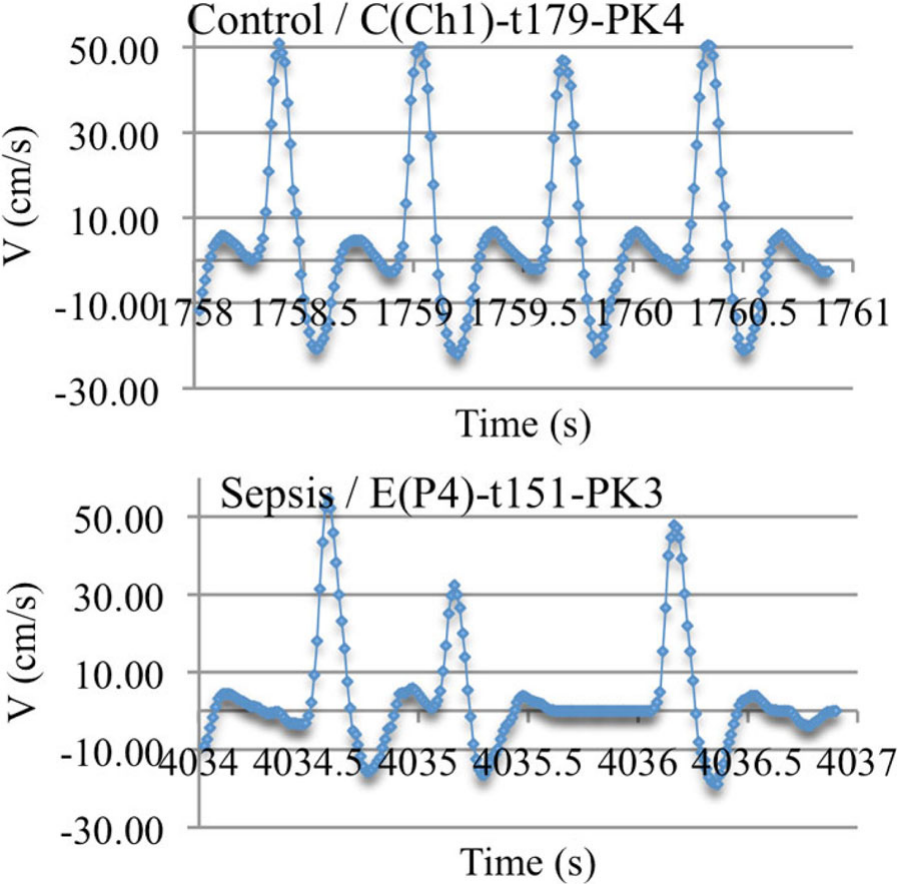
See text for description

**Fig. 73 (abstract P164).**
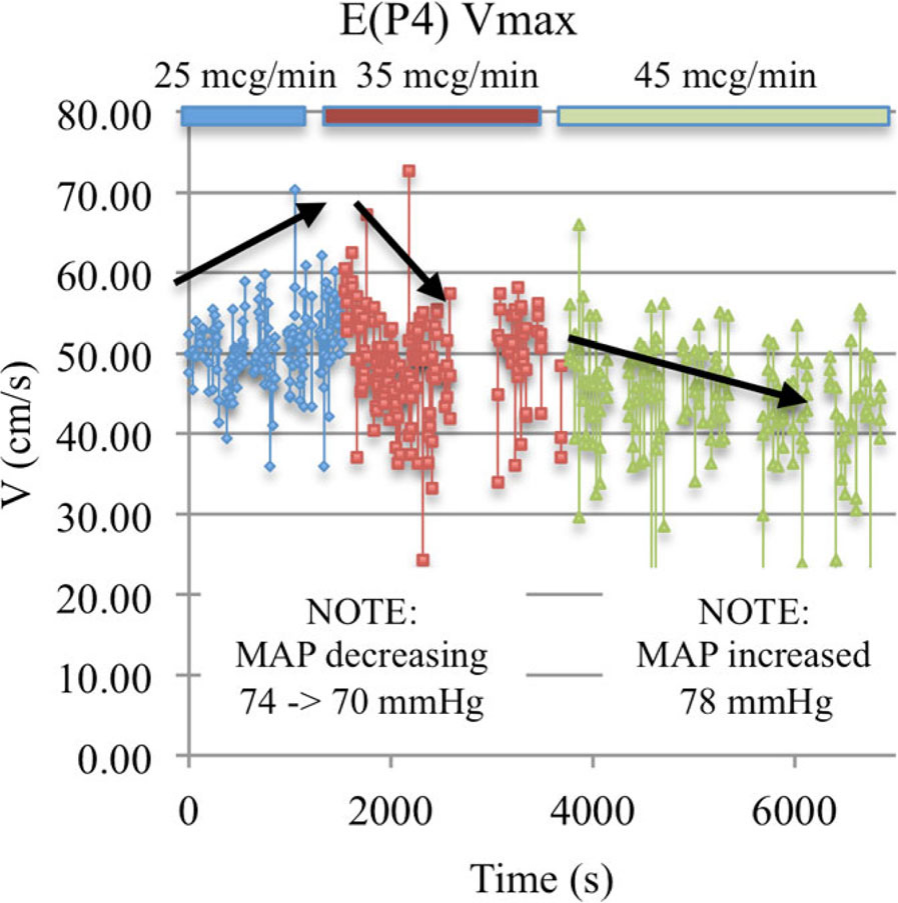
See text for description

**Results:** Figure 72 shows velocity waveforms from control and septic patient. Waveforms depict forward flow velocity (max peak), reverse flow velocity (min peak) and additional reflected waves, originating from “blind ends” or arterioles and capillaries of microvascular beds. Note the increased variability in max velocity in sepsis (range from 32.4 to 54.7 cm/s) compared to control (range from 46.9 to 50.78 cm/s)) and prolonged stasis (40% of time) between 2nd and 3rd max peaks. Figure 73 reveals norepinephrine had a rapid effect on arterial flow velocity. As norepinephrine increased from 25 to 35 mcg/min there was a rapid decrease in max velocity over 15 minutes. Increasing the dose to 45 mcg/min resulted in a further decrease in arterial velocity over 10 minutes. This was associated with increased MAP. In control experiments (see poster) transient responses in max flow velocity in the Dorsalis Pedis artery were detected in response to 20s Valsalva maneuver, 50s vascular occlusion and to passive leg raising.

**Conclusions:** Non-invasive peripheral arterial flow velocity, measured in the Dorsalis Pedis artery using an ultrasound sensor, is sensitive to a variety of physiological transient responses and to the effects of norepinephrine in sepsis. Taken together, peripheral arterial flow velocity is an additional hemodynamic parameter which may have broader application in terms of patient monitoring, fluid resuscitation and vasopressor evaluation, inter alia, though further validation is required.

Acknowledgements: The author thanks Dr. David Vilkomerson for use of the ultrasound sensor and help with experimental design, Arterium Medical for funding and staff at Erasme Hospital.

**References**

1. Donati A et al., Crit Care 2016, 20(1):311.

2. Orbegozo Cortes D et al., Respir Res 2016, 17(1):59.

3. Cecconi M et al., Intensive Care Med 2014, 40(12):1795–1815.

4. Vincent JL et al., N Engl J Med 2013, 369(18):1726–1734.

5. Vilkomerson D et al., IEEE Trans Ultrason Ferroelectr Freq Control 2013, 60(10):2079–2088.

## P165 Predictive value of measuring brachial artery reactivity in septic patients

### A Mokhtar^1^, W Omar^1^, K Abdel Aziz^2^, H El Azizy^2^

#### ^1^Salam International Hospital, Cairo, Egypt,^2^Cairo University Hospital, Cairo, Egypt

**Introduction:** Endothelial function alterations play a major role in the pathophysiology of septic shock and associated organ dysfunction and has been suggested to predict mortality in sepsis. Non-invasive assessment of endothelial function by measuring flow mediated dilatation and velocity of brachial artery by ultrasound in response to induced ischemia can detect endothelial dysfunction and may therefore directly correlate with outcome of patients with sepsis as it directly reflects the severity of illness. Ultrasound measurements of brachial artery are as efficient as sepsis-related markers in assessing severity of illness and therefore outcome. The aim of this study was to assess whether ultrasound measurement of brachial artery reactivity correlates with outcome of patients with sepsis or not and to compare its prognostic value with biomarkers (CRP, microalbuminuria).

**Methods:** 50 Septic patients were subjected to FMD of brachial artery within 24 hours from icu admission. Measurements were compared in sepsis survivors versus non survivors. Microalbuminuria and CRP values were obtained also for patients that fulfill criteria of sepsis.

**Results:** Hyperemic velocity was significantly lower in Sepsis group (71.77 ± 18.67 vs 81.64 ± 9.84, p 0.003) vs control. FMD % was significantly lower in sepsis group (3.72 ± 2.22 vs 5.29. ±1.74, p 0.001) vs control group Change in Velocity was significantly lower in sepsis group(19.53 ± 10.80 vs 31.25 ± 6.72, p <0.001) vs control group. 10/50 (20%) of enrolled patients died within 28 days of hospital admission. Change in Velocity was significantly lower in non survivors (7.82 ± 2.26 vs 22.45 ± 10.081 p = 0.001) vs survivors respectively. Microalbuminuria was significantly higher in non survivors (54.00 ± 19.24 vs 30.33 ± 44.92 p 0.006) vs survivors.

**Conclusions:** Ultrasound measurements of brachial artery hyperemic change in blood velocity is an easily available bedside method of assessing endothelial function alterations and can predict mortality in sepsis as it directly reflects the severity of illness. Brachial artery reactivity measurement is therefore an effective and easy tool in assessing severity of illness and therefore predicting outcome in septic patients and is as efficient as other sepsis related biomarkers. Microalbuminuria directly correlates with severity of illness and therefore outcome in septic patients.

## P166 (How) do patients with different shock etiologies differ in clinical presentation at arrival to the emergency department?

### DL Lykke Nielsen, JG Holler, A Lassen

#### Odense University Hospital, Odense C, Denmark

**Introduction:** Shock has been shown to have an in-hospital mortality as high as 33–52%. Early identification of the etiology improves prognosis, but the clinical picture is often obscured by altered mental status and comorbidities. The aim of this study is to describe clinical presentation, at arrival to the Emergency Department (ED), of patients with cardiogenic, septic or hemorrhagic shock.

**Methods:** We conducted a retrospective cohort study of all adult (Age > =18 years) patients arriving to the ED at Odense University Hospital between 2000 and 2011. Shock was defined as systolic blood pressure (SBT) < = 100 mmHg at arrival and > =1 organ failure. The three etiological groups were defined as: Cardiogenic shock: Patients with discharge diagnosis indicating a cardiogenic cause. Hemorrhagic shock: Patients with blood transfusion and a discharge diagnosis indicating bleeding. Septic shock: Patients with discharge diagnosis of infection or sepsis who also had blood cultures made at arrival, and patients with positive blood cultures from the two other groups. Analyzed variables presented at arrival to the ED: Age, heart rate (HR), SBT, diastolic blood pressure (DBP), Charlson Comorbidity Index (CCI), Creatinine, bilirubin, platelets, International Normalized Ratio (INR), Coagulation Factors, hemoglobin (Hb), c-reactive protein (CRP), temperature (Tp), alanine transaminase (ALAT), albumin, carbamide, potassium, sodium, troponin t, and leucocytes including neutrophils, basophils, eosinophils, lymphocytes and monocytes. Not all variables were measured on all patients. Analysis was done with a k-sample equality-of-median test, STATA v 14.0.

**Results:** We included 341 with septic, 226 with cardiogenic and 171 with hemorrhagic shock; their median age was 74 years, 76 years, and 71 years (p = 0.104), and 55%, 54% and 65% were male (p = 0.239), their median CCI was 2, 2, 1 (p = 0.064), respectively.

There were significant differences in median CRP: 133 mg/L, 20 mg/L, 13.5 mg/L (p < 0.001), Tp: 37.4°, 36.6°, 36 ° (p < 0.001), median creatinine: 154 μmol/L, 135 μmol/L, 112 μmol/L (p < 0.001), median troponin t: 0.06 μg/L, 0.07 μg/L, 0.01 μg/L (p = 0.001), median bilirubin: 11 μmol/L, 11.5 μmol/L, 7 μmol/L (p < 0.001), median ALAT: 25U/L, 28U/L, 17U/L (p = 0.002), median leucocytes: 14.2 × 10^9^/L, 10.9 × 10^9^/L, 12 × 10^9^/L (p < 0.001), median lymphocytes: 0.06 × 10^9^/LL 0.12 × 10^9^/L, 0.11 × 10^9^/L (p < 0.001), respectively. Significant differences were also found in DBT, carbamide, neutrophils and eosinophils

**Conclusions:** Patients who arrive to the ED with septic, cardiogenic or hemorrhagic shock differ in CRP and creatinine, and have other minor variations in clinical variables. The diagnostic value of these variables remains to be analyzed

## P167 Intraosseous administration of adrenaline does not impair uptake of a subsequent injection in hypovolemic shock

### M Eriksson^1^, G Strandberg^1^, M Lipcsey^1^, A Larsson^2^

#### ^1^Surgical Sciences, Uppsala, Sweden; ^2^Medical Sciences, Uppsala, Sweden

**Introduction:** Intraosseous (IO) catheterization is frequently used in medical emergencies, when venous access is difficult to achieve. It is proven to be a rapid way of establishing vascular access. Pediatric Advanced Life Support (PALS) and Advanced Trauma Life Support (ATLS) recommend placement of an IO line if adequate IV access cannot be quickly established. Use of IO adrenaline has been evaluated in a porcine model of cardiac arrest [1]. We decided to evaluate whether IO injection of adrenaline affects systemic uptake of a second drug, injected through the same IO needle in another shock model, where adrenaline may be administered i.e. hypovolemic shock.

**Methods:** Ten anaesthetized pigs were exsanguinated by 50% of the circulating blood volume. Adrenaline (n = 5) or saline (n = 5), respectively, were administered at a clinically relevant dose through a tibial IO needle (EZ-IO®, Teleflex Medical, Morrisville, NC, USA). A subsequent injection of a tracer substance (gentamicin at 7 mg/kg) was administered through the same IO needle. Central plasma concentrations were drawn at 5, 15 and 30 minutes after the IO injection of gentamicin. The concentrations of gentamicin were analyzed on an Architect Ci8200 analyzer. The total coefficients of variation for the gentamicin assay were 1.7% at 3.0 mg/L and 2.2% at 5.5 mg/L.

**Fig. 74 (abstract P167).**
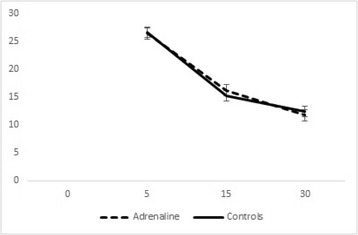
Tracer (gentamicin mg/L))

**Results:** After starting the endotoxin infusion, most of the animals showed signs of hemodynamic instability with reduced mean arterial blood pressure (MAP). As seen in Fig. 74, the concentration of the tracer (gentamicin), were nearly identical regardless of a preceding injection of adrenaline or not.

**Conclusions:** This study shows that IO injections of adrenaline, given according to CPR protocol, does not impair the uptake of a subsequent injection of gentamicin administered through the same IO needle in pigs subjected to hemorrhagic shock.

**References**

1. Wong et al. J Surg Res 201: 327–33, 2016

## P168 Vasopressin versus norepinephrine for the management of septic shock in cancer patients (vancs ii)

### C Capoletto, J Almeida, G Ferreira, J Fukushima, R Nakamura, S Risk, E Osawa, C Park, G Oliveira, F Galas, R Franco, L Hajjar

#### Cancer Institute of the University of Sao Paulo, Sao Paulo, Brazil

**Introduction:** Septic shock is a frequent and severe complication in oncologic patients, resulting in increasing number of intensive care unit (ICU) admissions, increased hospital costs and high mortality rates. Norepinephrine is the most frequently used vasopressor in this setting; however, approximately 40% of septic shock patients are refractory to this drug. Vasopressin is a non- catecholaminergic vasopressor commonly used as an adjunct to norepinephrine. The aim of this study is to evaluate the effect of vasopressin compared with norepinephrine in the mortality of cancer patients with septic shock admitted to the ICU.

**Methods:** In this single-center, prospective, randomized and double-blind study, 250 patients with cancer and septic shock were evaluated from July 2nd 2014 to July 2nd 2016 to randomly receive vasopressin (0.01 to 0.06 U/min) or norepinephrine (0.1 mcg/kg/min to 1.0 mcg/kg/min), and the infusion was titrated to maintain the target average blood pressure. Primary outcome was all-cause mortality within 28 days after randomization.

**Results:** Of 1125 patients analyzed for eligibility, 250 patients were included in the analysis to receive the study drug; 125 to receive vasopressin and 125 to receive norepinephrine.

There was no difference in 28-day mortality rate (52.8% in the norepinephrine group vs 56.8% in the vasopressin group, P = 0.525), as well as 90-day mortality (75.2% vs 72%, P = 0.566). New organ dysfunction occurred in 62.4% of norepinephrine group patients and in 73.6% of vasopressin group patients (p = 0.058). There were no significant differences between groups regarding cardiovascular, respiratory, neurological or hematologic complications, 28-day ICU stay, readmission to the ICU, dialysis-free days, ventilatory support, days free of vasopressor, and hospital length of stay.

**Conclusions:** Vasopressin compared to norepinephrine did not reduce mortality rates in cancer patients with septic shock admitted to the ICU.

Trial Registration: NCT01718613

**References**

1. Russell JA, Walley KR, Singer J, et al.; VASST Investigators. Vasopressin versus norepinephrine infusion in patients with septic shock. N Engl J Med. 2008;358(9):877–887.

2. Hajjar, LA, Vincent JL, Gomes Gallas FR, et al.; VANCS Investigators. Vasopressin versus norepinephrine in patients with vasoplegic shock after cardiac surgery: The VANCS randomized controlled trial. Anesteshiology. 2017: 126: 00–00.

## P169 Outcomes in septic shock patients requiring vasopressin

### F Dias, N D’Arrigo, F Fortuna, S Redaelli, L Zerman, L Becker

#### Hospital Pompeia, Caxias do Sul, Brazil

**Introduction:** Vasopressin (VP) has an important role in restoring blood pressure in septic shock patients. The aim of this study was to determine the outcome of patients with septic shock needing vasopressin to achive a MAP of 65 mm˙Hg despite NOR use.

**Methods:** Retrospective, observational, cohort study. The variables collected were: age, gender, SAPS 3, SOFA at admission, days on MV, need of RRT, LOS and, ICU mortality. Data were presented as percentages for cathegorical variables and means for continuous variables. Comparisons were made with the use of Student’s *T* test, Mann–Whitney *U* test, or Pearson Chi-square test as appropriate. To estimate the effects of vasopressin use on mortality, we used binary logistic regression with SAPS3, SOFA, and need for RRT as covariates. All statistical analysis were made using SPSS vs 20.0.

**Results:** There were 163 consecutive patients treated for septic shock, between January 2015 and August 2016, divided in group 1 (NOR plus VP; n = 54) and group 2 (NOR only; n = 109). The mean age (yr), male gender (%), days on MV, RRT (%) and LOS ICU was 58.8 and 62.00(NS), 61.1 and 52.3 (NS), 8.1 and 9.3 (NS), 24.1 and 11.9 (p < 0.001), 9.6 and 11.9 (NS), respectively. Severity and organ dysfunction score and, ICU mortality are in Table 24. After stepwise binary logistic regression modeling, vasopressin use was still significantly associated with ICU mortality, with an OR of 5,34 (2,41–11,87).

**Conclusions:** Patients needing vasopressin to sustain a MAP at least 65 mmHg had worst severity scores at admission, more severe organ dysfunction and needed more RRT during ICU stay. VP rescue therapy during septic shock had a high mortality in comparison to those who sustain MAP only with NOR even when adjusting for severity of disease.

**Table 24 (abstract P169).** Group characteristics

Variable	Group 1	Group 2	p value
SAPS 3	74.1	67.1	0.004
SAPS 3 SMR LA	71	60	0.005
SOFA admission	9.7	8.4	0.003
ICU mortality	77.8	35.8	<0.001

## P170 Cardiovascular and inflammatory effects of high-dose insulin infusion in a clinically relevant septic shock experimental model

### T Serrano, L Cotes, F Ramos, L Fadel, F Coelho, C Mendes, J Real, B Pedron, M Kuroki, E Costa, L Azevedo

#### Research and Education Institute, Hospital Sirio-Libanes, São Paulos, Brazil

**Introduction:** High-dose insulin (HDI) has hemodynamic and metabolic properties in several myocardial dysfunction scenarios. This study was carried out to evaluate if HDI infusion would improve hemodynamic, metabolic and inflammatory parameters in a clinically relevant septic shock model treated with fluid resuscitation and antibiotics.

**Methods:** Sixteen pigs (35–45 kg) were anesthetized, monitored with pressure-conductance catheters and submitted to peritonitis by fecal inoculation (1 g/kg). In the insulin group, dextrose (50%) was infused to maintain glucose in the 60–150 mg/dL range, and potassium to maintain a level greater than 2.8 mmol/L. After persistent hypotension, animals received antibiotics, fluids and vasopressors according to a predefined algorithm and were randomized to an insulin (HDI) (n = 9) or control (n = 7) group. The HDI group received an insulin infusion of 3 units/kg/hour during six hours. Inflammatory response was evaluated by plasma, myocardial and pulmonary concentration of cytokines (IL-6 and IL-8) by ELISA. Outcomes from the repeated-measures analysis were modeled using a mixed-effects linear model.

**Results:** After 6 hours of treatment, cardiovascular and metabolic variables were significantly better in the HDI group vs control: cardiac output (5.83 ± 0.76 vs 2.15 ± 0.52 L/min, p < 0.001), stroke volume (38.40 ± 9.55 vs 19.40 ± 4.51 mL, p < 0.05), systolic work (2.86 ± 0.93 vs 1.07 ± 0.31 (mL.mmHg)/kg.beat, p < 0.05), arterial elastance (2.65 ± 0.81 vs 4.21 ± 1.16 mmHg/mL, p < 0.05), dp/dtmax (4786 ± 630 vs 2363 ± 991 mmHg/s, p < 0.001), norepinephrine requirements (0.43 ± 0.49 vs 1.5 ± 1.7 mcg/kg/min, p < 0.05) and lactate concentrations (1.3 ± 1.1 vs 4.2 ± 1.8 mmol/L, p < 0.05). No differences in plasmatic, cardiac and pulmonary concentrations of IL-6 and IL-8 were found.

**Conclusions:** In this clinically relevant animal model of peritonitis-induced septic shock HDI is associated with improved cardiac function but does not modify inflammatory response. The beneficial cardiovascular effects of high-dose insulin infusion may be mediated by other pathophysiologic mechanisms.

