# Erratum to: P95 B-lines on chest ultrasound predicts elevated left ventricular diastolic pressures

**DOI:** 10.1186/s13054-017-1722-1

**Published:** 2017-07-11

**Authors:** Z. Bitar, O. Maadarani, R. Al Hamdan

**Affiliations:** 1 Hôpitaux universitaires Paris-Sud, Hôpital de Bicêtre, Inserm UMR S_999, Univ Paris-Sud, Le Kremlin-Bicêtre, France; 2 Zouheir bitar, Fahahil, Kuwait

## Erratum

After publication of this supplement abstract below in [1], it was brought to our attention that for abstract P95 all the authors should have the following affiliation: Internal Medicine, Ahmadi Hospital, Kuwait Oil Company, Ahmadi, Kuwait.

## P95 B-lines on chest ultrasound predicts elevated left ventricular diastolic pressures

### Z. Bitar^1^, O. Maadarani^2^, R. Al Hamdan^2^

#### ^1^Hôpitaux universitaires Paris-Sud, Hôpital de Bicêtre, Inserm UMR S_999, Univ Paris-Sud, Le Kremlin-Bicêtre, France; ^2^zouheir bitar, Fahahil, Kuwait

**Introduction:** We investigated the relationship between the ultrasonic B profiles and Spectral tissue Doppler echocardiography (E/E’ ratio), a non-invasive surrogate for left ventricular diastolic pressures, in patients presenting with suspicion of acute pulmonary edema.

**Methods:** This is a prospective observational study of 61 consecutive patients presenting with acute pulmonary edema and B - profile detected by echocardiography with a 5 MHz curvilinear probe. The Filling pressure of the left ventricle considered high when E/E’ is equal or > 15 or when value between 9 and 14 with ultrasound chest B pattern. The filling pressure is considered normal if E/E’ is equal or below 8 or the value between 9 and 14 with A-line pattern (1).

**Results:** Sixty-one participants were included (49.2% male, with a mean age 66.8). The mean E/E’ level in the patients with B-profile was (20.8), compared with the mean level in the patients with an A-profile of (8.2) (*p* = 0.003). Based on the value of E/E’, the sensitivity and specificity (including the 95% confidence interval) were determined and are shown in Table 13. The systolic function in the subjects with a B-profile was below 50% in 74.3% of the subjects. All the subjects with B profile and systolic function > 50% had elevated NT-proBNP and E/E’ > 15.

**Table 13:** Chest ultrasound profiles based Spectral tissue Doppler echocardiography E/E’

Thoracic ultrasound profile	High E/E’	Normal E/E’	Total
B- Profile	46	1	47
A -profile	4	10	14
Total	50	11	61
Variable	Value	95% confidence interval	
Sensitivity	0.92	0.812 to 0.968	
Specificity	0.91	0.623 to 0.98	
Positive predictive value	0.97	0.889 to 0.996	
Negative predictive value	0.714	0.454 to 0.883	

**Conclusions:** Detecting the B-profile in lung ultrasound is highly sensitive and specific for elevated left ventricular diastolic pressures in patients with acute pulmonary oedema.

